# Discovery of BAY-405: An Azaindole-Based MAP4K1 Inhibitor for the Enhancement of T-Cell Immunity against Cancer

**DOI:** 10.1021/acs.jmedchem.4c01325

**Published:** 2024-09-27

**Authors:** Jeffrey Mowat, Rafael Carretero, Gabriele Leder, Nuria Aiguabella Font, Roland Neuhaus, Sandra Berndt, Judith Günther, Anders Friberg, Martina Schäfer, Hans Briem, Marian Raschke, Hideki Miyatake Ondozabal, Bernd Buchmann, Ulf Boemer, Bertolt Kreft, Ingo V. Hartung, Rienk Offringa

**Affiliations:** † Bayer AG, Pharmaceutical R&D, 13342 Berlin, Germany; ‡ DKFZ-Bayer Joint Immunotherapeutics Laboratory, German Cancer Research Center, Heidelberg 69120, Germany; § Division of Molecular Oncology of Gastrointestinal Tumors, Department of Surgery, University Hospital Heidelberg, Heidelberg 69120, Germany

## Abstract

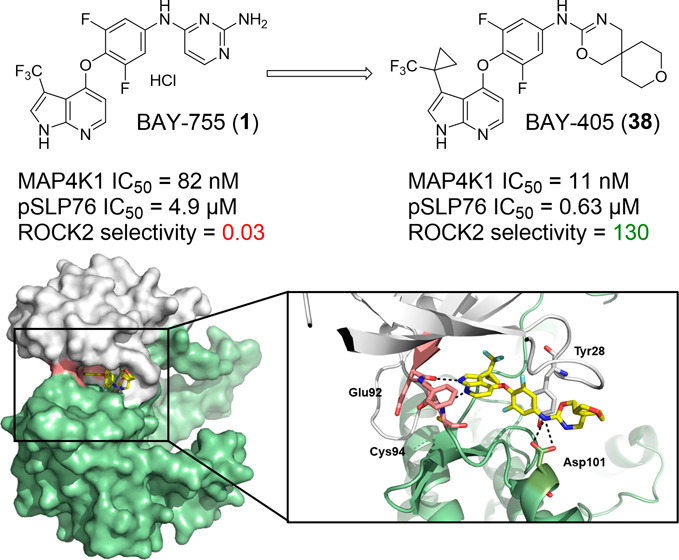

Mitogen-activated protein kinase kinase kinase kinase 1 (MAP4K1) is a serine/threonine kinase that acts as an immune checkpoint downstream of T-cell receptor stimulation. MAP4K1 activity is enhanced by prostaglandin E2 (PGE2) and transforming growth factor beta (TGFβ), immune modulators commonly present in the tumor microenvironment. Therefore, its pharmacological inhibition is an attractive immuno-oncology concept for inducing therapeutic T-cell responses in cancer patients. Here, we describe the systematic optimization of azaindole-based lead compound **1**, resulting in the discovery of potent and selective MAP4K1 inhibitor **38** (BAY-405) that displays nanomolar potency in biochemical and cellular assays as well as in vivo exposure after oral dosing. BAY-405 enhances T-cell immunity and overcomes the suppressive effect of PGE2 and TGFβ. Treatment of tumor-bearing mice shows T-cell-dependent antitumor efficacy. MAP4K1 inhibition in conjunction with PD-L1 blockade results in a superior antitumor impact, illustrating the complementarity of the single agent treatments.

## Introduction

The advent of immune checkpoint inhibitor (ICI) antibodies targeting the cell surface-expressed proteins CTLA-4, PD-1, and PD-L1 has profoundly broadened the scope of cancer drug discovery and prompted a search for complementary immunostimulatory strategies that can further increase the efficacy of immuno-oncology regimens.^1,2^ The latter is important because the majority of cancer indications are refractory to ICI treatment, in line with the notion that immune-subversion by the tumor microenvironment (TME) involves multiple mechanisms. A druggable target of particular interest in this respect is mitogen-activated protein kinase kinase kinase kinase 1 (MAP4K1), also known as hematopoietic progenitor kinase 1 (HPK1), a serine/threonine kinase expressed exclusively in hematopoietic cell lineages. Through its proline-rich domain, MAP4K1 binds to a diversity of adaptors in hematopoietic cells, including those involved in T-cell receptor (TCR), B-cell receptor, and cytokine-induced signaling.^3−6^ The function of MAP4K1 has been studied most extensively in the context of T-cell activation. Upon TCR stimulation, MAP4K1 is phosphorylated on tyrosine 381 (Y-381; Y-379 in mouse).^7^ Consequently, MAP4K1 is recruited to the TCR-signaling complex, where it induces dissociation of this complex through its serine/threonine kinase function, in particular by phosphorylating the SLP76 adaptor protein at Serine-376. In this manner, it acts as a negative feedback on TCR-downstream signaling.^4,5,7−9^ Notably, MAP4K1 can be triggered to suppress T cell function by prostaglandin E2 (PGE2) and transforming growth factor β (TGFβ), immune modulators that are commonly found in the tumor microenvironment.^10,11^ This suggests that pharmacological inhibition of MAP4K1 may offer a strategy to enhance antitumor T-cell immunity in a manner complementary to ICI antibodies. Accordingly, mice deficient for MAP4K1, or expressing a kinase-dead variant of MAP4K1, exhibit enhanced T-cell function, including increased antitumor immunity.^6,7,11−13^ These mice show an apparent normal phenotype, are fertile, and do not show deficiencies in normal lymphocyte development,^6,13^ supporting the notion that selective inhibition of this pathway may offer a therapeutic window for the enhancement of the antitumor T-cell response in cancer patients.

Numerous campaigns have been conducted to identify potent and selective inhibitors of MAP4K1, as illustrated by the abundance of disclosures in academic articles and patent applications covering a diversity of highly differentiated chemical scaffolds.^1,14−16^ Whereas several of these compounds have shown promising initial results in preclinical models, effectively combining kinase selectivity, potency, and pharmacokinetic properties remains a major challenge for this target. We therefore initiated the search for a selective MAP4K1 inhibitor by a high-throughput screen that resulted in several hits, including azaindole-based lead compound **1**. Although **1** displayed significant MAP4K1 inhibitory activity, kinase selectivity and DMPK properties were poor. Through iterative structure–activity relationship (SAR) studies, optimization efforts led to the discovery of compound **38** (BAY-405), which is a highly potent and selective inhibitor of MAP4K1. **38** exhibits favorable drug-like properties, achieves meaningful exposure in vivo, and is capable of inducing potent antitumor T-cell immunity in vivo.

## Results and Discussion

### High Throughput Screen and Initial Hit-to-Lead Process

**Figure 1:**
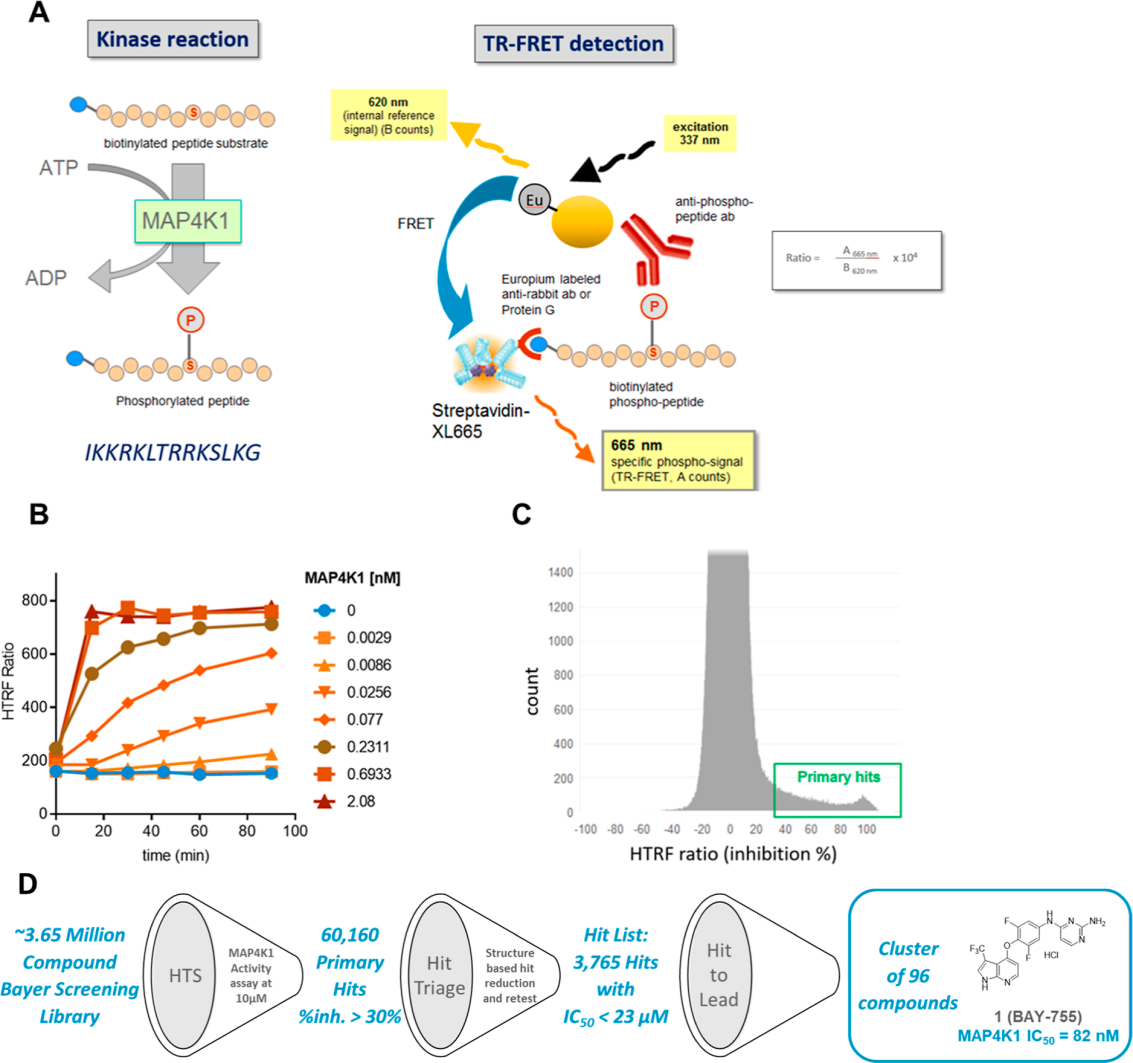
(A) Schematic representation of the high throughput biochemical kinase assay that is based on the phosphorylation of a synthetic peptide substrate (40 nM), representing IKKRKLTRRKSLKG, by recombinant human MAP4K1 protein in the presence of 10 μM ATP and 10 μM compound, where the quantity of phosphorylated substrate is measured by means of HTRF (Homogeneous Time Resolved Fluorescence). (B) Determination of assay linearity with respect to the concentration of human MAP4K1 protein and incubation time, based on which 77pM MAP4K1 and an incubation period of 60 min were selected. (C) Efficacy distribution of the 60,160 primary hits based on % inhibition in the primary screen. (D) High-throughput screen hit triaging resulted in the identification of one priority cluster represented by screening hit **1** containing a pyrrolo-pyridine as hinge binding motif. This binding mode features two hydrogen bonds and has been experimentally characterized by more than 150 different Protein Data Bank (PDB) entries in KLIFS.

To identify a small molecule inhibitor against MAP4K1, we developed and validated a biochemical kinase assay using purified, recombinant human GST-MAP4K1 fusion protein (Figure 1A,B). A diverse library of ∼3.65 million compounds was screened at 10 μM in the presence of 40 nM peptide substrate and 10 μM ATP, leading to the identification of 60,160 primary hits representing compounds inhibiting the enzyme by at least 30% (Figure 1C,D). Structure-based hit reduction and retesting in the biochemical assay rendered 4497 compounds for IC_50_ determination, resulting in a hit list of 3765 compounds exhibiting an IC_50_ of 23 μM or lower. In view of the importance of pharmacological testing in preclinical mouse models, we also determined the IC_50_ for the mouse orthologue of MAP4K1 at this early stage. This revealed excellent correlation of activity between the human and mouse targets for the majority of compounds, in line with a high degree of sequence identity (94%) across the full-length protein and identical amino acid residues in the ATP binding site (Supporting Information Figure S1). Further hit evaluation, including analysis of physicochemical properties, kinase selectivity testing, profiling in Bayer’s in silico ADMET platform,^17^ and final structural clustering, resulted in the selection of a priority cluster of 96 compounds exemplified by lead compound **1** (BAY-755) (Figure 1D). This compound displayed significant activity in a kinase inhibition assay against human MAP4K1 (IC_50_ = 82 nM), in accordance with its capacity to bind to this target in a tracer binding competition assay (IC_50_ = 75 nM) (Supporting Information Table S1).

Compound 1 belongs to a compound class known to inhibit the Rho-associated family of protein kinases (i.e., ROCK1 and ROCK2).^18^ Strong inhibition of these kinases was confirmed experimentally (Supporting Information Table S2). An early objective of our efforts was therefore to achieve sufficient Rho family selectivity, as the inhibition of these targets is implicated in unfavorable cardiovascular side-effects.^19^**1** displayed single digit nanomolar potency against ROCK2 (IC_50_ = 2 nM), representing a potency difference of approximately 40-fold as compared to MAP4K1 (Table 1). Given the absence of X-ray structural data for **1** with ROCK2, and the fact that the two kinases share a methionine gatekeeper, the C3 position of the azaindole pointing to the back wall of the ATP pocket was not the first choice to modulate ROCK selectivity. Hence, our initial efforts to identify potential handles to influence ROCK2 selectivity focused on the pyrimidine and the difluorophenyl (A-ring) moiety (Table 1). We found that replacement of the pyrimidine with ureas such as **2** increased potency 10-fold, delivering single digit nanomolar inhibitors (IC_50_ = 7 nM). Protein X-ray crystallography confirmed that the urea moiety interacts in a bidentate hydrogen bond with the Asp101 side chain in MAP4K1 (Asp112 in the surrogate kinase MST1). Despite this increase in potency, selectivity toward ROCK2 remained a challenge. We probed the influence of the A-ring through the synthesis of derivatives **3**–**5**. Removal of a single fluorine atom (e.g., **3**) resulted in a roughly 2-fold decrease in potency, while removal of the second fluorine (e.g., **4**) resulted in a further 2-fold decrease. The arrangement of the fluorine atoms also played an important role, as the 2,5-difluoro-isomer **5** showed a 4-fold reduction in potency as compared to **2**. Additionally, we observed that these modifications to the A-ring led to decreases in metabolic stability (Table 1), suggesting that the 2,6-difluoro substituted A-ring was important for potency as well as pharmacokinetic (PK) properties. Synthesis of the aza-indazole (not shown) resulted in a 10-fold decrease in potency compared with **2**, indicating a preference for the azaindole with respect to potency. Ureas synthesized from cyclic amines, which reduced overall H-bond donors by one (not shown), also displayed more than a 10-fold decrease in activity, highlighting the importance of the two hydrogen bonds for overall potency. Selectivity toward ROCK2 remained poor, while selectivity for other tested kinases (e.g., IRAK4 and MST1, Table 1) decreased or remained unchanged.

**Table 1:**
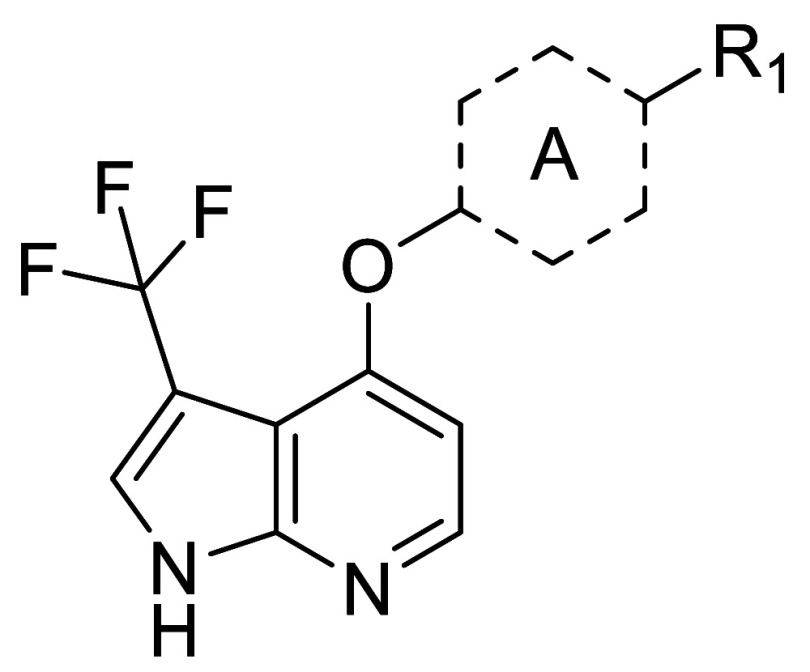

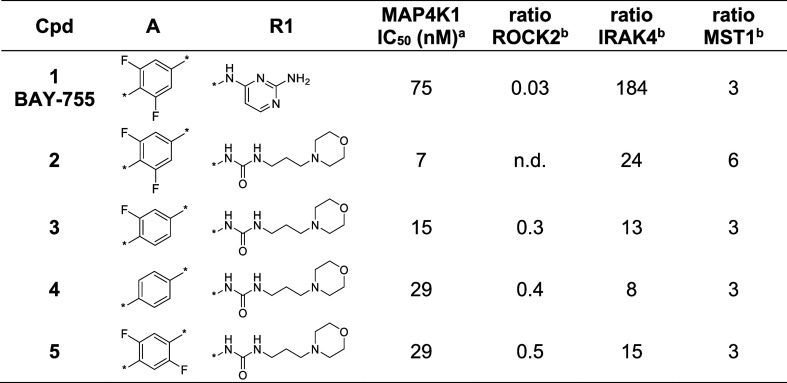
SAR Studies around Hit Compound **1** and the A-Ring

a Reported IC_50_ values from MAP4K1 binding competition assay are average values of at least two independent measurements according to the methods supplied in the Supporting Information.

b Selectivity expressed as the ratio of ROCK2, IRAK4, or MST1 IC_50_ over MAP4K1 IC_50_.

Investigation of the DMPK properties of this structural class of compounds in rat PK studies (Table 2) revealed that **2** displayed a moderate clearance in vivo and poor bioavailability (*F* = 8%). With the aim to improve PK parameters, in addition to probing for further opportunities to influence ROCK2 selectivity, alterations to the urea side chain were synthesized. Reducing the basicity of the morpholine by conversion to the corresponding amide **6** resulted in comparable MAP4K1 activity to **2**. In vivo clearance for **6** was reduced by more than 4-fold and bioavailability increased to 28%. Side chains carrying aromatic moieties (e.g., **7**) were also tolerated (IC_50_ = 9 nM), however clearance in vivo was nearly 3 times higher than for **6** and bioavailability suffered significantly (*F* = 2%). Investigation of 5-membered heterocycles, which preserved the two H-bond donor arrangement, rendered encouraging in vivo parameters, but potency was less favorable with **8** displaying an IC_50_ of 161 nM toward MAP4K1. Compounds carrying ether side chains (e.g., **9**) provided a balanced overall profile similar to that of **6**, indicating potential for further exploration. Surprisingly, when oxetane-containing side chains were installed, spontaneous acid-mediated rearrangement of the oxetane containing urea occurred in the final deprotection step of the synthetic route, resulting in the formation of oxazine **11** as a racemic mixture.^20^ This compound showed excellent potency (IC_50_ = 3 nM), but high clearance in vivo (CL_b_ = 3.7 L/h/kg) and moderate bioavailability (*F* = 26%). Importantly, **11** represented the first analogue with activity toward MAP4K1 greater than ROCK2, thereby providing first signs that selectivity may be possible. When the initially intended oxetane containing urea **10** was synthesized, a less favorable overall profile was obtained (Table 2). Encouraged by the properties of **11**, we synthesized fluorine containing oxetane **12**, which showed a comparable activity and selectivity profile, accompanied by reduced in vivo clearance (CL_b_ = 1.3 L/h/kg) and improved bioavailability (*F* = 69%). However, the overall kinase selectivity of this molecule, in addition to its stability profile, revealed significant challenges. Not only did **12** show instability under basic conditions due to the known lability of trifluoromethyl groups at the C3 position of azaindoles,^21^ but the oxazine moiety also displayed rapid degradation under acidic conditions (Table 3). Thorough analysis of the degradation products led us to postulate that protonation of the oxazine and addition of the hydroxymethyl group into the formed iminium ion led to a cascade of reactions that eventually resulted in the rearrangement of the oxazine skeleton, which was followed by further degradation and hydrolysis. In addition to challenges with hydrolytic stability, **12** also displayed plasma instability (Table 3). Most problematic though were cardiovascular toxicities driven by residual ROCK2 activity that were observed in an in vivo rat telemetry study, where **12** produced a dose-dependent decrease in mean arterial blood pressure (MAP) after a single dose (10, 15, 30% reduction compared to baseline in MAP at 15, 30, and 60 mg/kg of **12** respectively). In addition to these cardiovascular effects, several toxicities were also observed at low multiples in a 14 day repeat dose rat tolerability study for **12**. Furthermore, **12** showed a positive signal in the micronucleus test, a result which we attributed to the overall unfavorable kinase selectivity displayed by **12**, in addition to the lack of Rho kinase selectivity.^22,23^ With these parameters in mind, a further campaign was initiated with the goal of improving both the stability and the kinase selectivity profile of **12**.

**Table 2:**
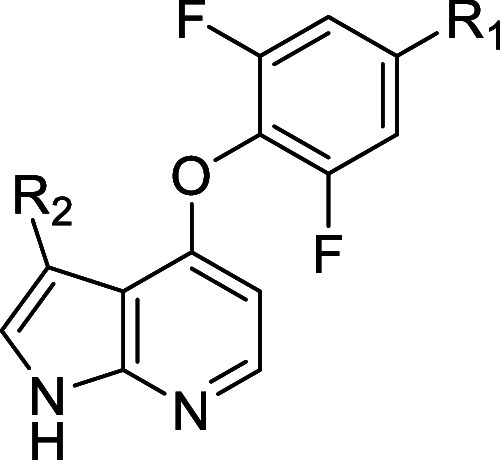

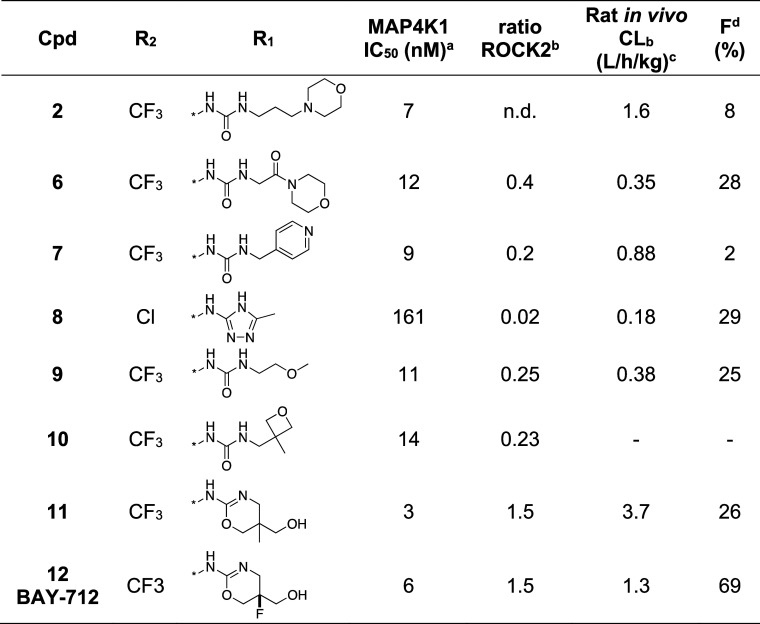
SAR and DMPK Studies of the Urea Moiety

a Reported IC_50_ values from MAP4K1 binding competition assay are average values of at least two independent measurements.

b Selectivity expressed as the ratio of ROCK2 IC_50_ over MAP4K1 IC_50_.

c *In vivo* clearance as determined from a single IV dose (0.4 mg/kg for compounds **2** and **6**, 0.3 mg/kg for compounds **7**, **8**, **9**, and **11**, 0.5 mg/kg for compound **12**) in male Wistar rat.

d Bioavailability after oral dosing (0.8 mg/kg for compounds **2** and **6**, 0.6 mg/kg for compounds **7**, **8**, **9**, and **11**, 1.0 mg/kg for compound **12**) in male Wistar rat.

**Table 3:**
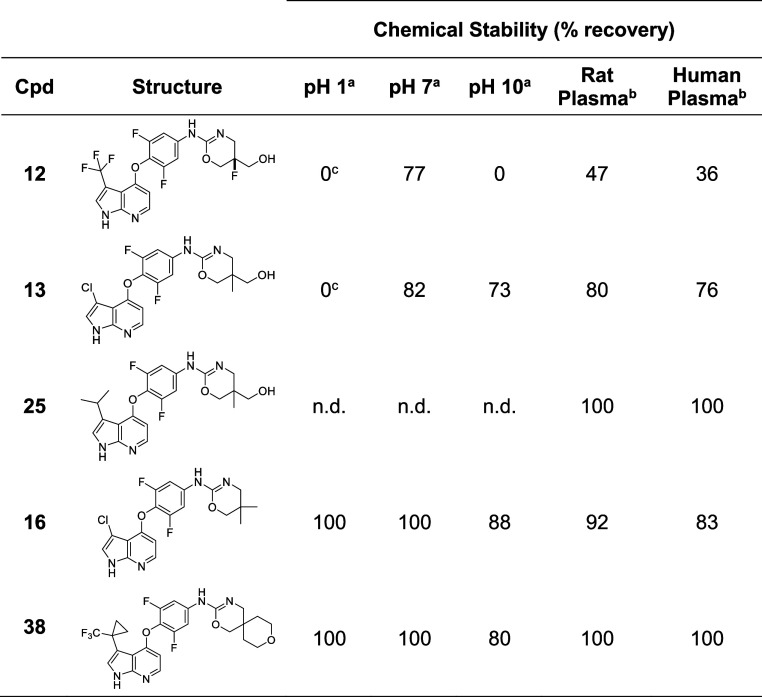
Hydrolytic and Plasma Stability of Diverse Oxazines

a % recovery recorded by HPLC-MS after 24 h of incubation at the indicated buffer pH at room temperature.

b % recovery recorded by HPLC-MS after 4 h of incubation at 37 °C.

c % recovery values recorded with HPLC-MS for **12** led to the erroneous perception of stability at pH 1. Samples analyzed by ^1^H NMR showed rapid degradation of parent signals at pH 1. not determined (n.d.).

### Lead Optimization

We started probing the SAR around the oxazine moiety (Table 4), working in matched molecular pairs/series,^24−26^ by synthesizing **13** with a chlorine at the C3 position of the azaindole (R_2_) as a benchmark compound. While displaying slightly lower activity and selectivity toward ROCK2 compared to **11**, this compound was associated with greater synthetic ease and increased stability in basic media. Methylation of the pendant hydroxy group on the oxazine produced **14**, which displayed activity comparable to that of **13**, indicating that the hydroxy group was not vital for activity and could therefore be replaced to potentially increase stability in acidic media. In vitro metabolic stability in rat hepatocytes for **13** was moderate (*F*_max_ = 36%). Both the naked oxazine (**15**) and a gem-dimethyl analogue (**16**) provided further evidence that oxazine substitution was not driving potency, despite both changes being associated with lower metabolic stability as compared to **13**. These results gave us confidence that significant flexibility existed for the oxazine substitution, and therefore the problematic hydroxymethyl could be replaced to increase hydrolytic stability. Oxazoline **17** displayed a >10-fold decrease in potency compared to **15**, highlighting the preference for the 6-membered ring. The thiazine **18** resulted in a similar decrease in potency, as did guanidine (IC_50_ = 34 nM) and amidine (IC_50_ = 44 nM), further supporting the notion that the oxazine was the preferred motif to preserve MAP4K1 potency. Fluorinated oxazine **19** resulted in excellent metabolic stability (*F*_max_ = 100%) but was associated with decreased activity compared with methylated analogues (e.g., **13** and **11**). Installation of a gem-difluoro group on the oxazine (**20**) led to a further decrease in potency compared to **15**, resulting in an IC_50_ of 167 nM. In all cases, ROCK2 selectivity remained relatively constant, although our data suggested that bulk on the oxazine could provide higher selectivity (e.g., **16** and **14** versus **15**). Taken together, these trends highlighted that oxazine substitution could be used to modulate metabolic stability by decreasing the electron density in the oxazine ring. However, installation of electron-withdrawing groups such as fluorine (**19**–**20**), but also CF_2_H, and CN (not shown), were associated with decreased activity and selectivity. In fact, with the exception of a guanidine ring, we observed that the calculated p*K*_a_ for a broad range of partially saturated heterocycles containing an imbedded imine (e.g., oxazine, thiazine, thiazoline, oxazoline, amidine) correlated well with biochemical potency, with reduced electron density (i.e., lower p*K*_a_) resulting in decreased activity. Cellular activity, as measured by a compound’s ability to inhibit phosphorylation of the signal-transducing adaptor protein SLP76, correlated well with data recorded from the binding competition assay, albeit with a shift in IC_50_ of roughly 100-fold. The discrepancy between the biochemical and cellular IC_50_ can partially be attributed to the absence of competing ATP in the binding competition assay as compared to the cellular context as well as the potential impact of protein binding in the assay medium for this chemical series.

**Table 4:**
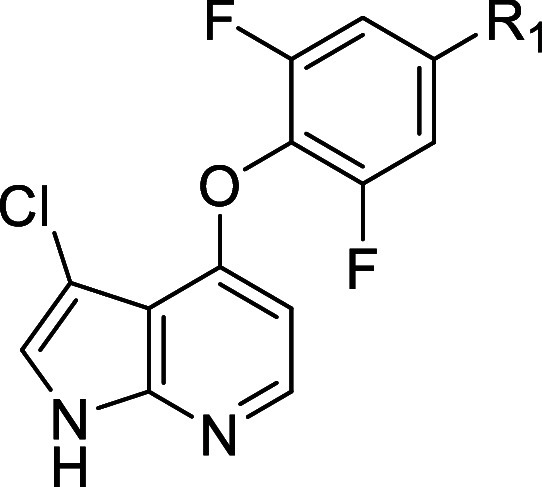

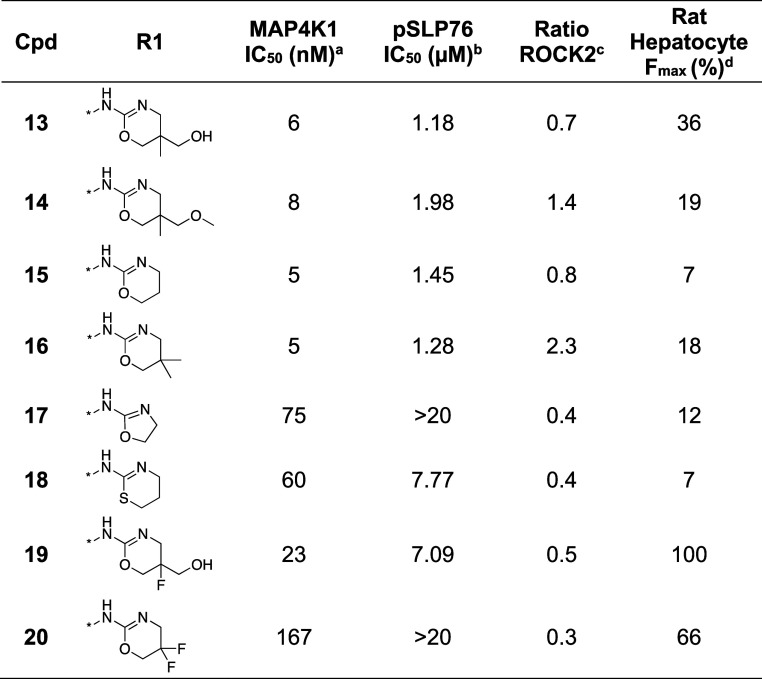
Initial SAR Studies of the Oxazine Moiety

a Reported IC_50_ values from MAP4K1 binding competition assay are average values of at least two independent measurements.

b HTRF-based SLP76 phosphorylation assay was performed in Jurkat human T cell line as described in the Experimental section.

c Selectivity expressed as the ratio of ROCK2 IC_50_ over MAP4K1 IC_50_.

d In vitro stability assays were performed as described in the Supporting Information.

We subsequently turned our attention to the C3 position of the azaindole, as we hypothesized that further modulation of both ADME and kinase selectivity properties could be realized via this gatekeeper facing vector. Protein-structure based computation methods were utilized whenever the selectivity against an off-target kinase appeared to be relevantly driven by changes in the amino acid composition of the ATP binding site. For other kinases such as ROCK2, where structural insights were less informative, we followed an empirical approach. The result of this exploration is summarized in Table 5. We synthesized a series of compounds containing small substituents, such as CF_3_ (**11**), CN (**22**), and Cl (**13**), at the C3 position. Apart from the naked azaindole, where R_2_ = H (**21**), which lost considerable activity, small substituents maintained low single digit nanomolar potency. Interestingly, larger substituents like CF_3_ (e.g., **11**) appeared to lead to greater selectivity toward ROCK2 than less sterically encumbered ones. Indeed, when bulky aryl substituents were installed (e.g., **23** and **24**), dramatic increases in selectivity toward ROCK2 were observed. Potency was maintained for **23** in both biochemical and cellular assays, but a moderate drop in potency for **24** was observed as compared to **23**. Unfortunately, due to poor physiochemical properties associated with bulky aryl substituent, very low bioavailability was observed in a rat PK experiment for compounds **23** and **24** (*F* % < 10%). Therefore, we focused on sp3-rich aliphatic substituents (**25**–**31**), which we reasoned could provide the steric bulk to influence ROCK2 selectivity while maintaining acceptable physiochemical and DMPK properties. Installation of isopropyl (**25**) resulted in excellent potency in biochemical (IC_50_ = 1 nM) and cellular (IC_50_ = 460 nM) assays and more than 10-fold selectivity versus ROCK2. This encouraging result gave us confidence that further selectivity increases could be achieved with this approach. **27** confirmed this trend, whereas the smaller cyclopropyl (**26**) showed lower selectivity, comparable to that of the CF_3_ (**11**). Introduction of the polar cyano residue on the cyclopropane (**28**), while maintaining the selectivity and potency of **27**, displayed improved metabolic stability, suggesting that the methyl substituent of **27** could be altered to influence metabolic stability. To this end, we synthesized fluorinated cyclopropanes **29** and **30**. While the profile of the difluoromethyl analogue **29** was similar to that of **27**, the installation of a trifluoromethyl group (e.g., **30**) was associated with increased selectivity (ratio ROCK2/MAP4K1 = 55) and metabolic stability (*F*_max_ = 56%). Although additional increases in selectivity could be realized by enlarging the ring to a cyclobutyl (e.g., **31**) or cyclopentyl (not shown), we found this to negatively affect metabolic stability.

**Table 5:**
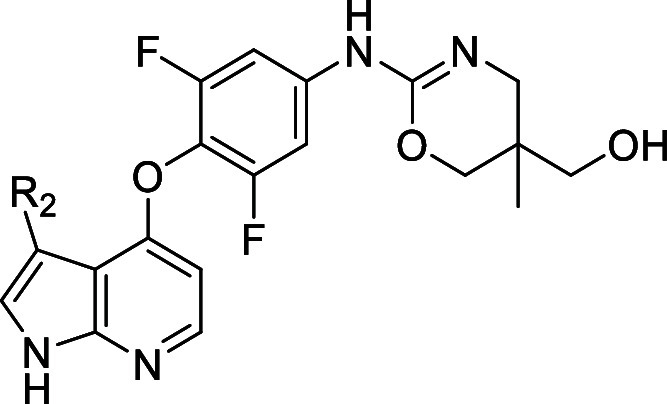

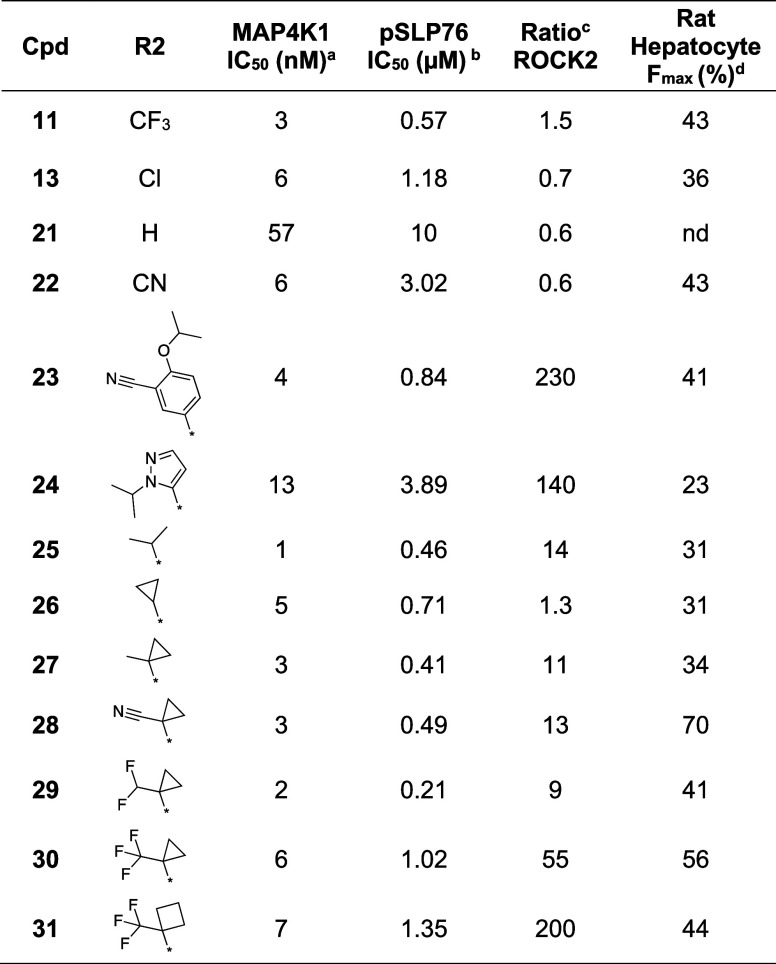
SAR Studies of the C3 Position of the Aza-Indole Core^e^

a Reported IC_50_ values from MAP4K1 binding competition assay are average values of at least two independent measurements.

b HTRF-based SLP76 phosphorylation assay was performed in Jurkat human T cell line as described in the Experimental section.

c Selectivity expressed as the ratio of ROCK2 IC_50_ over MAP4K1 IC_50_.

d In vitro stability assays were performed as described in the Supporting Information.

e All compounds were prepared and profiled as oxazine racemic mixtures.

With **30** representing what we believed to be an optimized azaindole C3 substituent for balancing potency, selectivity, and DMPK properties, we turned our attention back to the oxazine. With our previous SAR in mind, we reasoned that the choice of oxazine substitution could further influence potency and selectivity as well as metabolic stability. Since aliphatic substituents could increase selectivity toward ROCK2 (Table 4), we synthesized compounds **32**–**35**. Substitution of the 4-position of the oxazine (e.g., **32**, **34**, **35**) was associated with further increases in selectivity, whereas substitution of the 5-position (e.g., **33**) reduced potency (Table 6). While we generally observed good in vitro/in vivo correlations across the series, moderate stability in rat hepatocytes for **34** and **35** was accompanied by higher clearance in vivo and poor bioavailability. As selectivity toward ROCK2 appeared to improve with increasing size of the substituent on the oxazine (**34** vs **35**), we hypothesized that larger oxygen-containing cycles might maintain selectivity properties while increasing metabolic stability. Annulation of a tetrahydrofuran onto the oxazine resulted in compound **36**, which displayed a decrease in selectivity toward ROCK2 compared to **30**. Spiro-tetrahydrofuran **37** maintained good potency and selectivity compared with **34**; however, the corresponding in vivo PK was similar to **34**. Installation of a spiro-tetrahydropyran resulted in compound **38**, which showed further increases in potency and selectivity compared with **37**. To our delight, **38** was accompanied by an increase in metabolic stability in vitro, which translated into reduced in vivo clearance in rat (CL_b_ = 1.5 L/h/kg) and acceptable bioavailability (*F* = 38%).

**Table 6:**
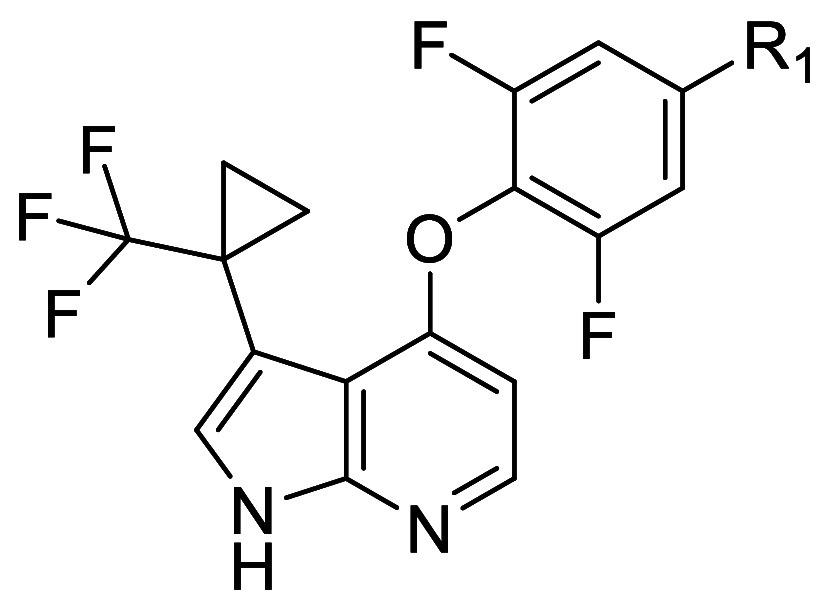

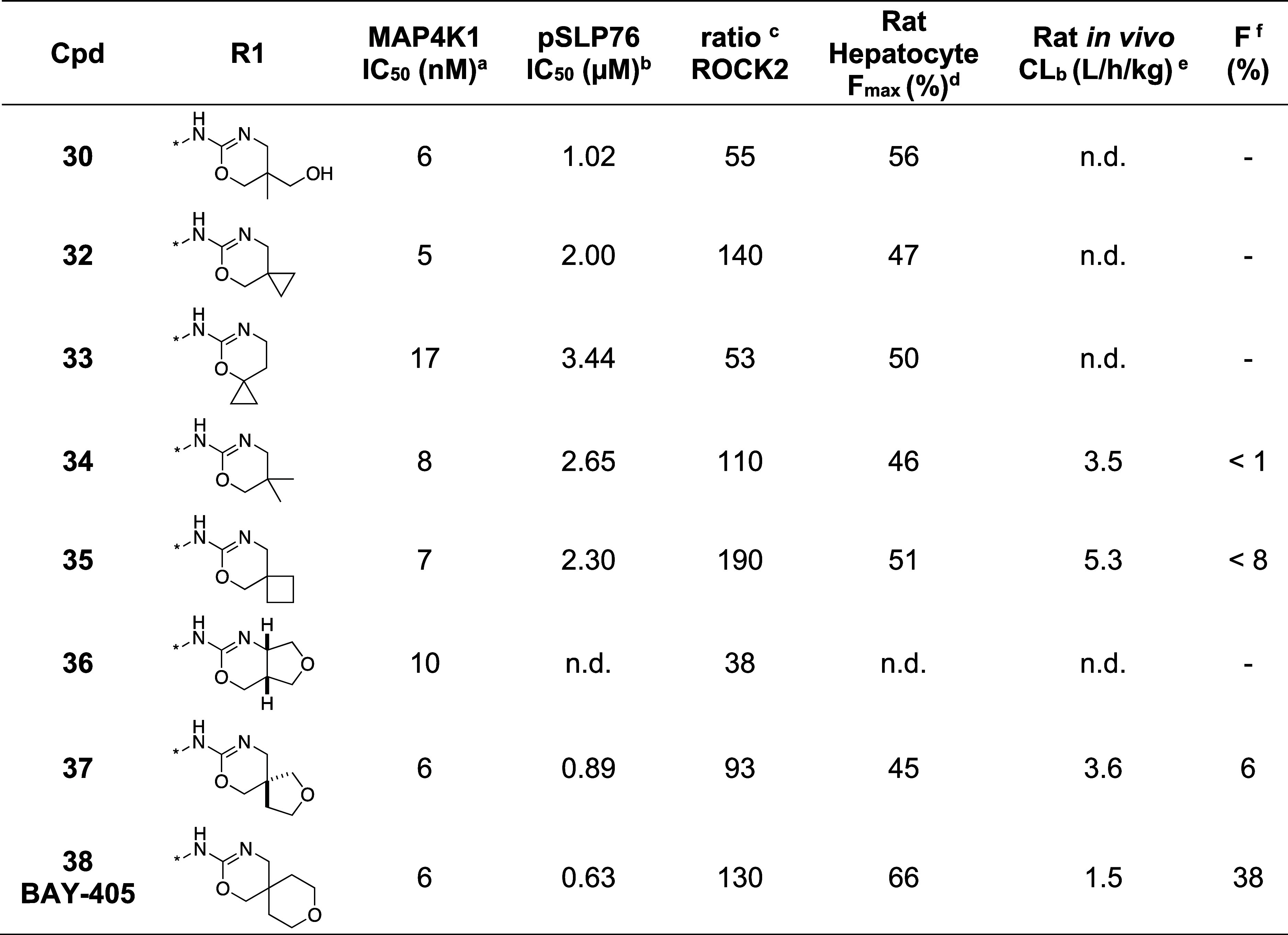
Optimization of Oxazine

a Reported IC_50_ values from MAP4K1 binding competition assay are average values of at least two independent measurements.

b HTRF-based SLP76 phosphorylation assay was performed in human primary PBMCs as described in the Experimental section.

c Selectivity expressed as the ratio of ROCK2 IC_50_ over MAP4K1 IC_50_.

d In vitro stability assays were performed as described in the Supporting Information.

e In vivo clearance as determined from a single IV dose (0.3 mg/kg for compounds **34**, **35**, and **37**, 1.0 mg/kg for compound **38**) in male Wistar rat.

f Bioavailability recorded after oral dosing (0.6 mg/kg for compounds **34**, **35**, and **37**, 1.0 mg/kg for compound **38**) in male Wistar rat.

With a broad range of azaindole C3 and oxazine substitutions at our disposal, we assessed the plasma and hydrolytic stability of a range of optimized analogues (Table 3). As previously noted, **12** showed poor hydrolytic stability across a range of pH values due to the labile CF_3_ and oxazine moiety. Replacement of the base labile CF_3_ group markedly improved plasma stability as well as hydrolytic stability at pH 10 (e.g., **13**). Unexpectedly, we found that C3 substitution on the azaindole was a primary driver of plasma stability, with **25** and other aliphatic substitutions showing excellent stability in rat and human plasma compared to compounds containing electron-withdrawing groups (e.g., **12** and **13**). Removal of the hydroxymethyl group (e.g., **16**) resulted in a significant increase in chemical stability at neutral and acidic pH with only minor hydrolytic instability observed at pH 10 (88% remaining after 24 h incubation). ^1^H NMR analysis of solutions of **16** containing HCl displayed an overall sharpening of the signals and a new peak at 11.4 ppm, both effects that we associated with the protonation of the oxazine and stable salt formation. No visible changes in the NMR spectrum were observed after 24 h, confirming the increased stability in acidic media. Finally, **38** demonstrated an encouraging stability profile, displaying excellent stability at pH 1 and 7, minor instability at pH 10, and no degradation in rat and human plasma.

### Profiling of BAY-405

**Figure 2:**
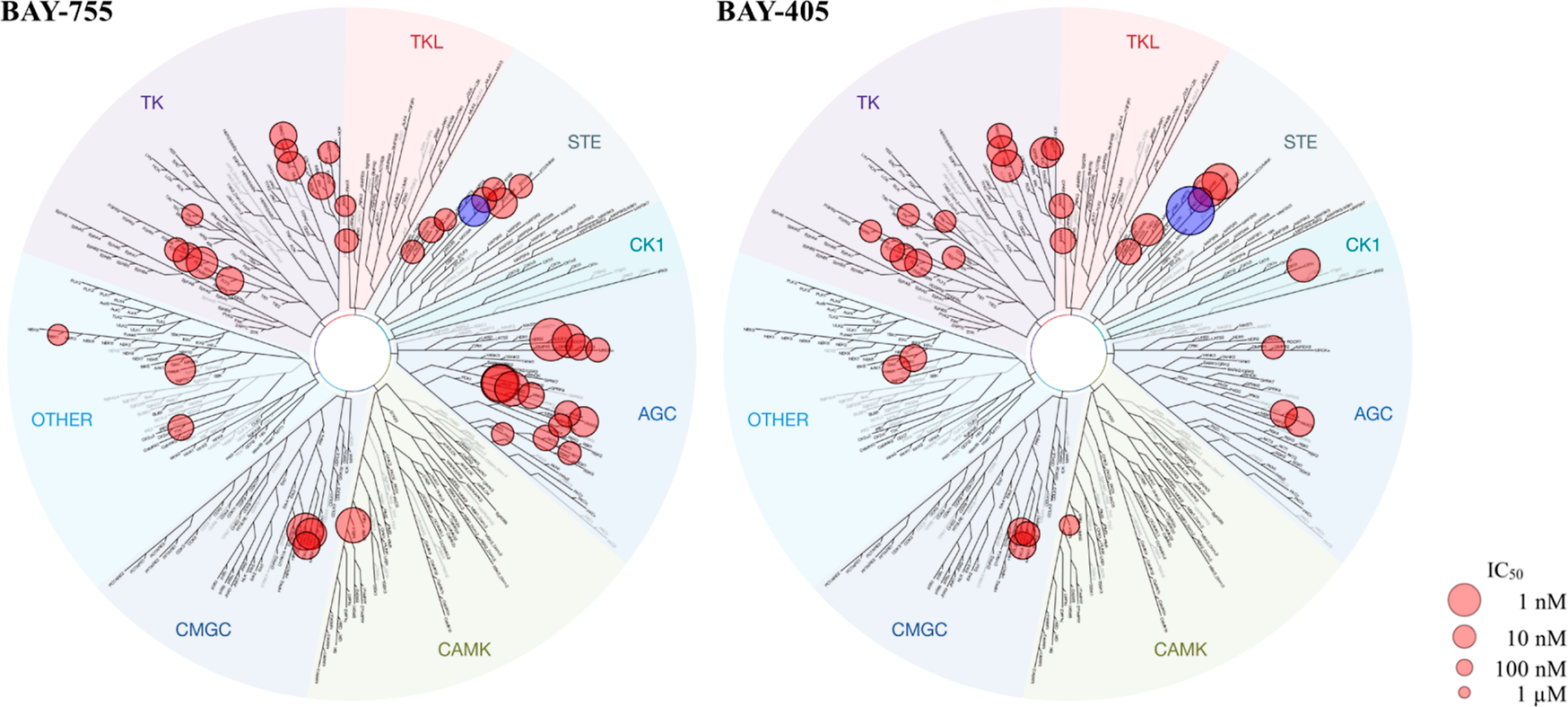
Kinome tree of **1** (BAY-755) and **38** (BAY-405) representing IC_50_ values recorded for all kinases with % inhibition >80% at 1 μM. MAP4K1 is represented by the blue dot. Total number of kinases tested for **1** (*n* = 318) and **38** (*n* = 373). Image generated using TREEspot Software Tool and reprinted with permission from KINOMEscan (DiscoveRx Corp., Fremont, USA).

As **38**, hereinafter referred to as BAY-405, represented our most promising candidate with respect to potency, selectivity, DMPK parameters, as well as plasma and chemical stability, we progressed this compound into further in vitro and in vivo profiling as summarized in Table 7. BAY-405 displayed potent activity toward MAP4K1 in biochemical and cellular assays, with a ROCK2 selectivity ratio of 130 in the biochemical assay and an overall selectivity score of *S* = 0.080 (80%, 1 μM)^27^ against an external kinase panel consisting of 373 kinases, representing a significant improvement over compound **1** (BAY-755; *S* = 0.13; Figure 2, Supporting Information Tables S2 & S3). Excellent selectivity was realized against a range of kinases involved in cell cycle regulation, whose inhibition may be associated with decreased ability of T-cells to efficiently proliferate (e.g., CDKs, PLKs, Aurora kinases, checkpoint kinases^28^). Furthermore, good selectivity was observed against kinases involved in TCR signaling (e.g., ZAP70, Lck, Fyn, Itk, Jak1/2/3^29^), an important prerequisite for immune cell activation. In this respect, a potential liability was the impact of BAY-405 on MAP4K3, a kinase closely related to MAP4K1 for which studies in knockout mice had indicated a role in the T-cell immune response,^12^ and for which a modest selectivity ratio of 6.5 was observed (Supporting Information Table S4).

**Table 7:** Profile of Compound **38** (BAY-405)

	38 (BAY-405)
MAP4K1 IC_50_ (nM)^a^	6 (h); 13 (m); 17 (r); 19 (cyno)
pSLP76 IC_50_ (μM)^b^	0.63
ratio ROCK2^c^	130
selectivity score (80% inh., 1 μM)^d^	0.08
log *D* at pH 7.5	2.8
solubility (mg/L) PBS pH 6.5	0.3
microsome stability *F*_max_(%)	70 (m); 77 (r); 72 (d); 46 (h); 42 (cyno)
hepatocyte stability *F*_max_(%)	61 (m); 66 (r); 41 (d); 35 (h); 36 (cyno)
Caco-2 permeability: P_app_: A-B [nm/s] (ER)	7.4 (5.1)
CYP Inh. IC_50_ (μM) (3A4/2D6/2C9/2C8/1A2)	>20/>20/13/13/>20
PXR MEC (μM)^e^	12
CYP 3A4 induction NOEL^f^ (μM)	0.75
plasma protein binding *f*_unbound_ (%)^g^	3.49 (h); 1.39 (m); 2.52 (r); 0.75 (d)
hERG IC_50_ (μM)	4.9
micronucleus test (MNT)	negative
AMES test	negative
In Vivo Pharmacokinetics	
CL_b_ (L/h/kg)^h^	2.0 (m), 1.5 (r), 1.3 (d)
V_ss_ (L/kg)^h^	6.9 (m), 7.0 (r), 3.0 (d)
*t*_1/2_ (h)^h^	4.0 (m), 3.8 (r), 3.3 (d)
*F* (%)	n.d. (m), 16^i^-38^j^ (r), 9 (d)^k^

a Reported IC_50_ values from MAP4K1 binding competition assay are average values of at least two independent measurements.

b HTRF-based SLP76 phosphorylation assay was performed in human primary PBMCs as described in the Experimental section.

c Selectivity expressed as the ratio of ROCK2 IC_50_ over MAP4K1 IC_50_.

d Selectivity score expressed as the ratio of the number of kinases with % inhibition values recorded at 1 μM greater than 80% (*n* = 30) over the total number of kinases tested (*n* = 373).

e Human pregnane xenobiotic receptor (PXR) nuclear receptor activation assay was performed as described in the Supporting Information.

f CYP 3A4 induction was assessed in human hepatocytes. mRNA expression no observed effect levels (NOEL) are reported as described in the Supporting Information.

g Plasma protein binding was measured by equilibrium dialysis as described in the Supporting Information.

h In vivo clearance (CL_b_), volume of distribution (*V*_ss_), and half-life (*t*_1/2_) parameters were recorded after a single intravenous administration of BAY-405 to male Wistar rat [1.0 mg/kg i.v. bolus in plasma/EtOH/DMSO (95/4/1)], female CD1 mouse [1.0 mg/kg i.v. bolus in PEG400/water/EtOH (50/45/5)], or female Beagle dog [0.5 mg/kg i.v. infusion of 10 min in water/PEG400/EtOH (50/40/10)].

i Bioavailability after oral administration of 0.6 mg/kg BAY-405 in a solution of water/solutol/EtOH (50/40/10) to male Wistar rat.

j Bioavailability after oral administration of 1.0 mg/kg BAY-405 in a solution of water/solutol/EtOH (50/40/10) to male Wistar rat.

k Bioavailability after oral administration of 1.0 mg/kg BAY-405 in a solution of water/solutol/EtOH (50/40/10) to female Beagle dog. Cynomolgus monkey (cyno); dog (d); efflux ratio (ER); human (h); human ether-a-go-go related gene (hERG); minimum effective concentration (MEC); mouse (m); rat (r).

The binding of BAY-405 with submicromolar *K*_d_ was confirmed in a cell lysate proteomics-based kinase selectivity assay^30^ (Supporting Information Table S5). BAY-405 was confirmed as an ATP competitive active site inhibitor, as demonstrated by the fact that both kinase inhibitor (IC_50_ = 11 nM) and ATP binding competition assays (IC_50_ = 6.2 nM) rendered highly comparable IC_50_ values, and further supported by a prominent shift in IC_50_ value in a high ATP kinase activity assay (IC_50_ = 56 nM at 1 mM ATP). BAY-405 showed species cross-reactivity with highly comparable potency against the monkey, rat, and murine MAP4K1 orthologues. Furthermore, binding constants recorded by Biacore surface plasma resonance (SPR) analysis utilizing the MAP4K1 kinase domain were in good agreement with data from the biochemical assay (Supporting Information Table S1). With respect to cellular activity, BAY-405 inhibited phosphorylation of SLP76 with submicromolar activity (IC_50_ = 0.63 μM; Supporting Information Table S6). Further profiling of BAY-405, as summarized in Table 7, showed that the solubility in phosphate-buffered saline (PBS) at pH 6.4 was low (0.3 mg/L), and logD at pH 7.5 was 2.8, which represented a somewhat higher lipophilicity range for this chemical series. Metabolic stability was measured across a range of preclinically relevant species and good correlation was found between both microsomal and hepatocyte assays. BAY-405 showed overall moderate to good stability, with lower turnover in rodent (e.g., mouse and rat) compared with higher order species (e.g., dog, monkey, and human). Caco-2 permeability was low (*P*_app_ (A → B) = 7.4 nm/s), with a moderate ER of 5. The CYP inhibition profile was favorable, with weak inhibition observed for both 2C8 and 2C9 CYP isoforms (IC_50_ = 13 μM for both enzymes). PXR transactivation was observed, which translated into a CYP 3A4 induction NOEL of 0.75 μM (Table 7). Even before a binding conversion factor is accounted for, which is likely to exacerbate the induction NOEL, the narrow selectivity window against MAP4K1 cellular activity could limit the further development of this lead. BAY-405 displays micromolar binding to the hERG channel (IC_50_ = 4.9 μM), with an estimated IC_20_ of ∼1.5–2.0 μM. Notably, the potential hERG selectivity window as compared to the unbound cellular pSLP IC_50_ (Supporting Information Table S9) is projected as greater than 10 and is therefore considered a minor risk. Despite these attributes, the compound was negative in the micronucleus and AMES test, a testament to the increased kinase selectivity realized over the course of medicinal chemistry optimization. The in vivo pharmacokinetic profile was analyzed in mouse, rat, and dog (Table 7). This revealed moderate clearance, high volume of distribution, and a long half-life in rodents. Higher clearance and a shorter half-life were observed in dog, in line with in vitro findings. Bioavailability was low to moderate in rat (*F* = 16–38%) and low in dog (*F* = 9%). The lower bioavailability observed in dog compared to rat may be a result of the differential pH in dog versus rat stomach, resulting in less protonation of the basic oxazine and an overall reduced solubility. Taken together, however, these data supported in-depth in vitro and in vivo biology profiling of BAY-405.

**Figure 3:**
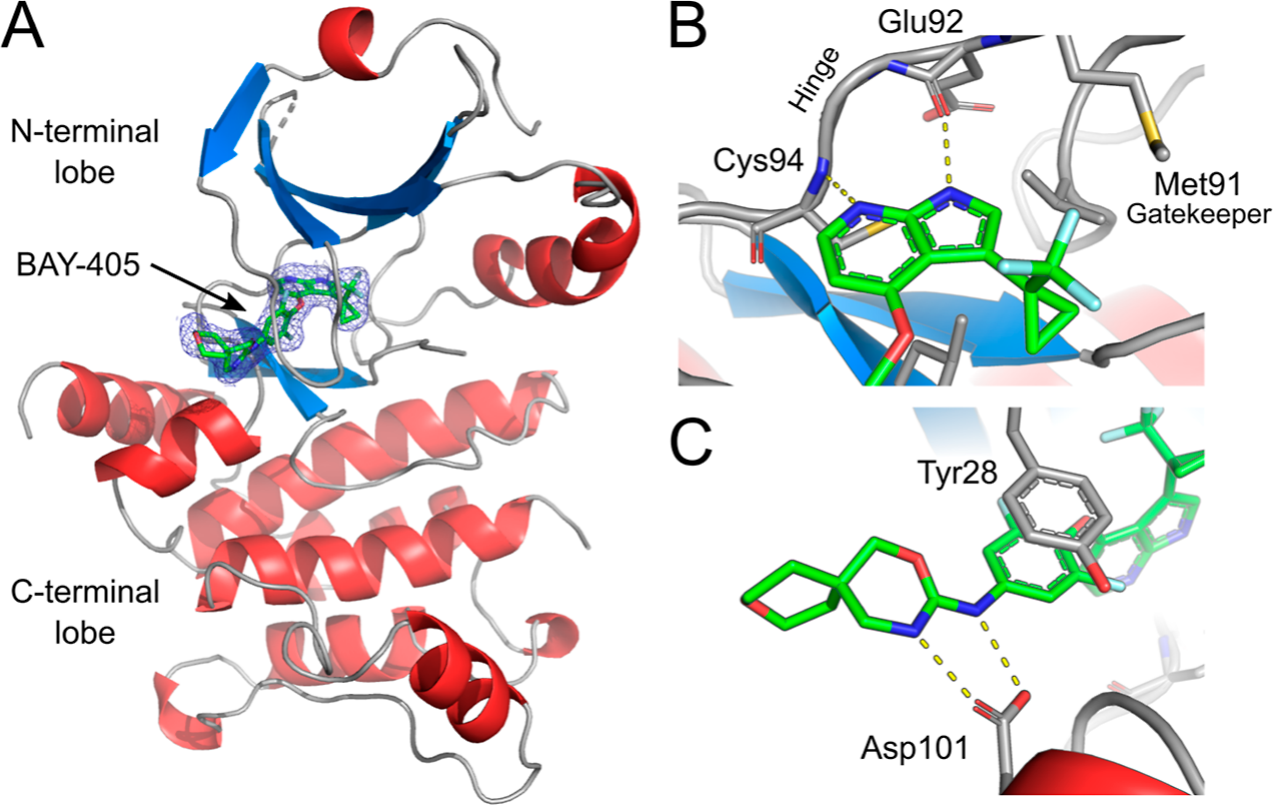
Co-crystal structure of human MAP4K1 in complex with **38** (PDB accession code 8PAR). (A) Overview of BAY-405 (green) bound to the ATP site of human MAP4K1. The electron density of the bound inhibitor is shown at a sigma of 1.0 (blue mesh). Protein secondary structures are designated in red (alpha-helices) and blue (beta-strands). (B) Hinge-binding interactions of the aza-indole of BAY-405. (C) Hydrogen bonding interactions of the oxazine of BAY-405 to Asp101.

The X-ray structure of BAY-405 in complex with the kinase domain of human MAP4K1 shows an ATP-competitive binding mode, with the pyrrolopyridine anchoring the molecule to the hinge region via two classical hydrogen bonds (Figure 3, Supporting Information Table S8). The trifluoromethyl-cyclopropyl moiety sits smoothly in the back pocket of the kinase, which remains in a DFG-in conformation. The central fluorinated phenyl ring (A-ring) is arranged perpendicular to the hinge binding heterocycle, with the conformation being stabilized by the *o*,*o*-disubstitution. The tip of the nucleotide binding loop folds back into the pocket, allowing for a π–π-stacking interaction of the A-ring with the side chain of Tyr28. While the oxazine moiety engages in a bidentate H-bond with the Asp101 carboxylic acid, the terminal tetrahydropyran ring in the spiro-ring system does not show any direct interactions with the crystallized kinase domain.

### In Vitro Pharmacology of BAY-405

**Figure 4:**
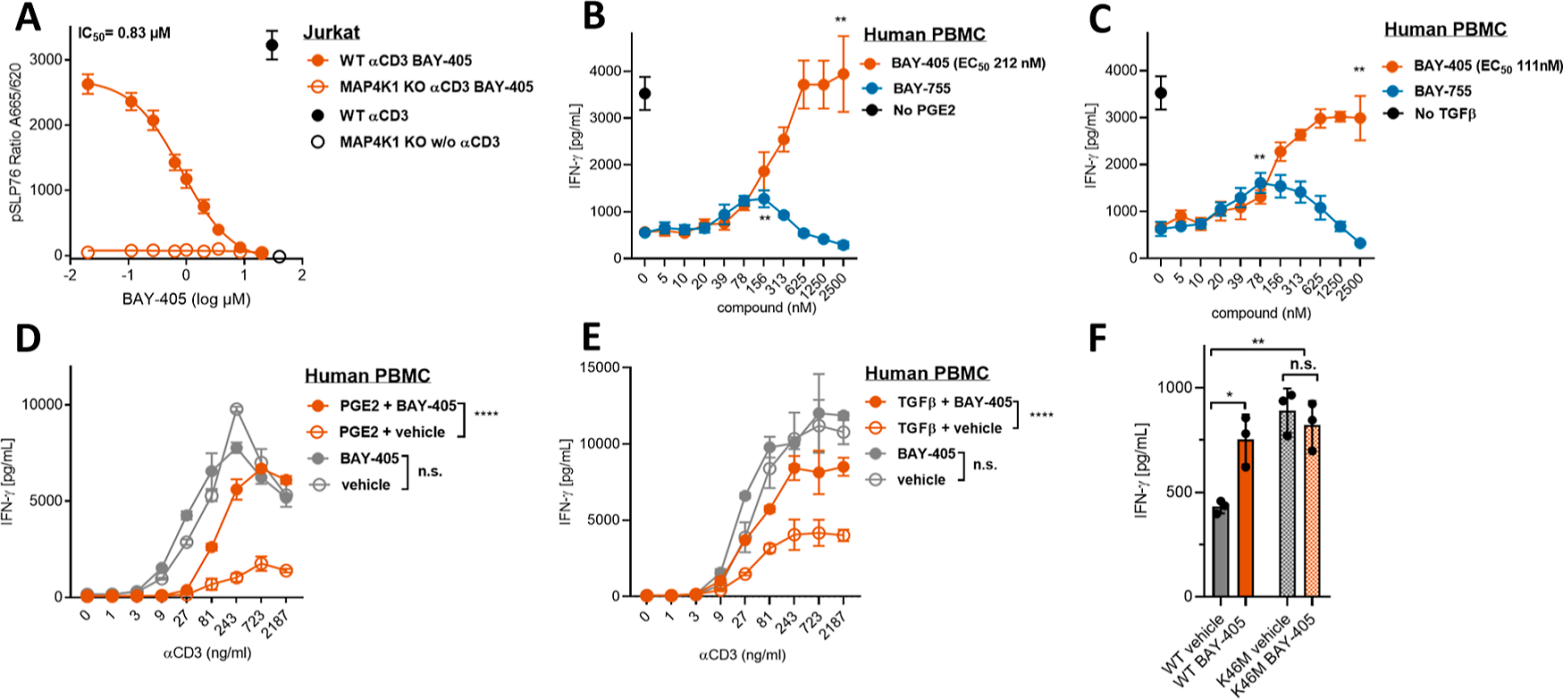
In vitro Pharmacology of BAY-405. (A) Inhibition of MAP4K1 activity in Jurkat T-cells by BAY-405 as measured on the basis of the intracellular levels of phospho-SER376-SLP76 (pSLP76), using MAP4K1 knockdown Jurkat T-cells as controls (Supporting Information Figure S2A). Jurkat T-cells were stimulated with plate-bound anti-CD3 Abs (1 mg/mL) for 30 min after which the pSLP76 levels were determined by a HTRF-based method. (B) Dose-dependent enhancement of T-cell reactivity by BAY-405 and its predecessor BAY-755 in human PBMC cultures stimulated with 30 ng/mL anti-CD3 Ab in the presence of 1 μM PGE2. Secreted IFNγ was analyzed after 24 h by means of ELISA. 2500 nM BAY-405 vs vehicle *p* = 0.0024; 156 nM BAY-755 vs vehicle *p* = 0.0059 (student *t*-test). (C) T-cell reactivity assay as in (B), but performed in the presence of 20 ng/mL human TGFβ. 2500 nM BAY-405 vs vehicle *p* = 0.0012; 156 nM BAY-755 vs vehicle *p* = 0.0028 (student *t*-test). (D,E) T-cell assays as in (A,B) showing the impact of 1 μM BAY-405 on T-cell activation in the presence of different concentrations of anti-CD3 Ab as well as in the presence and absence of 1 μM PGE2 or 20 ng/mL human TGFβ. PGE2 + BAY-405 vs PGE2 + vehicle *p* < 0.0001. BAY-405 vs vehicle *p* = 0.7175; TGFβ + BAY-405 vs TGFβ + vehicle *p* < 0.0001. BAY-405 vs vehicle *p* = 0.2195 (ANOVA). (F) T-cell reactivity assay with mouse splenocytes from wild-type and MAP4K1 kinase dead knock in mice (Supporting Information Figure S2B,C) in the presence of 300 ng/mL anti-CD3 Ab and 1 μM PGE2 as well as 500 nM BAY-405 or vehicle. WT vehicle vs KI vehicle *p* = 0.002; WT vehicle vs WT BAY-405 *p* = 0.0108; KI vehicle vs KI BAY-405 *p* = 0.4899 (Student’s *t*-test).

Upon TCR stimulation, MAP4K1 is tyrosine phosphorylated (Y-381 in human; Y-379 in mouse), triggering its recruitment to the TCR-signaling complex where it phosphorylates the adaptor protein SLP76 on Ser376 (pSLP76), thereby inducing dissociation of this complex.^4,5,7−9^ The capacity of BAY-405 to inhibit, in a target-specific manner, SLP76 phosphorylation by MAP4K1 was demonstrated in anti-CD3 antibody (Ab) stimulated Jurkat T-cells using MAP4K1-deficient cells as a control (Figure 4A, Supporting Information Figure S2A). Highly comparable data were obtained for anti-CD3 Ab stimulated primary human PBMC cultures as well as for anti-CD3/CD28-stimulated mouse splenocyte cultures(Supporting Information Figure S2B,C, Figure S3A,B, Table S6). The latter experiment provided further support for the MAP4K1-dependent action of BAY-405, in that no impact on pSLP76 levels was observed in splenocyte cultures from MAP4K1 K46M kinase-dead knock-in mice. The impact of BAY-405 on T-cell function was analyzed at the level of cytokine secretion in assays using primary human PBMC cultures activated with anti-CD3 Ab. These experiments showed that suppression of T-cell activity by PGE2 and by TGFβ could be overcome by BAY-405 (Figure 4B,C), in line with the previously documented role of MAP4K1 in the inhibitory action of these tumor-secreted factors on T-cells.^10,11^ The bell-shaped curve, as displayed by BAY-755, our initial lead compound **1**, vividly illustrates how off-target inhibition of other kinases by insufficient selective compounds can abolish the T-cell stimulatory impact of pharmacological MAP4K1 inhibition. The T-cell stimulatory impact of BAY-405 could be reproduced in PBMC cultures from multiple donors (Supporting Information Figure S3C,D), with EC_50_ levels ranging from 100 to 200 nM. Enhancement of human T-cell activation by BAY-405 was observed at a wide range of anti-CD3 Ab concentrations, albeit only in the presence of PGE2 or TGFβ, pointing at a preferential impact of our drug on suppressed T-cells in this setting (Figure 4D,E). BAY-405 was similarly able to enhance T cell reactivity in mouse splenocyte cultures stimulated with anti-CD3 Ab (Figure 4F, Supporting Information Figure S3E,F). The MAP4K1-specific mechanism of action of BAY-405 was further substantiated by the fact that enhancement of T-cell activity by the drug was not observed for MAP4K1 kinase-dead knock-in T-cells.

Even though the majority of aforementioned data are well aligned in this respect, the approximately 10-fold difference in IC_50_ with respect to SLP76 phosphorylation and EC_50_ in T-cell activation, as observed for both human and mouse T-cells (Supporting Information Table S6), cannot readily be explained. We hypothesize that the higher IC_50_ in the former assay is related to the short time window (30 min as compared to 24 h), and that therefore the EC_50_ values as observed in the T-cell activation assays bear greater relevance to the exposure levels required in vivo.

### BAY-405 Potentiates In Vitro and In Vivo Antitumor T-Cell Reactivity

**Figure 5:**
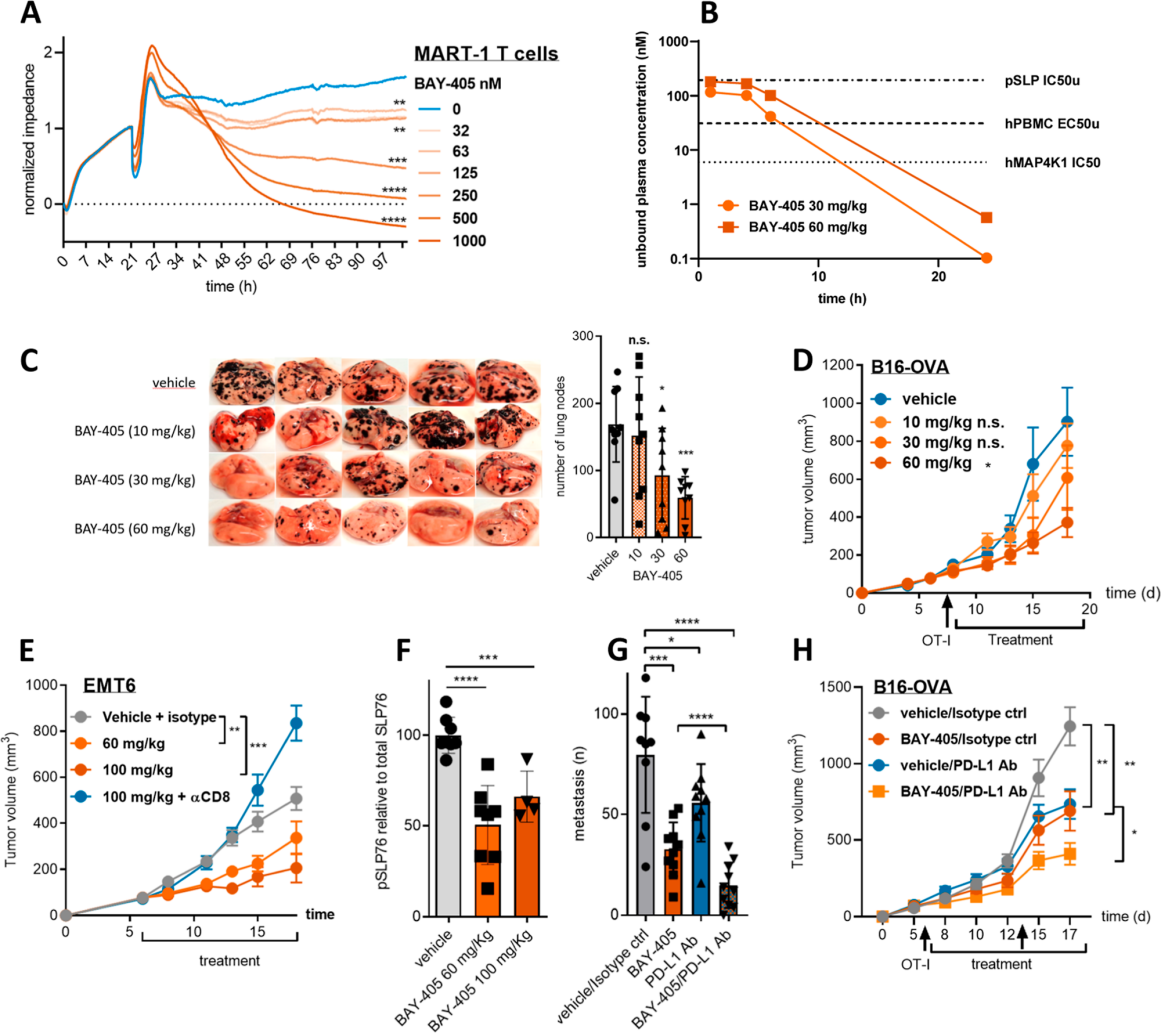
Enhancement of in vitro and in vivo antitumor T-cell reactivity. (A) xCelligence real time analysis of the cytotoxicity of MART-1-specific human T-cells toward the HLA-A*0201, MART-1 positive human melanoma cell line COLO800 in the presence of indicated concentrations of BAY-405. *P*-value represents the statistical differences at the 100 h time point (Student’s *t*-test). (B) Systemic exposure of BAY-405 (unbound plasma concentration) after a single oral dose in a mouse as indicated. Indicated in the graph are the BAY-405 IC_50_ levels determined in the relevant biochemical and cellular assays. See Supporting Information Table S9 for underlying data. (C) Impact of indicated b.i.d. doses of BAY-405 on tumor outgrowth in lungs after i.v. injection of 2 × 10^4^ B16-OVA cells. The numbers of lung nodes were analyzed at day 14 after tumor cell injection. Nine mice were used under each condition. Vehicle vs 30 mg/kg *p* = 0.0219. Vehicle vs 60 mg/kg *p* = 0.0001 (Student’s *t*-test). The experiment was repeated twice with similar outcomes (See Figure 5G and Supporting Information Figure S4B). (D) Suppression of tumor outgrowth by indicated doses of BAY-405 in mice subcutaneously challenged with 2 × 10 × 10^5^ of B16-OVA cells. Four million OT-I were injected iv at day 7. Treatment with BAY-405 started at day 8 and lasted until day 18. Nine mice were used for each condition. Vehicle vs 60 mg/kg b.i.d. *p* = 0.0393 (ANOVA). (E) Suppression of tumor outgrowth by indicated doses of BAY-405 in mice subcutaneously challenged with 5 × 10^5^ of EMT6 tumor cells. Ten mice were used for each condition. Vehicle vs 60 mg/kg b.i.d. *p* = 0.0026; vehicle vs 100 mg/kg b.i.d. *p* = 0.0002 (ANOVA). (F) Suppression pf pSLP76 levels in splenocytes of mice treated with BAY-405 at 60 mg/kg, 100 mg/kg or vehicle b.i.d., as detected by Immunoblotting using a proprietary antimouse pSer376-SLP76 antibody. From 8 mice of each treatment group, spleen samples were taken 1 h after the last compound treatment reflecting Cmax. P-value: vehicle vs BAY-405 60 mg/kg = 0.0006; vehicle vs BAY-405 100 mg/kg > 0.0001. (G) Impact of 60 mg/kg b.i.d. BAY-405, conc. PD-L1 blocking Ab (10 mg/kg twice weekly) and/or a combination thereof on B16-OVA melanoma tumor outgrowth in lungs after i.v. injection of 20,000 tumor cells. The numbers of lung tumor nodes were analyzed at day 14 after tumor grafting. Nine mice were used for each condition. Vehicle vs BAY-405 *p* = 0.0002; vehicle vs PD-L1 Ab *p* = 0.0472; vehicle vs BAY-405/PD-L1 Ab *p* < 0.0001; PD-L1 Ab vs BAY-405/PD-L1 Ab *p* < 0.0001 (Student’s *t*-test). (H) Suppression of B16 melanoma outgrowth by 60 mg/kg b.i.d. BAY-405, 10 mg/kg PD-L1 blocking Ab (twice weekly) and/or a combination thereof in mice subcutaneously challenged with 200,000 B16-OVA melanoma cells; 5 × 10^6^ splenocytes from OT-I mice were injected i.v. at day 6 and 11. Treatment with BAY-405 started at day 7 and lasted until day 17. Ten mice were used for each condition. Vehicle vs BAY-405 *p* = 0.0064; vehicle vs PD-L1 Ab *p* = 0.0045; PD-L1 Ab vs BAY-405/PD-L1 Ab *p* < 0.0146 (ANOVA). Experiment was repeated twice.

The capacity of BAY-405 to enhance T-cell-mediated tumor cell killing was first demonstrated in an in vitro real time assay. As shown in Figure 5A, the capacity of human T-cells transduced with a TCR against the human melanoma-associated antigen MART-1 to kill melanoma cells in vitro was boosted in a dose-dependent manner. Notably, the BAY-405 concentrations applied in this experiment did not significantly affect tumor cell growth in the absence of T-cells, excluding a direct cytostatic effect of our compound in this assay (Supporting Information Figure S4A). As a prelude toward in vivo testing of BAY-405, mice were exposed to single oral doses of 30 and 60 mg/kg of BAY-405, showing nearly dose-proportional increase in exposure. At both doses, BAY-405 unbound plasma concentrations exceeded levels required to activate human T-cells in our in vitro assay, while at 60 mg/kg, these approximated the pSLP76 IC_50,u_ (Figure 5B, Supporting Information Table S9). Based on our hypothesis concerning the discrepancy between the IC_50_ in pSLP76 phosphorylation assays and the EC_50_ in T-cell activation assays, we proceeded with our in vivo efficacy experiments using a dose range of 10–60 mg/kg. Accordingly, we found that BAY-405 enhanced the in vivo effector function of tumor-specific T-cells, resulting in a dose-dependent reduction in the numbers of lesions as observed in a lung colonization assay with B16 melanoma cells (Figure 5C). Importantly, the antitumor impact of BAY-405 was not observed in MAP4K1 kinase-dead knock-in mice (Supporting Information Figure S4B). Notably, PD-L1 blocking antibodies still showed a significant suppressive impact on tumor development in the MAP4K1-deficient mice, illustrating that MAP4K1 inhibition and PD-1/PD-L1 immune checkpoint blockade represent nonredundant strategies for enhancing the antitumor T-cell response. Antitumor efficacy of BAY-405 was furthermore demonstrated against pre-established, subcutaneous B16 melanoma tumors, as well as in the EMT6 tumor model (Figure 5D,E). Drug action was dependent on the T-cell immune response, as demonstrated by the lack of efficacy in CD8^+^ T-cell-depleted mice and in immunodeficient NSG mice (Supporting Information Figure S4C). This finding is in line with the fact that at relevant concentrations, our compound did not significantly affect the growth of the mouse tumor cell lines in vitro. Furthermore, repeated administration of BAY-405 at effective doses did not result in weight loss or other overt signs of toxicity (Supporting Information Figure S4D–G). BAY-405 unbound exposure levels in the EMT6 tumor model were in line with previous findings (Supporting Information Table S10, Figure S5). The intended mechanism of action of BAY-405 was further confirmed by the detection of reduced pSLP76 levels in the spleen of BAY-405-treated mice (Figure 5F), pointing at the value of this parameter as a pharmacodynamic biomarker. The notion that immune checkpoint inhibitors blocking the PD-L1/PD-1 axis are current applied as standard of care treatment for multiple cancer indications implies that MAP4K1 inhibitors may be applied either as single agent or in combination with PD-L1/PD-1 blocking antibodies. Accordingly, we found that both PD-L1 blocking Abs and BAY-405 have single agent activity in the B16 melanoma lung colonization model as well as in the corresponding subcutaneous tumor model, while showing a more profound tumor-suppressive effect when combined (Figure 5G,H).

## Conclusions

Over the course of a medicinal chemistry-driven optimization campaign, **1** was optimized to **38** (BAY-405), yielding a significant improvement in potency against human MAP4K1. Compared to **1**, which displays a selectivity score^27^ of *S* = 0.13 (80%, 1 μM) and inhibits several kinases more potently than MAP4K1, BAY-405 shows a significantly improved kinase selectivity score *S* = 0.080 (80%, 1 μM), with MAP4K1 representing the most potently inhibited kinase. Furthermore, BAY-405 features excellent drug-like properties that allow for oral application across a range of preclinical species as well as a favorable in vivo safety profile. Accordingly, application of BAY-405 in syngeneic mouse tumor models resulted in T-cell-dependent suppression of tumor outgrowth in the absence of weight loss or other overt signs of drug toxicity. Additionally, our preclinical data demonstrate that combination of pharmacological inhibition of MAP4K1 with antibody-mediated blockade of the PD-1/PD-L1 axis results in enhanced antitumor efficacy. The complementarity of these drugs is in line with the fact that they modulate distinct signals in T-cell activation; whereas MAP4K1 inhibitors modulate TCR-downstream signaling (signal 1), blockade of PD-1 signaling modulates T-cell costimulation (signal 2). Furthermore, pharmacological inhibition of MAP4K1 can overcome suppression of T-cell reactivity by TGFβ and PGE2, immunomodulators found in the TME of multiple solid tumor types. Lastly, the oral route of administration and shorter half-life of small molecule drugs provides potential for fine-tuning of the T-cell response and, thereby, the mitigation of immune-related adverse events,^1^ which is difficult to achieve with infused ICB antibodies.^31,32^ Taken together, the results of our study encourage the clinical investigation of selective MAP4K1 inhibitors, both as single agents and in conjunction with ICI antibodies.

## Experimental Section

### Chemistry

**Scheme 1:**
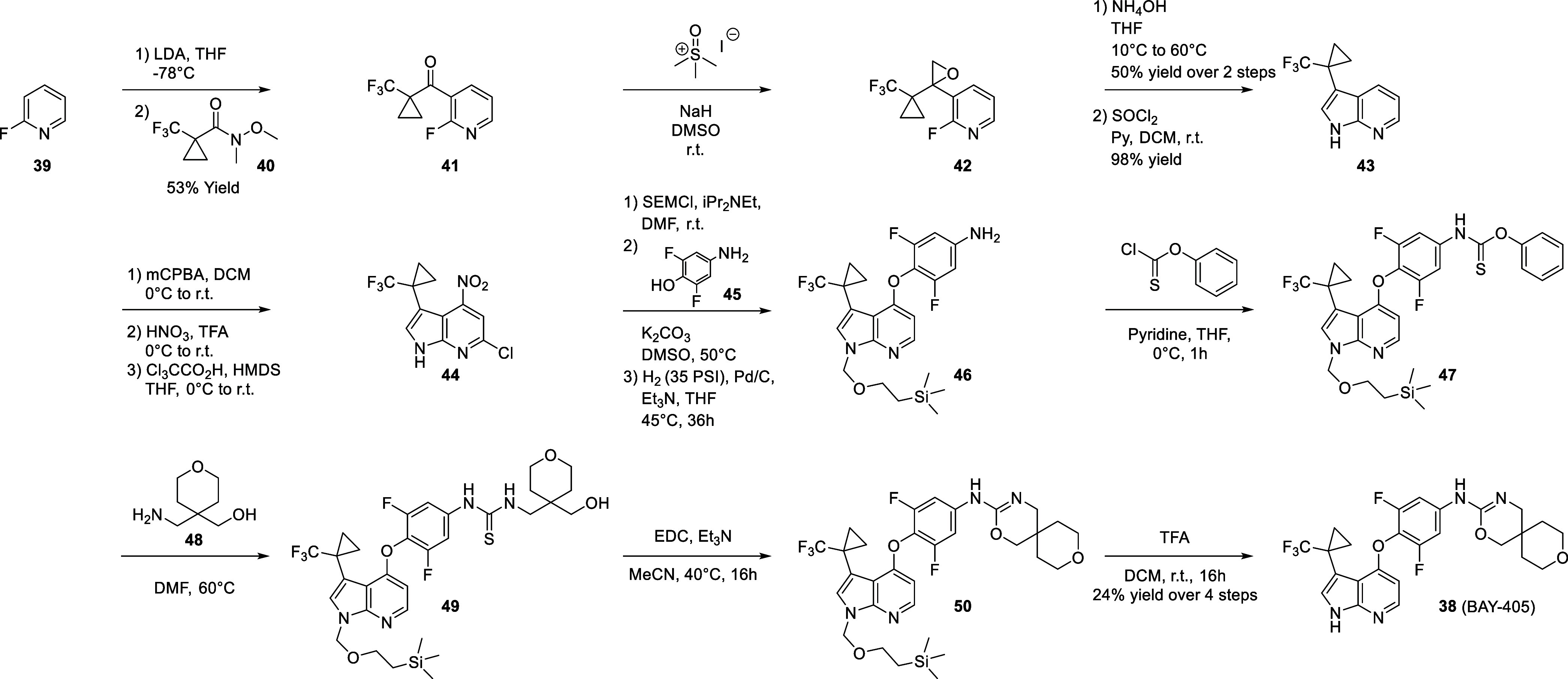
Synthesis of BAY-405 (**38**)

The synthesis of BAY-405 is outlined in Scheme 1. Commercially available 2-fluoropyridine (**39**) was metalated with a solution of LDA in THF at −78 °C and subsequently reacted with Weinreb amide **40** to afford trifluoromethyl-cyclopropyl ketone **41** in 53% yield. The ketone was converted to the epoxide **42** under Corey-Chaykovsky conditions in DMSO at room temperature.^33^ Reaction with a solution of ammonium hydroxide in THF led to epoxide ring opening followed by S_N_Ar to furnish the 5,6-bicyclic system (Supporting Information) in 50% yield over two steps.^34,35^ Dehydration with thionyl chloride under basic conditions led to 7-azaindole **43** bearing a trifluoromethyl-cyclopropyl substituent at the C3 position of the azaindole core in 98% yield. Formation of the pyridine *N*-oxide was carried out with *m*CPBA in dichloromethane, which was further nitrated at the C4 position with nitric acid in TFA. Chlorination of position C6 was then triggered by reaction with trichloroacetic acid and hexamethyl-disilizane in THF to give intermediate **44**. Final elaboration of the scaffold was carried out in a 3-step sequence including protection of the azaindole nitrogen with (trimethylsilyl)ethoxymethyl (SEM), displacement of the nitro group with difluoro-hydroxy-aniline (**45**), and hydrogenation of the remaining chlorine with palladium on charcoal in a hydrogen atmosphere under pressure to furnish intermediate **46**. The aniline was converted into thiocarbamate **47** via reaction with O-Phenyl chlorothionoformate under basic conditions. Subsequent reaction with pyran containing 1,3-aminoalcohol **48** in DMF at 60 °C afforded thio-urea **49**, which was cyclized into oxazine **50** with EDC and triethylamine in acetonitrile at 40 °C.^36^ Deprotection of the SEM with TFA in dichloromethane gave BAY-405 (**38**) in 24% yield over the final 4 steps.

As a large variety of commercially available amino-alcohols of type **48** exist, the formation of a diverse set of thio-ureas (e.g., **49**) bearing pendant hydroxy groups followed by further cyclization under dehydrating conditions represents an efficient method to assemble a range of oxazines and oxazolines (Tables 4 and 6).

**Scheme 2:**
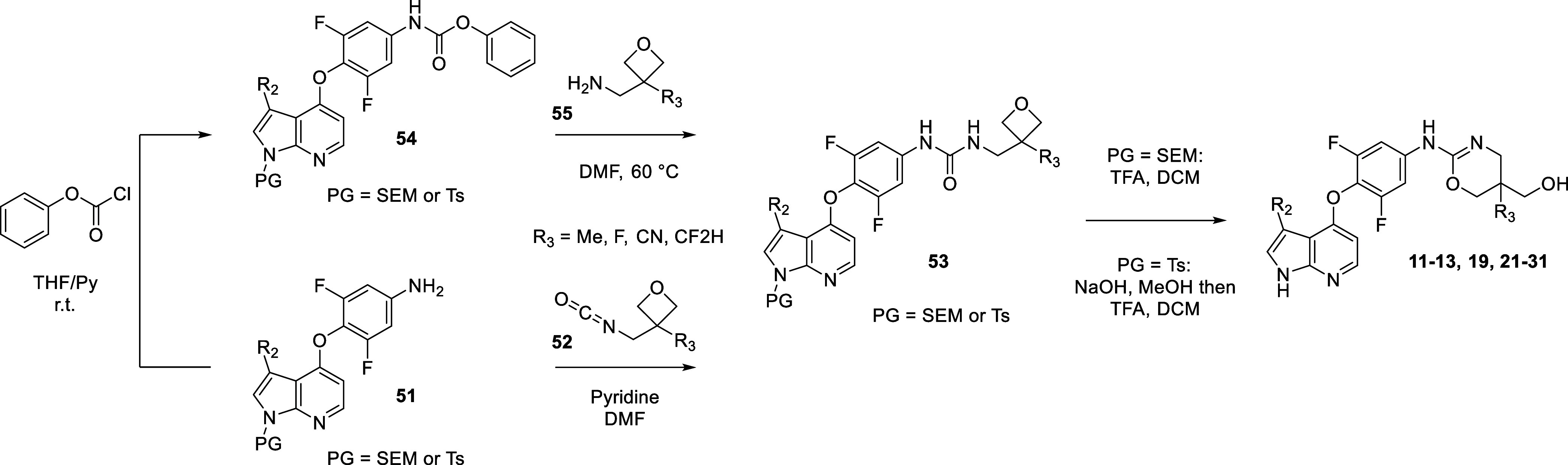
Synthesis of Oxazines via Acid-Induced Rearrangement of Oxetane Containing Ureas

For the synthesis of oxazines bearing a hydroxymethyl group (Tables 2, 4, 5, and 6), we utilized an alternative synthetic sequence, shown in Scheme 2. Azaindoles (**51**) protected with either SEM or para-methyl toluene sulfonamides (Ts) bearing an aniline in the northern phenyl group were reacted with commercially available isocyanates (**52**) containing substituted oxetanes to afford ureas bearing proximal oxetanes (**53**). When isocyanate building blocks containing exotic R_3_ groups (e.g., CN, CF_2_H) were not commercially available, an alternative two-step method was employed, which was initiated with the formation of phenyl carbamate **54**. Intermediate **54** was further reacted under thermal conditions with amino-bearing oxetanes (**55**) to afford urea intermediates of type **53**. Finally, treatment with trifluoroacetic acid triggered attack of the urea carbonyl onto the protonated oxetane resulting in rearrangement and formation of oxazines substituted with a hydroxymethyl tail (e.g., **11–13, 19, 21–31**). For SEM-protected intermediates, concomitant deprotection of the SEM group would proceed with the rearrangement reaction. For tosyl-protected azaindoles, a subsequent base-induced cleavage of the protecting was necessary. Importantly, the reaction of amines with phenyl carbamate intermediates of type **54** served as the basis for synthesizing ureas described in Tables 1 and 2.

Introduction of R_2_ groups to explore crucial SAR of the azaindole core was accomplished by a mix of reactions well described in the azaindole literature, including the route shown in Scheme 1, which was used to access compounds **30** – **38**, and compounds bearing R_2_ = CF_3_.^21,37^ Further substituents were accessed by first introducing reactive handles (e.g., Cl, Br, I) and further functionalizing with a range of reactions including Suzuki couplings (**23, 24, 26**),^38^ cyanation (**22**),^37^ photoredox C_sp_^2^–C_sp_^3^ coupling (**25**),^39^ and photochemically induced cyclopropanations (**27**, **29**).^40^ Utilizing this suite of reaction protocols, we were able to synthesize a diverse range of unique and novel azaindoles bearing sp^3^ rich substituents in the R_2_ position (see the Supporting Information for full details and methods for these transformations).

Commercially available reagents and anhydrous solvents were used as supplied without further purification. All air- and moisture-sensitive reactions were carried out under an inert atmosphere of argon. Reactions were monitored by TLC and UPLC analysis under conditions described in the Supporting Information. Flash chromatography was carried out using a Biotage Isolera One system with a 200–400 nm variable detector. Preparative HPLC was carried out with a Waters AutoPurification MS Single Quad system; column: Waters XBridge C18 5 μm, 100 × 30 mm; basic conditions: eluent A: H_2_O + 0.2 vol % aq. NH3 (32%), eluent B: MeCN; gradient: 0–0.5 min 5% B, flow: 25 mL/min; 0.51–5.50 min 10–100% B, flow: 70 mL/min; 5.51–6.5 min 100% B, flow: 70 mL/min; acidic conditions: eluent A: H_2_O + 0.1 vol % formic acid (99%), eluent B: MeCN; gradient: 0–0.5 min 5% B, flow: 25 mL/min; 0.51–5.50 min 10–100% B, flow: 70 mL/min; 5.51–6.5 min 100% B, flow: 70 mL/min; temperature: 25 °C; DAD scan: 210–400 nm.

NMR spectra were recorded at ambient temperature (22 ± 1 °C), unless otherwise noted, on Bruker AVANCE III HD spectrometers. ^1^H NMR spectra were obtained at 300, 400, 500, or 600 MHz, and referenced to the residual solvent signal (2.50 ppm for DMSO-*d*_6_). ^13^C NMR spectra were obtained at 125 MHz and also referenced to the residual solvent signal (39.52 ppm for DMSO-*d*_6_). ^1^H NMR data are reported as follows: chemical shift (δ) in ppm, multiplicity (s = singlet, d = doublet, t = triplet, q = quartet, br = broad, and m = multiplet), and integration.

Low-resolution mass spectra (electrospray ionization) were obtained via HPLC–MS (ESI) using a Waters Acquity UPLC system equipped with an SQ 3100 Mass Detector (detailed experimental methods are provided in the LC–MS methods section). Unless otherwise explicitly stated, the purity of all target compounds was at least 95%, as determined by ^1^H NMR spectroscopy and UPLC analysis. Compound names were generated by using ICS software.

### (2-Fluoropyridin-3-yl)[1-(trifluoromethyl)cyclopropyl]methanone (**41**)

To a solution of 2-fluoropyridine (1.9 g, 19.6 mmol, **39**) in THF (40 mL) was added a freshly prepared solution of LDA (1 M, 25 mL) dropwise at −78 °C under nitrogen. The mixture was stirred at −78 °C for 1 h. Then *N*-methoxy-*N*-methyl-1 (trifluoromethyl)cyclopropanecarboxamide (3.8 g, 19.3 mmol, **40**) was added. The mixture was warmed to 15 °C and stirred for 1 h. The reaction was quenched by adding a saturated aqueous solution of ammonium chloride (50 mL). The mixture was extracted with ethyl acetate (100 mL × 2). The combined organic phase was washed with brine (100 mL × 2), dried over anhydrous Na_2_SO_4_, filtered, and concentrated in vacuo. The residue was purified by chromatography over silica gel (petroleum ether: Ethyl acetate = 100:1) to give (2-fluoropyridin-3-yl)(1-(trifluoromethyl)cyclopropyl)methanone (2.4 g, 53% yield) as a yellow oil.

^1^H NMR (CDCl_3_, 400 MHz): δ = 1.65–1.54 (m, 4H), 7.31 (t, 1H), 7.87 (t, 1H), 8.38–8.37 (m, 1H).

### 2-Fluoro-3-{2-[1-(trifluoromethyl)cyclopropyl]oxiran-2-yl}pyridine (**42**)

NaH (900 mg, 22.50 mmol, 60% purity) was added to DMSO (40 mL) at 15 °C in one portion. The mixture was heated to 65 °C for 1 h. Then the mixture was cooled to 15 °C and trimethylsulfoxonium iodide (4.80 g, 21.81 mmol) was added. The mixture was stirred at 15 °C for 1 h. Then (2-fluoropyridin-3-yl)[1-(trifluoromethyl)cyclopropyl]methanone (2.4 g, 10.3 mmol, **41**) was added. The mixture was stirred at 15 °C for a further 13 h. The reaction mixture was quenched by the slow addition of water (100 mL). The suspension was extracted with ethyl acetate (100 mL × 2). The combined organic phases were washed with brine (100 mL × 2), dried over anhydrous Na_2_SO_4_, filtered, and concentrated in vacuo to give 2-fluoro-3-(2-(1-(trifluoromethyl)cyclopropyl)oxiran-2-yl)pyridine, which was used without further purification.

Method 7, LC–MS (ESI^+^): *t*_R_ = 0.82 min; *m*/*z* calcd for C_11_H_10_F_4_NO [M + H]^+^: 248.1; found, 248.1.

### 3-[1-(Trifluoromethyl)cyclopropyl]-2,3-dihydro-1*H*-pyrrolo[2,3-*b*]pyridin-3-ol (**S107**)

To a solution of 2-fluoro-3-{2-[1-(trifluoromethyl)cyclopropyl]oxiran-2-yl}pyridine (2.4 g, 9.7 mmol, **42**) in THF (12 mL) was added aq. NH_3_·H_2_O (50 mL, 364 mmol, 28% purity) at 10 °C. The mixture was stirred at 60 °C for 32 h. The mixture was poured into the water (50 mL). The suspension was extracted with ethyl acetate (50 mL × 3). The combined organic phases were washed with brine (50 mL × 2), dried over anhydrous Na_2_SO_4_, filtered, and concentrated in vacuo. The residue was purified by prep-HPLC (column: Phenomenex Gemini C18 250 × 50 mm × 10 μm; mobile phase: [water (0.05% ammonia hydroxide v/v)-ACN]; B %: 20%–45%, 26 MIN; 78% min) to get a solution, which was concentrated to 100 mL at 30 °C by rotary evaporator in vacuum. The formed solid was collected by filtration and dried in a vacuum to give the first batch of 3-(1-(trifluoromethyl)cyclopropyl)-2,3-dihydro-1H-pyrrolo[2,3-*b*]pyridin-3-ol (1.0 g, 42% yield) as a white solid. The filtrate was lyophilized to give the second batch of 3-(1-(trifluoromethyl)cyclopropyl)-2,3-dihydro-1H-pyrrolo[2,3-*b*]pyridin-3-ol (200 mg, 8% yield) as a white solid.

Method 8, LC–MS (ESI^+^): *t*_R_ = 0.67 min; *m*/*z* calcd for C_11_H_12_F_3_N_2_O [M + H]^+^: 245.1; found, 245.0.

^1^H NMR (DMSO-*d*_6_, 400 MHz): δ = 0.92–0.88 (m, 2H), 1.09–1.05 (m, 2H), 3.35 (d, 1H), 3.73 (d, 1H), 5.68 (s, 1H), 6.50 (dd, 1H), 6.53 (s, 1H), 7.40 (d, 1H), 7.85 (dd, 1H).

^19^F NMR (DMSO-*d*_6_, 400 MHz): δ = −62.

### 3-[1-(Trifluoromethyl)cyclopropyl]-1*H*-pyrrolo[2,3-*b*]pyridine (**43**)

To a mixture of 3-[1-(trifluoromethyl)cyclopropyl]-2,3-dihydro-1H-pyrrolo[2,3-*b*]pyridin-3-ol (45 g, 158 mmol, 86% purity, **S107**) and pyridine (25 mL, 310 mmol) in dichloromethane (500 mL) was added thionyl chloride (22 mL, 303 mmol) dropwise at 0 °C under a nitrogen atmosphere. The mixture was stirred at 15 °C for 12 h. The mixture was poured into ice water (500 mL) and neutralized to pH = 5–6 with 10% aqueous sodium hydroxide. The aqueous phase was extracted with dichloromethane (300 mL × 2). The combined organic phases were washed with brine (300 mL × 2), dried over sodium sulfate, filtered, and concentrated in vacuo. The residue was purified by flash column chromatography over silica gel (eluent: petroleum ether/ethyl acetate = 10:1 to 1:1) to give the title compound (35 g, 98% yield) as a yellow solid.

^1^H NMR (400 MHz, DMSO-*d*_6_): δ = 1.17–1.14 (m, 2H), 1.39–1.36 (m, 2H), 7.35–7.32 (m, 1H), 7.74 (d, 1H), 9.30 (d, 1H), 8.39–8.37 (m, 1H), 12.41 (s, 1H).

### 3-[1-(Trifluoromethyl)cyclopropyl]-1*H*-pyrrolo[2,3-*b*]pyridine 7-Oxide (**S108**)

To a solution of 3-[1-(trifluoromethyl)cyclopropyl]-1*H*-pyrrolo[2,3-*b*]pyridine (35 g, 155 mmol, intermediate **43**) in dichloromethane (350 mL) was added *m*-chloroperoxybenzoic acid (47 g, 232 mmol, 85% purity) in portions at 0 °C. The mixture was stirred at 15 °C for 12 h. The mixture was filtered, and the filtrate was washed with saturated sodium thiosulfate solution (300 mL × 2) and brine (300 mL × 2), dried over sodium sulfate, filtered, and concentrated in vacuo. The residue was suspended in methyl *tert*-butylether (50 mL) and stirred for 30 min. The suspension was filtered, and the filter cake was washed with methyl *tert*-butylether (20 mL × 2) and dried in vacuum to give the desired tile compound, which was used without further purification.

^1^H NMR (400 MHz, DMSO-*d*_6_): δ = 1.13 (m, 2H), 1.37–1.34 (m, 2H), 7.15–7.12 (m, 1H), 7.59 (s, 1H), 7.64 (d, 1H), 8.17 (d, 1H), 12.62 (s, 1H).

### 4-Nitro-3-[1-(trifluoromethyl)cyclopropyl]-1H-pyrrolo[2,3-*b*]pyridine 7-Oxide (**S109**)

To a solution of 3-[1-(trifluoromethyl)cyclopropyl]-1H-pyrrolo[2,3-*b*]pyridine 7-oxide (37 g, crude, **S108**) in trifluoroacetic acid (400 mL) was added nitric acid (30 g, 309 mmol, 65% purity) dropwise at 0 °C. The mixture was warmed to 15 °C and stirred for 14 h. Then additional nitric acid (14 g, 222 mmol, 65% purity) was added at 0 °C, and the mixture was stirred at 15 °C for another 14 h. The mixture was poured into ice water (800 mL) and stirred for 10 min. The aqueous phase was extracted with dichloromethane (300 mL × 3). The combined organic phases were washed with brine (300 mL × 2), dried over sodium sulfate, filtered, and concentrated in vacuo to give the desired title compound, which was used without further purification.

^1^H NMR (400 MHz, DMSO-*d*_6_): δ = 1.42–1.37 (m, 4H), 7.95–7.90 (m, 2H), 8.35 (d, 1H), 13.49 (s, 1H).

### 6-Chloro-4-nitro-3-[1-(trifluoromethyl)cyclopropyl]-1*H*-pyrrolo[2,3-*b*]pyridine (**44**)

To a solution of 4-nitro-3-[1-(trifluoromethyl)cyclopropyl]-1*H*-pyrrolo[2,3-*b*]pyridine 7-oxide (60 g, crude, **S109**) in THF (600 mL) was added hexamethyidisilazane (25 mL, 119 mmol) in one portion at 0 °C under a nitrogen atmosphere. Then 2,2,2-trichloroacetyl chloride (30 mL, 269 mmol) was added dropwise. The mixture was warmed to 15 °C and stirred for 12 h. The mixture was poured into ice water (1 L) and stirred for 30 min. The aqueous phase was extracted with ethyl acetate (500 mL × 2). The combined organic phases were washed with a saturated aqueous solution of sodium bicarbonate (500 mL × 2) and brine (500 mL × 2), dried over sodium sulfate, filtered, and concentrated in vacuo. The residue was purified by flash column chromatography over silica gel (eluent: petroleum ether/ethyl acetate = 100:1 to 10:1) to give the desired title compound (35 g, 57% purity) as a yellow solid.

Method 9, LC–MS (ESI^+^): *t*_R_ = 1.23 min; *m*/*z* calcd for C_11_H_8_ClF_3_N_3_O_2_ [M + H]^+^: 306.0; found, 306.0.

^1^H NMR (400 MHz, DMSO-*d*_6_): δ = 1.30–1.39 (m, 4H), 7.88 (s, 1H), 8.09 (d, 1H).

### 6-Chloro-4-nitro-3-[1-(trifluoromethyl)cyclopropyl]-1-{[2-(trimethylsilyl)ethoxy]methyl}-1*H*-pyrrolo[2,3-*b*]pyridine (**S110**)

To a solution of 6-chloro-4-nitro-3-[1-(trifluoromethyl)cyclopropyl]-1*H*-pyrrolo[2,3-*b*]pyridine (35 g, 65 mmol, 57% purity, intermediate **44**) in DMF (350 mL) was added *N*,*N*-diisopropyl-ethylamine (24 mL, 138 mmol) at 15 °C. The mixture was stirred at 15 °C for 10 min, and then 2-(trimethylsilyl)ethoxymethyl chloride (15 mL, 85 mmol) was added. The mixture was stirred at 15 °C for 20 min. The mixture was poured into ice water (1 L). The aqueous phase was extracted with ethyl acetate (500 mL × 2). The combined organic phases were washed with brine (500 mL × 2), dried over sodium sulfate, filtered, and concentrated in vacuo. The residue was purified by flash column chromatography over silica gel (eluent: petroleum ether to petroleum ether/ethyl acetate = 50:1) to give the desired title compound (25 g, 46.6% yield, 53% purity) as a yellow oil.

^1^H NMR (400 MHz, DMSO-*d*_6_): δ = 0.12 (s, 9H), 0.79–0.86 (m, 2H), 1.35–1.29 (m, 2H), 1.43 (m, 2H), 3.55 (d, 2H), 5.64 (s, 2H), 7.98 (s, 1H), 8.31 (s, 1H).

### 4-[(6-Chloro-3-[1-(trifluoromethyl)cyclopropyl]-1-{[2-(trimethylsilyl)ethoxy]methyl}-1*H*-pyrrolo[2,3-*b*]pyridin-4-yl)oxy]-3,5-difluoroaniline (**S111**)

To a mixture of 6-chloro-4-nitro-3-[1-(trifluoromethyl)cyclopropyl]-1-{[2-(trimethylsilyl)ethoxy]methyl}-1*H*-pyrrolo[2,3-*b*]pyridine (20 g, 24 mmol, 53% purity, **S110**) and 4-amino-2,6-difluoro-phenol (5.29 g, 36.5 mmol, **45**) in DMSO (200 mL) was added potassium carbonate (10.07 g, 72.86 mmol) at 15 °C under a nitrogen atmosphere. The mixture was heated to 50 °C and stirred for 2 h. After cooling to room temperature, the reaction mixture was combined with another second identical reaction mixture using 5 g of 6-chloro-4-nitro-3-(1-(trifluoromethyl)cyclopropyl)-1-((2-(trimethylsilyl)ethoxy)methyl)-1*H*-pyrrolo[2,3-*b*]pyridine (intermediate **S110**). The combined reaction mixtures were poured into ice/water (500 mL). The aqueous phase was extracted with ethyl acetate (500 mL × 3). The combined organic phases were washed with brine (500 mL × 2), dried over sodium sulfate, filtered, and concentrated by evaporator in vacuum. The residue was purified by flash column chromatography over silica gel (eluent: petroleum ether/ethyl acetate = 30:1 to 10:1) to give the desired title compound (9 g, 86% purity) as a yellow solid. Meanwhile, 6-chloro-4-nitro-3-[1-(trifluoromethyl)cyclopropyl]-1-{[2-(trimethylsilyl)ethoxy]methyl}-1*H*-pyrrolo[2,3-*b*]pyridine (5 g, 68% purity) was recovered as a yellow oil.

Method 10, LC–MS (ESI^+^): *t*_R_ = 1.00 min; *m*/*z* calcd for C_23_H_26_ClF_5_N_3_O_2_Si [M + H]^+^: 534.1; found, 534.1.

^1^H NMR (400 MHz, DMSO-*d*_6_): δ = −0.11 (s, 9H), 0.80 (t, 2H), 1.19–1.16 (m, 2H), 1.37–1.36 (m, 2H), 3.54 (t, 2H), 5.54 (s, 2H), 5.83 (s, 2H), 6.32 (s, 1H), 6.40 (d, 1H), 7.79 (s, 1H).

### 3,5-Difluoro-4-[(3-[1-(trifluoromethyl)cyclopropyl]-1-{[2-(trimethylsilyl)ethoxy]methyl}-1*H*-pyrrolo[2,3-*b*]pyridin-4-yl)oxy]aniline (**46**)

To 4-[(6-chloro-3-[1-(trifluoromethyl)cyclopropyl]-1-{[2-(trimethylsilyl)ethoxy]methyl}-1*H*-pyrrolo[2,3-*b*]pyridin-4-yl)oxy]-3,5-difluoroaniline (9 g, 86% purity and 3 g crude, **S111**) in THF (200 mL) were added palladium on charcoal (2 g, 10% purity, containing 50% water) and triethylamine (10 mL, 71.9 mmol) under a nitrogen atmosphere. The suspension was degassed under a vacuum and purged with hydrogen several times. The mixture was stirred under hydrogen (15 psi) at 45 °C for 36 h. The mixture was filtered through a pad of Celite and the filtrate was concentrated in vacuum. The residue was dissolved in THF (200 mL) and palladium on charcoal (2 g, 10% purity, containing 50% water) was added. The mixture was stirred under hydrogen (15 psi) at 45 °C for 60 h. The mixture was filtered through a pad of Celite, and the cake was washed with ethanol (100 mL × 2). The filtrate was concentrated in vacuo. The residue was purified by flash column chromatography over silica gel (eluent: 0–10% of ethyl acetate in petroleum ether) to give the desired title compound (9 g, containing solvent residue) as a brown oil. This product was combined with a second batch of product (3 g, containing solvent residue) by dissolving in acetonitrile (200 mL). Water (100 mL) was added. The solution was concentrated by evaporation in vacuum to ∼150 mL. The residue was lyophilized to give the desired title compound (10.2 g) as a white solid.

Method 5, LC–MS (ESI^+^): *t*_R_ = 1.03 min; *m*/*z* calcd for C_23_H_27_F_5_N_3_O_2_Si [M + H]^+^: 500.2; found, 500.1.

^1^H NMR (400 MHz, DMSO-*d*_6_): δ = −0.11 (s, 9H), 0.82–0.78 (m, 2H), 1.17 (m, 2H), 1.38–1.35 (m, 2H), 3.54 (t, 2H), 5.59 (m, 2H), 5.76 (s, 2H), 6.42–6.34 (m, 3H), 7.73 (s, 1H), 8.11 (d, 1H).

^19^F NMR (400 MHz, DMSO-*d*_6_): δ = −69, −129.

### O-Phenyl {3,5-Difluoro-4-[(3-[1-(trifluoromethyl)cyclopropyl]-1-{[2-(trimethylsilyl)ethoxy]methyl}-1*H*-pyrrolo[2,3-*b*]pyridin-4-yl)oxy]phenyl}carbamothioate (**47**)

To a stirred solution of 3,5-difluoro-4-[(3-[1-(trifluoromethyl)cyclopropyl]-1-{[2-(trimethylsilyl)ethoxy]methyl}-1*H*-pyrrolo[2,3-*b*]pyridin-4-yl)oxy]aniline (500 mg, 1.00 mmol, intermediate **46**) in a mixture of pyridine (750 μL, 9.3 mmol) and THF (7.5 mL) was added O-phenyl carbonochloridothioate (150 μL, 1.1 mmol, CAS no. [1005-56-7]). The reaction mixture was stirred at 0 °C for 1 h, at which time the solvent was evaporated to afford the crude material, which was used in the next step without further purification.

Method 2, UPLC-MS (ESI-): *t*_R_ = 1.73 min; *m*/*z* calcd for C_30_H_29_F_5_N_3_O_3_SSi [M - H]^−^: 634.2; found: 634.5.

### *N*-{3,5-difluoro-4-[(3-[1-(trifluoromethyl)cyclopropyl]-1-{[2-(trimethylsilyl)ethoxy]methyl}-1*H*-pyrrolo[2,3-*b*]pyridin-4-yl)oxy]phenyl}-*N*′-{[4-(hydroxymethyl)oxan-4-yl]methyl}thiourea (**49**)

To a stirred solution of O-phenyl {3,5-difluoro-4-[(3-[1-(trifluoromethyl)cyclopropyl]-1-{[2-(trimethylsilyl)ethoxy]methyl}-1*H*-pyrrolo[2,3-*b*]pyridin-4-yl)oxy]phenyl}carbamothioate (190 mg, 0.30 mmol, intermediate **47**) in DMF (4.0 mL) was added [4-(aminomethyl)oxan-4-yl]methanol (87 mg, 0.60 mmol, CAS no. [959238-22-3]). The resulting mixture was heated to 60 °C for 2 h, at which time water and ethyl acetate were added and the layers were separated. The aqueous phase was extracted twice with ethyl acetate, and the combined organic layers were washed with brine, dried over sodium sulfate, filtered, and evaporated to give the crude product, which was used without further purification.

Method 1, UPLC-MS (ESI^+^): *t*_R_ = 1.50 min; *m*/*z* calcd for C_31_H_39_F_5_N_4_O_4_SSi [M + H]^+^: 687.2; found, 688.

### *N*-{3,5-difluoro-4-[(3-[1-(trifluoromethyl)cyclopropyl]-1-{[2-(trimethylsilyl)ethoxy]methyl}-1*H*-pyrrolo[2,3-*b*]pyridin-4-yl)oxy]phenyl}-2,9-dioxa-4-azaspiro[5.5]undec-3-en-3-amine (**50**)

To a stirred solution of *N*-{3,5-difluoro-4-[(3-[1-(trifluoromethyl)cyclopropyl]-1-{[2-(trimethylsilyl)ethoxy]methyl}-1*H*-pyrrolo[2,3-*b*]pyridin-4-yl)oxy]phenyl}-*N*′-{[4-(hydroxymethyl)oxan-4-yl]methyl}thiourea (200 mg, 0.30 mmol, intermediate **49**) in acetonitrile (4.0 mL) were added 1-(3-(Dimethylamino)propyl)-3-ethylcarbodiimide hydrochloride (112 mg, 582 μmol) and triethylamine (122 μL, 726 μmol). The resulting mixture was stirred at 40 °C overnight, at which time water and ethyl acetate were added and the layers were separated. The aqueous phase was extracted twice with ethyl acetate, and the combined organic layers were washed with brine, dried over sodium sulfate, filtered, and evaporated to afford the crude product which was used without further purification.

Method 1, UPLC-MS (ESI^+^): *t*_R_ = 1.59 min; *m*/*z* calcd for C_31_H_37_F_5_N_4_O_4_Si [M + H]^+^: 653.3; found, 654.

### *N*-[3,5-difluoro-4-({3-[1-(trifluoromethyl)cyclopropyl]-1*H*-pyrrolo[2,3-*b*]pyridin-4-yl}oxy)phenyl]-2,9-dioxa-4-azaspiro[5.5]undec-3-en-3-amine. BAY-405 (**38**)

To a solution of *N*-{3,5-difluoro-4-[(3-[1-(trifluoromethyl)cyclopropyl]-1-{[2-(trimethylsilyl)ethoxy]methyl}-1*H*-pyrrolo[2,3-*b*]pyridin-4-yl)oxy]phenyl}-2,9-dioxa-4-azaspiro[5.5]undec-3-en-3-amine (190 mg, 291 μmol, intermediate **50**) in dichloromethane (2.0 mL) was added trifluoroacetic acid (1.0 mL). The reaction mixture was stirred at room temperature overnight, at which time the mixture was basified to pH > 10 with 2 M sodium hydroxide and ethyl acetate was added. The layers were separated, and the aqueous layer was extracted three times with ethyl acetate. The combined organic layers were washed with brine, dried over sodium sulfate, filtered, and evaporated. The crude material was dissolved in acetonitrile (10 mL) and treated with a 25% aqueous solution of ammonia (5 mL). The resulting solution was stirred for 1 h and then purified by preparative HPLC to afford the title compound **38** (BAY-405) (38 mg, 24% yield over 4 steps).

Method 1, UPLC-MS (ESI^+^): *t*_R_ = 1.16 min; *m*/*z* calcd for C_25_H_23_F_5_N_4_O_3_ [M + H]^+^: 523.2; found, 523.5.

^1^H NMR (400 MHz, DMSO-*d*_6_): δ ppm 1.18 (br s, 2H), 1.31–1.35 (m, 2H), 1.44 (br s, 4H), 3.28 (br d, 2H), 3.54–3.67 (m, 4H), 4.07 (br s, 2H), 6.26 (d, 1H), 7.53 (s, 1H), 7.57 (br s, 1H), 8.04 (d, 1H), 9.03 (br s, 1H), 11.91 (s, 1H).

^13^C NMR (DMSO-*d*_6_, 126 MHz): δ 157.7, 155.6, 155.5, 153.6, 153.6, 150.4, 144.2, 127.4, 126.8, 125.2, 121.7, 108.6, 107.7, 98.8, 78.8, 78.7, 78.5, 78.3, 71.8, 62.2, 40.4, 40.1, 39.9, 39.8, 39.6, 39.4, 31.1, 28.1, 19.9, 19.7, 10.6.

### Cloning, Expression, and Purification of Recombinant Full-Length Human MAP4K1 and Orthologues for Screening

Synthetic cDNA encoding full-length human, rat, mouse, and cynomolgus monkey MAP4K1 (uniprot accession codes: Q92918, D3Z8I4, P70218 and G8F5R9 with variations) was codon-optimized by GeneArt (Regensburg, Germany) for expression in insect cells and subcloned into pDONR221. Expression plasmids were generated via the LR recombination reaction between the entry clone and a required destination vector introducing a GST-tag and a TEV protease cleavage site at the *N*-terminus (Invitrogen). The flashBAC Gold (Oxford Expression Technologies, UK) expression system was used to generate baculoviruses for infection in High Five cells (monkey and rat) or Sf9 cells (human and mouse) using a multiplicity of infection of 1. Infected insect cells were harvested 48 h post infection by centrifugation and stored at −80 °C until lysis.

The harvested cells were resuspended in lysis buffer containing 50 mM Tris–HCl (pH 7.5) 150 mM NaCl 10% glycerol, 0.1 mM EDTA, 1 mM DTT, 0.1% NP40, and protease inhibitor cocktail (complete EDTA free, Roche) and incubated on ice for 30 min. The lysate was clarified by centrifugation at 25,000*g* for 45 min at 4 °C and incubated with glutathione beads (Glutathione sepharose 4B, GE Healthcare) overnight at 4 °C using a rotator. The beads were washed with 50 mM Tris–HCl (pH 7.5) 150 mM NaCl 10% glycerol, 0.1 mM EDTA, 1 mM DTT, resuspended, and transferred into a Kronlab glass column connected to an Äkta pure (GE Healthcare) operated at 6 °C. Proteins were eluted by using 50 mM Tris–HCl (pH 7.5) 150 mM NaCl 10% glycerol, 0.1 mM EDTA, 1 mM DTT and 25 mM glutathione. No further purification steps were performed.

### Cloning, Expression, and Purification of Recombinant Human MAP4K1 Kinase Domain

For crystallization, the synthetic DNA for the human kinase domain of MAP4K1 (comprising the amino acids 1-307) containing the mutation sites T165E and S171E was prepared by GeneArt (Regensburg, Germany). Cloning of the kinase domain constructs and baculovirus generation have been done as described for the full-length proteins, with the exception that the kinase domain contained a N-terminal FLAG tag with a TEV cleavage site. The cell pellet from 16L Sf9-ESF cells was lysed in 50 mM Tris (pH 8.0), 150 mM NaCl, protease inhibitor cocktail (complete, Roche), 0.1% NP-40, and 1 mM TCEP and incubated for 60 min at 4 °C. The lysate was clarified by centrifugation at 25,000*g* for 60 min at 4 °C and the supernatant was loaded onto a column containing 15 mM anti-FLAG agarose (Pierce), which were equilibrated with wash buffer (50 mM Tris (pH 8.0), 150 mM NaCl, 1 mM TCEP). Bound kinase domain was washed with wash buffer and eluted with 50 mM Tris (pH 8.0), 150 mM NaCl, 250 μg/mL FLAG peptide, and 1 mM TCEP. The affinity purified protein was treated with TEV protease at a ratio of 1:10 for 6 h at 10 °C in order to remove the FLAG tag. The tag-free protein was diluted in 20 mM Tris (pH8.0), 10% glycerol, and 0.5 mM TCEP and further purified by strong anion exchange (SAX) chromatography using a MonoQ GL 10/10 column (Sigma-Aldrich). The elution was performed by increasing the concentration of NaCl in 20 mM Tris (pH 8.0), 10% glycerol, and 0.5 mM TCEP. Two main peaks were observed in SAX, which were pooled and purified by size exclusion chromatography (SEC) separately. SEC was performed on a Superdex S75 26/60 column (GE Healthcare) and 50 mM Tris (pH8.0), 150 mM NaCl, 10% glycerol, and 0.5 mM TCEP as running buffer. The SAX peak 1, which contained mainly monomeric MAP4K1, was concentrated up to 8 mg/mL and used for crystallization, whereas SAX peak 2 contained mainly dimeric protein.

For biophysical purposes, a construct of the human wildtype kinase domain of MAP4K1 (comprising the amino acids 1–346) with an *N*-terminal GST tag was cloned, and the baculovirus was generated as described for the structural biology construct. The cell pellet from 16L Sf9 cells was lysed in 50 mM Tris (pH 7.5), 150 mM NaCl, protease inhibitor cocktail (complete, Roche), 0.2% NP-40, 1 mM DTT and incubated for 60 min at 4 °C. The lysate was clarified by centrifugation at 25,000*g* for 60 min at 4 °C and affinity purified by incubating with glutathione beads (Glutathione agarose 4B, GE Healthcare) for 3 h at 15 °C in a spinner flask. The beads were transferred into an empty glass column (Pierce) connected to an Äkta pure (GE Healthcare) and washed with 50 mM Tris–HCl (pH 7.5), 150 mM NaCl, 10% glycerol, 0.1 mM EDTA, and 1 mM DTT. The column was washed with 3 column volumes (CV) of wash buffer and eluted with 2 CVs elution buffer (wash buffer with 25 mM glutathione). The protein was further purified by SEC using a Superdex S200 26/60.

### MAP4K1 Kinase Activity Inhibition Assays (Human, Mouse)

The ability of the compounds to inhibit the kinase activity of MAP4K1 was quantified employing TR-FRET-based binding competition assays as described in the following paragraphs.

Recombinant fusion proteins of N-terminal GST and full-length MAP4K1 (as described above) were used as enzymes and the biotinylated artificial peptide biotin-Ahx-IKKRKLTRRKSLKG (C-terminus in amide form) as substrate.

For the assay, 50 nL of a 100-fold concentrated solution of the test compound in DMSO was pipetted into a black 1536-well microtiter plate (Greiner Bio-One, Germany), and 2 μL of solution of enzyme in aqueous assay buffer [25 mM Hepes pH 7.5, 5 mM MgCl_2_, 1 mM β-glycerolphosphate, 1 mM dithiothreitol, 1 mM ethylene glycol-bis(2-aminoethyl ether)-*N*,*N*,*N*′,*N*′-tetraacetic acid [EGTA], 0.001% (w/v) bovine serum albumin [BSA], 0.001% (w/v) Pluronic F-127 (Sigma)] was added and incubated for 15 min at 22 °C. Then the enzyme reaction was started by the addition of 3 μI of a solution of the peptide substrate (67 nM = >final concentration in the 5 μI assay volume is 40 nM) and ATP (16.7 μM or 1.67 mM = > final concentration in the 5 μI assay volume is 10 μM or 1 mM) in assay buffer. The resulting mixture was incubated for a reaction time of 60 min (20 min for the assay with 1 mM ATP) at 22 °C. The concentration of the enzyme was adjusted depending on the activity of the enzyme lot and chosen appropriate to have the assay in the linear range; typical concentrations were 77 pM for the human and 63 pM for the mouse MAP4K1. The reaction was stopped by addition of a solution of TR-FRET detection reagents (13.3 nM streptavidine-XL665 [Cisbio Bioassays, France] and 0.83 nM antiribosomal protein S6 (pSer236)-antibody from Invitrogen [# 44921G] and 1.33 nM LANCE EU-W1024 labeled Protein G [PerkinElmer, product no. AD0071]) in an aqueous EDTA solution (66.7 mM EDTA, 0.1% (w/v) BSA in 133 mM Hepes pH 7.5).

The resulting mixture was incubated for 60 min at 22 °C to allow the formation of a complex between the phosphorylated biotinylated peptide and the detection reagents. Subsequently, the amount of phosphorylated substrate was evaluated by measurement of the resonance energy transfer from the Eu-chelate to the streptavidin-XL665. Therefore, the fluorescence emissions at 620 and 665 nm after excitation at 350 nm were measured in a TR-FRET reader (Pherastar FS, BMG Labtechnologies, Germany). The ratio of the emissions at 665 and at 622 nm was taken as the measure of the amount of the complex. The data were normalized (assay reaction without inhibitor = 0% inhibition, all other assay components but GST-fusion protein = 100% inhibition). The test compounds were tested on the same microtiter plate in 11 different concentrations in the range of 20 mM to 0.07 nM (20 pM, 5.7 pM, 1.6 pM, 0.47 pM, 0.13 pM, 38 nM, 1 1 nM, 3.1 nM, 0.9 nM, 0.25 nM, and 0.07 nM; the dilution series prepared separately before the assay on the level of the 100-fold concentrated solutions in DMSO by serial dilutions) in duplicate values for each concentration and IC_50_ values were calculated using Genedata Screener software.

### MAP4K1 Tracer Binding Competition Assays (Human, Mouse, Rat, Monkey)

The ability of the compounds to inhibit the binding of an Alexa647-labeled ATP-competitive kinase inhibitor to Glutathione-*S*-transferase-(GST-) fusion protein was quantified employing the TR-FRET-based binding competition assays as described in the following paragraphs.

Recombinant fusion protein of N-terminal GST and full-length MAP4K1 (as described above) were used as the GST-fusion protein. Tracer 222 from Invitrogen (catalogue no. PR9198A) was used as Alexa647-labeled ATP-competitive kinase inhibitor.

For the assay, 50 nL of a 100-fold concentrated solution of the test compound in DMSO was pipetted into a black 1536-well microtiter plate (Greiner Bio-One, Germany), and a 3 μL solution of Tracer 222 (25 nM = >final concentration in 5 μI assay volume is 15 nM) in aqueous assay buffer [25 mM Tris/HCl pH 7.5, 10 mM MgCl_2_, 5 mM β-glycerolphosphate, 2.5 mM dithiothreitol, 0.5 mM ethylene glycol-bis(2-aminoethyl ether)-*N*,*N*,*N*′,*N*′-tetraacetic acid [EGTA], 0.5 mM sodium ortho-vanadate, 0.01% (w/v) BSA, 0.005% (w/v) Pluronic F-127 (Sigma)] was added. The binding competition was started by the addition of 2 μI of a solution of the GST-fusion protein (2.5 nM = > final conc. in the 5 μI assay volume is 1 nM for human, rat, and monkey proteins, for mouse protein twice the conc. was used) and of Anti-GST-Tb (1.25 nM = >final conc. in the 5 μI assay volume is 0.5 nM), a Lumi4-Tb Cryptate-conjugated anti-GST-antibody (Cisbio Bioassays, France), in assay buffer.

The resulting mixture was incubated for 30 min at 22 °C to allow the formation of a complex between Tracer 222, the fusion protein, and Anti-GST-Tb. Subsequently, the amount of this complex was evaluated by measurement of the resonance energy transfer from Tb-cryptate to Tracer 222. Therefore, the fluorescence emissions at 620 and 665 nm after excitation at 350 nm were measured in a TR-FRET reader (Pherastar FS, BMG Labtechnologies, Germany). The ratio of the emissions at 665 and 622 nm was taken as the measure of the amount of complex. The data were normalized (assay reaction without inhibitor = 0% inhibition, all other assay components but GST-fusion protein = 100% inhibition). The test compounds were tested on the same microtiter plate in 11 different concentrations in the range of 20 mM to 0.07 nM (20 pM, 5.7 pM, 1.6 pM, 0.47 pM, 0.13 pM, 38 nM, 1 1 nM, 3.1 nM, 0.9 nM, 0.25 nM and 0.07 nM); the dilution series prepared separately before the assay on the level of the 100-fold concentrated solutions in DMSO by serial dilutions in duplicate values for each concentration and IC_50_ values were calculated using Genedata Screener software.

### Biochemical Kinase Panel

For biochemical kinase selectivity testing, the KinaseProfiler (Eurofins Discovery, France) was performed, which comprises a radiometric assay platform directly measuring kinase catalytic activity inhibition.

### Cellular Kinase Selectivity Testing

For cell-lysate-based kinase selectivity testing of BAY-405, the KinomeScout profiling technology was utilized (OmicScouts, Germany). This technique comprises chemical proteomics affinity and selectivity profiling in cell lysates. The assays were performed as previously described.^30^

The cell lysate mixture used to profile BAY-405 was generated from Jurkat E6-1 [American Type Culture Collection (ATCC), TIB-152], COLO 205 (ATCC, CCL-222) and MV-4–11 (ATCC, CRL-9591) cells grown in RPMI 1640 medium (Biochrom GmbH), and SK-N-BE-(2) (ATCC, CRL-2271) cultured in DMEM/HAM’s F-12 medium (Biochrom GmbH). All were supplemented with 10% FBS (Biochrom GmbH) and 1% antibiotic solution (Sigma). At the lysate level, a 1:1:1:1 mixture of all 4 cell lines was generated.

When KinomeScout profiling was performed using the described cell lysate mix, 422 protein kinases (covering almost 80% of the human kinome) were identified and quantified utilizing two different complementary broad band kinase inhibitor matrices. The compound concentrations applied for competition experiment to assess dose–response curves were DMSO control, 0.001, 0.003, 0.01, 0.03, 0.1, 0.3, 1, 3, and 30 μM.

Peptide and protein identification and quantification, and data analyses were performed as previously described.^30^

### Biacore SPR Analysis

For SPR measurements, recombinant MAP4K1 protein (GST-tagged, amino acids X–Y tag as described above) was immobilized using standard amine coupling methods. Briefly, carboxymethylated dextran biosensor chips (Series S Sensor Chip CM5, GE Healthcare) were activated with 1 -ethyl-3-(3-(dimethylamino)propyl)-carbodiimide hydrochloride (EDC) and *N*-hydroxysuccinimide (NHS) according to the supplier’s instructions. The protein (stabilized by addition of 1 μM of a high-affinity ligand) was diluted in 20 mM MES pH 7.0 and injected on the activated chip surface. Subsequently, a solution of 1 M ethanolamine-HCI (GE Healthcare) was injected into the cytosol to block unreacted groups. Typically, immobilization levels of approximately 1500–5000 response units were reached. A reference surface was generated by treatment with NHS-EDC and ethanolamine-HCI. Compounds were dissolved in 100% dimethyl sulfoxide (DMSO) to a concentration of 10 mM and subsequently diluted in running buffer (generated from HBS-EP + Buffer 10× (GE Healthcare) and 1% v/v DMSO). For SPR binding measurements, serial dilutions of the compound (five dilution steps, ranging from 4 nM up to 1 μM) were injected over the immobilized protein. Compound binding was measured in a single cycle mode at 25 °C with a flow rate of 100 μL/min in running buffer. Compounds were injected for 60 s, followed by a final dissociation time of up to 500 s. Measurements were performed on a Biacore T200 instrument (GE Healthcare) The double-referenced sensorgrams were fit to a simple reversible 1:1 reaction mechanism as implemented in the Biacore Insight Evaluation software.

### Crystallization

The purified protein was concentrated to 8 mg/mL in a buffer of 50 mM TRIS at pH 8.0, 150 mM sodium chloride, 10% Glycerol, and 0.5 mM TCEP. BAY-405 was dissolved in DMSO to a concentration of 200 mM and was added to the protein solution to give a final concentration of 5 mM (∼20-fold excess). Prior to crystallization, the protein:BAY 405 mixture was incubated for ∼2 h on ice. Crystallization was performed using vapor diffusion in sitting drops with equal volumes of protein solution and reservoir solution (0.1 M TRIS buffer (pH, 8.0) and 20% MPD). Crystal plates with a maximum size of ∼0.2 to 0.3 mm grew within a few days at RT.

### Data Collection, Processing, and Refinement

For data collection, 20% glycerol was added to the drop, and crystals were shock frozen in liquid nitrogen. A diffraction data set to 2 Å resolution was collected on beamline BL14.1 operated by the Helmholtz-Zentrum Berlin at the BESSY II electron storage ring^41^ and processed using XDS^42^ via the graphical user interface XDSAPP.^43^ The structure was solved by rigid body refinement with REFMAC5^44^ with an internal crystal structure as the starting model. The initial model was rebuilt in COOT^45^ and refined using REFMAC5. The data collection and refinement statistics are summarized in Supporting Information Table S8. The crystallographic data for the cocrystal structure of BAY-405 with human MAP4K1 has been deposited with the RCSB PDB with accession code 8PAR.

Additional cocrystal structures of structurally related derivatives, with MAP4K1 as target but also with MST1 as surrogate kinase, have been deposited with accession codes 8PAS, 8PAU, 8PAV and 8PAW.

### Computational Studies

Modeling studies were performed using the maestro suite by Schrodinger in versions 2017–2 to 2018–1. For docking experiments, Glide^46^ was used in versions 75013 to 78011, and protein structures were prepared using the protein preparation wizard with default parameters, with ligand protonation states being manually adapted as required. The preparation of 3D ligand structures for docking employed an in-house Pipeline Pilot script BIOVIA Pipeline Pilot; Dassault Systèmes; San Diego; https://www.3ds.com/products/biovia/pipeline-pilot that utilizes Corina^47^ for 3D structure generation and SimPlus ADMET predictor https://www.simulations-plus.com/software/admetpredictor/ for the assignment of protonation states.

### Ethics

Blood from healthy donors was obtained from the blood bank Mannheim as approved by the Research Ethics Committee (Votum 2015–630N-MA, January 22, 2016, Study title: Gewinnung peripherer mononulearere Zellen aus Leukaphereseprodukten für die ex vivo Expansion and Reaktivierung von tumorinfiltrierenden Lymphozyten sowie von Tumorantigen- and Virusspezifischen Zellen).

C57BL/6N mice and C57BL/6N Ly5.1 mice were bred at the central animal facility of the German Cancer Research Center. Balb/c, C57/Bl6N, and OT-I Rag2–/– mice (deficient in recombination-activating gene 2 mice) were purchased from external providers (Charles River, Germany, Envigo, The Netherlands or TACONICS, USA). Mice were kept in the central animal facility at Bayer AG Berlin or DKFZ institute, Heidelberg, Germany under strict hygienic, specific pathogen–free, and health monitoring conditions. 8- to 12 week-old mice were used for experiments. Experiments were conducted according to governmental and institutional guidelines and regulations (Regierungspräsidium Karlsruhe, permit no. A12/18 and A12/15) (LAGeSo Berlin A0089/17 and A0333/17).

### Generation of the MAP4K1 KI Mouse: C57BL/6NTac Map4k1 K46M Kinase-Dead Mouse

The MAP4K1 KI (knock-in) mouse was generated by a constitutive K46M point mutation introduction in the Map4k1 gene via CRISPR/Cas9-mediated gene editing (custom-made by Taconic Biosciences GmbH, Germany; see Supporting Information Figure S2B–C). Sequence of the sgRNA is depicted in extended data in Figure 2B. Validation of the constitutive knock-in was performed using target site PCR amplification and DNA sequence analysis of isolated genomic tail DNA.

### OT-1 Mice

RAG2 OT-I mice were obtained from TACONIC Biosciences. The RAG2 OT-I cell line carries a transgene that encodes a TCR (Valpha2-Jalpha26 and Vbeta5-Dbeta2-Jbeta.6) specific for a chicken ovalbumin peptide fragment (257–264) presented by the MHC class I molecule H2-Kb. Deficiency in the recombination activating gene 2 impairs the expression of endogenous T cell receptors, and therefore this line does not develop mature T or B.

### Specification Human and Mouse Tumor Cell Lines

HCC2935 (CRL-2869), B16F10 (CRL-6475), and EMT6 (CRL-2755) cell lines were obtained from the ATCC. COLO 800 (AC193) was obtained from the DSMZ. B16F10 was stably transduced with an EGF-OVA construct to generate the B16-OVA cell line used in this study (custom-made by NMI, Germany). C38 was purchased from the NCI Frederick.

### Jurkat MAP4K1 KO Cell Line

MAP4K1-deficient Jurkat T-REX IL2 NLUCP cells (PROMEGA) were generated stably expressing Cas9 using the LVCAS9NEO-1EA EF1α-CAS9–2A-Neomycin lentiviral particles from Sigma and pRSG20-U6-sgMAP4K1-CMV-TagRFP-2A-Puro (Cellecta, target sequence: TTCCGTTCTCCATCAGACGA) in Jurkat cells expressing the IL2 nanoluciferase reporter construct. The Jurkat T-REX IL2 NLUCP MAP4K1 KO clone 3 (Jurkat MAP4K1 KO cells) was identified completely lacking MAP4K1 protein expression as shown by Western Blot analysis (rabbit anti MAP4K1, Abcam, ab33910; Supporting Information Figure S2A). To monitor potential effects of MAP4K1 knockout on immune response, IL2-NLuc activity was monitored. IL2 Nano-Luc Jurkat cells expressing or lacking MAP4K1 were treated with vehicle, 20 μg/mL IgG and 5 μg/mL anti-CD3 or 20 μg/mL IgG, 5 μg/mL anti-CD3, and 5 μg/mL anti-CD28 (Goat antimouse IgG (H + L) (Thermo Fisher, #31160), Mouse antihuman CD3 (BD Pharmingen, #555329), Mouse antihuman CD28 (BD Pharmingen, #555725)) for 24 h and Nano-Glo Luciferase Assay (Promega) was performed according to the manufacturer’s instructions.

### PBMC Source and Preparation

Human PBMC were purified from buffy coats (Blood Transfusion Centre, Mannheim, Germany) using Ficoll-Hypaque gradient centrifugation. PBMC were harvested from the interface, washed five times with RPMI 1640, and finally the cells were suspended in freezing medium (ibidi) and stored in liquid nitrogen at a concentration of 50 × 10^6^ cells/mL.

### Mouse Splenocyte Isolation

Spleens and peripheral lymph nodes were collected from C57BL/6N or OT-I mice and were dissociated to obtain single-cell suspensions. Red blood cells were lysed with an ACK buffer. For in vitro activation, cells from C57BL/6N or OT-I were suspended at a density of 4 × 10^6^ cells per mL in RPMI-1640 medium supplemented with 10% FCS, 2 nM glutamine. For in vivo adoptive therapy, OT-I cells were suspended in PBS at a density of 50 × 10^6^ cells per mL.

### In Vitro Cytotoxicity Assay with B16F10 and EMT-6 Tumor Cells

To assess a direct antiproliferative or cytotoxic effect of the compounds on in vitro tumor cell growth, CellTiter-Glo Luminescent Cell Viability assays were performed according to the manufacturer’s instruction (Promega #G7573). B16F10 cells were cultured in DMEM/Mas F12 supplemented with 10% FCS medium (Millipore; #S0615), EMT-6 cells in Waymouth’s MB752/1 (Gibco #31220-023) supplemented with 15% FCS, C38 cells in Ham’s F12 (Gibco #FG0815) supplemented with 10% FCS medium, Jurkat E6–1 cells in RPMI 1640 with GlutaMAX (GIBCO; #61870–010) supplemented with 10% FCS for 72h in the presence of the indicated compound concentration. Read-out of luminescence was done with a Plate Reader Victor X2 (PerkinElmer).

### HTRF-Based SLP76 Phosphorylation Assay in Human Cell Lines: Jurkat MAP4K1 WT and Jurkat MAP4K1 KO Cell Lines

Phosphorylation assays were carried out in Jurkat E6.1 cells from the ATCC (TIB-152) stably overexpressing human FLAG-tagged SLP-76 (proprietary, Jurkat MAP4K1 WT) or in Jurkat MAP4K1 KO cells. Cultured cells were kept in RPMI 1640 medium (Biochrom cat. no. F1275, without phenol red) supplemented with 1% FCS (Biochrom cat. no. S0615) at a cell density of 2 × 10 × 10^6^/mL 24 h prior to compound testing. Starved cells were transferred to a 384-well format plate at a cell density of 140.000 cells/well and simultaneously treated with 1 μg/mL anti-CD3 antibody (clone OKT3. eBioscience #16–0037–85) and 4 μg/mL anti-IgG cross-linking antibody (Invitrogen goat antimouse IgG (H + L) 2 #31160) together with the test compound for 30 min at 37 °C. Compounds were tested a 9-point dose response titration of compound concentration with 20, 8.43, 3.56, 2.0, 1.0, 0.632, 0.267, 0.112, 0.02 μM in triplicate. The cells were washed once in PBS (pH 7.4). The detection of pSer376-SLP76 levels in the cells was carried out utilizing an adapted protocol of the HTRF pSLP76 Assay (Cisbio # 63ADK076PEG). Cells were lysed using 4 μL of the supplemented lysis buffer (Cisbio no. 63ADK076PEG) for 60 min at room temperature. Subsequently, 4 μL of the premixed antibody solution (Cisbio no. 63ADK076PEG) was added and incubated overnight at room temperature. Read-out and analyses were carried out using Pherastar and the MARS software (BMG Labtechnologies, Offenburg, Germany).

As control for maximal effect (max control, which represents the maximally possible inhibition of pSer376-SLP-76 by a test compound), cells with no anti-CD3 (clone OKT3. eBioscience #16–0037–85) and no test compound treatment were used. Cells with anti-CD3 treatment only were used as a negative control (min control represents the minimally possible inhibition of pSer376-SLP-76 by a test compound).

### HTRF-Based Phosphorylation Assay in Primary Human PBMCs

For phosphorylation assays, primary human PBMCs were thawed and allowed to recover for 5 h in an X-Vivo 20 medium (Lonza, #BE04–448Q). PBMCs were transferred to RPMI 1640 medium (Biochrom cat. no. F1275, without phenol red) supplemented with 1% FCS (Biochrom cat. no. S0615) for 12 h. Starved cells were transferred to a 384-well format plate at a cell density of 140.000 cells/well and simultaneously treated with 1 μg/mL anti-CD3 antibody (clone OKT3. eBioscience #16–0037–85) and 4 μg/mL anti-IgG cross-linking antibody (Invitrogen goat antimouse IgG (H + L) 2 #31160) together with the test compound and 1 μM PGE2 for 30 min at 37 °C. The further assay was carried out as described in pSer376-SLP76 testing in human cell lines.

### Immunoblotting-Based Phosphorylation Assay of in Ex Vivo-Stimulated Murine Splenocytes: C57BL WT Mouse and C57BL MAP4K1 K46 M Kinase-Dead Mouse (MAP4K1 KI Mouse)

To validate the MAP4K1 kinase-dead mutant in the MAP4K1 KI mouse, spleens were harvested either from C57BL wild-type or from MAP4K1 KI animals and splenocyte suspension was generated by filter spleen tissue through 70 μm filters, spinning at 300*g*/8 min, and resuspending in erythrocytes lysis buffer for 10 min. Splenocytes were washed and resuspended in RPMI 1640 medium supplemented with 1% FCS and cell counts were adjusted to 8 × 10^6^ cells/mL. Cells were activated with aCD3 and aCD28 (eBioscience antimouse CD3 clone 17A2 #14–0032,82; antimouse CD28 clone 37.51 #16–0281,85) with a final 1 μg/mL concentration for 60 min at 37 °C. Splenocytes were collected and immediately lysed in ice-cold lysis buffer for 1 h at 4 °C. Protein concentrations were determined using the Pierce BCA test. Immunoblotting was carried out using 10 μg of protein per lane. For protein denaturation, DTT and 4x LDS were added and heated for 10 min at 70 °C. For antibody detection, proteins were transferred to membranes. Respective membranes were processed and separately incubated with primary antibodies proprietary antimouse pSer376-SLP76 antibody ASH-1–13–5 (rabbit mAb, custom-made by Abcam), SLP76 (Cell Signaling #4958), MAP4K1 (HPK1, Cell Signaling #4472), and GAPDH (Zytomed #RGM2–6C5). For secondary detection, antibody staining membranes were washed 5 × 5 min with TBS-T 0,1% and incubated for 1 h at RT with donkey antirabbit (IRDye 800CW, LI COR# 92632213) or goat antirabbit IgG (Alexa Flour 680, Invitrogen # A21058), respectively. Then membranes were once washed in fully desalinated water and analyzed using an Invitrogen iBright and Li-cor Odyssey.

### Immunoblotting-Based Phosphorylation Assay of Murine Spleen from Mouse Tumor Experiments: EMT-6 Model

To determine the compound effect with respect to reduction of phosphor-Ser376-SLP76 in EMT6 tumor-bearing mice, fresh spleens were harvested from either vehicle- or BAY-405-treated mice at day 21 of the tumor experiment 1 h after the last compound treatment. Test groups of vehicle-treated, 60 mg/kg bid-treated, or 100 mg/kg bid-treated animals consisted of 8 animals each. Fresh mouse spleen tissue was lysed in ice-cold 400 μL of CST lysis buffer incl. complete/PhosSTOP/HALT and disrupted using Qiashredder 3 min 30/s, lysed for 30 min on ice, and cleared by spinning down 15 min/4 °C/21,000 rcf. Protein concentrations were determined using the Pierce BCA test. Immunoblotting was carried out using 10 μg of protein per lane. For protein denaturation, DTT and 4x LDS were added and heated for 10 min at 70 °C. For antibody detection, proteins were transferred to membranes. Respective membranes were processed and separately incubated with primary antibodies as a proprietary antimouse pSer376-SLP76 antibody ASH-1–13–5 (rabbit mAb, custom-made by Abcam), SLP76 (Cell Signaling #4958), MAP4K1 (HPK1, Cell Signaling #4472), and GAPDH (Zytomed #RGM2–6C5), respectively. For secondary detection antibody staining, membranes were washed 5 × 5 min with TBS-T 0,1% and incubated for 1 h at RT with donkey antirabbit (IRDye 800CW, LI COR# 92632213) or goat antirabbit IgG (Alexa Flour 680, Invitrogen # A21058), respectively. Then membranes were once washed in fully desalinated water and analyzed using Invitrogen iBright and Li-cor Odyssey.

### FACS-Based Phosphorylation Assay in Ex Vivo-Stimulated Murine Splenocytes: C57BL WT Mouse and C57BL Map4k1 K46 M Kinase-Dead Mouse (MAP4K1 KI Mouse)

To determine the compound effect with respect to phosphorylation inhibition in murine splenocytes, fresh mouse splenocytes were generated by filter spleen tissue using a 70 μm filter, spinning at 300*g*/8 min, and resuspending in erythrocytes lysis buffer for 10 min. Splenocytes were washed and resuspended in RPMI 1640 medium supplemented with 1% FCS. Cells were activated with antimouse CD3 and CD28 (eBioscience antimouse CD3 clone 17A2 #14–0032,82; antimouse CD28 clone 37.51 #16–0281,85) with final 1 μg/mL together with the test compound for 60 min at 37 °C. Compounds were tested a 9-point dose response titration of compound concentration with 20, 8.43, 3.56, 2.0, 1.0, 0.632, 0.267, 0.112, 0.02 μM in triplicate. For FACS analyses, cells were incubated for 10 min at 37 °C in Lys/Fix Buffer (BD #55849), washed, and incubated for 10 min at 37 °C in Perm/Wash Buffer (BD #557885). In a first staining step, cells were incubated with a proprietary antimouse pSer376-SLP76 antibody ASH-1-13-5 (rabbit mAb, custom-made by Abcam) for 1 h at room temperature in Perm/Wash Buffer. Cells were washed and stained in a second step with a mix of αCD4 BUV737 (BD #564933), αCD8 PE Cy7 (Biolegend #100722), and antirabbit 647 (Invitrogen #A21244 647) in Perm/Wash Buffer for 1 h at room temperature. Labeled cells were analyzed using a BD Fortessa flow cytometer and were evaluated with FlowJo software.

### Human PBMC In Vitro Activation Assay

The effect of the compound on the activation of human T cells was tested by measuring the production of the proinflammatory cytokine IFNγ in vitro. Human PBMCs were isolated and activated in vitro with coated anti-CD3 (clone OKT3. eBioscience #16-0037-85). The anti-CD3 antibodies were coated in the plates by adding 50 μL of the suspended reagent. Concentration of anti-CD3 was titrated in order to obtain a suboptimal activation of PBMCs. Cells were seeded at 2 × 10^6^ PBMCs/mL in X-VIVO 20 medium (Lonza) and activated with suboptimal concentration of anti-CD3 (100 ng/mL) and 1 μM PGE2 or 20 ng/mL TGFβ for 22 h in the presence of the compounds. The supernatant of the culture was isolated and tested for the IFNγ concentration. Applied compounds were tested at either a fixed concentration of 1000 nM or in a dose response titration of increased compound concentration in triplicate.

### Mouse Splenocyte In Vitro Activation Assay

Fresh mouse splenocytes were isolated and activated in vitro with coated anti-CD3. The anti-CD3 antibodies were coated in the plates by adding 50 μL of the suspended reagent. The concentration of anti-CD3 was titrated in order to obtain a suboptimal activation of the splenocytes. Cells were seeded (2 × 10^6^ PBMCs/mL) in RPMI-1640 medium supplemented with 10% FCS, 2 nM glutamine, and activated with suboptimal concentration of anti-CD3 (500 ng/mL) and 1 μM PGE2 or 10 ng/mL TGFβ for 22 h in the presence of the compounds, and the supernatant of the culture was isolated and tested for IFNγ concentration by ELISA. Applied compounds were tested at either a fixed concentration of 1000 nM or in a dose response titration of increased compound concentration in triplicate.

### Human MART-1 T-Cell Assay

MART-1 ELAGIGILTV/A2 TCR (DMF5)^48^ was transduced into human PBMC. DMF5-positive cells were expanded in the presence of PBMCs feeder cells loaded with the MART-1 peptide (ProImmune) and IL2 (70 IU/mL) for 14 days; >80% of T cells were DMF5 positive after the procedure.

To analyze the modulatory activity of inhibitors on the immunosuppressive function of MAP4K1 in vitro, the MART-1-peptide-specific CD8^+^ T cells were cocultivated together with COLO 800 melanoma cells. IFNγ secretion was measured as readout for T-cell activity by IFNγ ELISA. For the coculture, COLO 800 tumor cells were detached nonenzymatically using PBS-EDTA for 5 min, centrifuged, washed, and counted. Cell concentration was adjusted to 1 × 10^6^ cells/mL in X-Vivo-20 (Lonza) and 40,000 tumor cells were seeded in triplicate to 96-Well plates. In the meantime, MART-1-peptide-specific T cells were harvested, washed with X-Vivo-20, and seeded at 40.000 cells per well. The coculture of tumor cells and T cells was incubated for 20–26 h at 37 °C.

Cell-mediated cytotoxicity was analyzed in an impedance-based cytotoxicity assay (xCELLigence) system.^49^ In this label-free assay system, cytotoxicity is measured directly and continuously over a period of around 96–120 h (real time). 10,000 adherent COLO 800 tumor cells are attached to microelectrodes at the bottom of a 96-Well E-plate (E-Plate VIEW 96 PET; ACEA Biosciences #ID:H000568), which changes the electrical impedance of these electrodes. This is monitored as an increase of the dimensionless “cell index”. After adherence of the tumor cells (−24 h), MAP4K1 compounds and 10,000 MART-1-specific T cells are added to the wells, which results in lysis of the tumor cells and detachment from the electrodes. This detachment changes the impedance of the wells and is measured as a decrease in the cell index or normalized cell index (the cell index normalized to the time point of T-cell addition). The T cells alone do not affect the electrical impedance of the electrodes, and thus only the cytolysis of the tumor cells is measured.

### Drug Formulation for In Vivo Use; Vehicle; Oral Dosing Protocol

For the in vivo PK experiment, BAY-405 was administered intravenously at a dose of 1 mg/kg as a solution in PEG400/EtOH/Water (5 mL/kg) to three female CD1 (crl:CD1(lcr)) mice. Nine samples (2, 5, 15, 30, and 60 min; 2, 4, 7, 24 h) were taken per animal via a jugular vein catheter.

Bioanalysis of in vivo PK samples was performed by LC/MS/MS employing triple quadrupole mass spectrometers from Sciex (e.g., API5000, API5500, or API6500) and liquid chromatography systems from Agilent (1260) or Waters (UPLC) coupled via a standard electrospray source using positive ion mode.

An aliquot of 100 μL of mouse plasma was taken and precipitated by addition of 400 μL of cold acetonitrile containing an internal standard of choice and frozen at −20 °C overnight. Samples were subsequently thawed and centrifuged at 3000 rpm, 4 °C for 20 min and transferred to bioanalysis.

Applying QC samples, each validation and study run is validated according to internal SOPs, which are based on FDA’s and EMA’s guidance for bioanalysis. The calibration range covered roughly 0.001 to 5 μM and can be extended by sample dilution up to 500 μM.

According to internal SOPs, study samples are analyzed by employing internally validated LC/MS/MS methods. Column oven temperature is kept at 40 °C; injection volume amounts to 5 μL on the column; and eluent flow amounts to 500 to 650 μL/min. For example, a Phenomenex Kinetex reversed phase C18 column (2.6 μm, 2.1 mm × 50 mm) and a gradient consisting of 5 to 95% acetonitrile (0.5% acetic acid) and water (0.1% acetic acid) is applied within three min, followed by one min of re-equilibration and subsequent injection of the next sample. Each run is initiated by solvent blanks and a system suitability test prior to sample analysis. Calibration samples are analyzed in the beginning and at the end of each run, and QC samples are distributed over the run. After five–ten sample injections, a solvent blank is included. Concentration data are calculated based on the internal standard method and are reported in μM.

### B16-OVA Lung Colonization Assay

0.2 × 10^6^ OVA-expressing B16 melanoma cells were injected i.v. in C57BL/6N Ly5.1 and MAP4K1 KI mice. The next day, treatment with vehicle or MAP4K1 inhibitor was started. After 14 days, the mice were sacrificed and the lungs were isolated and washed in PBS. The number of B16-OVA nodules in the lungs were measured by the researcher.

### B16-OVA Subcutaneous Tumor Model

0.5 × 10^6^ OVA-expressing B16 melanoma cells were injected s.c. in the flank of host mice (C57BL/6N Ly5.1 or NSG mice). Mouse weight and tumor sizes were measured with a caliper every 2–3 days, and tumor volume was calculated according to the following formula: volume = length × width × width/2. OT-I adoptive therapy was performed when a tumor reached 50 mm3 in volume. Five × 10^6^ OT-I splenocytes were injected i.v. in 100 μL of PBS. Treatment with BAY-405 was performed by oral gavage, dissolving the compound in 100 μL of 50% solutol, 40% water, and 10% ethanol. Treatment was performed bidaily. Mice were sacrificed when the tumor length reached 15 mm or when analysis of the tumor was performed. Measurements were performed by a researcher unaware of the allocation of mice into groups.

### EMT6 Subcutaneous Tumor Model

Female Balb/c mice (Balb/c Ola, Envigo, The Netherlands) were subcutaneously injected with 5 × 10^5^ ETM6 cells (murine breast cancer, ATCC) in PBS (100 μL/animal) into the right flank. After approximately 5–7 days when tumors reached a volume of about 100 mm3, mice were randomized and grouped with *n* = 10 mice per group. When CD8^+^ T-cell depletion was performed, animals received 8 mg/kg of an anti-CD8 depletion Ab (clone YTS169, BioXcell) 1 day before tumor inoculation. BAY 405, formulated in solutol–ethanol–water (40/10/50) was applied orally twice daily with a minimum interval of 8 h. Body weights and tumor volumes were measured at least three times per week. All animal studies were conducted under the German Animal Welfare conduct of law.

### Tumor Isolation

Tumor samples for analysis were isolated from mice either at day 4 after treatment or at the end of the tumor efficacy experiment, before the control group reached 15 mm in length. Tumors were analyzed at day 13, 6 d after the depletion Treg cells or 2 d after the cotransfer.

B16-OVA tumors were isolated from the mice and placed in a 40 μm filter above a 50 mL Falcon tube. Tissues were disrupted by the use of a syringe through the filter. Erythrocytes were removed by incubation for 1 min with ACK buffer (Dulbecco’s PBS containing 0.15 M NH_4_Cl, 10 mM KHCO_3_ and 0.1 mM EDTA). Cells were then washed with PBS to generate a cell suspension.

### Origin of Human Tumor Samples

Tumor samples from gastric, colorectal, melanoma, and lung cancer patients were obtained via Provitro GmbH (Berlin, Germany) with informed consent and with the approval of the local authorities.

### Immunohistochemistry for MAP4K1, PD-L1, and CD8

50 tumor samples per indication were analyzed on tissue micro arrays (TMAs). Three μm in size of the respective TMAs were treated for 17 min in Target Retrieval Solution pH 9 (Dako S2367) in a steam cooking device. Sections were then stained using the respective antibodies PD-L1: Ventana rabbit antihuman PD-L1 antibody, clone SP263 (Ventana 741–4905) at a 0.4 μg/mL final concentration; MAP4K1: mouse antihuman MAP4K1 clone 2A1, (Origene AM31910SU-N) at a 0.25 μg/mL final concentration; CD8: DAKO mouse antihuman CD8 Clone C8/144B, (DAKO #M7103) at a 1:500 dilution in DAKO antibody diluent (S3022) for 60 min at RT. Primary antibodies were detected using either antirabbit Envision secondary antibody (DAKO K4010) or antimouse Envision secondary antibody (Dako #K4001) for 30 min at RT and DAB-Substrate (Dako K3468) for 10 min at RT. Stained slides were scanned using a Mirax P250 slide scanner and scored manually for the presence of the different biomarkers. The scoring of immune infiltrates was based on the frequency of positive cells and not on the intensity of the staining.

## Supporting Information Available

Analysis of human and mouse MAP4K1 orthologues; species selectivity and biophysics of BAY-755 and BAY-405; selectivity profiling of BAY-755 and BAY-405 Eurofins kinase enzyme panel; evaluation of kinases showing over 80% inhibition by BAY-405; cellular kinase selectivity profiling and inhibition of MAP4K1 activity by BAY-405 in different assays; pharmacokinetic properties of BAY-405 after IV administration; crystallization data collection and refinement statistics; exposure of BAY-405 single dose; generation of MAP4K1-deficient Jurkat cells and kinase-dead knock-in mice; in vitro pharmacology; in vitro and in vivo anti-tumor T-cell reactivity; exposure of BAY-405 in EMT6 efficacy experiments; experimental methods; synthesis of compounds **1**–**38**; and HPLC-MS trace for compound **38** (PDF)
Crystallographic data of the compounds (CSV)

## Abbreviations

^1^H NMR: proton nuclear magnetic resonance
CL_b_: hepatic in vivo blood clearance
CTLA4: cytotoxic T-lymphocyte-associated Protein 4
cyno: cynomolgus monkey
d: dog
DCM: dichloromethane
DMF: dimethylformamide
DMPK: drug metabolism and pharmacokinetics
DMSO: dimethyl sulfoxide
EDC: 1-ethyl-3-(3-(dimethylamino)propyl)carbodiimide
Et: ethyl
*F*: bioavailability
HMDS: hexamethyldisilazane
HPK1: Hematopoietic progenitor kinase 1
HPLC: high performance liquid chromatography
HTS: high throughput screen
IC_50_: half-maximum inhibitory concentration
ICI: Immune checkpoint inhibitor
LDA: lithium diisopropylamide
m: mouse
MAP4K1: itogen-activated protein kinase kinase kinase kinase 1
mCPBA: *meta*-chloroperbenzoic acid
MeCN: acetonitrile
MeOH: methanol
MS: mass spectrometry
PD-1: programmed cell death protein 1
PD-L1: programmed death-ligand 1
PGE2: prostaglandin E2
Pr: propyl
Py: pyridine
r: rat
r.t.: room temperature
SAR: structure activity relationship
SEM: trimethylsilylethoxymethyl
SEMCl: 2-(trimethylsilyl)ethoxymethyl chloride
*t*_1/2_: half-life
TCR: T-cell receptor, TGFβ, Transforming growth factor beta
TFA: trifluoroacetic acid
THF: tetrahydrofuran
*T*_s_: toluenesulfonate
*V*_ss_: steady-state volume of distribution

## References

[ref1] Offringa R.; Kotzner L.; Huck B.; Urbahns K. The expanding role for small molecules in immuno-oncology. Nat. Rev. Drug Discovery 2022, 21 (11), 821–840. 10.1038/s41573-022-00538-9. 35982333

[ref2] Wei S. C.; Duffy C. R.; Allison J. P. Fundamental Mechanisms of Immune Checkpoint Blockade Therapy. Cancer Discovery 2018, 8 (9), 1069–1086. 10.1158/2159-8290.CD-18-0367. 30115704

[ref3] Boomer J. S.; Tan T. H. Functional interactions of HPK1 with adaptor proteins. J. Cell. Biochem. 2005, 95 (1), 34–44. 10.1002/jcb.20401. 15770651

[ref4] Hu M. C.; Qiu W. R.; Wang X.; Meyer C. F.; Tan T. H. Human HPK1, a novel human hematopoietic progenitor kinase that activates the JNK/SAPK kinase cascade. Genes Dev. 1996, 10 (18), 2251–2264. 10.1101/gad.10.18.2251. 8824585

[ref5] Ling P.; Meyer C. F.; Redmond L. P.; Shui J. W.; Davis B.; Rich R. R.; Hu M. C.; Wange R. L.; Tan T. H. Involvement of hematopoietic progenitor kinase 1 in T cell receptor signaling. J. Biol. Chem. 2001, 276 (22), 18908–18914. 10.1074/jbc.M101485200. 11279207

[ref6] Shui J. W.; Boomer J. S.; Han J.; Xu J.; Dement G. A.; Zhou G.; Tan T. H. Hematopoietic progenitor kinase 1 negatively regulates T cell receptor signaling and T cell-mediated immune responses. Nat. Immunol. 2007, 8 (1), 84–91. 10.1038/ni1416. 17115060

[ref7] Bartolo V. D.; Montagne B.; Salek M.; Jungwirth B.; Carrette F.; Fourtane J.; Sol-Foulon N.; Michel F.; Schwartz O.; Lehmann W. D.; et al. A novel pathway down-modulating T cell activation involves HPK-1-dependent recruitment of 14–3-3 proteins on SLP-76. J. Exp. Med. 2007, 204 (3), 681–691. 10.1084/jem.20062066. 17353368 PMC2137917

[ref8] Lasserre R.; Cuche C.; Blecher-Gonen R.; Libman E.; Biquand E.; Danckaert A.; Yablonski D.; Alcover A.; Di Bartolo V. Release of serine/threonine-phosphorylated adaptors from signaling microclusters down-regulates T cell activation. J. Cell Biol. 2011, 195 (5), 839–853. 10.1083/jcb.201103105. 22105350 PMC3257567

[ref9] Sauer K.; Liou J.; Singh S. B.; Yablonski D.; Weiss A.; Perlmutter R. M. Hematopoietic progenitor kinase 1 associates physically and functionally with the adaptor proteins B cell linker protein and SLP-76 in lymphocytes. J. Biol. Chem. 2001, 276 (48), 45207–45216. 10.1074/jbc.M106811200. 11487585

[ref10] Alzabin S.; Pyarajan S.; Yee H.; Kiefer F.; Suzuki A.; Burakoff S.; Sawasdikosol S. Hematopoietic progenitor kinase 1 is a critical component of prostaglandin E2-mediated suppression of the anti-tumor immune response. Cancer Immunol., Immunother. 2010, 59 (3), 419–429. 10.1007/s00262-009-0761-0. 19787351 PMC2798028

[ref11] Sawasdikosol S.; Zha R.; Yang B.; Burakoff S. HPK1 as a novel target for cancer immunotherapy. Immunol. Res. 2012, 54 (1–3), 262–265. 10.1007/s12026-012-8319-1. 22477524

[ref12] Chuang H. C.; Wang X.; Tan T. H. MAP4K Family Kinases in Immunity and Inflammation. Adv. Immunol. 2016, 129, 277–314. 10.1016/bs.ai.2015.09.006. 26791862

[ref13] Hernandez S.; Qing J.; Thibodeau R. H.; Du X.; Park S.; Lee H. M.; Xu M.; Oh S.; Navarro A.; Roose-Girma M.; et al. The Kinase Activity of Hematopoietic Progenitor Kinase 1 Is Essential for the Regulation of T Cell Function. Cell Rep. 2018, 25 (1), 80–94. 10.1016/j.celrep.2018.09.012. 30282040

[ref14] Linney I. D.; Kaila N. Inhibitors of immuno-oncology target HPK1 - a patent review (2016 to 2020). Expert Opin. Ther. Pat. 2021, 31 (10), 893–910. 10.1080/13543776.2021.1924671. 33956554

[ref15] Zhou L.; Wang T.; Zhang K.; Zhang X.; Jiang S. The development of small-molecule inhibitors targeting HPK1. Eur. J. Med. Chem. 2022, 244, 114819 10.1016/j.ejmech.2022.114819. 36209628

[ref16] Zhu Q.; Chen N.; Tian X.; Zhou Y.; You Q.; Xu X. Hematopoietic Progenitor Kinase 1 in Tumor Immunology: A Medicinal Chemistry Perspective. J. Med. Chem. 2022, 65 (12), 8065–8090. 10.1021/acs.jmedchem.2c00172. 35696642

[ref17] Goller A. H.; Kuhnke L.; Montanari F.; Bonin A.; Schneckener S.; Ter Laak A.; Wichard J.; Lobell M.; Hillisch A. Bayer’s in silico ADMET platform: a journey of machine learning over the past two decades. Drug Discovery Today 2020, 25 (9), 1702–1709. 10.1016/j.drudis.2020.07.001. 32652309

[ref18] Schirok H. R. M.; Mittendorf J.; Kast R.; Stasch J.-P.; Gnoth M. J.; Muenter K.; Lang D.; Figeuro P. S.; Thutewohl M.; Bennabi S.; Ehmke. Heteroaryloxy-substituted phenylaminopyrimidines as Rho-kinase inhibitors, WO 2004039796 A1

[ref19] Julian L.; Olson M. F. Rho-associated coiled-coil containing kinases (ROCK): structure, regulation, and functions. Small GTPases 2014, 5, e29846 10.4161/sgtp.29846. 25010901 PMC4114931

[ref20] Kanoh S. N. M.; Naka M.; Nishimura T.; Motoi M. Isomerization of cyclic ethers having a carbonyl functional group: new entries into different heterocyclic compounds. Tetrahedron 2002, 58, 7049–7064. 10.1016/s0040-4020(02)00701-9.

[ref21] Schirok H.; Paulsen H.; Kroh W.; Chen G.; Gao P. Improved Synthesis of the Selective Rho-Kinase Inhibitor 6-Chloro-N4-{3,5-difluoro-4-[(3-methyl-1H-pyrrolo[2,3-b]pyridin-4-yl)oxy]phenyl}pyrimidin-2,4-diamine. Org. Process Res. Dev. 2010, 14 (1), 168–173. 10.1021/op900260k.

[ref22] Martino J.; Siri S. O.; Calzetta N. L.; Paviolo N. S.; Garro C.; Pansa M. F.; Carbajosa S.; Brown A. C.; Bocco J. L.; Gloger I.; et al. Inhibitors of Rho kinases (ROCK) induce multiple mitotic defects and synthetic lethality in BRCA2-deficient cells. Elife 2023, 12, e80254 10.7554/eLife.80254. 37073955 PMC10185344

[ref23] Sambandam A.; Storm E.; Tauc H.; Hackney J. A.; Garfield D.; Caplazi P.; Liu J.; Zhang J.; Zhang H.; Duggan J.; et al. Obligate role for Rock1 and Rock2 in adult stem cell viability and function. Heliyon 2023, 9 (3), e14238 10.1016/j.heliyon.2023.e14238. 36950615 PMC10025895

[ref24] Awale M.; Riniker S.; Kramer C. Matched Molecular Series Analysis for ADME Property Prediction. J. Chem. Inf. Model. 2020, 60 (6), 2903–2914. 10.1021/acs.jcim.0c00269. 32369360

[ref25] Dossetter A. G.; Griffen E. J.; Leach A. G. Matched molecular pair analysis in drug discovery. Drug Discovery Today 2013, 18 (15–16), 724–731. 10.1016/j.drudis.2013.03.003. 23557664

[ref26] O’Boyle N. M.; Bostrom J.; Sayle R. A.; Gill A. Using matched molecular series as a predictive tool to optimize biological activity. J. Med. Chem. 2014, 57 (6), 2704–2713. 10.1021/jm500022q. 24601597 PMC3968889

[ref27] Bosc N.; Meyer C.; Bonnet P. The use of novel selectivity metrics in kinase research. BMC Bioinf. 2017, 18 (1), 17 10.1186/s12859-016-1413-y. PMC5217660 28056771

[ref28] Malumbres M. Physiological relevance of cell cycle kinases. Physiol. Rev. 2011, 91 (3), 973–1007. 10.1152/physrev.00025.2010. 21742793

[ref29] Bolen J. B.; Brugge J. S. Leukocyte protein tyrosine kinases: potential targets for drug discovery. Annu. Rev. Immunol. 1997, 15, 371–404. 10.1146/annurev.immunol.15.1.371. 9143693

[ref30] Klaeger S.; Heinzlmeir S.; Wilhelm M.; Polzer H.; Vick B.; Koenig P. A.; Reinecke M.; Ruprecht B.; Petzoldt S.; Meng C.; et al. The target landscape of clinical kinase drugs. Science 2017, 358 (6367), eaan4368 10.1126/science.aan4368. 29191878 PMC6542668

[ref31] Haanen J. B. A. G.; Carbonnel F.; Robert C.; Kerr K. M.; Peters S.; Larkin J.; Jordan K.; Committee E. G. Management of toxicities from immunotherapy: ESMO Clinical Practice Guidelines for diagnosis, treatment and follow-up. Ann. Oncol. 2017, 28, IV119 10.1093/annonc/mdx225. 28881921

[ref32] Ramos-Casals M.; Brahmer J. R.; Callahan M. K.; Flores-Chavez A.; Keegan N.; Khamashta M. A.; Lambotte O.; Mariette X.; Prat A.; Suarez-Almazor M. E. Immune-related adverse events of checkpoint inhibitors. Nat. Rev. Dis. Primers 2020, 6 (1), 38 10.1038/s41572-020-0160-6. 32382051 PMC9728094

[ref33] Schirok H. Microwave-assisted flexible synthesis of 7-azaindoles. J. Org. Chem. 2006, 71 (15), 5538–5545. 10.1021/jo060512h. 16839132

[ref34] Schirok H. A Versatile Synthesis of 7-Azaindoles. Synlett 2005, 2005, 1255–1258. 10.1055/s-2005-865243.

[ref35] Schirok H.; Figueroa-Perez S.; Thutewohl M.; Paulsen H.; Kroh W.; Klewer D. Synthesis and Derivatization of 3-Perfluoroalkyl-Substituted 7-Azaindoles. Synthesis 2007, 38, 251 10.1002/chin.200720135.

[ref36] Nakahara K.; Fuchino K.; Komano K.; Asada N.; Tadano G.; Hasegawa T.; Yamamoto T.; Sako Y.; Ogawa M.; Unemura C.; et al. Discovery of Potent and Centrally Active 6-Substituted 5-Fluoro-1,3-dihydro-oxazine β-Secretase (BACE1) Inhibitors via Active Conformation Stabilization. J. Med. Chem. 2018, 61 (13), 5525–5546. 10.1021/acs.jmedchem.8b00011. 29775538

[ref37] Schirok H.; Kast R.; Figueroa-Perez S.; Bennabi S.; Gnoth M. J.; Feurer A.; Heckroth H.; Thutewohl M.; Paulsen H.; Knorr A.; et al. Design and synthesis of potent and selective azaindole-based Rho kinase (ROCK) inhibitors. ChemMedChem 2008, 3 (12), 1893–1904. 10.1002/cmdc.200800211. 18973168

[ref38] Gyárfás P.; Gerencsér J.; Wade W. S.; Ürögdi L.; Novák Z.; Meyer S. T. Efficient Synthesis of 3,4-Disubstituted 7-Azaindoles Employing SEM as a Dual Protecting-Activating Group. Synlett 2019, 30 (20), 2273–2278. 10.1055/s-0039-1690735.

[ref39] Zhang P.; Le C. C.; MacMillan D. W. Silyl Radical Activation of Alkyl Halides in Metallaphotoredox Catalysis: A Unique Pathway for Cross-Electrophile Coupling. J. Am. Chem. Soc. 2016, 138 (26), 8084–8087. 10.1021/jacs.6b04818. 27263662 PMC5103281

[ref40] Phelan J. P.; Lang S. B.; Compton J. S.; Kelly C. B.; Dykstra R.; Gutierrez O.; Molander G. A. Redox-Neutral Photocatalytic Cyclopropanation via Radical/Polar Crossover. J. Am. Chem. Soc. 2018, 140 (25), 8037–8047. 10.1021/jacs.8b05243. 29916711 PMC6540794

[ref41] Mueller U.; Förster R.; Hellmig M.; Huschmann F. U.; Kastner A.; Malecki P.; Pühringer S.; Röwer M.; Sparta K.; Steffien M.; et al. The macromolecular crystallography beamlines at BESSY II of the Helmholtz-Zentrum Berlin: Current status and perspectives. Eur. Phys. J. Plus 2015, 130, 141 10.1140/epjp/i2015-15141-2.

[ref42] Kabsch W. XDS. Acta Crystallogr. 2010, 66, 125–132. 10.1107/S0907444909047337. PMC2815665 20124692

[ref43] Sparta K. M.; Krug M.; Heinemann U.; Mueller U.; Weiss M. S. XDSAPP2.0. J. Appl. Crystallogr. 2016, 49, 1085–1092. 10.1107/s1600576716004416.

[ref44] Winn M. D.; Murshudov G. N.; Papiz M. Z. Macromolecular TLS refinement in REFMAC at moderate resolutions. Methods Enzymol. 2003, 374, 300–321. 10.1016/S0076-6879(03)74014-2. 14696379

[ref45] Emsley P.; Lohkamp B.; Scott W. G.; Cowtan K. Features and Development of Coot. Acta Crystallogr., Sect. D: Biol. Crystallogr. 2010, 66, 486–501. 10.1107/S0907444910007493. 20383002 PMC2852313

[ref46] Halgren T. A.; Murphy R. B.; Friesner R. A.; Beard H. S.; Frye L. L.; Pollard W. T.; Banks J. L. Glide: a new approach for rapid, accurate docking and scoring. 2. Enrichment factors in database screening. J. Med. Chem. 2004, 47 (7), 1750–1759. 10.1021/jm030644s. 15027866

[ref47] Gasteiger J. R. C.; Rudolph C.; Sadowski J. Automatic generation of 3D-atomic coordinates for organic molecules. Tetrahedron Comput. Methodol. 1990, 3 (6), 537–547. 10.1016/0898-5529(90)90156-3.

[ref48] Johnson L. A.; Morgan R. A.; Dudley M. E.; Cassard L.; Yang J. C.; Hughes M. S.; Kammula U. S.; Royal R. E.; Sherry R. M.; Wunderlich J. R.; et al. Gene therapy with human and mouse T-cell receptors mediates cancer regression and targets normal tissues expressing cognate antigen. Blood 2009, 114 (3), 535–546. 10.1182/blood-2009-03-211714. 19451549 PMC2929689

[ref49] Peper J. K.; Schuster H.; Loffler M. W.; Schmid-Horch B.; Rammensee H. G.; Stevanovic S. An impedance-based cytotoxicity assay for real-time and label-free assessment of T-cell-mediated killing of adherent cells. J. Immunol. Methods 2014, 405, 192–198. 10.1016/j.jim.2014.01.012. 24486140

