# Supplementary material for: Discovery of BAY-405: An Azaindole-Based MAP4K1 Inhibitor for the Enhancement of T-Cell Immunity against Cancer
Source: J Med Chem. 2024 Sep 27;67(19):17429–53. doi: 10.1021/acs.jmedchem.4c01325 (PMC11472321; doi:10.1021/acs.jmedchem.4c01325)
Supplement: Supplementary file 1 — jm4c01325_si_001.pdf [file jm4c01325_si_001.pdf]

Supporting Information

Discovery of BAY-405: An Azaindole-based MAP4K1 Inhibitor for the Enhancement of T-Cell Immunity against Cancer

Jeffrey Mowat[a], Rafael Carretero[a],[b], Gabriele Leder[a], Nuria Aiguabella Font[a], Roland Neuhaus[b], Sandra Berndt[a], Judith Günther[a], Anders Friberg[a], Martina Schäfer[a], Hans Briem[a], Marian Raschke[a], Hideki Miyatake Ondozabal[a], Bernd Buchmann[a], Ulf Boemer[a], Bertolt Kreft[a], Ingo Hartung[a], and Rienk Offringa[b],[c] * [a] Bayer AG, Pharmaceutical R&D, 13342 Berlin, Germany [b] DKFZ-Bayer Joint Immunotherapeutics Laboratory, German Cancer Research Center, Heidelberg 69120, Germany. [c] Division Molecular Oncology of Gastrointestinal Tumors, Department of Surgery, University Hospital Heidelberg, Heidelberg 69120, Germany. * Corresponding author: r.offringa@dkfz.de

Contents

1. Analysis of human and mouse MAP4K1 orthologues S2
2. Species selectivity and biophysics for BAY-755 and BAY-405 S3
3. Selectivity profiling of BAY-755 in Eurofins kinase enzyme panel S4
4. Selectivity profiling of BAY-405 in Eurofins kinase enzyme panel S6
5. Evaluation of kinases showing > 80% inhibition by BAY-405 S7
6. Cellular kinase selectivity profiling of BAY-405 in KinomeScout ™ assay S13
7. Inhibition of MAP4K1 kinase activity by BAY-405 in cell-based assays S15
8. Pharmacokinetic Properties of BAY-405 after IV administration S16
9. Crystallization data collection and refinement statistics S17
10. Exposure of BAY-405 from single dose S18
11. Generation of MAP4K1-deficient Jurkat T-cells and kinase-dead knock-in mice S19
12. In vitro pharmacology S21
13. In vitro and in vivo anti-tumor T-cell reactivity S23
14. Exposure of BAY-405 in EMT6 efficacy experiment S25
15. Experimental Methods S26
16. Synthesis of Compounds 1-38 S31
17. HPLC-MS Trace for Compound 38 (BAY-405) S154
18. References S155

Figure S1 Analysis of human and mouse MAP4K1 orthologues

A

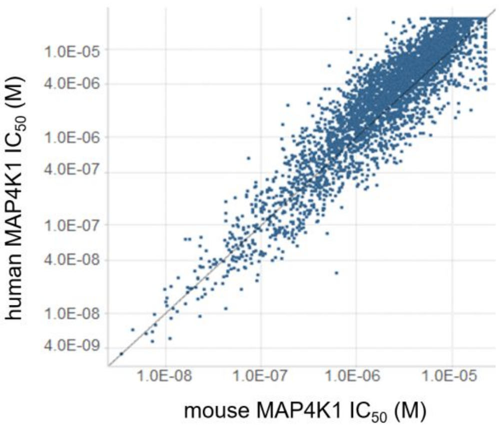

B

Overall

Sequence Name	MAP4K1_human	MAP4K1_rat	MAP4K1_mouse	MAP4K1_macfa
MAP4K1_human	100	83,2	81,9	98,1
MAP4K1_rat	90,1	100	95,4	83,1
MAP4K1_mouse	89,3	97,8	100	82
MAP4K1_macfa	99,4	90,1	89,2	100

Kinase Domain: AA 17 – 274

Sequence Name	MAP4K1_human	MAP4K1_rat	MAP4K1_mouse	MAP4K1_macfa
MAP4K1_human	100	96,1	95	99,2
MAP4K1_rat	98,8	100	97,7	95,3
MAP4K1_mouse	98,1	99,2	100	94,2
MAP4K1_macfa	99,6	98,4	97,4	100

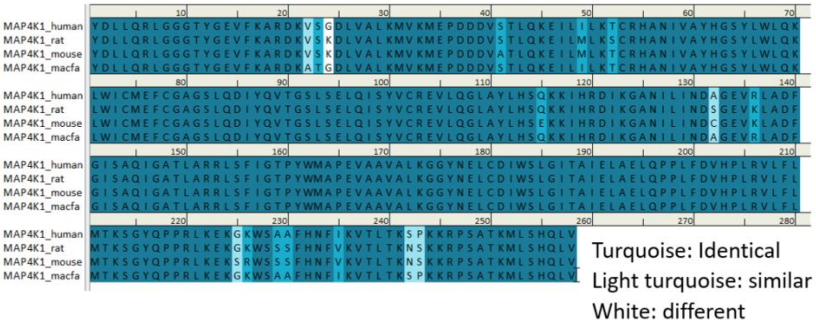

Legend for Figure S1.

A. Comparison of the IC_{50} for the human and mouse MAP4K1 orthologues for the consolidated list of 3,765 compounds exhibiting an IC_{50} of <u><</u>23 µM for human MAP4K1. **B.** Evaluation of sequence identity and similarity of indicated MAP4K1 orthologues.

Table S1. Species selectivity and biophysics for BAY-755 and BAY-405

Summary of the data for compound **1** (BAY-755) and compound **38** (BAY-405) with respect to biochemical kinase activity and binding assays. The biochemical kinase activity assay was performed for human and mouse MAP4K1 at ATP concentrations of 10 µM and 1 mM. Compound binding to the human, mouse, rat and monkey MAP4K1 orthologues was determined by means of a tracer binding competition assay, as well as for human MAP4K1 by surface plasmon resonance (SPR). See *Figure S1B* for a representation of the sequence identity and similarity of MAP4K1 orthologues.

**Assay type \ Orthologue**	**human**	**monkey**	**mouse**	**rat**
**BAY-755**				
**Kinase activity assay**				
IC_{50} / SEM (nM) 10 µM ATP	82 / 30	n.d.	82 / 14	n.d.
IC_{50} / SEM (nM) 1 mM ATP	330 / 206	n.d.	1020 / 50	n.d.
**Tracer binding competition assay**				
IC_{50} / SEM (nM)	75 / 5.6	80 / 8.3	115 / 42	102 / 7.8
**BAY-405**				
**Kinase activity assay**				
IC_{50} / SEM (nM) 10 µM ATP	11 / 2.5	n.d.	17 / 1.7	n.d.
IC_{50} / SEM (nM) 1 mM ATP	56 / 16	n.d.	195 / 34	n.d.
**Tracer binding competition assay**				
IC_{50} / SEM (nM)	6.2 / 0.9	19.1 / 8	12.5 / 4.6	17.1 / 6.8
**SPR**				
K_D (nM)	19.7			
K_{off} (s^{-1})	4.32×10^{-3}			
K_{on} ($M^{-1}s^{-1}$)	2.35×10^{5}			

Table S2. Selectivity profiling of BAY-755 in Eurofins kinase enzyme panel

Left panel. Listing of kinases showing > 80% inhibition by BAY-755 at 1 µM in the presence of 10 µM ATP. Notably, in between the times that BAY-755 and BAY-405 were tested, the kinase panel has been significantly extended (www.eurofinsdiscoveryservices.com). This extension includes MAP4K3 and MRCKgamma, which are listed in *Table S3* as showing >80% inhibition by BAY-405 at 1 µM and therefore also expected to be inhibited to similar or greater extend by BAY-755 **Right panel.** BAY-755 biochemical IC_{50} (nM) at 10 µM ATP for all kinases showing >80% inhibition at 1 µM. The S(80%, 1µM) selectivity score for BAY-755 is calculated on these data as follows. S(x) = number of kinases ≥X/total number of kinases tested = 41 / 318 = 0.13

Kinases showing > 80% inhibition			BAY-755 biochemical IC_{50} (nM)	
**Kinase**	**% inhibition**		**Kinase**	**IC_{50} (nM)**
MELK	102.3		ROCK-II	5
ROCK-II	101.2		PKG1alpha	8
Haspin	101.1		CLK2	10
PKG1beta	100.1		PKG1beta	12
PKACbeta	99.6		MELK	16
Flt4	99.5		PRKG2	17
PKG1alpha	99.5		ROCK-I	24
CLK2	99.1		Flt4	33
PRKG2	98.5		**HPK1 (MAP4K1)**	**36**
CLK4	98.5		CLK1	39
TNIK	98.4		Haspin	43
CLK1	97.5		TNIK	45
ROCK-I	97.3		PKACbeta	48
Flt3	96.7		TrkA	56
TrkA	96.4		KDR	61
**HPK1 (MAP4K1)**	**96**		Flt3	71
PKACalpha	94.9		CLK4	79
MRCKbeta	94.7		TrkC	83
TrkB	94.6		IRR	85
GCK	94.4		PrKX	101
NEK1	92		MRCKbeta	102
IRR	90.9		TTK	111
TTK	90.6		PKACalpha	112

MST2	90.4		PKBgamma	125
TAO1	90.2		TAO1	135
TrkC	90		GCK	146
PKBgamma	89		Flt1	203
PrKX	88.5		MRCKalpha	219
MRCKalpha	88.3		MAP4K4	242
Mer	87.6		IRAK4	243
PKCtheta	86.3		MAP4K5	264
PKBbeta	86.1		TAO3	269
MAP4K5	85.7		p70S6K	269
p70S6K	83.9		TrkB	272
Flt1	83.6		PKBbeta	272
TAO3	83.6		PKCtheta	316
MAP4K4	82.7		Mer	368
KDR	82.5		MST2	380
IRAK4	82.1		Itk	455
IRAK1	81.1		NEK1	503
Itk	80.8		IRAK1	515

Table S3. Selectivity profiling of BAY-405 in Eurofins kinase enzyme panel

BAY-405 (**38**) biochemical IC_{50} (nM) in the presence of 10 µM ATP for all kinases showing >80% inhibition at 1 µM. The S(80%, 1µM) selectivity score for BAY-405 is calculated on these data as follows. S(x) = number of kinases ≥X/total number of kinases tested = 30 / 373 = 0.08

Kinase	BAY-405 biochemical IC_{50} (nM)
**HPK1 (MAP4K1)**	**2**
MAP4K3	13
MAP4K5	20
CK1epsilon	24
TAO1	31
TrkA	33
Mer	43
TrkB	46
PKACbeta	59
Haspin	67
Flt4	78
CLK2	79
KDR	80
PKA	83
CLK4	83
STK16	87
TAO3	110
IRAK4	120
CLK1	160
TrkC	170
IRAK1	170
GCK	190
Flt1	200
MRCKgamma	250
Fes	300
Met	350
Itk	380
FGFR1	380
PTK5	410
MELK	590

Table S4: Evaluation of kinases showing >80% inhibition by BAY-405 in biochemical assay on basis of publicly available data

Nomenclature in left hand column refers to that in *Table S3*. Expression pattern and further information is based on Human Protein Atlas, IST Online, JAX database and referenced publications.

Kinase	Name(s)	IC_{50} nM	Expression pattern	Key physiological functions as reported	Ko-phenotype	Human disease	Ref.
MAP4K1	Mitogen-activated protein kinase kinase kinase kinase 1	2	Hematopoietic cells	Immune cell regulation	Mice: enhanced T-cell response	-	[1-6]
MAP4K3	Mitogen-activated protein kinase kinase kinase kinase 3	13	Various cell types and tissues	Regulation cell metabolism; inhibition autophagy; enhancement T-cell response; promotion cancer metastasis	Cell: autophagy induction Mice: viable; impaired T-cell immune response	Autoimmune disease (high expression in T-cells); Cancer (over-expression in tumor cells)	[2]
MAP4K5	MAP kinase kinase kinase kinase 5	20	Various cell types and tissues	Potential role in TNFR and CD40 downstream signaling	Mice: viable; no specific phenotype described thus far	-	[2]
CK1 epsilon	Casein kinase 1-epsilon (CSNK1E)	24	Various cell types and tissues	Regulation circadian clock; DNA damage-induced signal transduction; genome stability; granulocyte differentiation /function; wnt signaling pathway	Cell: circadian period lengthening Mice: viable; shortened circadian period of behavior (confirmed using pharmacol	Cancer; neuro-degenerative disease (mutation, aberrant expression)	[7-9]

					ogical inhibition)		
TAO1	Thousand And One Kinases 1 (TAOK1)	31	Various cell types and tissues	Various signaling pathways (Hippo, DNA damage, IL17R); cell mitosis; regulator of apoptosis	Mice: viable	-	[10-11]
TrkA	Tropomyosin receptor kinase A; Neurotrophic receptor tyrosine kinase 1 (NTRK1)	33	Enriched in CNS	Neurological development (NGF receptor), B-cell development	Mice: die at 2-3 weeks of age due to defects in neurological development	Cancer (gene fusions); behavioral disorders (mutations)	[12-13]
Mer	Myeloid epithelial reproductive tyrosine kinase (MerTK)	43	Various cell types and tissues; enriched in hematopoietic cells, especially innate immune cells	Macrophage function, in particular efferocytosis, the immunologically silent clearance of apoptotic cells	Mice: viable; enhanced inflammatory response to endotoxin; accumulation of apoptotic cells; photoreceptor degeneration.	Inherited retinal dysfunction; cancer (aberrant expression), cancer-associated immunosuppression	[14-16]
TrkB	See Trk A; Neurotrophic receptor tyrosine kinase 2 (NTRK2)	46	Enriched in CNS, adipose tissue	Neurological development (BDNF receptor)	Mice: viable; sensory deficiencies	Cancer (gene fusions); obesity and behavioral disorders (mutations)	[13, 17-18]
PKA-Cbeta	protein kinase cAMP-activated catalytic	59	Enriched in CNS and hematopoi	Signal transduction in various	Mice: viable; no major neurologic	-	[19]

	subunit beta (PKACB)		etic stem cells	physiological processes	al phenotype		
Haspin	haploid germ cell-specific nuclear protein kinase; Histone H3 associated protein kinase (GSG2)	67	Ubiquitous	Mitosis	Cells: inhibition of mitosis Mice: viable; no obvious phenotype	-	20-23
Flt4	Fms related tyrosine kinase 4; Vascular endothelial growth factor receptor 3 (VEGFR3)	78	Enriched in lymphatic endotheliu m	Lymphangioge nesis	Mice: embryonal lethal	Cancer and metastasis (aberrant expression); Defects in lymphatic developme nt (deficiency)	24-26
CLK2	cdc-like kinase 2	79	Various cell types and tissues	Regulation of mRNA splicing		Neurodege nerative disorders (gene deficiencies); Cancer (over-expression)	27-31
KDR	Kinase insert domain receptor; vascular endothelial growth factor receptor 2 (VEGFR-2)	80	Various cell types and tissues	Angiogenesis	Mice: embryonal lethal	Cancer and metastasis (aberrant expression)	26
PKA-Calpha	protein kinase cAMP-activated catalytic subunit alpha (PKACA)	83	Ubiquitous; see PKACbeta				

CLK4	cdc-like kinase 4	83	Various cell types and tissues	Regulation of mRNA splicing			27, 31
STK16	Serine/threonine kinase 16	87	Various cell types and tissues				32
TAO3	Thousand And One Kinases 3 (TAOK3)	110	Various cell types and tissues	DNA damage response; positive regulator of TCR signaling	T-cells: hypo-responsiveness Mice: defect in B-cell development	Cancer (elevated expression in CSCs)	10, 33-35
IRAK4	Interleukin 1 receptor associated kinase 4	120	Various cell types; enriched in immune cells	Signaling pathways towards immune cell activation	Mice: immune deficiencies	Immune deficiency (mutation); autoimmune disorders, inflammation and cancer (overstimulation/expression)	36-38
CLK1	cdc2-like kinase 1	160	Various cell types and tissues	Regulation of mRNA splicing		Neurodegenerative disorders (gene deficiencies); Cancer (over-expression)	27, 31
TrkC	Neurotrophic receptor tyrosine kinase 3 (NTRK3)	170	Enriched in CNS	Neurological development (NT3)	Mice: die at 2-3 weeks of age due to defects in neurological development	Cancer (gene fusions)	13, 18
IRAK1	Interleukin 1 receptor	170	Various cell types; enriched in		Mice: immune	Immune deficiency (mutation);	37, 39-40

	associated kinase 1		immune cells		deficiencies	autoimmune disorders, inflammation and cancer (overstimulation/expression)	
GCK	Glucokinase	190	Various cell types and tissues	Glucose metabolism	Mice: decreased insulin secretory response to glucose	Diabetes mellitus (mutation)	[41]
Flt1	Fms related tyrosine kinase 1; vascular endothelial growth factor receptor 1 (VEGFR-1)	200	Various cell types and tissues	Angiogenesis	Mice: embryonal lethal	Cancer and metastasis (aberrant expression)	[26]
MRCKgamma	CDC42 binding protein kinase gamma (CDC42BPG)	150	Various cell types and tissues	Regulation of actin cytoskeletal reorganization			[42-43]
Fes	FES tyrosine kinase	300	Enriched in hematopoietic cells	Myeloid cell function	Mice: no prominent phenotype	Cancer: possible role in tumor-related angiogenesis and myeloid cell infiltrate	[44-45]
Met	MET proto-oncogene	350	Various cell types and tissues	Activation of several growth related signaling cascades	Mice: embryonal lethal	Cancer (mutation and over-expression)	[46]
Ltk	Leukocyte receptor tyrosine kinase	380	Various cell types and tissues	Neurogenesis; immune regulation	Mice: viable; behavioral phenotype	Cancer (high expression in leukemia)	[47]

FGFR1	Fibroblast growth factor receptor 1	380	Various cell types and tissues	Regulation of embryonic development, cell proliferation, differentiation and migration	Mice: embryonal lethal	Cancer (over-expression; mutation)	[48-49]
PTK5	Fyn related Src family tyrosine kinase (FRK)	410	Various cell types and tissues	Activation of several growth related signaling cascades	Mice: no prominent phenotype	Cancer (aberrant expression)	[50-51]
MELK	Maternal embryonic leucine zipper kinase	590	Various cell types and tissues, enriched in hematopoietic system	Involved in various processes such as cell cycle regulation, self-renewal of stem cells, apoptosis	Mice: no prominent phenotype	Cancer (elevated expression)	[52-53]

Table S5. Cellular kinase selectivity profiling of BAY-405 in KinomeScout ™ assay (Omicscouts)

Cellular kinase inhibition IC_{50} values were determined using the KinomeScout ™ profiler assay offered by OmicScouts (Germany)[54]. KinomeScout™ technology comprises chemical proteomics affinity and selectivity profiling in cell lysates. The cell lysate mixture used to profile BAY-405 was generated from Jurkat E6-1, COLO 205 MV-4-11 and SK-N-BE-(2) cells. Compound concentrations of BAY-405 applied for broad band kinase inhibitor competition experiment to assess dose-response curves were 0.001 µM, 0.003 µM, 0.01 µM, 0.03 µM, 0.1 µM, 0.3 µM, 1 µM, 3 µM and 30 µM. Cellular IC_{50}-unbound was calculated from the measured dose-response curve data by taking into account that only 0.9% of BAY-405 was available as unbound fraction in the cell lysate mixture. Kinases are listed in order of their BAY-405 IC_{50} as determined in the biochemical assay, in accordance with *Table 1.*

**BAY-405 cellular IC_{50}**		
**Kinase**	**IC_{50} (nM) measured**<br>n.d. not detected by MassSpec	**IC_{50} (nM) unbound calculated**<br>**$Fu_{BAY-405}$ = 0.9%**
**HPK1 (MAP4K1)**	**4427**	**40**
MAP4K3	4188	38
MAP4K5	> 30 µM	> 270
CK1epsilon	2168	38
TAO1	> 30 µM	> 270
TrkA	> 30 µM	> 270
Mer	> 30 µM	> 270
TrkB	n.d.	n.d.
PKACbeta	> 30 µM	> 270
Haspin	> 30 µM	> 270
Flt4	n.d.	n.d.
CLK2	> 30 µM	> 270
KDR	n.d.	n.d.
PKACalpha	> 30 µM	> 270
CLK4	> 30 µM	> 270
STK16	> 30 µM	> 270
TAO3	> 30 µM	> 270
IRAK4	> 30 µM	> 270
CLK1	> 30 µM	> 270
TrkC	n.d.	n.d.

IRAK1	> 30 µM	> 270
GCK / MAP4K2	> 30 µM	> 270
Flt1	n.d.	n.d.
MRCKgamma	> 30 µM	> 270
Fes	> 30 µM	> 270
Met	> 30 µM	> 270
Itk	> 30 µM	> 270
FGFR1	> 30 µM	> 270
PTK5	n.d.	n.d.
MELK	> 30 µM	> 270

Table S6. Inhibition of MAP4K1 kinase activity by BAY-405 in cell-based assays

Summary of BAY-405 biological activity in vitro as determined by means of HTRF-based SLP76 phosphorylation assay in different cell cultures as indicated and further described in the Methods section.

**Assay \ Cell system**	**Jurkat**	**Jurkat MAP4K1 KO**	**Primary Human PBMC**	**Mouse splenocytes WT**	**Mouse splenocytes MAP4K1 KI**
**pSer376-SLP76 IC_{50} (μM)**	0.8-1.5	>10	0.63	1.1	>10
**IFNγ ELISA EC_{50} (μM)**	-	-	0.212 (PGE2)<br>0.111 (TGFß)	0.194 (PGE2)<br>0.048 (TGFß)	-

Table S7. Pharmacokinetic Properties of BAY-405 after IV administration.

Unbound plasma concentrations and resulting PK parameters of BAY-405 after intravenous administration (bolus) of 1 mg/kg to female CD1 mice (n=3)

Plasma concentration [µg/L]				
Time	Animal No.			GeoMean
[h]	1	2	3	
0.033	160	149	240	179
0.083	148	167	225	177
0.25	184	178	223	194
0.5	165	318	159	162
1	139	119	167	140
2	73	83.1	90.9	82
4	21.4	51.3	28.7	31.6
7	5.3	32	7.73	11
24	1.05	<LLOQ	0.63	0.81

Selected PK parameters		
CL_{blood}	[L/h/kg]	2.0
V_{ss}	[L/kg]	6.9
$t_{1/2}$	[h]	4.0
AUC_{norm}	[kg·h/L]	0.51

Table S8. Crystallization data collection and refinement statistics

Values in brackets refer to the highest resolution shell

**Compound Number**	**BAY-405**
PDB ID	8PAR
**Data Collection and Processing**	
Wavelength [Å]	0.9184
Space group (no.)	$P2_12_12$ (18)
Unit cell parameters, *a, b*, *c* [Å]	76.72<br>100.21<br>39.50
Resolution limit [Å]	2.0 – 41.95<br>(2.0 – 2.12)
No. of reflections	150802 (23296)
No. of unique reflections	20999 (3287)
Multiplicity	7.2 (7.1)
$I/\sigma(I)$	14.22 (1.29)
R_{meas} [%]	11.3 (169.4)
CC (1/2)	99.9 (70.3)
Completeness [%]	98.6 (97.7)
**Refinement**	
R_{work}/R_{free} [%]	23.95 / 29.24
RMSD bond length [Å]	0.007
RMSD bond angles [deg]	1.519
Mean B value [Å^2]	56.1

Table S9. Exposure of BAY-405 after single oral administration

Total and unbound plasma concentrations and resulting PK parameters for BAY-405 following oral administration of 30 and 60 mg/kg to female Balb/c mice (single dose - vehicle Solutol/Ethanol/Water 40/10/50).

Unbound potency values were calculated as follows:

- pSLP76 IC50,u (1% FCS): 0.63 (value from Table S6) x 0.31 (fu-medium 1% FCS) = 0.195 uM
- hPBMC EC50,u (Xvivo-20 Medium): 0.111 (value from Table S6) x 0.28 (fu-XVivo-20 medium hPMBC) = 0.031 uM

Plasma concentration	Total (uM)		Unbound (nM)	
Time (h)	30 mg/kg (p.o.)	60 mg/kg (p.o.)	30 mg/kg (p.o.)	60 mg/kg (p.o.)
1	8.4	13	117	181
4	7.3	12	102	167
6	3.0	7.3	41.7	102
24	0.0074	0.041	0.103	0.57
AUC(0-tlast) (h·µM)	46	88	0.64	1.2
AUC(0-tlast) norm (h·kg/L)	0.81	0.77	0.011	0.011
Cmax (µM)	8.4	13	0.12	0.18
Cmax, norm (kg/L)	0.15	0.11	2.09E-03	1.5E-03

Binding Data for BAY-405 (**38**)		Unbound Potency for BAY-405 (**38**) [nM]	
Ppb fu mouse	1.39%	MAP4K1 binding competition IC_{50}	6
fu-medium (10% FCS)	4.3%	pSLP76 $IC_{50,u}$ (1% FCS)	195
fu-medium (1% FCS)	31%	hPBMC $EC_{50,u}$ (Xvivo-20 Medium)	31
fu-XVivo-20 medium (hPMBC)	28%		

Figure S2. Generation and validation of MAP4K1-deficient Jurkat T-cells and kinase-dead knock-in mice

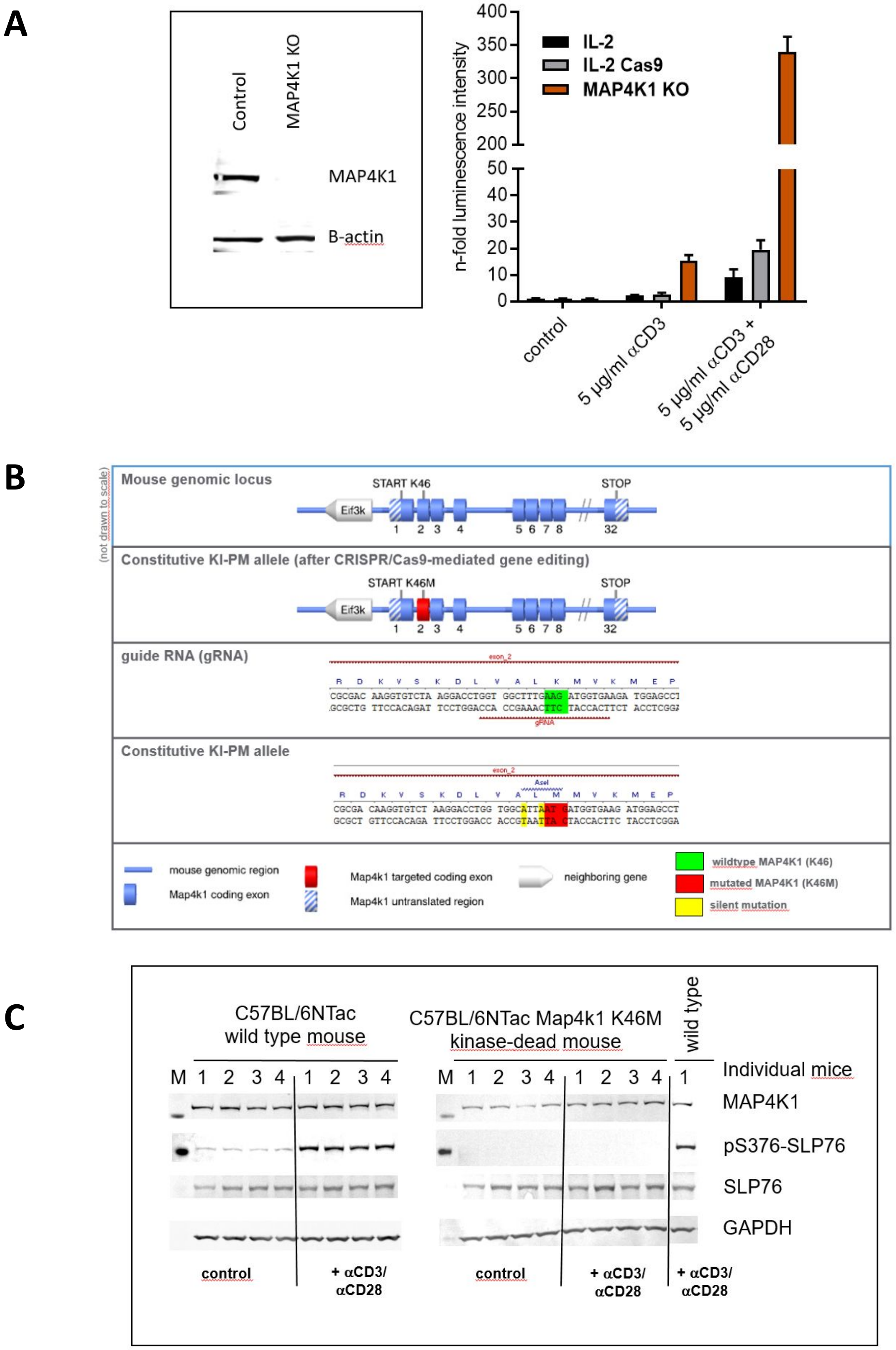

Legend to Figure S2

A. MAP4K1-deficient Jurkat T-REX IL2 NLUCP have been generated on the basis of stably expressing Cas9 in Jurkat T-REX IL2 NLUCP cells. Clonal line 3 completely lacks MAP4K1 protein expression as shown by Western Blot analysis. MAP4K1 knockout potentiated CD3/CD28-induced IL2 expression as detected by measuring IL2-Luc luciferase-activity **B.** MAP4K1 kinase-dead knock-in mouse: Schematic representation of constitutive knock-in of a K46M point mutation introduction in the *Map4k1* gene via CRISPR/Cas9-mediated gene editing (Taconic Biosciences GmbH, Germany) which was confirmed by target site PCR amplification and DNA sequence analysis of isolated genomic tail DNA (data not shown). **C.** The selected MAP4K1 K46M kinase-dead mouse strain (K46M) was further validated through immunoblotting of wild type and K46M splenocytes ex vivo stimulated with plate-bound CD3 and CD28 Abs. Protein expression of MAP4K1, SLP76 and pSer376-SLP76 was determined using respective antibodies. K46M splenocytes showed no phosphorylation of Ser376-SLP76 upon TCR stimulation, while still showing MAP4K1 expression.

Fig. S3. ***In vitro*** **pharmacology**

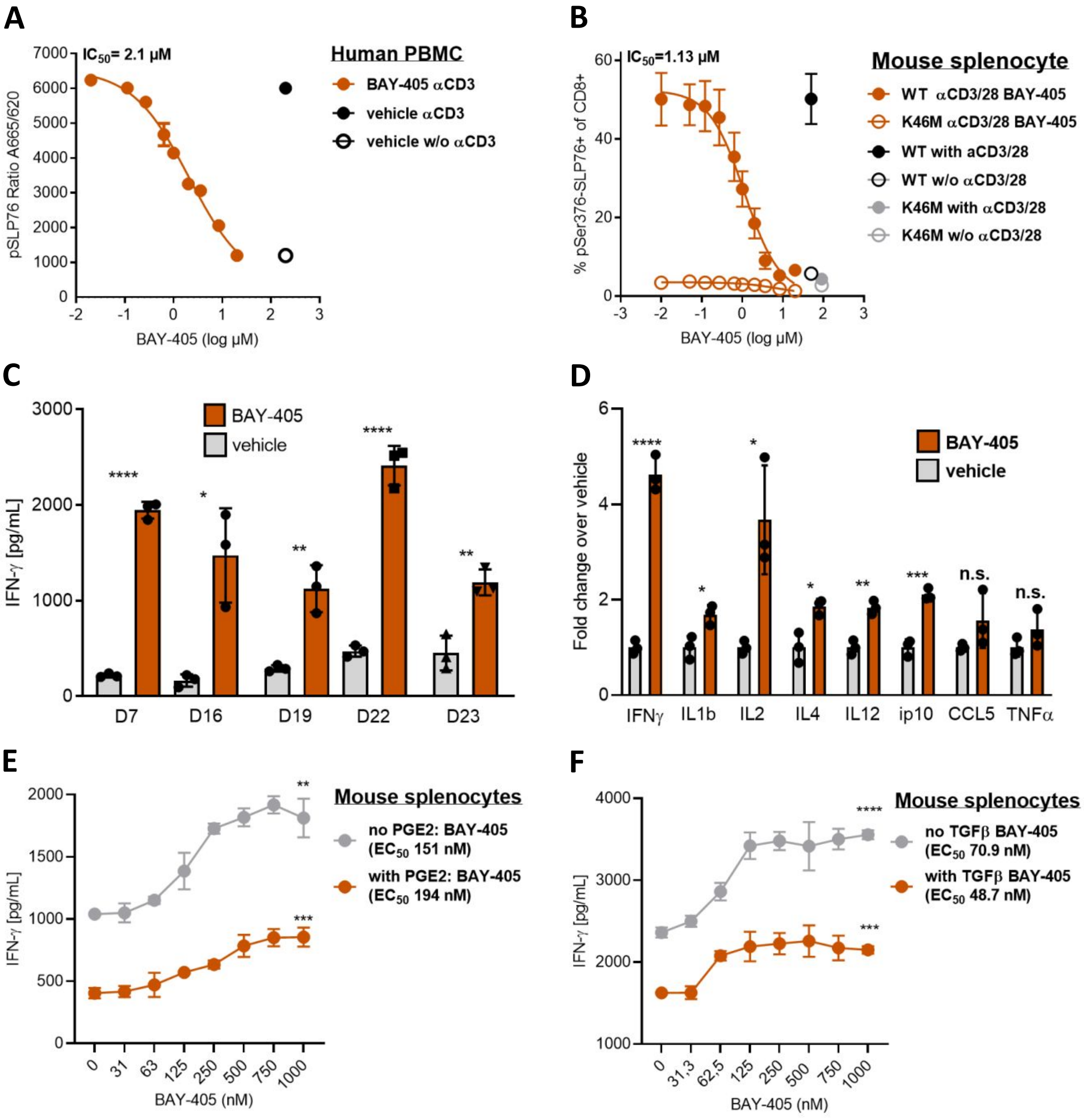

Legend to Fig. S3.

A. Analysis of the impact of BAY-405 on pSLP76 levels in anti-CD3 Ab stimulated primary human PBMC cultures. **B.** Measurement of the suppression of pSLP76 levels by BAY-405 in CD3/CD28-Ab stimulated primary mouse splenocyte cultures from wild type mice and MAP4K1 kinase-dead knock-in mice, using an anti-mouse pSer376-SLP76 Ab by means of FACS methodology. **C.** Enhancement of T-cell reactivity by 1000 nM BAY-405 human PBMC cultures from different donors stimulated with 30 ng/ml anti-CD3 Ab in the presence of 1µM PGE2. Secreted IFNɣ was analysed after 24 hrs by means of ELISA. P-values: D7 < 0.0001; D16 = 0.0104; D19 = 0,0049; D22 < 0.0001; D23 = 0.0043. **D.** Detection of T-cell stimulation by BAY-405 as in A on the basis of analysis of additional effector cytokines in the culture media. P-values: INFγ < 0.0001; IL1b = 0.0185; IL2 = 0.157; IL4 = 0.137; IL12 = 0.0021; CXCL10 = 0.000.8. **E.** Dose-dependent enhancement of T-cell reactivity in mouse splenocyte cultures stimulated with 300ng/ml anti-CD3 Ab in the absence or presence of 1µM PGE2, as determined on the basis of secreted IFNɣ after 24 hours. 750 nM BAY-405 no PGE2 vs vehicle no PGE2 p<0.0001; 1000 nM BAY-405 no PGE2 vs vehicle no PGE2 p=0.0007 (student t-test). **F.** Experiment as in E, but with 10 ng/ml TGF-β as inhibitory substance. 1000 nM BAY-405/with TGFβ vs vehicle/with TGFβ p=0.0002. 1000 nM BAY-405/no TGFβ vs vehicle/no TGFβ p<0.0001 (student t-test).

Fig. S4. ***In vitro and in vivo*** **anti-tumor T-cell reactivity**

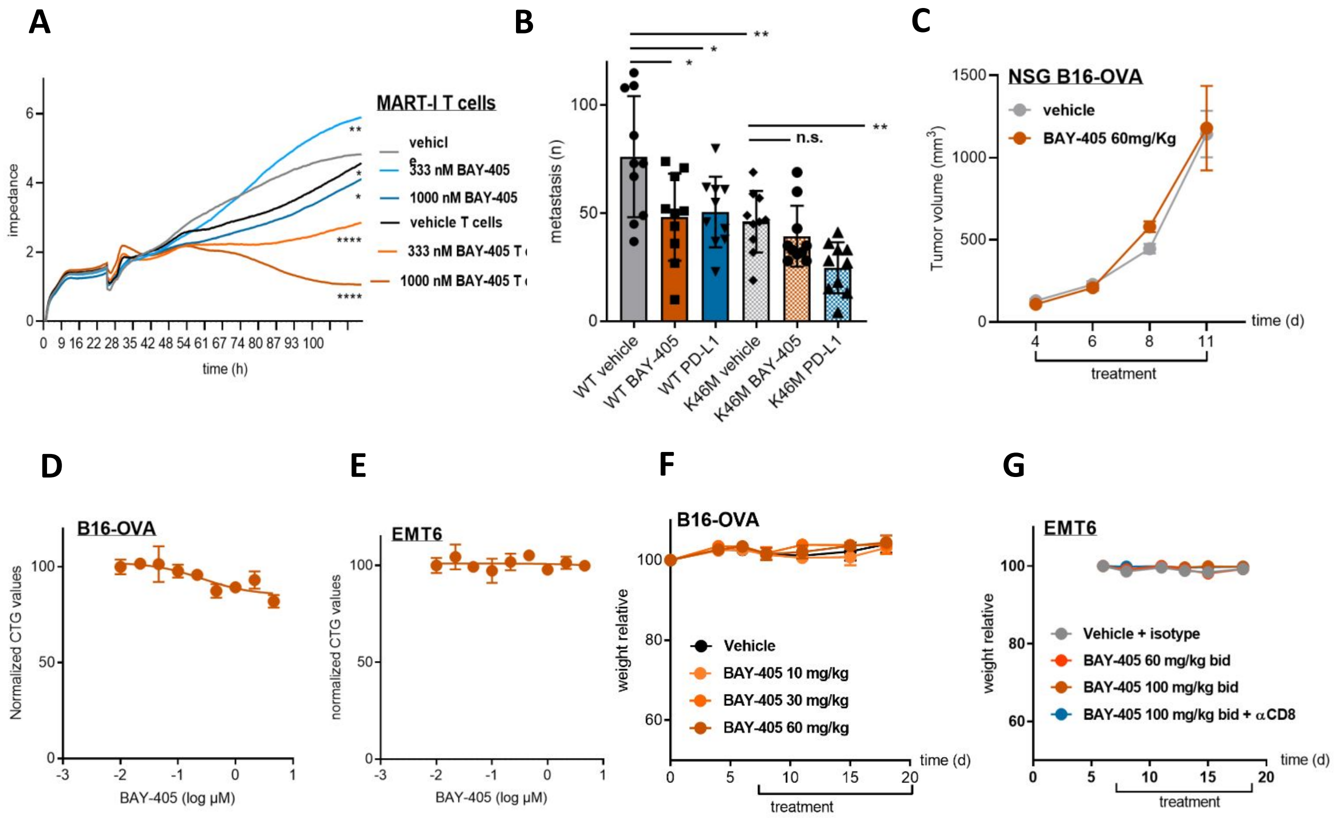

Legend to Fig. S4.

A. xCelligence^{TM} real time analysis of the cytotoxicity of BAY-405 towards the HLA-A*0201, MART-1 positive human melanoma cell line COLO800 in the presence/absence of MART-1 specific T cells. **B.** Impact of 60 mg/kg BAY-405 on B16 melanoma tumor outgrowth in lungs after i.v. injection of 2x10E4 B16-OVA cells. The numbers of lung tumor nodes were analysed at day 14 after tumor grafting. The experiment was performed in wild type B6 mice as well as in MAP4K1 kinase dead knock in mice, as indicated. Furthermore, the anti-tumor efficacy of BAY-405 was benchmarked against that of anti-PD-L1 blocking Ab at 10 mg/kg twice weekly. Ten mice were used for each condition. P-values: WT vehicle vs WT BAY-405 = 0.0193; WT vehicle vs WT PD-L1 = 0.0224; WT vehicle vs K46M vehicle= 0.0071; K46M vehicle vs K46M PD-L1 = 0.0018. **C.** Lack of anti-tumor impact of BAY-405 at 60 mg/kg in immunodeficient NSG mice challenged subcutaneously with 2x10E5 B16-OVA tumor cells. **D & E**. BAY-405 was added at indicated oncentrations to actively proliferating in vitro cultures of respecytively B16-OVA and EMT6 tumor cells. Cell viability was measured by CellTiterGlow assay at 72h timepoint. Each cell line proliferation assay was tested three times with similar outcome. **F & G.** Monitoring of mouse total body weight in the context of BAY-405 at indicated levels, demonstrating no detectable weight loss. **F.** Body weight data of mice from B16-OVA tumor growth experiment treated with indicated doses of BAY-405 (*corresponding to Fig. 5D*), 10 mice each group. **G.** Body weight data of mice from EMT6 tumor growth experiment treated with indicated doses of BAY-405 (*corresponding to Fig. 5E*), 10 mice each group.

Table S10. Exposure of BAY-405 in EMT6 efficacy model

Total and unbound plasma concentrations for BAY-405 at day 1 and 13 following repeated twice daily oral dosing of 60 and 100 mg/kg to female Balb/c mice (vehicle: Solutol/Ethanol/Water 40/10/50) from EMT6 efficacy model presented in **Figure 5E**. See Table S9 for calculation unbound potency values.

Plasma concentration	Total (uM)				Unbound (nM)			
Time (h)	60 mg/kg Day 1	60 mg/kg Day 13	100 mg/kg Day 1	100 mg/kg Day 13	60 mg/kg Day 1	60 mg/kg Day 13	100 mg/kg Day 1	100 mg/kg Day 13
1	6.8	3.1	8.0	3.9	95.1	42.8	111	54.7
4	7.0	3.5	6.6	5.1	98.0	49.3	92.1	71.5
6	3.9	1.6	5.6	2.8	54.6	21.7	78.3	38.6
24	n.s	0.023	n.s.	0.077	n/a	0.319	n/a	1.07

n.s. = not sampled

Binding Data for BAY-405 (**38**)		Unbound Potency for BAY-405 (**38**) [nM]	
Ppb fu mouse	1.39%	MAP4K1 binding competition IC_{50}	6
fu-medium (10% FCS)	4.3%	pSLP76 $IC_{50,u}$ (1% FCS)	195
fu-medium (1% FCS)	31%	hPBMC $EC_{50,u}$ (Xvivo-20 Medium)	31
fu XVivo-20 medium (hPMBC)	28%		

Figure S5. Exposure of BAY-405 in EMT6 (Figure 5E) efficacy model at day 1 and 13 after oral twice daily dosing.

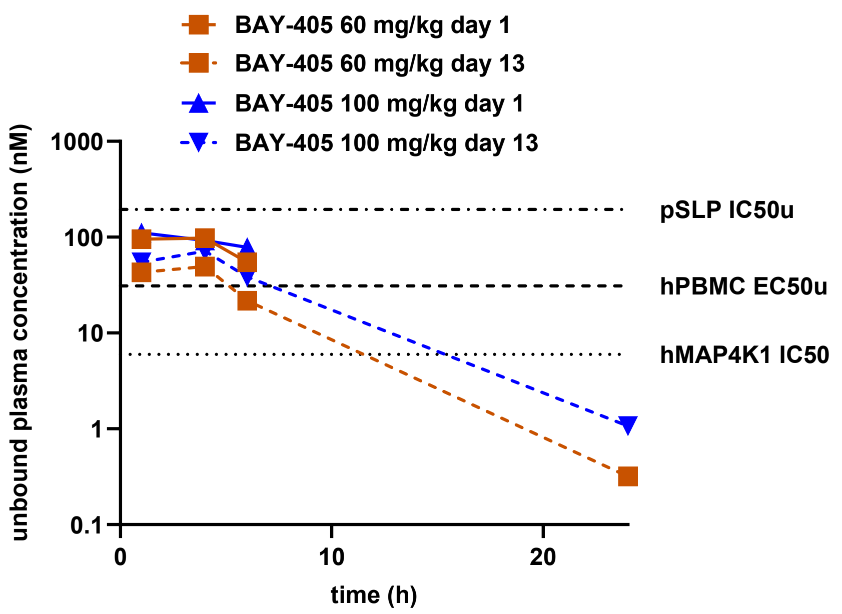

Experimental Methods

ROCK2 assay

ROCK2 inhibitory activity of compounds of the present invention was quantified employing the ROCK-II assay as described in the following 5 paragraphs. In essence, the enzyme activity is measured by quantification of the adenosine-diphosphate (ADP), which is generated as a co-product of the enzyme reaction, via the “ADP-Glo™ Kinase Assay” kit from the company Promega. This detection system works as follows: In a first step the adenosine-tri-phosphate (ATP) not consumed in the kinase reaction is quantitatively converted to cAMP employing an adenylate cyclase (“ADP-Glo-reagent”), then the adenylate cyclase is stopped and the ADP generated in the kinase reaction converted to ATP which generates in a luciferase-based reaction a glow-luminescence signal (“Kinase Detection Reagent”).

Recombinant N-terminal His6-tagged human ROCK-II (amino acids 11-552), expressed by baculovirus infected SF21 insect cells and purified via Ni2+-NTAagarose affinity chromatography, was purchased from Eurofins (product no. 14-451-K) and used as enzyme. As substrate for the kinase reaction the biotinylated peptide biotin-Ahx-KEAKEKRQEQIAKRRRLSSLRASTSKSGGSQK (C-terminus in amide form) was used which can be purchased e.g. from the company Biosyntan (Berlin-Buch, Germany).

For the assay 50 nl of a 100fold concentrated solution of the test compound in DMSO was pipetted into a white 1536well microtiter plate (Greiner Bio-One, Frickenhausen, Germany), 2 µl of a solution of ROCK-II in aqueous assay buffer [50 mM TRIS/HCl pH 7.5, 10 mM MgCl2, 0.1 mM EGTA, 0.001 % (w/v) bovine serum albumin] were added and the mixture was incubated for 15 min at 22°C to allow pre-binding of the test compounds to the enzyme before the start of the kinase reaction. Then the kinase reaction was started by the addition of 3 µl of a solution of ATP (16.7 µM => final conc. in the 5 µl assay volume is 10 µM) and peptide substrate (16.7 µM => final conc. in the 5 µl assay volume is 10 µM) in assay buffer and the resulting mixture was incubated for a reaction time of 30 min at 22°C. The concentration of ROCK-II was a djusted depending of the activity of the enzyme lot and was chosen appropriate to have the assay in the linear range, a typical concentration is about 5 nM. The reaction was stopped by the addition of 2.5 µl of “ADP-Glo-reagent” (1:1.5fold diluted) and the resulting mixture was incubated at 22°C for 1 h to convert the ATP not consumed in the kinase reaction completely to cAMP. Subsequently 2.5 µl of the “kinase detection reagent” (1.2fold more concentrated than recommended by the producer) were added, the resulting mixture was incubated at 22°C for 1 h and then the luminescence measured with a suitable measurement instrument (e.g. Viewlux™ from Perkin-Elmer). The amount of emitted light was taken as a measure for the amount of ADP generated and thereby for the activity of the ROCK-II.

The data were normalised (enzyme reaction without inhibitor = 0 % inhibition, all other assay components but no enzyme = 100 % inhibition). Usually the test 15 compounds were tested on the same microtiterplate in 11 different concentrations in the range of 20 µM to 0.1 nM (20 µM, 5.7 µM, 1.6 µM, 0.47 µM, 0.13 µM, 38 nM, 11 nM, 3.1 nM, 0.9 nM, 0.25 nM and 0.07 nM, the dilution series prepared separately before the assay on the level of the 100fold concentrated solutions in DMSO by serial dilutions, exact concentrations may 20 vary depending pipettors used) in duplicate values for each concentration and IC_{50} values were calculated using Genedata Screener™ software.

Metabolic Stability in Rat Hepatocytes

Test compounds were incubated in freshly isolated rat liver cells suspended in Williams' medium E to a final concentration of 1 µM. During incubation, the hepatocyte suspensions were continuously shaken and aliquots were taken after 2, 8, 16, 30, 45, and 90 min. Samples were frozen, and subsequently centrifuged. The supernatant was analyzed with LC-MS/MS detection to determine the rate of degradation. This provided the in vitro intrinsic clearance according to Lau2 based on the average liver cell count per gram liver and specific liver weight. Predicted in vivo clearance (CL_H) was calculated using the 'well-stirred' liver model based on rat or human liver blood flow (Q_H). Maximum bioavailability (Fmax [%]) was calculated from the predicted in vivo clearance and liver blood flow according to the equation:

$$F_{max} [\%] = 1 - CL_H/Q_H$$

Cytochrome P450 (CYP) Inhibition Assay

Human liver microsomes (pooled, >30 male and female donors) were incubated with individual CYP isoform selective standard probes (phenacetin, amodiaquine, diclofenac, dextromethorphan, midazolam) in the absence or presence of increasing concentrations of the test compound to compare the extent of formation of the respective metabolite. Incubation conditions (protein and substrate concentration, incubation time) were optimized with regard to linearity and metabolite turnover. Incubation medium consisted of 50 mM potassium phosphate buffer (pH 7.4) containing 1 mM EDTA, NADPH regenerating system [1 mM NADP, 5 mM glucose-6-phosphate, glucose-6-phosphate dehydrogenase (1.5 U/mL)]. Sequential dilutions and incubations were performed on a Genesis Workstation (Tecan, Crailsheim, Germany) in 96-well plates at 37 °C. A final incubation volume of 200 µL was used. Reactions were stopped S6 by addition of MeCN (100 µL) containing the respective internal standard. Precipitated proteins were removed by centrifugation of the well plate, supernatants were combined, and analyses were performed by LC-MS/MS. LC-MS/MS quantification of the metabolites paracetamol (CYP1A2), desethylamodiaquine

(CYP2C8), 4-hydroxydiclofenac (CYP2C9), dextrorphan (CYP2D6), and 1-hydroxymidazolam (CYP3A4) was performed with a PE SCIEX API 3000 LC/MS/MS system (Applied Biosystems/MDS Sciex, Concord, Ontario, Canada). A sigmoidshaped curve was fitted to the data, and the enzyme inhibition parameter IC_{50} was calculated using a nonlinear least-squares regression analysis of the plot of percent control activity versus concentration of the test inhibitor.

Pegnane xenobiotic receptor (PXR) Nuclear Receptor Activation

DPX2 cells (hepatoma cell line, stably cotransfected with a vector for human PXR and a luciferase reporter gene under the control of two human CYP3A4 promotors; Puracyp, Carlsbad, CA, USA) were cultivated according to the manufacturer's instructions with the following modifications: Cells were seeded in a 384-well plate and cultivated at 37 °C/5% CO2 in humidified air. 24 h prior readout, the cells were treated with compound in a 10-point serial dilution of 1:3 starting at the highest test concentration of 50 μM and ending at 2 nM. Rifampicin was incubated in the same manner as a positive control. In addition, for normalization of the luminescence signal, cells were incubated with rifampicin at a concentration of 16.7 μM corresponding to 100% activation, as well as with DMSO for background luminescence corresponding to 0% activation (n = 32 wells each). Cells were lysed and incubated with the luciferase substrate ONE-Glo Reagent (Promega, Madison, WI, USA) according to the manufacturer's instructions, and the luminescence signal was detected in a plate reader. A concentration-dependent increase of the luciferase activity above 10% of the rifampicin control was classified as PXR transactivation. The concentration at 10% of the rifampicin control was recorded as the minimal effective concentration (MEC).

CYP 3A4 Induction Assay

Human hepatocytes are seeded at a density of ∼40 000 cells/96 well in a collagen sandwich and cultured for 1 day before compound treatment. The cells are treated with a 1:3 serial dilution of 8 concentrations for two consecutive days with media change every day. After 48 h of compound treatment, the cells are lysed and mRNA is prepared by the state-of-the-art magnetic beads technique. Briefly, approximately 24 h after the last treatment, hepatocytes were harvested for mRNA isolation. Thus, cell culture medium was removed from each well, cells were washed with 150 μL supplement free cell culture medium prior to cell lysis, cells were lysed with 100 μL lysis buffer containing proteinase K (50 μg/μL; final concentration in well 30 ng/μL), and final cell lysates were stored at −80 °C. mRNA was isolated using the Dynabeads mRNA Direct Kit (Life Technologies); 150 μL of each cell lysate was mixed with 100 μg magnetic beads, incubated for a few minutes, then the supernatant was removed and beads were washed twice with Washing Buffer

A and twice with Washing Buffer B with 200 and 100 μL per well, respectively. Single-stranded cDNA was prepared from mRNA with the High Capacity RNA to cDNA Kit (Life Technologies). RT Master Mix (20 μL) was added to each well and transcribed for 60 min at 37 °C using the Gene Amp PCR System 9700 thermocycling program (Biometra). The RT Master Mix is composed of 10× RT buffer, 25× deoxyNTPs, 10× Random hexamers, RNase Inhibitor (20 U/μL), MultiScribe reverse transcriptase (50 U/μL), and RNase-free water. The prepared cDNA samples were stored at −80 °C prior to analysis by quantitative real-time polymerase chain reaction (qRT-PCR). Quantitative RT-PCR was carried out on a QuantStudio7 Flex PCR system (Applied Biosystems) according to manufacturer's protocol. A primer mix was prepared for each gene expression assay. A typical primer mix contained TaqMan Fast Advanced Master Mix (1×), Gene Expression Assay (1×, 900 nM forward and reverse primers), and RNase-free water and was added to the cDNA. The relative quantity of the target cDNA compared to that of the control cDNA (Actin, Tubulin) was determined by the ΔΔCt method. Relative quantification measures the change in mRNA expression in a test sample relative to that in a control sample (e.g., vehicle-treated). In summary, CYP induction is calculated based on the ΔΔCt method and expressed as fold induction over vehicle-treated control.

Caco-2 Permeation Assay

Caco-2 cells were seeded at a density of 4.5 × 104 cells/well on 24-well insert plates and grown for 15 d in DMEM. Cells were maintained at 37 °C in a humidified atmosphere. Medium was changed every 2–3 d. Before the permeation assay was run, the culture medium was replaced by transport medium (FCS-free HEPES carbonate transport buffer, pH 7.2). For the assessment S7 of monolayer integrity, the transepithelial electrical resistance was measured. Test compounds were predissolved in DMSO and added either to the apical (A) or basolateral (B) compartment at a final concentration of 2 μM. Before and after incubation for 2 h, samples were taken from both compartments and, after precipitation with MeOH, analyzed by LC-MS/MS. The apparent permeability coefficient (Papp) was calculated for the apical to basolateral (A→B) and the basolateral to apical (B→A) direction. The efflux ratio basolateral to apical was calculated by dividing the Papp B–A value by the Papp A–B value. In addition, the compound recovery was calculated, and assay control reference compounds were analyzed in parallel.

Estimation of Plasma Protein Binding by Equilibrium Dialysis

Binding of test compounds to plasma proteins was measured by equilibrium dialysis in a 96- well format using HTdialysis equipment [55]. A semipermeable membrane separated the plasma and buffer side (50 mM phosphate buffer), each filled with 150 μL. Test compound, added to the plasma side at a concentration

of 3 µM, binds to plasma proteins. Only the unbound fraction of the test compound can pass the membrane and distributes between both sides until equilibrium is reached, which is usually the case within the incubation time of 6–8 h at 37 °C. Compound concentration of the plasma and the buffer side was measured by LC-MS/MS analysis. For this, both sides were diluted with buffer and plasma to achieve the same matrix (10% plasma) and subsequently precipitated with MeOH. From the quotient of buffer and plasma concentration, the unbound fraction was calculated. Stability and recovery controls were included. Additionally, the test compound was dialyzed in buffer against buffer to estimate nonspecific binding to equipment and/or membrane and to ensure the establishment of equilibrium.

In Vivo Pharmacokinetics in Rats

All animal experiments were conducted in accordance with the German Animal Welfare Law and were approved by local authorities. For in vivo pharmacokinetic experiments, test compounds were administered to male Wistar rats intravenously and per oral gavage at doses of 0.3–5 mg/kg formulated as solutions using solubilizers such as PEG400 in well-tolerated amounts. Depending on the expected half-life, samples were taken at 7–10 time points after dosing. Blood was collected into lithium heparin tubes and centrifuged at 2000g for at least 5 min. An aliquot of 100 µL from the supernatant (plasma) was taken and precipitated by the S8 addition of cold MeOH. Samples were frozen overnight and subsequently thawed and centrifuged at 3000g for at least 15 min. Aliquots of the supernatants were analyzed with LCMS/MS detection. Pharmacokinetic parameters [including $AUC_{(0\text{–}t\ last)}$ and $AUC_{(0\text{–}infinity)}$, both in kg·h/L] were calculated by noncompartmental analysis using Phoenix WinNonlin software [Certara L.P. (Pharsight), St. Louis, MO, USA].

Synthesis of Compounds 1-38

Commercially available reagents and anhydrous solvents were used as supplied, without further purification. All air- and moisture-sensitive reactions were carried under an inert atmosphere of argon. Reactions were monitored by TLC and UPLC analysis with a Waters Acquity UPLC MS Single Quad system. Flash chromatography was carried out using a Biotage Isolera One system with 200−400 nm variable detector. Preparative HPLC was carried out with a Waters AutoPurification MS Single Quad system; column: Waters XBridge C18 5 µm, 100 × 30 mm; basic conditions: eluent A: H2O + 0.2 vol % aq NH3 (32%), eluent B: MeCN; gradient: 0−0.5 min 5% B, flow: 25 mL/min; 0.51−5.50 min 10−100% B, flow: 70 mL/min; 5.51−6.5 min 100% B, flow: 70 mL/min; acidic conditions: eluent A: H2O + 0.1 vol % formic acid (99%), eluent B: MeCN; gradient: 0−0.5 min 5% B, flow: 25 mL/min; 0.51−5.50 min 10− 100% B, flow: 70 mL/min; 5.51−6.5 min 100% B, flow: 70 mL/min; temperature: 25 °C; DAD scan: 210−400 nm.

NMR spectra were recorded at ambient temperature (22 ± 1 °C), unless otherwise noted, on Bruker AVANCE III HD spectrometers. ^{1}H NMR spectra were obtained at 300, 400, 500, or 600 MHz, and referenced to the residual solvent signal (2.50 ppm for [D6]DMSO). ^{13}C NMR spectra were obtained at 125 MHz and also referenced to the residual solvent signal (39.52 ppm for [D6]DMSO). ^{1}H NMR data are reported as follows: chemical shift (δ) in ppm, multiplicity (s = singlet, d = doublet, t = triplet, q = quartet, br = broad, and m = multiplet) and integration.

Low-resolution mass spectra (electrospray ionization) were obtained via HPLC−MS (ESI) using a Waters Acquity UPLC system equipped with an SQ 3100 Mass Detector (detailed experimental methods are provided in the LC-MS methods section). Unless otherwise explicitly stated, the purity of all target compounds was at least 95%, as determined by ^{1}H NMR spectroscopy and UPLC analysis. Compound names were generated using ICS software.

Analytical LCMS methods

Method 1:

Instrument: Waters Acquity UPLC-MS SQD 3001; Column: Acquity UPLC BEH C18 1.7 µm, 50x2.1 mm; Eluent A: water + 0.2 vol % ammonia, Eluent B: acetonitrile; Gradient: 0-1.6 min 1-99% B, 1.6-2.0 min 99% B; Flow rate: 0.8 mL/min; Temperature: 60 °C; Injection: 2 µL; DAD scan: 210-400 nm; ELSD.

Method 2:

Instrument: Waters Acquity UPLC-MS SQD 3001; Column: Acquity UPLC BEH C18 1.7 µm, 50x2.1 mm; Eluent A: water + 0.1 vol % formic acid , Eluent B: acetonitrile; Gradient: 0-1.6 min 1-99% B, 1.6-2.0 min 99% B; Flow rate: 0.8 mL/min; Temperature: 60 °C; Injection: 2 µL; DAD scan: 210-400 nm.

Method 3:

Instrument: Agilent 1290 UPLCMS 6230 TOF; column: BEH C 18 1.7 µm, 50x2.1mm; Eluent A: water + 0.05 % formic acid (99%); Eluent B: acetonitrile + 0.05 % formic acid (99%); gradient: 0-1.7 2-90% B, 1.7-2.0 90% B; flow 1.2 ml/min; temperature: 60°C; DAD scan: 190-400 nm.

Method 4:

Instrument: Waters Acquity UPLCMS SingleQuad; column: Acquity UPLC BEH C18 1.7 µm, 50x2.1mm; Eluent A: water + 0.2 vol-% ammonia (32%), Eluent B: acetonitrile; Gradient: 0-1.6 min 1-99% B, 1.6-2.0 min 99% B; Flow 0.8 ml/min; Temperature: 60 °C; DAD scan: 210-400 nm

Method 5:

Instrument: Agilent 1200\G6110A; column: Kinetex@ 5um EVO C18 30*2.1mm; mobile phase A: 0.0375% TFA in water (v/v), mobile phase B: 0.01875% TFA in Acetonitrile (v/v); gradient: 0.01 min 5% B → 0.80 min 95% B → 1.20 min 95% B → 1.21 min 5% B → 1.5 min 5% B; flow rate: 1.5 mL/min; oven temperature: 50 °C; UV detection: DAD (220&254nm).

Method 6:

Instrument: SHIMADZU LCMS-2020; column: Kinetex EVO C18 2.1*30 mm,5um; mobile phase A: 0.0375% TFA in water (v/v), mobile phase B: 0.01875% TFA in Acetonitrile (v/v); gradient: 0.0 min 30% B → 3.0 min 90% B → 3.50 min 90% B → 3.51 min 30% B → 4.0 min 30% B; flow rate: 0.8 mL/min; oven temperature: 50 °C; UV detection: DAD (220&254nm).

Method 7:

MS instrument type: Agilent 1200 LC/G1956A MSD; HPLC instrument type: Agilent ChemStation Rev.B.04.03; column: Kinetex EVO C18 2.1X30mm,5um; mobile phase A: 0.0375% TFA in Water (v/v), mobile phase B: 0.01875% TFA in Acetonitrile (v/v); gradient: 0.01 min 5% B → 0.80 min 95% B → 1.2 min 95% B → 1.21 min 5% B → 1.5 min 5% B; flow rate: 1.5 mL/min; oven temperature: 50 °C; UV detection: 220 nm & 254 nm.

Method 8:

MS instrument type: SHIMADZU LCMS-2020; HPLC instrument type: LabSolutions Version 5.72; column: Chromolith@Flash RP-18E 25-2 MM; mobile phase A: 0.0375% TFA in water (v/v), mobile phase B: 0.01875% TFA in Acetonitrile (v/v); gradient: 0.00 min 0% B → 0.80 min 60% B → 1.20 min 60% B → 1.21 min 0% B → 1.5 min 0% B; flow rate: 1.5 mL/min; oven temperature: 50 °C; UV detection: 220 nm & 254 nm.

Method 9:

Instrument: SHIMADZU LCMS-2020; column: Kinetex EVO C18 30*2.1mm,5um; mobile phase A: 0.0375% TFA in water (v/v), mobile phase B: 0.01875% TFA in Acetonitrile (v/v); gradient: 0.0 min 0% B → 0.80 min 60% B → 1.20 min 60% B → 1.21 min 0% B → 1.55 min 0% B; flow rate: 1.5 mL/min; oven temperature: 50 °C; UV detection: DAD (220&254nm).

Method 10:

Instrument: Agilent 1100\G1956A; column: Kinetex@ 5um EVO C18 30*2.1mm; mobile phase A: 0.0375% TFA in water (v/v), mobile phase B: 0.01875% TFA in Acetonitrile (v/v); gradient: 0.0 min 5% B → 0.80 min 95% B → 1.20 min 95% B → 1.21 min 5% B → 1.50 min 5% B; flow rate: 1.5 mL/min; oven temperature: 50 °C; UV detection: DAD (220&254nm)

N-(3,5-difluoro-4-{[3-(trifluoromethyl)-1H-pyrrolo[2,3-b]pyridin-4-yl]oxy}phenyl)pyrimidine-2,4-diamine (S1)

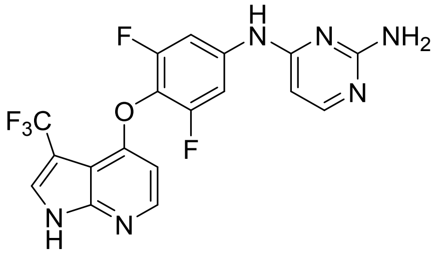

3,5-Difluoro-4-{[3-(trifluoromethyl)-1H-pyrrolo[2,3-b]pyridin-4-yl]oxy}aniline (1.50 g, 4.56 mmol, see Chem.Med.Chem. 2008, 3, 1893-1904) was dissolved in H_2O (15 mL) and EtOH (15 mL). To this solution, 4-chloropyrimidin-2-amine (590 mg, 4.56 mmol) and aq. HCl solution (36%, 0.58 mL) were added and the resulting stirring mixture was heated at reflux for 3 h. The reaction mixture was cooled down to room temperature and H_2O (50 mL) was added and stirred for 10 mins at room temperature. The resulting mixture was then neutralized using sat. aq. $NaHCO_3$ solution. The solid was formed and dissolved using ethyl acetate and washed with sat. aq. NaCl solution. The separated organic phase was dried ($MgSO_4$) and evaporated under reduced pressure to give

a crude material. The crude material was then purified by column chromatography (Hexane/ethyl acetate; 100 % → 0% and then 100% ethyl acetate followed by 100% MeOH) to give the desired product after evaporation of collected fractions. The resulting material was triturated with CH2Cl2 (50 mL) to give the title compound (680 mg, 35% yield) as a white solid.

Method 2, UPLC-MS (ESI+): t_R = 0.87 min; *m/z* calcd for $C_{18}H_{12}F_5N_6O$ $[M + H]^+$: 423.1; found: 423.3

^{1}H NMR (400 MHz, DMSO-d_6, 22ºC): δ = 12.63 (br s, 1H), 9.6 (br s, 1H), 8.21- 8.23 (m, 1H), 8.10 – 8.11 (m, 1H), 7.89 – 7.90 (m, 1H), 7.77 – 7-80 (m, 2H), 6.48 – 6.50 (m, 3H), 6.02 (d, *J* = 7.4 Hz, 1H).

<u>Compound 1</u>N-(3,5-difluoro-4-{[3-(trifluoromethyl)-1H-pyrrolo[2,3-b]pyridin-4-yl]oxy}phenyl)pyrimidine-2,4-diamine; hydrogen chloride (1/1)

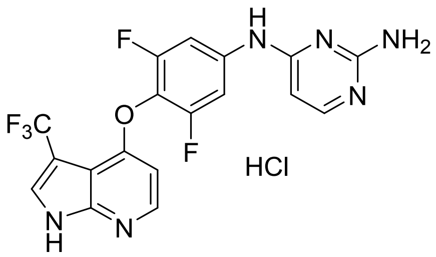

N-(3,5-difluoro-4-{[3-(trifluoromethyl)-1H-pyrrolo[2,3-b]pyridin-4-yl]oxy}phenyl)pyrimidine-2,4-diamine (680 mg, 1.61 mmol, **S1**) was dissolved in dioxane (15 mL) and 4M HCl in dioxane (0.48 mL) was added and the resulting mixture stirred for 18 h at room temperature. The reaction mixture was evaporated under reduced pressure and dried at 80°C for 5 h and then at 90°C for 5 h using an oil pump to give the title compound (653 mg, 85% yield).

Method 3, LC-MS (ESI+): t_R = 0.72 min; *m/z* calcd for $C_{18}H_{12}F_5N_6O$ $[M + H]^+$: 423.1; found: 423.1

^{1}H NMR (400 MHz, DMSO-d_6, 22ºC): δ = 12.69 (br s, 1H), 12.48 (br. s, 1H), 11.27 (br s, 1H), 8.24 (d, *J* = 5.6 Hz, 1H), 8.14-8.16 (m, 1H), 7.88-7.95 (m, 3H), 6.52 (d, *J* = 5.6 Hz, 1H), 6.44 (d, *J* = 7.4 Hz, 1H), 3.97 ppm (br s, 3H).

phenyl (3,5-difluoro-4-{[3-(trifluoromethyl)-1-{[2-(trimethylsilyl)ethoxy]methyl}-1H-pyrrolo[2,3-b]pyridin-4-yl]oxy}phenyl)carbamate (S2)

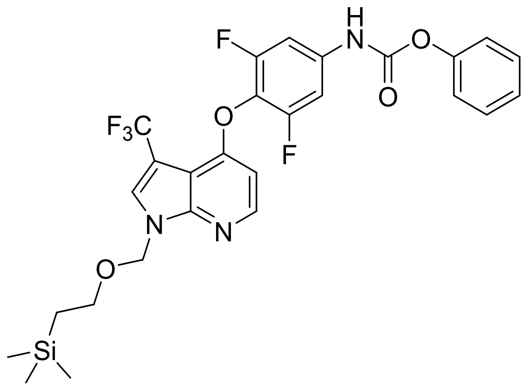

To solution of 3,5-difluoro-4-{[3-(trifluoromethyl)-1-{[2-(trimethylsilyl)ethoxy]methyl}-1H-pyrrolo[2,3-b]pyridin-4-yl]oxy}aniline (4.00 g, 8.71 mmol), in pyridine (4.0 mL) and THF (60 mL) was slowly added at 0°C phenyl carbonochloridate (1.2 mL, 9.6 mmol)[56]. After stirring this mixture 5 minutes at 0°C and then 30 minutes at room temperature ethyl acetate was added. This organic phase was washed with 1N hydrochloric acid (75mL), water, concentrated aqueous sodium bicarbonate, brine, dried over sodium sulfate, filtered and concentrated to dryness. The resulting residue was purified via a Biotage chromatography system (100g snap KP-Sil column, hexane / 0 – 100% ethyl acetate, then ethyl acetate / 0 – 75% methanol) to obtain 4.53 g (92 % purity, 83 % yield) of the desired title compound.

Method 2, UPLC-MS (ESI+): t_R = 1.66 min; *m/z* calcd for $C_{27}H_{27}F_5N_3O_4Si$ $[M+H]^+$: 580.2; found: 580.3

^{1}H-NMR (400 MHz, DMSO-d6) δ [ppm]: -0.10 (s, 9H), 0.79 - 0.86 (m, 2H), 3.54 - 3.61 (m, 2H), 5.69 (s, 2H), 6.61 (d, 1H), 7.24 - 7.32 (m, 3H), 7.42 - 7.50 (m, 4H), 8.27 - 8.33 (m, 1H), 8.35 - 8.40 (m, 1H), 10.81 (s, 1H).

1-(3,5-difluoro-4-{[3-(trifluoromethyl)-1-{[2-(trimethylsilyl)ethoxy]methyl}-1H-pyrrolo[2,3-b]pyridin-4-yl]oxy}phenyl)-3-[3-(morpholin-4-yl)propyl]urea (S3)

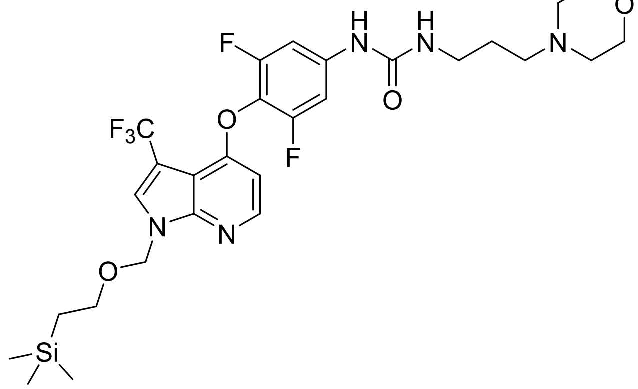

To solution of phenyl (3,5-difluoro-4-{[3-(trifluoromethyl)-1-{[2-(trimethylsilyl)ethoxy]methyl}-1H-pyrrolo[2,3-b]pyridin-4-yl]oxy}phenyl)carbamate (1.20 g, 2.07 mmol, **S2**) in DMF (10 mL) was added 3-(morpholin-4-yl)propan-1-amine (300 µL, 2.1 mmol) and this mixture was stirred at 60°C for 2 hours. After cooling to room temperature ethyl acetate and water was added. After separation of the organic phase the aqueous phase was extracted two times with ethyl acetate. The combined organic phases were washed 3 times with half concentrated aqueous sodium chloride solution, dried over sodium sulfate, filtered and concentrated to dryness.
The resulting residue was purified 3 times via a Biotage chromatography system (55g and 2 times 28g snap KP-NH column, hexane / 0 – 100% ethyl acetate, then ethyl acetate / 0 – 100% methanol) to obtain 970 mg (95 % purity, 71 % yield) of the desired title compound.
Method 2, UPLC-MS (ESI+): t_R = 1.14 min; *m*/*z* calcd for $C_{28}H_{37}F_5N_5O_4Si$ $[M+H]^+$: 630.2; found: 630.4

^{1}H-NMR (400 MHz, DMSO-d6) δ [ppm]: -0.12 - -0.08 (m, 9H), 0.83 (t, 2H), 1.60 (quin, 2H), 2.27 - 2.36 (m, 6H), 3.13 (q, 2H), 3.53 - 3.62 (m, 6H), 5.68 (s, 2H), 6.42 (t, 1H), 6.57 (d, 1H), 7.34 - 7.41 (m, 2H), 8.28 (d, 1H), 8.36 (s, 1H), 8.99 (s, 1H).

Compound 2

1-(3,5-difluoro-4-{[3-(trifluoromethyl)-1H-pyrrolo[2,3-b]pyridin-4-yl]oxy}phenyl)-3-[3-(morpholin-4-yl)propyl]urea

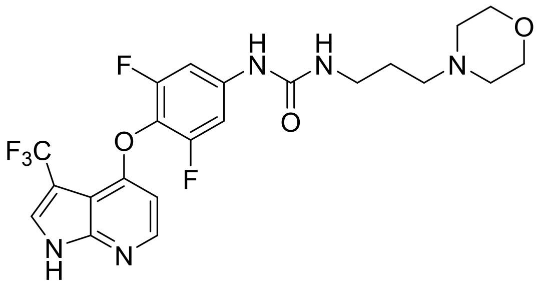

To a solution of 1-(3,5-difluoro-4-{[3-(trifluoromethyl)-1-{[2-(trimethylsilyl)ethoxy]methyl}-1H-pyrrolo[2,3-b]pyridin-4-yl]oxy}phenyl)-3-[3-(morpholin-4-yl)propyl]urea (970 mg, 1.54 mmol **S3**) in dichloromethane (21 mL) was added trifluoroacetic acid (10 mL, 140 mmol) and this mixture was stirred for 3 hours at room temperature. The reaction mixture was carefully poured into an aqueous solution of sodium bicarbonate. This aqueous phase was extracted two times with ethyl acetate. Then the combined organic phases were washed with brine, dried over sodium sulfate and then after filtration evaporated to dryness in vacuum. The obtained crude product was purified via a Biotage chromatography system (25 g snap KP-NH column, hexane / 0 – 100% ethyl acetate, then ethyl acetate / 0 – 100% methanol) to obtain 547 mg (97 % purity, 69 % yield) of the desired title compound.

Method 1, UPLC-MS (ESI+): t_R = 1.06 min; *m*/*z* calcd for $C_{22}H_{23}F_5N_5O_3$ $[M + H]^+$: 500.2; found: 500.4

^{1}H-NMR (400 MHz, DMSO-d6) δ [ppm]: 1.60 (quin, 2H), 2.27 - 2.37 (m, 6H), 3.12 (q, 2H), 3.58 (t, 4H), 6.42 (t, 1H), 6.46 (d, 1H), 7.34 - 7.40 (m, 2H), 8.11 (s, 1H), 8.21 (d, 1H), 8.98 (s, 1H), 12.63 (s, 1H).

4-{[6-chloro-3-(trifluoromethyl)-1-{[2-(trimethylsilyl)ethoxy]methyl}-1H-pyrrolo[2,3-b]pyridin-4-yl]oxy}-3-fluoroaniline (S4)

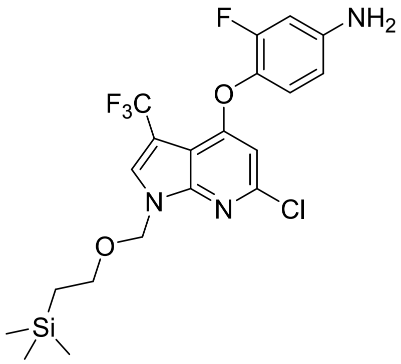

In analogy to intermediate **S8**, using 6-chloro-4-nitro-3-(trifluoromethyl)-1-{[2-(trimethylsilyl)ethoxy]methyl}-1H-pyrrolo[2,3-b]pyridine (500 mg, 1.26 mmol), 4-amino-2-fluorophenol (241 mg, 1.89 mmol) and K2CO3 (524 mg, 3.79 mmol)[56] in DMSO (5.0 mL) we obtained after one single purification using a Biotage chromatography system 341 mg (88 % purity, 50 % yield) of the desired title compound.

Method 1, UPLC-MS (ESI+): t_R = 1.61 min; *m*/*z* calcd for $C_{20}H_{23}ClF_4N_3O_2Si$ $[M + H]^+$: 476.1; found: 476.3

^{1}H-NMR (400 MHz, DMSO-d6) δ [ppm]: -0.10 - -0.06 (m, 9H), 0.81 - 0.88 (m, 2H), 3.54 - 3.60 (m, 2H), 5.57 (s, 2H), 5.61 (s, 2H), 6.36 (d, 1H), 6.45 (ddd, 1H), 6.54 (dd, 1H), 7.07 (t, 1H), 8.35 (d, 1H).

3-fluoro-4-{[3-(trifluoromethyl)-1-{[2-(trimethylsilyl)ethoxy]methyl}-1H-pyrrolo[2,3-b]pyridin-4-yl]oxy}aniline (S5)

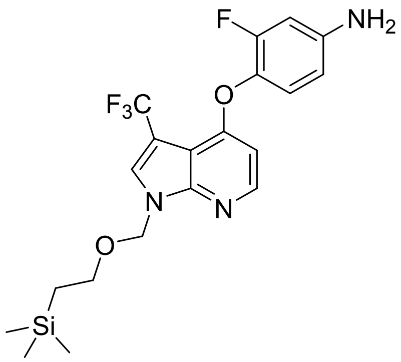

In analogy to intermediate **S9**, using 4-{[6-chloro-3-(trifluoromethyl)-1-{[2-(trimethylsilyl)ethoxy]methyl}-1H-pyrrolo[2,3-b]pyridin-4-yl]oxy}-3-fluoroaniline (335 mg, 704 µmol), **S4**), triethylamine (120 µL, 840 µmol) and 10% Pd on carbon (33.5 mg) in ethanol (24 mL),

we obtained after one single purification using a Biotage chromatography system 240 mg (92 % purity, 66 % yield) of the desired title compound.

Method 1, UPLC-MS (ESI+): t_R = 1.51 min; *m/z* calcd for $C_{20}H_{24}F_4N_3O_2Si$ $[M + H]^+$: 442.2; found: 442.4

^{1}H-NMR (400 MHz, DMSO-d6) δ [ppm]: -0.11 - -0.07 (m, 9H), 0.77 - 0.89 (m, 2H), 3.53 - 3.60 (m, 2H), 5.49 (s, 2H), 5.66 (s, 2H), 6.40 - 6.47 (m, 2H), 6.52 (dd, 1H), 7.03 (t, 1H), 8.24 (d, 1H), 8.29 (s, 1H).

Phenyl (3-fluoro-4-{[3-(trifluoromethyl)-1-{[2-(trimethylsilyl)ethoxy]methyl}-1H-pyrrolo[2,3-b]pyridin-4-yl]oxy}phenyl)carbamate (S6)

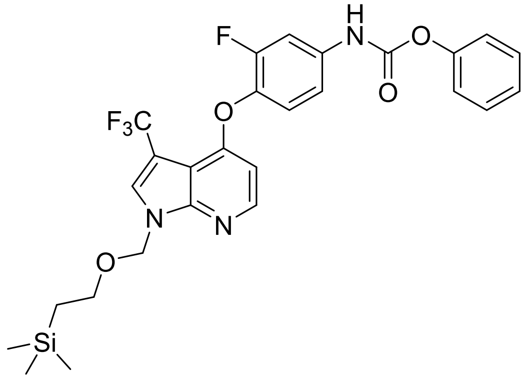

In analogy to intermediate **S2**, using 3-fluoro-4-{[3-(trifluoromethyl)-1-{[2-(trimethylsilyl)ethoxy]methyl}-1H-pyrrolo[2,3-b]pyridin-4-yl]oxy}aniline (235 mg, 532 µmol, **S5**) and phenyl carbonochloridate (73 µL, 590 µmol) in pyridine (240 µL, 3.0 mmol) and THF (3.7 mL), we obtained after one single purification using a Biotage chromatography system 83.8 mg (72 % purity, 20 % yield) of the desired title compound.

Method 1, UPLC-MS (ESI+): t_R = 1.64 min; *m/z* calcd for $C_{27}H_{28}F_4N_3O_4Si$ $[M + H]^+$: 562.2; found: 562.4

^{1}H-NMR (400 MHz, DMSO-d6) δ [ppm]: -0.10 - -0.08 (m, 9H), 0.80 - 0.87 (m, 2H), 3.54 - 3.60 (m, 2H), 5.68 (s, 2H), 6.49 (dd, 1H), 6.72 - 6.78 (m, 2H), 7.12 - 7.31 (m, 4H), 7.37 - 7.48 (m, 1H), 7.65 (dd, 1H), 8.27 (d, 1H), 8.35 (s, 1H), 10.62 (br s, 1H).

1-(3-fluoro-4-{[3-(trifluoromethyl)-1-{[2-(trimethylsilyl)ethoxy]methyl}-1H-pyrrolo[2,3-b]pyridin-4-yl]oxy}phenyl)-3-[3-(morpholin-4-yl)propyl]urea (S7)

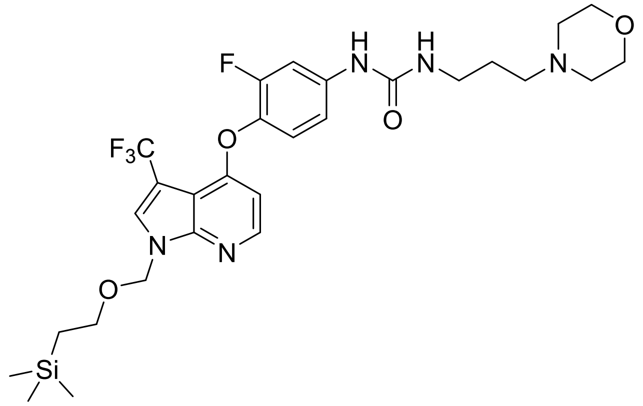

In analogy to intermediate **S3**, using phenyl (3-fluoro-4-{[3-(trifluoromethyl)-1-{[2-(trimethylsilyl)ethoxy]methyl}-1H-pyrrolo[2,3-b]pyridin-4-yl]oxy}phenyl)carbamate (80.0 mg, 142 µmol, **S6**) and 3-(morpholin-4-yl)propan-1-amine (21 µL, 140 µmol) in DMF (700 µL), we obtained after one single purification using a Biotage chromatography system 58.9 mg (93 % purity, 63 % yield) of the desired title compound.

Method 1, UPLC-MS (ESI+): t_R = 1.45 min; *m/z* calcd for $C_{28}H_{38}F_4N_5O_4Si$ $[M + H]^+$: 612.3; found: 612.5

^{1}H-NMR (400 MHz, DMSO-d6) δ [ppm]: -0.10 - -0.08 (m, 9H), 0.80 - 0.87 (m, 2H), 1.60 (quin, 2H), 2.27 - 2.37 (m, 6H), 3.09 - 3.16 (m, 2H), 3.54 - 3.61 (m, 6H), 5.68 (s, 2H), 6.28 (t, 1H), 6.45 (dd, 1H), 7.12 - 7.18 (m, 1H), 7.27 (t, 1H), 7.68 (dd, 1H), 8.26 (d, 1H), 8.33 (s, 1H), 8.81 (s, 1H).

Compound 3

1-(3-fluoro-4-{[3-(trifluoromethyl)-1H-pyrrolo[2,3-b]pyridin-4-yl]oxy}phenyl)-3-[3-(morpholin-4-yl)propyl]urea

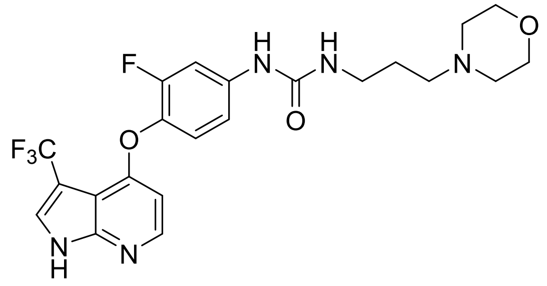

In analogy to compound **2**, 1-(3-fluoro-4-{[3-(trifluoromethyl)-1-{[2-(trimethylsilyl)ethoxy]methyl}-1H-pyrrolo[2,3-b]pyridin-4-yl]oxy}phenyl)-3-[3-(morpholin-4-yl)propyl]urea (56.0 mg, 91.5 µmol, **S7**) was stirred with trifluoroacetic acid (630 µL, 8.1 mmol) in dichloromethane (1.4 mL) to obtain the title compound (33.0 mg, 70 % yield).

Method 1, UPLC-MS (ESI+): t_R = 1.00 min; *m/z* calcd for $C_{22}H_{24}F_4N_5O_3$ $[M + H]^+$: 482.2; found: 482.4

^{1}H-NMR (400 MHz, DMSO-d6) δ [ppm]: 1.60 (quin, 2H), 2.27 - 2.37 (m, 6H), 3.12 (q, 2H), 3.58 (t, 4H), 6.29 (t, 1H), 6.36 (dd, 1H), 7.10 - 7.16 (m, 1H), 7.25 (t, 1H), 7.69 (dd, 1H), 8.08 (s, 1H), 8.18 (d, 1H), 8.81 (s, 1H), 12.56 (br s, 1H).

4-{[6-chloro-3-(trifluoromethyl)-1-{[2-(trimethylsilyl)ethoxy]methyl}-1H-pyrrolo[2,3-b]pyridin-4-yl]oxy}aniline (S8)

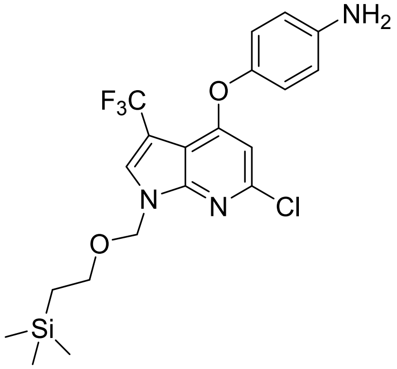

A solution of 6-chloro-4-nitro-3-(trifluoromethyl)-1-{[2-(trimethylsilyl)ethoxy]methyl}-1H-pyrrolo[2,3-b]pyridine (500 mg, 1.26 mmol)[57-58], 4-aminophenol (207 mg, 1.89 mmol) and potassium carbonate (524 mg, 3.79 mmol) in DMSO (5.0 mL) was stirred at 120°C for 3 hours. After cooling to room temperature the reaction mixture was diluted with ethyl acetate (200 mL). This organic phase was washed twice with water (30 mL) and once with brine (20 mL), then dried over sodium sulfate and after filtration dried to dryness. The resulting residue was purified via a Biotage chromatography system (28g snap KP-NH column, hexane / 0 – 70% ethyl acetate) to obtain 479 mg (100 % purity, 83 % yield) of the desired title compound.
Method 1, UPLC-MS (ESI+): t_R = 1.61 min; *m/z* calcd for $C_{20}H_{24}ClF_3N_3O_2Si$ $[M + H]^+$: 458.1; found: 458.3

^{1}H-NMR (400 MHz, DMSO-d6) δ [ppm]: -0.10 - -0.06 (m, 9H), 0.81 - 0.89 (m, 2H), 3.53 - 3.60 (m, 2H), 5.24 (s, 2H), 5.60 (s, 2H), 6.31 (s, 1H), 6.63 - 6.68 (m, 2H), 6.89 - 6.94 (m, 2H), 8.32 (d, 1H).

4-{[3-(trifluoromethyl)-1-{[2-(trimethylsilyl)ethoxy]methyl}-1H-pyrrolo[2,3-b]pyridin-4-yl]oxy}aniline (S9)

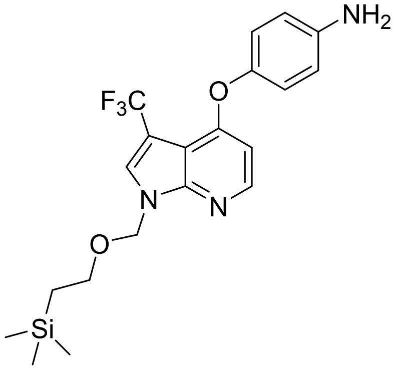

To a a solution of 4-{[6-chloro-3-(trifluoromethyl)-1-{[2-(trimethylsilyl)ethoxy]methyl}-1H-pyrrolo[2,3-b]pyridin-4-yl]oxy}aniline (474 mg, 1.04 mmol, **S8**) and triethylamine (170 µL, 1.2 mmol) in ethanol (35 mL) was given 10% Pd on carbon (47.4 mg). This mixture was stirred in an hydrogen atmosphere for 7 hours at room temperature. Then the mixture was filtered through Celite and the Celite was washed with ethyl acetate. The organic phase was evaporated to dryness and the resulting residue was purified via a Biotage chromatography system (28g snap KP-NH column, hexane / 0 – 70% ethyl acetate) to obtain 424 mg (100 % purity, 97 % yield) of the desired title compound.

Method 1, UPLC-MS (ESI+): t_R = 1.50 min; *m/z* calcd for $C_{20}H_{25}F_3N_3O_2Si$ $[M + H]^+$: 424.2; found: 424.4

^{1}H-NMR (400 MHz, DMSO-d6) δ [ppm]: -0.10 - -0.07 (m, 9H), 0.79 - 0.87 (m, 2H), 3.53 - 3.59 (m, 2H), 5.16 (s, 2H), 5.66 (s, 2H), 6.41 (d, 1H), 6.62 - 6.67 (m, 2H), 6.85 - 6.90 (m, 2H), 8.20 - 8.22 (m, 1H), 8.21 (d, 1H), 8.26 (s, 1H).

Phenyl (4-{[3-(trifluoromethyl)-1-{[2-(trimethylsilyl)ethoxy]methyl}-1H-pyrrolo[2,3-b]pyridin-4-yl]oxy}phenyl)carbamate (S10)

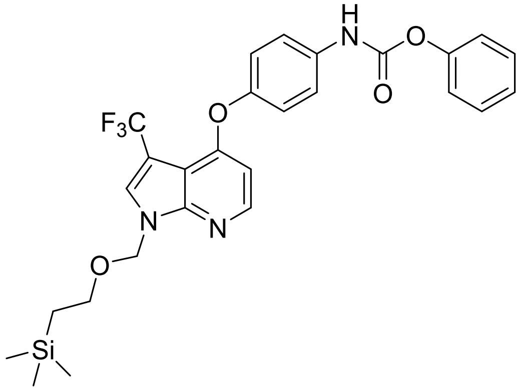

To solution of 4-{[3-(trifluoromethyl)-1-{[2-(trimethylsilyl)ethoxy]methyl}-1H-pyrrolo[2,3-b]pyridin-4-yl]oxy}aniline (420 mg, 992 µmol, **S9**) in pyridine (0.46 mL) and THF (6.8 mL) was slowly added phenyl carbonochloridate (140 µL, 1.1 mmol) at 0°C. After stirring this mixture for 5 minutes at 0°C and then for 30 minutes at room temperature ethyl acetate (150 mL) was added. This organic phase was washed with 1N hydrochloric acid (30 mL), water, concentrated aqueous sodium bicarbonate, brine, dried over sodium sulfate, filtered and concentrated to dryness. The resulting residue was purified via a Biotage chromatography system (100g snap KP-NH column, hexane / 10 – 70% ethyl acetate) to obtain 471 mg (79 % purity, 69 % yield) of the desired title compound. Method 2, UPLC-MS (ESI+): t_R = 1.61 min; *m/z* calcd for $C_{27}H_{29}F_3N_3O_4Si$ $[M + H]^+$: 544.2; found: 544.3

^1H-NMR (400 MHz, DMSO-d6) δ [ppm]: -0.11 - -0.07 (m, 9H), 0.80 - 0.88 (m, 2H), 3.54 - 3.60 (m, 2H), 5.68 (s, 2H), 6.72 - 6.76 (m, 3H), 7.12 - 7.30 (m, 4H), 7.40 - 7.48 (m, 1H), 7.62 (d, 2H), 8.26 (d, 1H), 8.32 (s, 1H), 10.39 (br s, 1H).

1-[3-(morpholin-4-yl)propyl]-3-(4-{[3-(trifluoromethyl)-1-{[2-(trimethylsilyl)ethoxy]methyl}-1H-pyrrolo[2,3-b]pyridin-4-yl]oxy}phenyl)urea (S11)

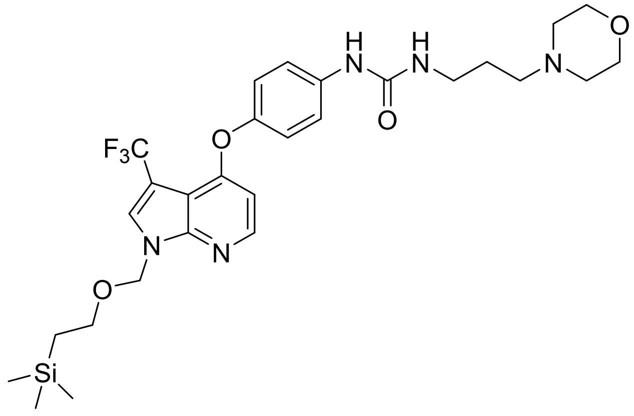

In analogy to intermediate **S3**, using phenyl (4-{[3-(trifluoromethyl)-1-{[2-(trimethylsilyl)ethoxy]methyl}-1H-pyrrolo[2,3-b]pyridin-4-yl]oxy}phenyl)carbamate (150 mg, 276 µmol, **S10**) and 3-(morpholin-4-yl)propan-1-amine (40 µL, 280 µmol) in DMF (1.3 mL), we obtained after one single purification using a Biotage chromatography system 119 mg (93 % purity, 68 % yield) of the desired title compound.

Method 1, UPLC-MS (ESI+): t_R = 1.43 min; *m/z* calcd for $C_{28}H_{39}F_3N_5O_4Si$ $[M + H]^+$: 594.3; found: 594.5

^{1}H-NMR (400 MHz, DMSO-d6) δ [ppm]: -0.11 - -0.06 (m, 9H), 0.80 - 0.88 (m, 2H), 1.59 (quin, 2H), 2.25 - 2.40 (m, 6H), 3.12 (q, 2H), 3.53 - 3.60 (m, 6H), 5.67 (s, 2H), 6.17 (t, 1H), 6.45 (d, 1H), 7.06 - 7.11 (m, 2H), 7.47 - 7.52 (m, 2H), 8.24 (d, 1H), 8.30 (s, 1H), 8.58 (s, 1H).

<u>**Compound 4**</u>

1-[3-(morpholin-4-yl)propyl]-3-(4-{[3-(trifluoromethyl)-1H-pyrrolo[2,3-b]pyridin-4-yl]oxy}phenyl)urea

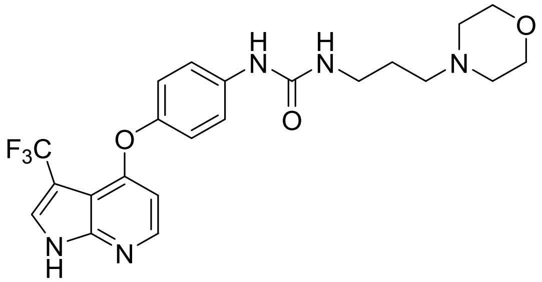

In analogy to compound **2**, 1-[3-(morpholin-4-yl)propyl]-3-(4-{[3-(trifluoromethyl)-1-{[2-(trimethylsilyl)ethoxy]methyl}-1H-pyrrolo[2,3-b]pyridin-4-yl]oxy}phenyl)urea (115 mg, 194 µmol)

was stirred with trifluoroacetic acid (1.3 mL, 17 mmol) in dichloromethane (2.6 mL) to obtain 71.1 mg (97 % purity, 77 % yield) of the desired title compound.

Method 1, UPLC-MS (ESI+): t_R = 0.97 min; *m/z* calcd for $C_{22}H_{25}F_3N_5O_3$ [M + H]$^+$: 464.2; found: 464.5

^{1}H-NMR (400 MHz, DMSO-d6) δ [ppm]: 1.59 (quin, 2H), 2.27 - 2.38 (m, 6H), 3.12 (q, 2H), 3.57 (t, 4H), 6.16 (t, 1H), 6.36 (d, 1H), 7.05 - 7.11 (m, 2H), 7.46 - 7.51 (m, 2H), 8.05 (s, 1H), 8.17 (d, 1H), 8.57 (s, 1H), 12.50 (s, 1H).

4-{[6-chloro-3-(trifluoromethyl)-1-{[2-(trimethylsilyl)ethoxy]methyl}-1H-pyrrolo[2,3-b]pyridin-4-yl]oxy}-2,5-difluoroaniline (S12)

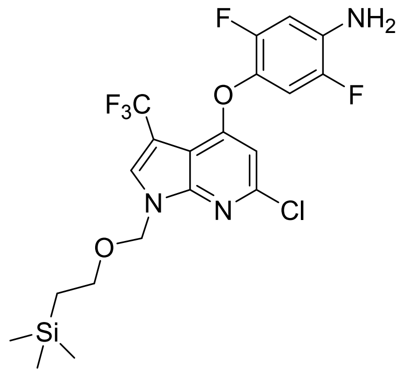

6-chloro-4-nitro-3-(trifluoromethyl)-1-{[2-(trimethylsilyl)ethoxy]methyl}-1H-pyrrolo[2,3-b]pyridine (500 mg, 1.26 mmol)[56] and 4-amino-2,5-difluorophenol (202 mg, 1.39 mmol) were dissolved in dimethyl sulfoxide (5 mL) with ground K_2CO_3 (524 mg, 3.79 mmol; CAS-RN:[584-08-7]) and stirred for 120°C for 3 h. The reaction mixture was cooled down to room temperature and diluted with ethyl acetate (150 mL), washed twice with water (30 mL x 2) and once with brine (20 mL). The organic layer was dried over sodium sulfate and after filtration dried to dryness under reduced pressure. The crude material was purified by silico gel column chromatography (hexane:ethyl acetate 0 to 70%) to give 364 mg of the desired title compound (90% purity, 53% yield).

Method 1, UPLC-MS (ESI+): t_R = 1.61 min; *m/z* calcd for $C_{20}H_{21}ClF_5N_3O_2Si$ [M + H]$^+$: 494.1; found: 494.4

^{1}H-NMR (400MHz, DMSO-d6): δ [ppm]= -0.08 (s, 9H), 0.83 – 0.87 (m, 2H), 3.55 - 3.59 (m, 2H), 5.58 - 5.62 (m, 4H), 6.48 - 6.50 (m, 1H), 6.76 (dd, 1H, *J*=8.4, 12.2 Hz), 7.30 (dd, 1H, *J*=7.5, 11.0 Hz), 8.37 (s, 1H).

2,5-difluoro-4-{[3-(trifluoromethyl)-1-{[2-(trimethylsilyl)ethoxy]methyl}-1H-pyrrolo[2,3-b]pyridin-4-yl]oxy}aniline (S13)

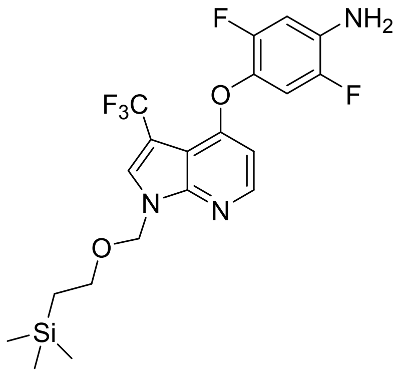

To a solution of 4-{[6-chloro-3-(trifluoromethyl)-1-{[2-(trimethylsilyl)ethoxy]methyl}-1H-pyrrolo[2,3-b]pyridin-4-yl]oxy}-2,5-difluoroaniline (298 mg, 603 µmol, **S12**) and triethylamine (100 µL, 720 µmol) in ethanol (21 mL) was given 10% Pd on carbon (29.8 mg). This mixture was stirred in an hydrogen atmosphere for 7 hours at room temperature. Then the mixture was filtered through Celite and the Celite was washed with ethyl acetate. The organic phase was evaporated to dryness and the resulting residue was purified via a Biotage chromatography system (28g snap KP-NH column, hexane / 10 – 70% ethyl acetate) to obtain 174 mg (90 % purity, 61 % yield) of the desired title compound.

Method 2, UPLC-MS (ESI+): t_R = 1.54 min; *m*/*z* calcd for $C_{20}H_{23}F_5N_3O_2Si$ $[M + H]^+$: 460.1; found: 460.4

^{1}H-NMR (400 MHz, DMSO-d6) δ [ppm]: -0.10 - -0.07 (m, 9H), 0.80 - 0.87 (m, 2H), 3.53 - 3.60 (m, 2H), 5.54 (s, 2H), 5.67 (s, 2H), 6.50 (dd, 1H), 6.75 (dd, 1H), 7.23 (dd, 1H), 8.25 (d, 1H), 8.31 (d, 1H).

Phenyl (2,5-difluoro-4-{[3-(trifluoromethyl)-1-{[2-(trimethylsilyl)ethoxy]methyl}-1H-pyrrolo[2,3-b]pyridin-4-yl]oxy}phenyl)carbamate (S14)

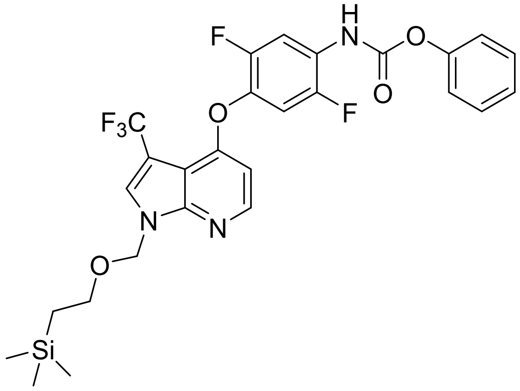

In analogy to intermediate **S2**, using 2,5-difluoro-4-{[3-(trifluoromethyl)-1-{[2-(trimethylsilyl)ethoxy]methyl}-1H-pyrrolo[2,3-b]pyridin-4-yl]oxy}aniline (170 mg, 370 µmol, **S13**) and phenyl carbonochloridate (51 µL, 407 µmol) in pyridine (170 µL, 2.1 mmol), we obtained after one single purification using a Biotage chromatography system 195 mg (95 % purity, 91 % yield) of the desired title compound.

Method 2, UPLC-MS (ESI+): t_R = 1.65 min; *m/z* calcd for $C_{27}H_{27}F_5N_3O_4Si$ $[M + H]^+$: 580.2; found: 580.4

^{1}H-NMR (400 MHz, DMSO-d6) δ [ppm]: -0.10 - -0.07 (m, 9H), 0.79 - 0.87 (m, 2H), 3.54 - 3.61 (m, 2H), 5.69 (s, 2H), 6.64 (dd, 1H), 6.72 - 6.78 (m, 2H), 7.13 - 7.31 (m, 2H), 7.42 - 7.48 (m, 1H), 7.61 (dd, 1H), 7.88 (dd, 1H), 8.30 (d, 1H), 8.37 (d, 1H), 9.32 (s, 1H).

1-(2,5-difluoro-4-{[3-(trifluoromethyl)-1-{[2-(trimethylsilyl)ethoxy]methyl}-1H-pyrrolo[2,3-b]pyridin-4-yl]oxy}phenyl)-3-[3-(morpholin-4-yl)propyl]urea (S15)

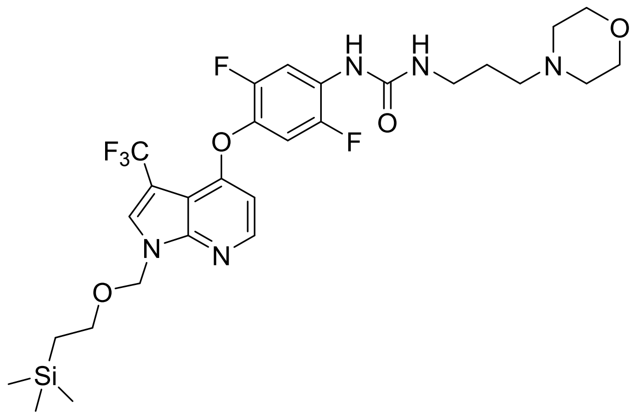

In analogy to intermediate **S3**, phenyl (2,5-difluoro-4-{[3-(trifluoromethyl)-1-{[2-(trimethylsilyl)ethoxy]methyl}-1H-pyrrolo[2,3-b]pyridin-4-yl]oxy}phenyl)carbamate (90.0 mg, 155

µmol, **S14**) and 3-(morpholin-4-yl)propan-1-amine (45 µL, 311 µmol) in DMF (750 µL), we obtained after one single purification using a Biotage chromatography system 93.9 mg (89 % purity, 94 % yield) of the desired title compound.
Method 2, UPLC-MS (ESI+): t_R = 1.19 min; *m/z* calcd for $C_{28}H_{37}F_5N_5O_4Si$ [M + H]$^+$: 630.3; found: 630.5

^{1}H-NMR (400 MHz, DMSO-d6) δ [ppm]: -0.11 - -0.07 (m, 9H), 0.80 - 0.88 (m, 2H), 1.60 (quin, 2H), 2.27 - 2.38 (m, 6H), 3.14 (q, 2H), 3.54 - 3.61 (m, 6H), 5.68 (s, 2H), 6.56 (dd, 1H), 6.69 - 6.78 (m, 1H), 7.51 (dd, 1H), 8.21 - 8.29 (m, 2H), 8.34 (d, 1H), 8.59 (s, 1H).

Compound 5

1-(2,5-difluoro-4-{[3-(trifluoromethyl)-1H-pyrrolo[2,3-b]pyridin-4-yl]oxy}phenyl)-3-[3-(morpholin-4-yl)propyl]urea

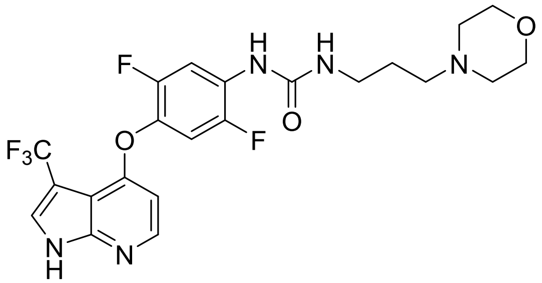

In analogy to compound **2**, 1-(2,5-difluoro-4-{[3-(trifluoromethyl)-1-{[2-(trimethylsilyl)ethoxy]methyl}-1H-pyrrolo[2,3-b]pyridin-4-yl]oxy}phenyl)-3-[3-(morpholin-4-yl)propyl]urea (90.0 mg, 143 µmol) was stirred with trifluoroacetic acid (2.2 mL, 28.6 mmol) in dichloromethane (4.4 mL) to obtain 72 mg (90 % purity, 91 % yield) of the desired title compound.
Method 2, UPLC-MS (ESI+): t_R = 0.86 min; *m/z* calcd for $C_{22}H_{23}F_5N_5O_3$ [M + H]$^+$: 500.2; found: 500.2

^{1}H-NMR (400MHz, DMSO-d6) δ [ppm]: 1.60 (quin, 2H), 2.27 - 2.38 (m, 6H), 3.10 - 3.18 (m, 2H), 3.58 (t, 4H), 6.46 (d, 1H), 6.72 (t, 1H), 7.48 (dd, 1H), 8.09 (s, 1H), 8.20 (d, 1H), 8.24 (dd, 1H), 8.58 (s, 1H), 12.59 (br s, 1H).

1-(3,5-difluoro-4-{[3-(trifluoromethyl)-1-{[2-(trimethylsilyl)ethoxy]methyl}-1H-pyrrolo[2,3-b]pyridin-4-yl]oxy}phenyl)-3-[2-(morpholin-4-yl)-2-oxoethyl]urea (S16)

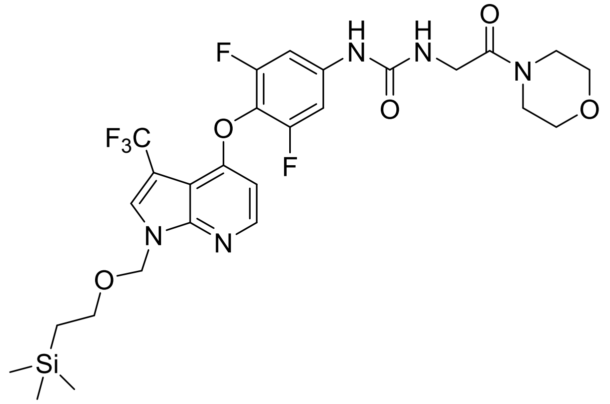

In analogy to intermediate **S3,** using phenyl (3,5-difluoro-4-{[3-(trifluoromethyl)-1-{[2-(trimethylsilyl)ethoxy]methyl}-1H-pyrrolo[2,3-b]pyridin-4-yl]oxy}phenyl)carbamate (150 mg, 259 µmol, **S2**) and 2-amino-1-(morpholin-4-yl)ethanone (37.3 mg, 259 µmol) in DMF (1.5 mL), we obtained after one single purification using a Biotage chromatography system 124 mg (94 % purity, 72 % yield) of the desired title compound.

Method 2, UPLC-MS (ESI+): t_R = 1.44 min; *m/z* calcd for $C_{27}H_{33}F_5N_5O_5Si$ [M + H]$^+$: 630.2; found: 630.3

^{1}H-NMR (400 MHz, DMSO-d6) δ [ppm]: -0.11 - -0.08 (m, 9H), 0.80 - 0.86 (m, 2H), 3.39 - 3.49 (m, 4H), 3.54 - 3.62 (m, 6H), 4.01 (d, 2H), 5.68 (s, 2H), 6.54 - 6.62 (m, 2H), 7.33 - 7.40 (m, 2H), 8.28 (d, 1H), 8.36 (s, 1H), 9.46 (s, 1H).

__Compound 6__

1-(3,5-difluoro-4-{[3-(trifluoromethyl)-1H-pyrrolo[2,3-b]pyridin-4-yl]oxy}phenyl)-3-[2-(morpholin-4-yl)-2-oxoethyl]urea

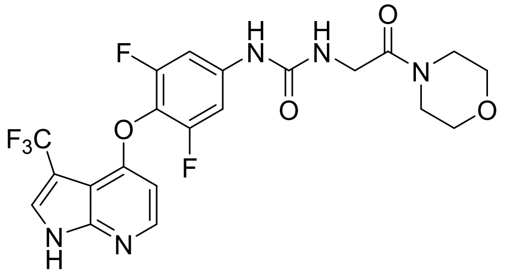

In analogy to compound **2**, 1-(3,5-difluoro-4-{[3-(trifluoromethyl)-1-{[2-(trimethylsilyl)ethoxy]methyl}-1H-pyrrolo[2,3-b]pyridin-4-yl]oxy}phenyl)-3-[2-(morpholin-4-yl)-2-oxoethyl]urea (124 mg, 197 µmol, **S16**) was stirred with trifluoroacetic acid (1.4 mL, 18 mmol) in

dichloromethane (2.8 mL) to obtain 41.5 mg (97 % purity, 41 % yield) of the desired title compound.

Method 1, UPLC-MS (ESI+): t_R = 0.98 min; *m/z* calcd for $C_{21}H_{19}F_5N_5O_4$ $[M + H]^+$: 500.1; found: 500.3

^{1}H-NMR (400 MHz, DMSO-d6) δ [ppm]: 3.39 - 3.49 (m, 4H), 3.58 (dt, 4H), 4.01 (d, 2H), 6.47 (d, 1H), 6.56 (t, 1H), 7.33 - 7.39 (m, 2H), 8.10 - 8.12 (m, 1H), 8.21 (d, 1H), 9.46 (s, 1H), 12.63 (br s, 1H).

1-(3,5-difluoro-4-{[3-(trifluoromethyl)-1-{[2-(trimethylsilyl)ethoxy]methyl}-1H-pyrrolo[2,3-b]pyridin-4-yl]oxy}phenyl)-3-(pyridin-4-ylmethyl)urea (S17)

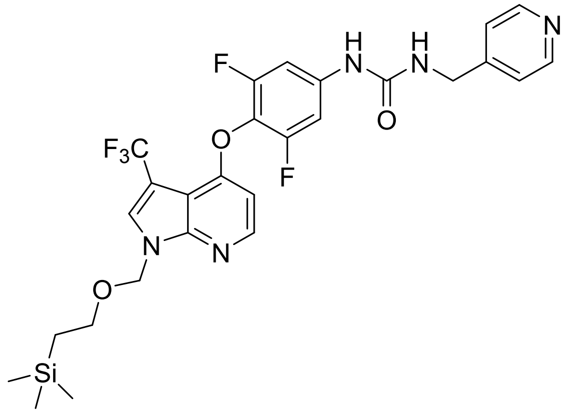

In analogy to intermediate **S3,** using phenyl (3,5-difluoro-4-{[3-(trifluoromethyl)-1-{[2-(trimethylsilyl)ethoxy]methyl}-1H-pyrrolo[2,3-b]pyridin-4-yl]oxy}phenyl)carbamate (150 mg, 259 µmol, **S2**) and 1-(pyridin-4-yl)methanamine (28.0 mg, 259 µmol) in DMF (1.5 mL), we obtained after one single purification using a Biotage chromatography system 145 mg (92 % purity, 87 % yield) of the desired title compound.

Method 2, UPLC-MS (ESI+): t_R = 1.23 min; *m/z* calcd for $C_{27}H_{29}F_5N_5O_3Si$ $[M + H]^+$: 594.2; found: 594.3

^{1}H-NMR (400 MHz, DMSO-d6) δ [ppm]: -0.11 - -0.08 (m, 9H), 0.79 - 0.86 (m, 2H), 3.54 - 3.61 (m, 2H), 4.35 (d, 2H), 5.68 (s, 2H), 6.58 (d, 1H), 7.04 (t, 1H), 7.30 (d, 2H), 7.38 - 7.45 (m, 2H), 8.28 (d, 1H), 8.36 (s, 1H), 8.52 (d, 2H), 9.30 (s, 1H).

<u>**Compound 7**</u>

1-(3,5-difluoro-4-{[3-(trifluoromethyl)-1H-pyrrolo[2,3-b]pyridin-4-yl]oxy}phenyl)-3-(pyridin-4-ylmethyl)urea

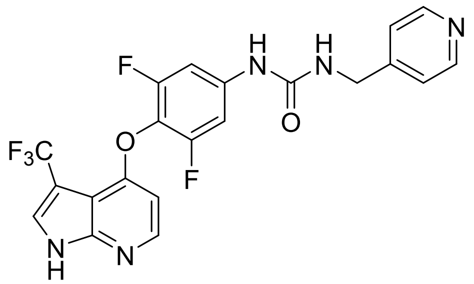

In analogy to compound **2**, 1-(3,5-difluoro-4-{[3-(trifluoromethyl)-1-{[2-(trimethylsilyl)ethoxy]methyl}-1H-pyrrolo[2,3-b]pyridin-4-yl]oxy}phenyl)-3-(pyridin-4-ylmethyl)urea (145 mg, 244 µmol, **S17**) was stirred with trifluoroacetic acid (1.8 mL, 23 mmol) in dichloromethane (3.5 mL) to obtain 61.7 mg (95 % purity, 52 % yield) of the desired title compound.

Method 1, UPLC-MS (ESI+): t_R = 1.02 min; *m/z* calcd for $C_{21}H_{15}F_5N_5O_2$ $[M + H]^+$: 464.1; found: 464.3

^{1}H-NMR (400 MHz, DMSO-d6) δ [ppm]: 4.35 (d, 2H), 6.47 (d, 1H), 7.04 (t, 1H), 7.27 - 7.32 (m, 2H), 7.37 - 7.45 (m, 2H), 8.09 - 8.12 (m, 1H), 8.21 (d, 1H), 8.49 - 8.54 (m, 2H), 9.29 (s, 1H), 12.63 (br s, 1H).

4-(2,6-difluoro-4-nitro-phenoxy)-1H-pyrrolo[2,3-b]pyridine (S18)

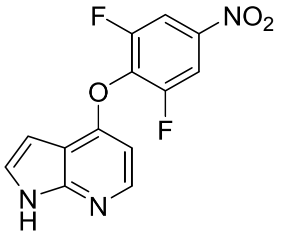

A solution of 1,2,3-trifluoro-5-nitrobenzene (CAS No. [66684-58-0]; 3.59 g, 20.3 mmol) and 1*H*-pyrrolo[2,3-b]pyridin-4-ol (CAS No. [74420-02-3]; 1.10 eq., 2.99 g, 22.3 mmol) in DMSO (65 mL) was treated with potassium carbonate (4.00 eq, 11.2 g, 81.1 mmol) and stirred at room temperature for 1 hour. The reaction mixture was diluted with ethyl acetate (500 mL) and washed with water (3 x 200 mL) and brine (150 mL), dried with sodium sulfate and concentrated in vacuo. The obtained material was purified by flash chromatography (SiO_2-hexane/ ethyl acetate) to give the title compound (3.1 g, 52%).

Method 1, UPLC-MS (ESI+): t_R = 1.13 min; *m/z* calcd for $C_{13}H_8F_2N_3O_3$ [M + H]$^+$: 292.1; found: 291.9

^{1}H-NMR (400 MHz, DMSO-d_6) δ [ppm] = 6.35 (d, 1H), 6.59 (d, 1H), 7.47 (d, 1H), 8.13 (d, 1H), 8.37 – 8.43 (m, 2H), 11.97 (br s, 1H).

4-(2,6-difluoro-4-nitrophenoxy)-1-{[2-(trimethylsilyl)ethoxy]methyl}-1H-pyrrolo[2,3-b]pyridine (S19)

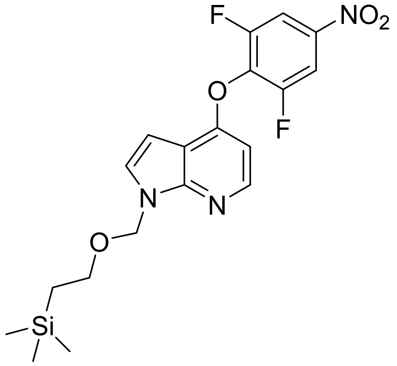

An ice-cooled solution of 4-(2,6-difluoro-4-nitrophenoxy)-1*H*-pyrrolo[2,3-b]pyridine (3.11 g, 10.7 mmol, **S18**) in acetonitrile (100 mL) was treated with *N*,*N*-diisopropyl ethylamine (1.80 eq, 3.35 mL, 19.2 mmol) and [2-(chloromethoxy)ethyl](trimethyl)silane (CAS No. [76513-69-4]; 1.40 eq, 2.65 mL, 15.0 mmol), warmed to rt and stirring continued overnight. The reaction mixture was poured on ice water (150 mL) and the aqueous phase extracted with ethyl acetate (2 x 400 mL). The combined organic layers were washed with brine (150 mL), dried with sodium sulfate and concentrated in vacuo. The obtained material was purified by flash chromatography (KP-NH®-SiO_2-hexane/ ethyl acetate) to give the title compound (3.9 g, 85%).

Method 1, UPLC-MS (ESI+): t_R = 1.59 min; *m/z* calcd for $C_{19}H_{22}F_2N_3O_4Si$ [M + H]$^+$: 422.1; found: 422.4

^{1}H-NMR (400 MHz, DMSO-d_6) δ [ppm] = -0.11 (s, 9H), 0.79 – 0.83 (m, 2H), 3.50 – 3.54 (m, 2H), 5.64 (s, 2H), 6.48 (d, 1H), 6.69 (d, 1H), 7.66 (d, 1H), 8.20 (d, 1H), 8.38 – 8.44 (m, 2H).

4-[3-chloro-1-(2-trimethylsilylethoxymethyl)pyrrolo[2,3-b]pyridin-4-yl]oxy-3,5-difluoro-aniline (S20)

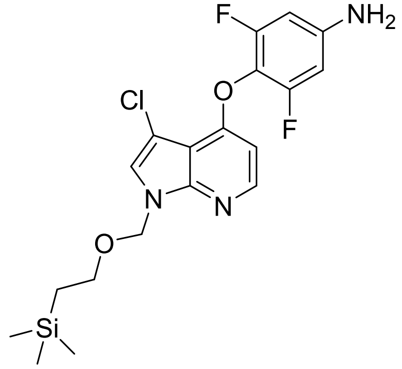

A solution of 2-[[4-(2,6-difluoro-4-nitro-phenoxy)pyrrolo[2,3-b]pyridin-1-yl]methoxy]ethyl-trimethyl-silane (45 g, 107 mmol, **S19**) and N-Chlorosuccinimide (15.7 g, 118 mmol) in CCl_4 (500 mL) was stirred at 60°C for 16 h. The mixture was diluted with water (800 mL) extracted with ethyl acetate (500 mL x 3) and the combined organic layers washed with brine(1.0 L), dried by Na_2SO_4, filtered and evaporated to give the desired chloride intermediate (50 g) as a crude yellow gum, which was directly used in the next step without further purification.

To a solution of aforementioned crude SEM protected nitro intermediate (50 g), NH_4Cl (30 g, 560 mmol, 19.6 mL) in H_2O (250 mL), THF (250 mL) and MeOH (500 mL) was added Fe (30 g, 537 mmol) and the resulting mixture was stirred at 80°C for 2 h. The mixture was filtered, the filtrate was diluted with water (800 mL) extracted with ethyl acetate (300 mL x 3) and the combined organic layers washed with brine (1.0 L), dried by Na_2SO_4, filtered and evaporated to give 39 g of the desired title compound (91.6 mmol, 83.5% yield) as a yellow gum.

^{1}H-NMR (400 MHz, $CDCl_3$) δ [ppm]: -0.04 (s, 9H), 0.95 - 0.91 (m, 2H), 3.58-3.53 (m, 2H), 5.63 (s, 2H), 6.35-6.33 (m, 3H), 8.24 (s, 1H), 8.15-8.13 (m, 1H)

N-({4-[(3-chloro-1-{[2-(trimethylsilyl)ethoxy]methyl}-1H-pyrrolo[2,3-b]pyridin-4-yl)oxy]-3,5-difluorophenyl}carbamothioyl)acetamide (S21)

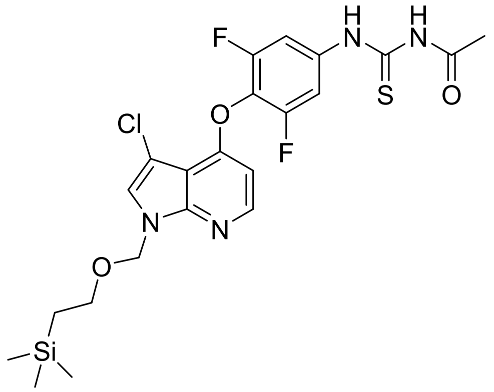

To a stirred solution of 4-[(3-chloro-1-{[2-(trimethylsilyl)ethoxy]methyl}-1H-pyrrolo[2,3-b]pyridin-4-yl)oxy]-3,5-difluoroaniline (100 mg, 235 µmol, **S20**) in acetonitrile (3 mL) was added acetyl isothiocyanate (21 µl, 230 µmol). The resulting mixture was stirred overnight at room temperature at which time the reaction was concentrated under reduced pressure and used without further purification.

Method 1, UPLC-MS (ESI+): t_R = 1.53 min; *m/z* calcd for $C_{22}H_{26}ClF_2N_4O_3SSi$ $[M + H]^+$: 527.1; found: 527.1

^{1}H-NMR (400 MHz, DMSO-d6) δ [ppm]: -0.09 (s, 9H), 0.80 – 0.84 (m, 2H), 2.18 (s, 3H), 3.51 – 3.55 (m, 2H), 5.61 (s, 2H), 6.48 (d, 1H), 7.84 – 7.89 (m, 3H), 8.20 (d, 1H), 11.69 (br s, 1H), 12.64 (br s, 1H).

N-{4-[(3-chloro-1-{[2-(trimethylsilyl)ethoxy]methyl}-1H-pyrrolo[2,3-b]pyridin-4-yl)oxy]-3,5-difluorophenyl}-5-methyl-4H-1,2,4-triazol-3-amine (S22)

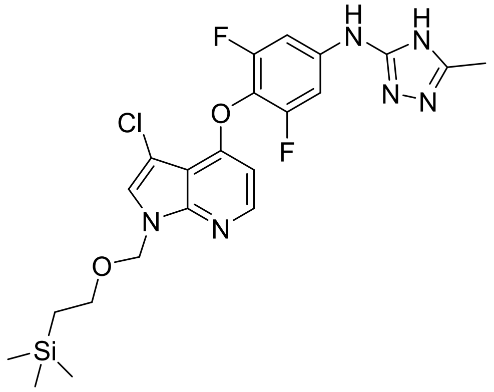

To a stirred solution of N-({4-[(3-chloro-1-{[2-(trimethylsilyl)ethoxy]methyl}-1H-pyrrolo[2,3-b]pyridin-4-yl)oxy]-3,5-difluorophenyl}carbamothioyl)acetamide (120 mg, 228 µmol, **S21**) in

chloroform (3.0 mL) was added hydrazine hydrate (50% in water, 55 µL, 1.1 mmol). The resulting mixture was heated to 70°C for 2.5h, at which time the reaction was concentrated under reduced pressure and issued without further purification.

Method 1, UPLC-MS (ESI+): t_R = 1.40 min; *m/z* calcd for $C_{22}H_{26}ClF_2N_6O_2Si$ $[M + H]^+$: 507.1; found: 507.2

Compound 8

N-{4-[(3-chloro-1H-pyrrolo[2,3-b]pyridin-4-yl)oxy]-3,5-difluorophenyl}-5-methyl-4H-1,2,4-triazol-3-amine

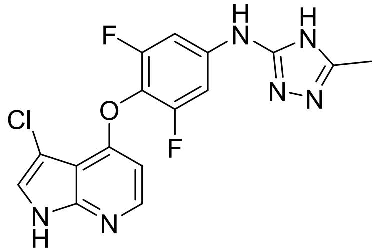

To a stirred solution of N-{4-[(3-chloro-1-{[2-(trimethylsilyl)ethoxy]methyl}-1H-pyrrolo[2,3-b]pyridin-4-yl)oxy]-3,5-difluorophenyl}-5-methyl-4H-1,2,4-triazol-3-amine (103 mg, 203 µmol, **S22**) in dichloromethane (3.0 mL) was added trifluoroacetic acid (520 µl, 6.7 mmol). The resulting mixture was stirred overnight at room temperature, at which time the mixture was poured into a saturated aqueous solution of sodium bicarbonate. The resulting precipitate was collected by filtration and subsequently purified by HPLC to afford the title product N-{4-[(3-chloro-1H-pyrrolo[2,3-b]pyridin-4-yl)oxy]-3,5-difluorophenyl}-5-methyl-4H-1,2,4-triazol-3-amine (20mg, 23% yield over 3 steps).

Method 1, LC-MS (ESI+): t_R = 0.91 min; *m/z* calcd for $C_{16}H_{12}ClF_2N_6O$ $[M + H]^+$: 377.1; found: 377.1

^{1}H-NMR (400 MHz, DMSO-d6) δ [ppm]: 2.32 (s, 3H), 6.33 (d, *J* = 5.6 Hz, 1H), 7.46 (d, *J* = 11.2 Hz, 2H), 7.60 (s, 1H), 8.09 (d, 1H), 9.66 (s, 1H), 12.10 (br s, 1H), 13.07 (br s, 1H).

1-(3,5-difluoro-4-{[3-(trifluoromethyl)-1-{[2-(trimethylsilyl)ethoxy]methyl}-1H-pyrrolo[2,3-b]pyridin-4-yl]oxy}phenyl)-3-(2-methoxyethyl)urea (S23)

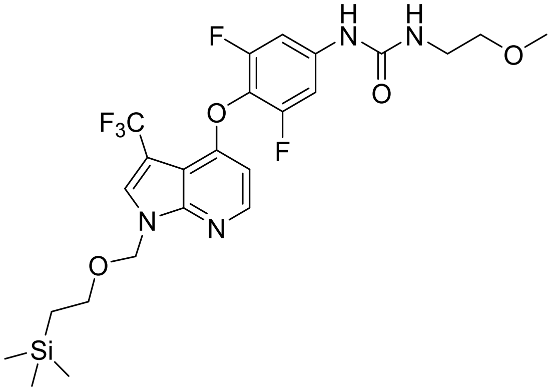

In analogy to intermediate **S3,** using 1-(3,5-difluoro-4-{[3-(trifluoromethyl)-1-{[2-(trimethylsilyl)ethoxy]methyl}-1H-pyrrolo[2,3-b]pyridin-4-yl]oxy}phenyl)-3-(2-methoxyethyl)urea (150 mg, 259 µmol, **S2**) and 2-methoxyethanamine (19.4 mg, 259 µmol) in DMF (1.5 mL), we obtained after one single purification using a Biotage chromatography system 99.8 mg (93 % purity, 64 % yield) of the desired title compound.

Method 2, UPLC-MS (ESI+): t_R = 1.50 min; *m/z* calcd for $C_{24}H_{30}F_5N_4O_4Si$ $[M + H]^+$: 561.2; found: 561.3

^{1}H-NMR (400 MHz, DMSO-d6) δ [ppm]: -0.08 (m, 9H), 0.79 - 0.86 (m, 2H), 3.24 - 3.29 (m, 5H), 3.39 (t, 2H), 3.54 - 3.60 (m, 2H), 5.68 (s, 2H), 6.49 (t, 1H), 6.57 (d, 1H), 7.33 - 7.40 (m, 2H), 8.29 (d, 1H), 8.36 (s, 1H), 9.11 (s, 1H).

<u>Compound 9</u>

1-(3,5-difluoro-4-{[3-(trifluoromethyl)-1H-pyrrolo[2,3-b]pyridin-4-yl]oxy}phenyl)-3-(2-methoxyethyl)urea

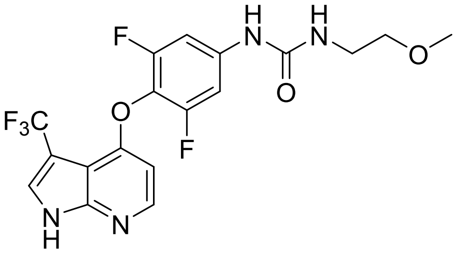

In analogy to compound **2,** 1-(3,5-difluoro-4-{[3-(trifluoromethyl)-1-{[2-(trimethylsilyl)ethoxy]methyl}-1H-pyrrolo[2,3-b]pyridin-4-yl]oxy}phenyl)-3-(2-methoxyethyl)urea (99.8 mg, 178 µmol, **S23**) was stirred with trifluoroacetic acid (1.2 mL, 16 mmol) in dichloromethane (2.4 mL). The Biotage purified product was stirred in dichlormethane (5 mL).

Then the solid was isolated by filtration and dried in vacuum to yield 39 mg (97 % purity, 49 % yield) of the desired title compound.

Method 2, UPLC-MS (ESI+): t_R = 1.04 min; *m/z* calcd for $C_{18}H_{16}F_5N_4O_3$ $[M + H]^+$: 431.1; found: 431.3

^{1}H-NMR (400 MHz, DMSO-d6) δ [ppm]: 3.24 - 3.30 (m, 5H), 3.39 (t, 2H), 6.44 - 6.51 (m, 2H), 7.33 - 7.39 (m, 2H), 8.10 (s, 1H), 8.20 (d, 1H), 9.11 (s, 1H), 12.63 (br s, 1H).

1-(3,5-difluoro-4-{[3-(trifluoromethyl)-1-{[2-(trimethylsilyl)ethoxy]methyl}-1H-pyrrolo[2,3-b]pyridin-4-yl]oxy}phenyl)-3-[(3-methyloxetan-3-yl)methyl]urea (S24)

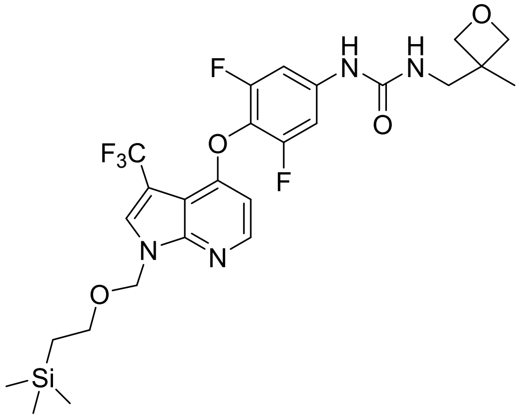

To a solution of phenyl (3,5-difluoro-4-{[3-(trifluoromethyl)-1-{[2-(trimethylsilyl)ethoxy]methyl}-1H-pyrrolo[2,3-b]pyridin-4-yl]oxy}phenyl)carbamate (250 mg, 431 µmol, **S2**) in DMF (2.1 mL) was added 1-(3-methyloxetan-3-yl)methanamine (43.6 mg, 431 µmol, CAS No. [153209-97-3]) and this mixture was stirred at 50 °C for 12 hours. After cooling to room temperature ethyl acetate and water was added. After separation of the organic phase the aqueous phase was extracted two times with ethyl acetate. The combined organic phases were washed with brine, dried over sodium sulfate, filtered and concentrated to dryness. The resulting residue was purified via a Biotage chromatography system (10g snap KP-Sil hexane / 0 – 100% ethyl acetate, then ethyl acetate / 0 – 100% methanol) to obtain 178 mg (80 % purity, 56 % yield) of the desired title compound.

Method 2, UPLC-MS (ESI+): t_R = 1.64 min; *m/z* calcd for $C_{26}H_{32}F_5N_4O_4Si$ $[M + H]^+$: 587.2; found: 587.4

^{1}H-NMR (400 MHz, DMSO-d6) δ [ppm]: -0.15 - -0.06 (m, 9H), 0.79 - 0.87 (m, 2H), 1.23 (s, 3H), 3.25 - 3.31 (m, 2H), 3.54 - 3.62 (m, 2H), 4.20 (d, 2H), 4.39 (d, 2H), 5.68 (s, 2H), 6.58 (d, 1H), 6.93 (t, 1H), 7.36 - 7.44 (m, 2H), 8.28 (d, 1H), 8.36 (s, 1H), 9.26 (br s, 1H).

Compound 10

N-(3,5-difluoro-4-{[3-(trifluoromethyl)-1H-pyrrolo[2,3-b]pyridin-4-yl]oxy}phenyl)-N'-[(3-methyloxetan-3-yl)methyl]urea

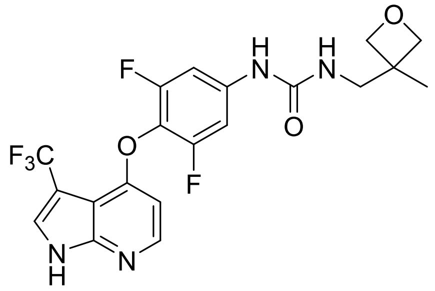

To a stirred solution of N-(3,5-difluoro-4-{[3-(trifluoromethyl)-1-{[2-(trimethylsilyl)ethoxy]methyl}-1H-pyrrolo[2,3-b]pyridin-4-yl]oxy}phenyl)-N'-[(3-methyloxetan-3-yl)methyl]urea (124 mg, 212 µmol, **S24**) in dimethyl formamide (2.5 mL) was added ethylenediamine (58 µl, 870 µmol) and tetra-n-butyl ammonium fluoride (1N in THF, 850 µl, 850 µmol). The resulting mixture was stirred at 45°C overnight at which time the reaction was diluted with ethyl acetate, water was added and the layers were separated. The aqueous phase was extracted 3 times with ethyl acetate, and the combined organic phases were washed with brine, dried over magnesium sulfate, filtered, and evaporated under reduced pressure to afford the crude product. The crude product was purified by HPLC to afford the product (35mg, 34% yield).

Method 1, UPLC-MS (ESI+): t_R = 1.03 min; *m/z* calcd for $C_{20}H_{18}F_5N_4O_3$ $[M + H]^+$: 457.1; found: 457.3

^{1}H-NMR (400 MHz, DMSO-d6) δ [ppm]: 1.23 (s, 3H), 3.29 (m, 2H), 4.39 (d, *J* = 5.8 Hz, 2H), 4.20 (d, *J* = 5.8 Hz, 2H), 6.47 (d, 1H), 6.78 – 6.81 (m, 1H), 7.35 – 7.45 (m, 2H), 8.11, (br s, 1H), 8.20 – 8.22 (m, 1H), 9.12 (br s, 1H),12.63 (br s, 1H).

Compound 11

(+/-)-{2-[(3,5-difluoro-4-{[3-(trifluoromethyl)-1H-pyrrolo[2,3-b]pyridin-4-yl]oxy}phenyl)amino]-5-methyl-5,6-dihydro-4H-1,3-oxazin-5-yl}methanol

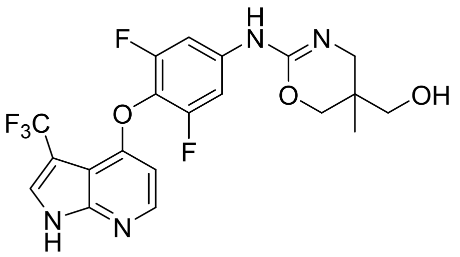

To a solution of 1-(3,5-difluoro-4-{[3-(trifluoromethyl)-1-{[2-(trimethylsilyl)ethoxy]methyl}-1H-pyrrolo[2,3-b]pyridin-4-yl]oxy}phenyl)-3-[(3-methyloxetan-3-yl)methyl]urea (178 mg, 303 µmol, **S24**) in dichloromethane (3.2 mL) was added trifluoroacetic acid (1.6 mL, 21 mmol) and this mixture was stirred for 3 hours at room temperature. The reaction mixture was diluted with ethyl acetate and two times washed with an aqueous solution of sodium bicarbonate, then water, brine, dried over sodium sulfate and after filtration evaporated to dryness in vacuum. The obtained crude product was purified via a Biotage chromatography system (11 g snap KP-NH column, hexane / 0 – 100% ethyl acetate, then ethyl acetate / 0 – 100% methanol) to obtain a product which was finally purified via a preparative HPLC (method 1) to yield 29.2 mg (95% purity, 25.5 % yield) of the desired title compound.

Method 1, UPLC-MS (ESI+): t_R = 1.02 min; *m/z* calcd for $C_{20}H_{18}F_5N_4O_3$ $[M + H]^+$: 457.1; found: 457.3

^{1}H-NMR (400 MHz, DMSO-d6) δ [ppm]: 0.90 (s, 3H), 3.01 (br d, 1H), 3.15 - 3.29 (m, 2H), 3.29 - 3.37 (m, 1H), 3.87 (br d, 1H), 4.06 (br d, 1H), 4.83 (br s, 1H), 6.45 (d, 1H), 7.31 - 7.71 (m, 2H), 8.10 (s, 1H), 8.20 (d, 1H), 9.03 (br s, 1H), 12.60 (br s, 1H).

1-(3,5-difluoro-4-{[3-(trifluoromethyl)-1-{[2-(trimethylsilyl)ethoxy]methyl}-1H-pyrrolo[2,3-b]pyridin-4-yl]oxy}phenyl)-3-[(3-fluorooxetan-3-yl)methyl]urea (S25)

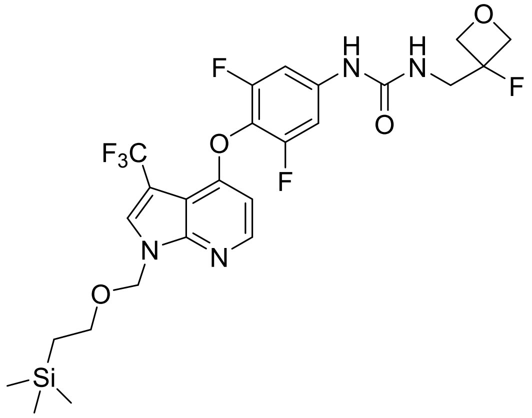

In analogy to intermediate **S3**, phenyl (3,5-difluoro-4-{[3-(trifluoromethyl)-1-{[2-(trimethylsilyl)ethoxy]methyl}-1H-pyrrolo[2,3-b]pyridin-4-yl]oxy}phenyl)carbamate (150 mg, 259 µmol, **S2**), 1-(3-fluorooxetan-3-yl)methanamine (27.2 mg, 259 µmol, CAS No. [883311-82-8]) together in DMF (1.5 mL) and N,N-diisopropylethylamine (45 µL, 260 µmol) we obtained the crude product, which was purified via a Biotage chromatography system (11g snap KP-NH column, hexane/ethyl acetate / 50 – 100% ethyl acetate and ethyl acetate/ethanol / 0 – 40% ethanol) to obtain 133 mg (100 % purity, 87 % yield) of the desired title compound.
Method 1, UPLC-MS (ESI+): t_R = 1.49 min; *m/z* calcd for $C_{25}H_{29}F_6N_4O_3Si$ $[M + H]^+$: 591.2; found: 591.4

^{1}H-NMR (400 MHz, DMSO-d6) δ [ppm]: -0.12 - -0.06 (m, 9H), 0.79 - 0.86 (m, 2H), 3.54 - 3.60 (m, 2H), 3.61 - 3.70 (m, 2H), 4.55 - 4.67 (m, 4H), 5.68 (s, 2H), 6.58 (d, 1H), 6.81 (t, 1H), 7.37 - 7.43 (m, 2H), 8.28 (d, 1H), 8.36 (s, 1H), 9.13 (s, 1H).

(+/-)-{2-[(3,5-difluoro-4-{[3-(trifluoromethyl)-1H-pyrrolo[2,3-b]pyridin-4-yl]oxy}phenyl)amino]-5-fluoro-5,6-dihydro-4H-1,3-oxazin-5-yl}methanol (S26)

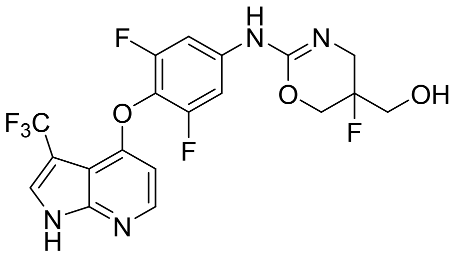

In analogy to compound **11**, 1-(3,5-difluoro-4-{[3-(trifluoromethyl)-1-{[2-(trimethylsilyl)ethoxy]methyl}-1H-pyrrolo[2,3-b]pyridin-4-yl]oxy}phenyl)-3-[(3-fluorooxetan-3-yl)methyl]urea (129 mg, 218 µmol, **S25**) was stirred with trifluoroacetic acid (1.5 mL,19 mmol) in dichloromethane (3.0 mL). After purification using a Biotage chromatography system we obtained 82.5 mg (97 % purity, 80 % yield) of the desired title compound. Method 1, UPLC-MS (ESI+): t_R = 1.00 min; *m/z* calcd for $C_{19}H_{15}F_6N_4O_3$ $[M + H]^+$: 461.1; found: 461.3

^{1}H-NMR (400 MHz, DMSO-d6) δ [ppm]: 3.37 - 3.67 (m, 4H), 4.22 - 4.36 (m, 2H), 5.25 (br t, 1H), 6.45 (d, 1H), 7.57 (d, 2H), 8.10 (s, 1H), 8.20 (d, 1H), 9.23 (br s, 1H), 12.62 (s, 1H).

Compound 12

[(5R*)-2-(3,5-difluoro-4-{[3-(trifluoromethyl)-1H-pyrrolo[2,3-b]pyridin-4-yl]oxy}anilino)-5-fluoro-5,6-dihydro-4H-1,3-oxazin-5-yl]methanol

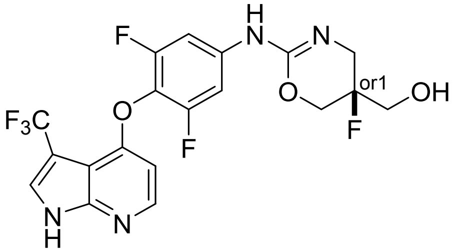

Compound **S26** was separated by chiral HPLC (conditions below) to afford two isomers. As a result, 191 mg of compound **12** (97.9% purity, ee = 96.2%) was obtained.

Analytical HPLC Method:

Instrument: Agilent HPLC 1260; Column: Chiralpak IE 3µ

100x4,6mm; Eluent A: Hexane + 0.1 Vol-% Diethylamine (99%); Eluent B: Ethanol;

Isocratic: 85%A+15%B; Flow rate 1.4 ml/min; Temperature: 25 °C; DAD 254 nm

Compound 12: t_R = 3.99 min

Other enantiomer: t_R = 3.08 min

Preparative Chiral HPLC Method:

Instrument: Labomatic HD5000, Labocord-5000; Gilson GX-241, Labcol Vario 4000,

Column: Chiralpak IE 5µ 250x30mm; Eluent A: Hexane + 0.1 Vol-% Diethylamine (99%);

Eluent B: Ethanol; Isocratic: 85%A+15%B; Flow rate 50.0 ml/min; UV 254 nm

Compound 12:
Method 3, LC-MS (ESI+): t_R = 0.66 min; *m/z* calcd for $C_{19}H_{15}F_6N_4O_3$ $[M + H]^+$: 461.1; found: 461.0

^{1}H-NMR (400 MHz, DMSO-d6) δ [ppm]: 3.37 - 3.67 (m, 4H), 4.22 - 4.36 (m, 2H), 5.25 (br t, 1H), 6.45 (d, 1H), 7.57 (d, 2H), 8.10 (s, 1H), 8.20 (d, 1H), 9.23 (br s, 1H), 12.62 (s, 1H).

Phenyl {4-[(3-chloro-1-{[2-(trimethylsilyl)ethoxy]methyl}-1H-pyrrolo[2,3-b]pyridin-4-yl)oxy]-3,5-difluorophenyl}carbamate (S27)

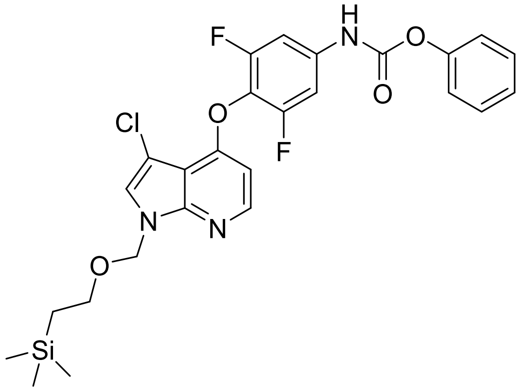

To solution of 4-[(3-chloro-1-{[2-(trimethylsilyl)ethoxy]methyl}-1H-pyrrolo[2,3-b]pyridin-4-yl)oxy]-3,5-difluoroaniline (1.20 g, 2.82 mmol, **S20**) in ethyl acetate (20 mL) and saturated sodium bicarbonate solution (10 mL) was slowly added at room temperature phenyl carbonochloridate (441 mg, 2.82 mmol). After stirring this mixture 5 hours, the mixture was diluted with water. The organic phase was washed with brine, dried over sodium sulfate, filtered and concentrated to dryness. The resulting residue was purified via a Biotage chromatography system (KP-Sil snap column; hexane / ethyl acetate gradient with up to 35% ethyl acetate) to obtain 1.37 g (89 % yield) of the desired title compound.

Method 2, UPLC-MS (ESI+): t_R = 1.65 min; *m/z* calcd for $C_{26}H_{27}ClF_2N_3O_4Si$ $[M + H]^+$: 546.1; found: 546.0

^{1}H-NMR (400 MHz, $CDCl_3$) δ [ppm]: -0.05 (s, 9H), 0.89 - 0.95 (m, 2H), 3.52 - 3.58 (m, 2H), 5.63 (s, 2H), 6.31 (d, 1H), 7.12 (bs, 1H); 7.17-7.22 (m, 2H); 7.23-7.31 (m, 4H + $CHCl_3$); 7.40-7.45 (m, 2H); 8.14 (d, 1H).

1-{4-[(3-chloro-1-{[2-(trimethylsilyl)ethoxy]methyl}-1H-pyrrolo[2,3-b]pyridin-4-yl)oxy]-3,5-difluorophenyl}-3-[(3-methyloxetan-3-yl)methyl]urea (S28)

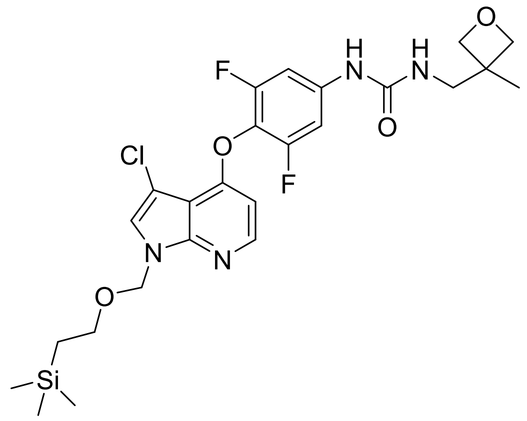

To solution of phenyl {4-[(3-chloro-1-{[2-(trimethylsilyl)ethoxy]methyl}-1H-pyrrolo[2,3-b]pyridin-4-yl)oxy]-3,5-difluorophenyl}carbamate (150 mg, 275 µmol, **S27**) in DMF (4.0mL) was added 1-(3-methyloxetan-3-yl)methanamine (55.6 mg, 549 µmol, CAS No. [153209-97-3]). The resulting mixture was stirred at 60°C for 16 hours, at which time the reaction mixture was cooled to room temperature, diluted with ethyl acetate and water was added. The layers were separated, and the aqueous phase was extracted two times with ethyl acetate. The combined organic phases were washed with brine, dried over sodium sulfate, filtered and concentrated to dryness to give crude 1-{4-[(3-chloro-1-{[2-(trimethylsilyl)ethoxy]methyl}-1H-pyrrolo[2,3-b]pyridin-4-yl)oxy]-3,5-difluorophenyl}-3-[(3-methyloxetan-3-yl)methyl]urea, which was used in the subsequent reaction without further purification.

Method 1, UPLC-MS (ESI+): t_R = 1.47 min; m/z calcd for $C_{25}H_{32}ClF_2N_4O_4Si$ $[M + H]^+$: 553.2; found: 553.2

Compound 13

(+/-)-[2-({4-[(3-chloro-1H-pyrrolo[2,3-b]pyridin-4-yl)oxy]-3,5-difluorophenyl}amino)-5-methyl-5,6-dihydro-4H-1,3-oxazin-5-yl]methanol

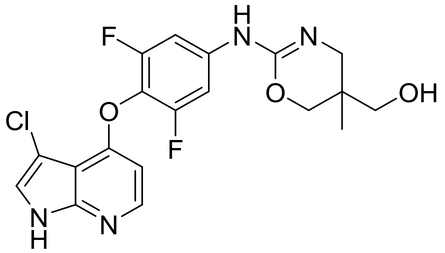

To a solution of 1-{4-[(3-chloro-1-{[2-(trimethylsilyl)ethoxy]methyl}-1H-pyrrolo[2,3-b]pyridin-4-yl)oxy]-3,5-difluorophenyl}-3-[(3-methyloxetan-3-yl)methyl]urea (150 mg, 0.27 mmol, **S28**) in dichloromethane (5.0 mL) was added trifluoroacetic acid (1.0 mL, 13 mmol). The resulting mixture was stirred at room temperature for 16 hours. 2M NaOH was added, and the mixture extracted two times with ethyl acetate. The combined organic layers were washed with water, brine, dried over sodium sulfate, filtered and evaporated to afford the crude oxazine. The crude product was purified by preparative HPLC to afford (+/-)-[2-({4-[(3-chloro-1H-pyrrolo[2,3-b]pyridin-4-yl)oxy]-3,5-difluorophenyl}amino)-5-methyl-5,6-dihydro-4H-1,3-oxazin-5-yl]methanol (55 mg, 48% over two steps).

Method 1, UPLC-MS (ESI+): t_R = 0.98 min; *m/z* calcd for $C_{19}H_{18}ClF_2N_4O_3$ $[M + H]^+$: 423.1; found: 423.1

1H NMR (500 MHz, DMSO-d6, measured at 80°C) δ ppm 0.94 (s, 3 H), 3.01 (br d, 1 H), 3.24 (br d, 1 H), 3.30 (dd, 1 H), 3.36 (dd, 1 H), 3.88 (d, 1 H), 4.08 (dd, 1 H), 4.59 (br s, 1 H), 6.31 (d, 1 H), 7.36 (br s, 1 H), 7.50 (s, 1 H), 8.09 (d, 1 H), 8.72 (br s, 1 H), 11.85 (br s, 1 H)

O-phenyl {4-[(3-chloro-1-{[2-(trimethylsilyl)ethoxy]methyl}-1H-pyrrolo[2,3-b]pyridin-4-yl)oxy]-3,5-difluorophenyl}carbamothioate (S29)

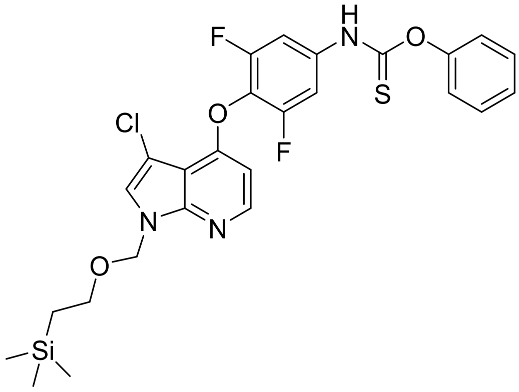

4-[(3-chloro-1-{[2-(trimethylsilyl)ethoxy]methyl}-1H-pyrrolo[2,3-b]pyridin-4-yl)oxy]-3,5-difluoroaniline (600 mg, 1.41 mmol, **S20**) was dissolved in THF (9.0 mL) and pyridine (750 μL) and cooled down to 0°C. O-phenyl carbonochloridothioate (CAS No: 1005-56-7, 210 μL, 1.5 mmol) was added dropwise and the mixture allowed to warm up to room temperature for 2h. The solvent was removed under vacuum and the crude product used in the following transformation without purification.
Method 2, UPLC-MS (ESI+): t_R = 1.68 min; *m/z* calcd for $C_{26}H_{27}ClF_2N_3O_3SSi$ $[M + H]^+$: 562.1; found: 562.3

3-(methoxymethyl)-3-methyloxetane (S30)

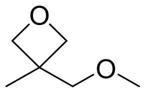

(3-methyloxetan-3-yl)methanol (CAS No: 3143-02-0, 2.0 mL, 20 mmol) was dissolved in THF (80 mL, 1.0 mol), put under argon and cooled down to 0°C. Sodium hydride (862 mg, 60 % purity, 21.5 mmol) was added portion-wise, and the resulting suspension was stirred for 30 min. iodomethane (1.3 mL, 21 mmol) was added dropwise, the ice batch was removed and the temperature was allowed to rise over 2h. The reaction was quenched with water, and extracted with hexanes (x2). The organic layers were dried over sodium sulfate, filtered, and the solvent removed under vacuum to yield the title compound (2.10 g, 83 % yield).
^{1}H-NMR (400 MHz, CHLOROFORM-d) δ [ppm]: 1.32 (s, 3H), 3.46 (s, 2H), 4.37 (d, 2H), 4.52 (d, 2H).

(+/-)-2-[(benzylamino)methyl]-3-methoxy-2-methylpropan-1-ol (S31)

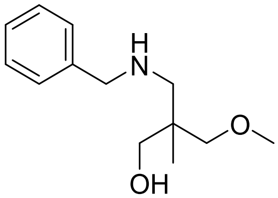

3-(methoxymethyl)-3-methyloxetane (2.10 g, 16.3 mmol, **S30**) and benzylamine (5.3 mL, 49 mmol) were dissolved in acetonitrile (63 mL), and zirconium (IV) chloride (3.79 g, 16.3 mmol) was added. The reaction was stirred at room temperature for 3h, filtered and concentrated under vacuum. The residue was filtered through silica to yield the title compound (513 mg, 14 % yield).
^{1}H-NMR (400 MHz, DMSO-d6) δ [ppm]: 0.78 (s, 3H), 2.36 (d, 2H), 3.16 (d, 2H), 3.20 (s, 3H), 3.25 (d, 2H), 3.66 (s, 2H), 7.31 (m, 5H).

(+/-)-2-(aminomethyl)-3-methoxy-2-methylpropan-1-ol (S32)

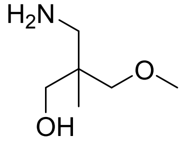

(+/-)-2-[(benzylamino)methyl]-3-methoxy-2-methylpropan-1-ol (510 mg, 2.28 mmol, **S31**) was dissolved in DMF (5.3 mL), and palladium on charcoal (48.6 mg, 10% purity, 457 µmol) was added. The reaction was put under a hydrogen atmosphere (1 atm) and stirred at room temperature for 100h. The crude was filtered over a celite pad and used as a DMF solution in the following transformation.

(+/-)-N-{4-[(3-chloro-1-{[2-(trimethylsilyl)ethoxy]methyl}-1H-pyrrolo[2,3-b]pyridin-4-yl)oxy]-3,5-difluorophenyl}-N'-[2-(hydroxymethyl)-3-methoxy-2-methylpropyl]thiourea (S33)

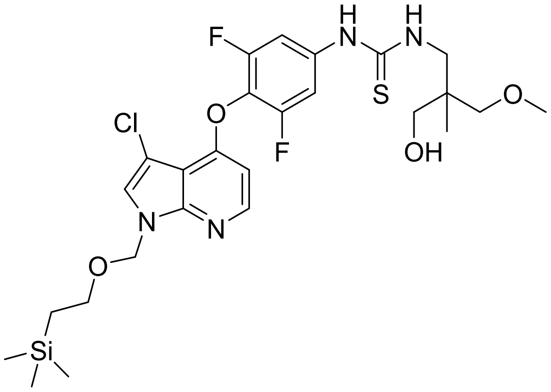

In analogy to intermediate **S**35, O-phenyl {4-[(3-chloro-1-{[2-(trimethylsilyl)ethoxy]methyl}-1H-pyrrolo[2,3-b]pyridin-4-yl)oxy]-3,5-difluorophenyl}carbamothioate (250 mg, 311 µmol, **S29**) and 2-(aminomethyl)-3-methoxy-2-methylpropan-1-ol (1.8 mL, 0.21 M, 370 µmol, **S32**) were stirred in DMF (4.0 mL) at 60°C to yield the title compound (143 mg, 73 % yield), which was filtered over silica and used directly in the next transformation.

Method 1, UPLC-MS (ESI+): t_R = 1.51 min; *m/z* calcd for $C_{26}H_{36}ClF_2N_4O_4SSi$ $[M + H]^+$: 601.2; found: 601.3

(+/-)-N-{4-[(3-chloro-1-{[2-(trimethylsilyl)ethoxy]methyl}-1H-pyrrolo[2,3-b]pyridin-4-yl)oxy]-3,5-difluorophenyl}-5-(methoxymethyl)-5-methyl-5,6-dihydro-4H-1,3-oxazin-2-amine (S34)

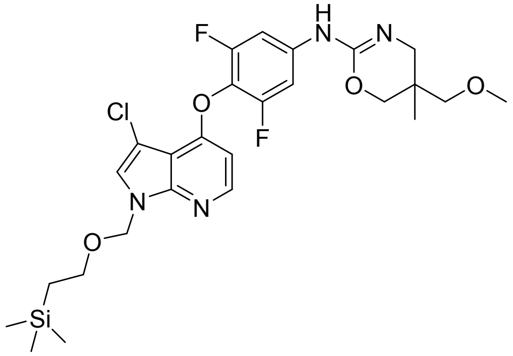

(+/-)-N-{4-[(3-chloro-1-{[2-(trimethylsilyl)ethoxy]methyl}-1H-pyrrolo[2,3-b]pyridin-4-yl)oxy]-3,5-difluorophenyl}-N'-[2-(hydroxymethyl)-3-methoxy-2-methylpropyl]thiourea (140 mg, 210 µmol, **S33**) was dissolved in tetrahydrofuran (1.0 mL), and 1-(3-Dimethylaminopropyl)-3-ethylcarbodiimide hydrochloride (44.2 mg, 231 µmol) was added. The reaction was stirred at 60°C overnight. The solvent was then evaporated, and the crude filtered over silica. The raw product was used directly in the following transformation.

Method 1, UPLC-MS (ESI+): t_R = 1.60 min; *m*/*z* calcd for $C_{26}H_{34}ClF_2N_4O_4Si$ $[M + H]^+$: 567.2; found: 567.5

<u>Compound 14</u>

(+/-)-N-{4-[(3-chloro-1H-pyrrolo[2,3-b]pyridin-4-yl)oxy]-3,5-difluorophenyl}-5-(methoxymethyl)-5-methyl-5,6-dihydro-4H-1,3-oxazin-2-amine

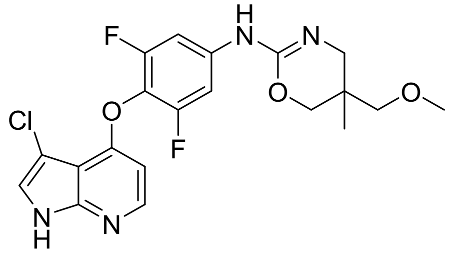

(+/-)-N-{4-[(3-chloro-1-{[2-(trimethylsilyl)ethoxy]methyl}-1H-pyrrolo[2,3-b]pyridin-4-yl)oxy]-3,5-difluorophenyl}-5-(methoxymethyl)-5-methyl-5,6-dihydro-4H-1,3-oxazin-2-amine (81.0 mg, 100 µmol, **S34**) was dissolved in dichloromethane (3.2 mL) and trifluoroacetic acid (330 µL, 4.3 mmol) was added. The mixture was stirred under argon overnight at room temperature. The solvent was removed under vacuum, and the residue stirred with acetonitrile (3 mL) and a 33% ammonia solution (1,5 mL) at room temperature for 1h and

then dried again. The residue was dissolved with ethyl acetate and washed with water (x2) and brine. The organic residue was dried with sodium sulfate, filtered and evaporated. The product was purified by preparative TLC (dichloromethane:methanol, 90:10) followed by preparative HPLC to yield the title compound (24.1 mg, 50 % yield).

Method 1, UPLC-MS (ESI+): t_R = 1.15 min; *m/z* calcd for $C_{20}H_{20}ClF_2N_4O_3$ [M + H]$^+$: 437.1; found: 437.3

^{1}H-NMR (400 MHz, DMSO-d6) δ [ppm]: 0.93 (s, 3H), 3.06 (m, 1H), 3.17-3.27 (m, 3H), 3.28 (2, 3H), 3.87 (m, 1H), 4.06 (m, 1H), 6.30 (d, 1H), 7.55 (br s, 2H), 7.60 (s, 1H), 8.08 (d, 1H), 9.09 (br s, 1H), 12.08 (d, 1H).

N-{4-[(3-chloro-1-{[2-(trimethylsilyl)ethoxy]methyl}-1H-pyrrolo[2,3-b]pyridin-4-yl)oxy]-3,5-difluorophenyl}-N'-(3-hydroxypropyl)thiourea (S35)

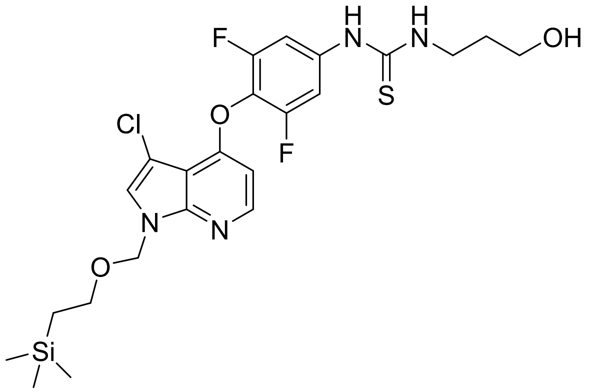

To a stirred solution of O-phenyl {4-[(3-chloro-1-{[2-(trimethylsilyl)ethoxy]methyl}-1H-pyrrolo[2,3-b]pyridin-4-yl)oxy]-3,5-difluorophenyl}carbamothioate (600 mg, 1.07 mmol, **S29**) in DMF (7.0 mL) was added 3-aminopropan-1-ol (160 mg, 2.13 mmol, CAS No. [156-87-6]). The resulting mixture was heated to 60°C for 2h at which time water and ethyl acetate were added and the layers were separated. The aqueous phase was extracted twice with ethyl acetate and the combined organic layers were washed with brine, dried over sodium sulfate, filtered and evaporated to give the crude product which was used without further purification.

Method 1, UPLC-MS (ESI+): t_R = 1.43 min; *m/z* calcd for $C_{23}H_{30}ClF_2N_4O_3SSi$ [M + H]$^+$: 543.1; found: 543.5

N-{4-[(3-chloro-1-{[2-(trimethylsilyl)ethoxy]methyl}-1H-pyrrolo[2,3-b]pyridin-4-yl)oxy]-3,5-difluorophenyl}-5,6-dihydro-4H-1,3-oxazin-2-amine (S36)

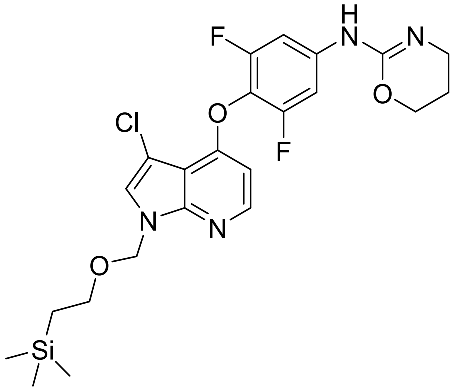

To a solution of N-{4-[(3-chloro-1-{[2-(trimethylsilyl)ethoxy]methyl}-1H-pyrrolo[2,3-b]pyridin-4-yl)oxy]-3,5-difluorophenyl}-N'-(3-hydroxypropyl)thiourea (290 mg, 534 µmol, **S35**) in acetonitrile (25 mL, 470 mmol) was added 1-(3-Dimethylaminopropyl)-3-ethylcarbodiimide hydrochloride (205 mg, 1.07 mmol) and triethylamine (220 µL, 1.6 mmol). The resulting mixture was stirred at 40°C overnight at which time water and ethyl acetate were added and the layers separated. The aqueous phase was extracted twice with ethyl acetate and the combined organic layers were washed with brine, dried over sodium sulfate, filtered and evaporated to afford the crude product which was used without further purification.

Method 1, UPLC-MS (ESI+): t_R = 1.52 min; *m/z* calcd for $C_{23}H_{28}ClF_2N_4O_3Si$ $[M + H]^+$: 509.2; found: 509.5

Compound 15

N-{4-[(3-chloro-1H-pyrrolo[2,3-b]pyridin-4-yl)oxy]-3,5-difluorophenyl}-5,6-dihydro-4H-1,3-oxazin-2-amine

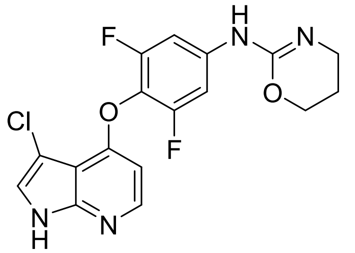

To a stirred solution of N-{4-[(3-chloro-1-{[2-(trimethylsilyl)ethoxy]methyl}-1H-pyrrolo[2,3-b]pyridin-4-yl)oxy]-3,5-difluorophenyl}-5,6-dihydro-4H-1,3-oxazin-2-amine (270 mg, 530 µmol, **S36**) in dichloromethane (4.0 mL) was added trifluoroacetic acid (4.0 mL, 52 mmol). The mixture was stirred at room temperature overnight, at which time the mixture was basified

with a 2M aqueous solution of sodium hydroxide. Ethyl acetate was added and the layers were separated. The aqueous phase was extracted twice with ethyl acetate, and the combined organic layers were washed with brine, dried with sodium sulfate, filtered and evaporated to afford the crude product. The crude material was purified by preparative HPLC to afford the title compound (24 mg, 12 % yield)

Method 1, UPLC-MS (ESI+): t_R = 1.02 min; *m/z* calcd for $C_{17}H_{14}ClF_2N_4O_2$ $[M + H]^+$: 379.1; found: 379.4

^{1}H NMR (400 MHz, DMSO-d_6) δ ppm 1.86 (br s, 2 H), 4.26 (t, 2 H), 6.29 (d, 1 H), 7.59 (s, 1 H), 8.09 (d, 1 H), 8.97 (br s, 1 H), 11.72 - 12.29 (m, 1 H)

N-{4-[(3-chloro-1-{[2-(trimethylsilyl)ethoxy]methyl}-1H-pyrrolo[2,3-b]pyridin-4-yl)oxy]-3,5-difluorophenyl}-N'-(3-hydroxy-2,2-dimethylpropyl)thiourea (S37)

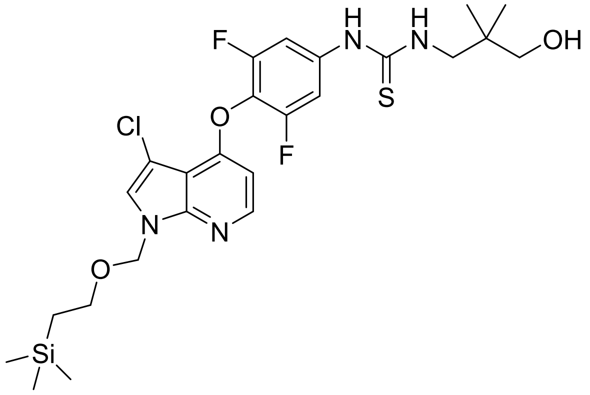

In analogy to intermediate **S35**, O-phenyl {4-[(3-chloro-1-{[2-(trimethylsilyl)ethoxy]methyl}-1H-pyrrolo[2,3-b]pyridin-4-yl)oxy]-3,5-difluorophenyl}carbamothioate (300 mg, 534 µmol, **S29**), and 3-amino-2,2-dimethylpropan-1-ol (116 mg, 1.07 mmol, CAS No. [141-43-5]), in DMF (5.0 mL) were reacted to obtain a crude product which was used in the next step without further purification.

Method 1, UPLC-MS (ESI+): t_R = 1.53 min; *m/z* calcd for $C_{25}H_{34}ClF_2N_4O_3SSi$ $[M + H]^+$: 571.2; found: 571.5

N-{4-[(3-chloro-1-{[2-(trimethylsilyl)ethoxy]methyl}-1H-pyrrolo[2,3-b]pyridin-4-yl)oxy]-3,5-difluorophenyl}-5,5-dimethyl-5,6-dihydro-4H-1,3-oxazin-2-amine (S38)

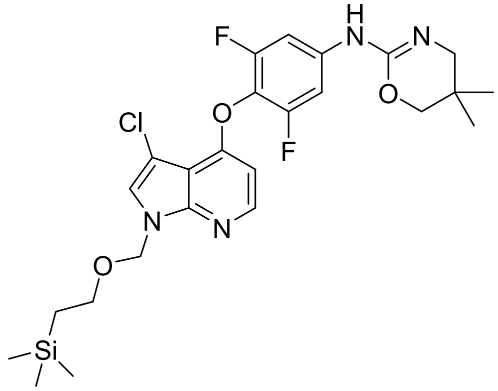

In analogy to Intermediate **S36**, N-{4-[(3-chloro-1-{[2-(trimethylsilyl)ethoxy]methyl}-1H-pyrrolo[2,3-b]pyridin-4-yl)oxy]-3,5-difluorophenyl}-N'-(3-hydroxy-2,2-dimethylpropyl)thiourea (300 mg, 525 µmol, **S37**) was reacted with 1-(3-Dimethylaminopropyl)-3-ethylcarbodiimide hydrochloride (201 mg, 1.05 mmol) and triethylamine (220 µL, 1.6 mmol) in acetonitrile (10 mL) to obtain a crude product which was used in the next step without further purification.

Method 1, UPLC-MS (ESI+): t_R = 1.61 min; *m/z* calcd for $C_{25}H_{32}ClF_2N_4O_3Si$ $[M + H]^+$: 537.2; found: 537.6

Compound 16

N-{4-[(3-chloro-1H-pyrrolo[2,3-b]pyridin-4-yl)oxy]-3,5-difluorophenyl}-5,5-dimethyl-5,6-dihydro-4H-1,3-oxazin-2-amine

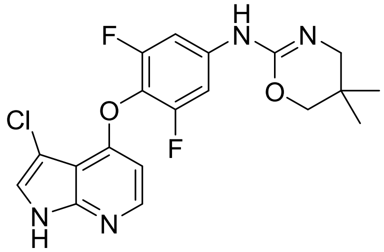

In analogy to compound **2**, N-{4-[(3-chloro-1-{[2-(trimethylsilyl)ethoxy]methyl}-1H-pyrrolo[2,3-b]pyridin-4-yl)oxy]-3,5-difluorophenyl}-5,5-dimethyl-5,6-dihydro-4H-1,3-oxazin-2-amine (280 mg, 521 µmol, **S38** was treated with trifluoroacetic acid (4.0 mL, 52 mmol) in dichloromethane (4.0 mL, 62 mmol) to afford after preparative HPLC purification the title compound (33 mg, 15 % yield).

Method 1, UPLC-MS (ESI+): t_R = 1.16 min; *m/z* calcd for $C_{19}H_{18}ClF_2N_4O_2$ $[M + H]^+$: 407.1; found: 407.5

^1H NMR (400 MHz, DMSO-d_6) δ ppm 0.96 (s, 6 H), 3.08 (br s, 2 H), 3.88 (s, 2 H), 6.30 (d, 1 H), 7.60 (s, 1 H), 8.08 (d, 1 H), 9.04 (br s, 1 H), 12.11 (br s, 1 H)

N-{4-[(3-chloro-1-{[2-(trimethylsilyl)ethoxy]methyl}-1H-pyrrolo[2,3-b]pyridin-4-yl)oxy]-3,5-difluorophenyl}-N'-(2-hydroxyethyl)thiourea (S39)

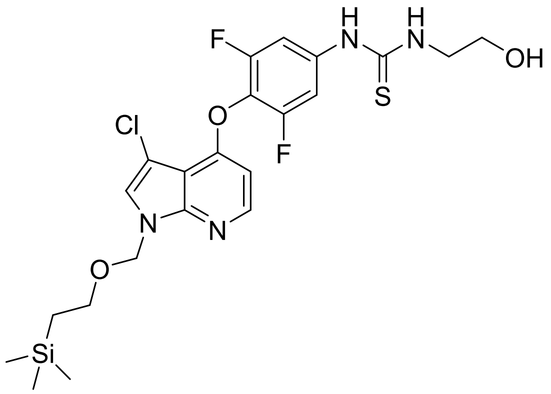

In analogy to **S35**, O-phenyl {4-[(3-chloro-1-{[2-(trimethylsilyl)ethoxy]methyl}-1H-pyrrolo[2,3-b]pyridin-4-yl)oxy]-3,5-difluorophenyl}carbamothioate (600 mg, 1.07 mmol, **S29**) and 2-aminoethan-1-ol (130 µL, 2.1 mmol, CAS No. [141-43-5], in DMF (7.0 mL) were reacted to obtain a crude product which was used in the next step without further purification.
Method 1, UPLC-MS (ESI+): t_R = 1.42 min; *m/z* calcd for $C_{22}H_{28}ClF_2N_4O_3SSi$ $[M + H]^+$: 529.1; found: 529.5

N-{4-[(3-chloro-1-{[2-(trimethylsilyl)ethoxy]methyl}-1H-pyrrolo[2,3-b]pyridin-4-yl)oxy]-3,5-difluorophenyl}-4,5-dihydro-1,3-oxazol-2-amine (S40)

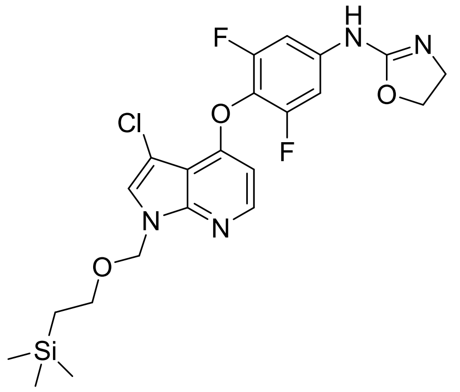

In analogy to **S36**, N-{4-[(3-chloro-1-{[2-(trimethylsilyl)ethoxy]methyl}-1H-pyrrolo[2,3-b]pyridin-4-yl)oxy]-3,5-difluorophenyl}-N'-(2-hydroxyethyl)thiourea (280 mg, 529 µmol, **S39**) was reacted with 1-(3-Dimethylaminopropyl)-3-ethylcarbodiimide hydrochloride (203 mg, 1.06 mmol) and triethylamine (220 µL, 1.6 mmol) in acetonitrile (25 mLl) to obtain a crude product which was used in the next step without further purification.
Method 1, UPLC-MS (ESI+): t_R = 1.48 min; *m*/*z* calcd for $C_{22}H_{26}ClF_2N_4O_3Si$ $[M + H]^+$: 495.1; found: 495.5

Compound 17

N-{4-[(3-chloro-1H-pyrrolo[2,3-b]pyridin-4-yl)oxy]-3,5-difluorophenyl}-4,5-dihydro-1,3-oxazol-2-amine

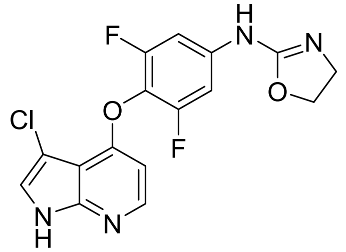

In analogy to compound **2**, N-{4-[(3-chloro-1-{[2-(trimethylsilyl)ethoxy]methyl}-1H-pyrrolo[2,3-b]pyridin-4-yl)oxy]-3,5-difluorophenyl}-4,5-dihydro-1,3-oxazol-2-amine (260 mg, 525 µmol) was treated with trifluoroacetic acid (4.0 mL, 52 mmol) in dichloromethane (4.0 mL) to afford after preparative HPLC purification the title compound(49 mg, 25 % yield).
Method 1, UPLC-MS (ESI+): t_R = 0.98 min; *m*/*z* calcd for $C_{16}H_{12}ClF_2N_4O_2$ $[M + H]^+$: 365.1; found: 365.5

^{1}H NMR (400 MHz, DMSO-*d*$_6$) δ ppm 3.71 (br s, 2 H), 4.35 (br s, 2 H), 6.31 (d, 1 H), 7.60 (s, 1 H), 8.10 (d, 1 H), 12.11 (br s, 1 H)

N-{4-[(3-chloro-1-{[2-(trimethylsilyl)ethoxy]methyl}-1H-pyrrolo[2,3-b]pyridin-4-yl)oxy]-3,5-difluorophenyl}-5,6-dihydro-4H-1,3-thiazin-2-amine (S41)

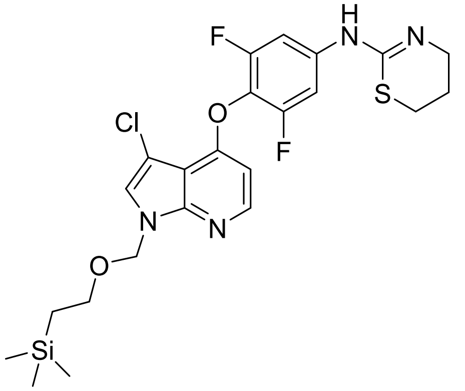

To a stirred solution of N-{4-[(3-chloro-1-{[2-(trimethylsilyl)ethoxy]methyl}-1H-pyrrolo[2,3-b]pyridin-4-yl)oxy]-3,5-difluorophenyl}-N'-(3-hydroxypropyl)thiourea (290 mg, 534 µmol, **S35**) in THF (25 mL) was added 1,1'-Carbonyldiimidazole (173 mg, 1.07 mmol). The resulting mixture was stirred at 70°C overnight at which time the solvent was evaporated to give a crude product which was used without further purification.

Method 1, UPLC-MS (ESI+): t_R = 1.59 min; *m/z* calcd for $C_{23}H_{28}ClF_2N_4O_2SSi[M + H]^+$: 525.1; found: 525.5

<u>Compound 18</u>

N-{4-[(3-chloro-1H-pyrrolo[2,3-b]pyridin-4-yl)oxy]-3,5-difluorophenyl}-5,6-dihydro-4H-1,3-thiazin-2-amine

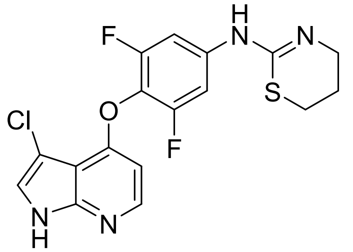

In analogy to compound **2,** N-{4-[(3-chloro-1-{[2-(trimethylsilyl)ethoxy]methyl}-1H-pyrrolo[2,3-b]pyridin-4-yl)oxy]-3,5-difluorophenyl}-5,6-dihydro-4H-1,3-thiazin-2-amine (280 mg, 533 µmol, **S41**) was treated with trifluoroacetic acid (4.0 mL, 52 mmol) in dichloromethane (4.0 mL) to afford after preparative HPLC purification the title compound (16 mg, 8 % yield).

Method 1, UPLC-MS (ESI+): t_R = 1.12 min; *m/z* calcd for $C_{17}H_{14}ClF_2N_4OS$ $[M + H]^+$: 395.1; found: 395.4

^{1}H NMR (400 MHz, DMSO-d_6) δ ppm 1.83 (br s, 2 H), 3.10 (br t, 2 H), 3.51 (br s, 2 H), 6.25 (d, 1 H), 7.57 (s, 1 H), 8.06 (d, 1 H)

1-{4-[(3-chloro-1-{[2-(trimethylsilyl)ethoxy]methyl}-1H-pyrrolo[2,3-b]pyridin-4-yl)oxy]-3,5-difluorophenyl}-3-[(3-fluorooxetan-3-yl)methyl]urea (S42)

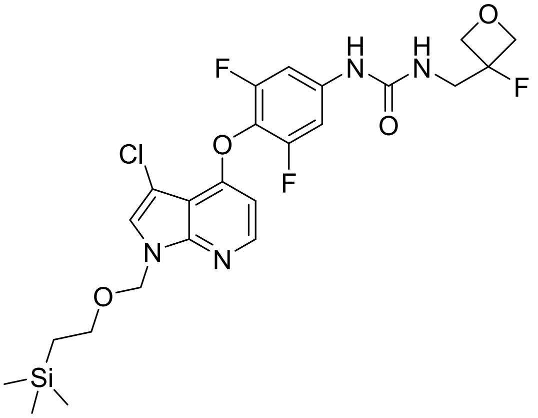

To solution of phenyl {4-[(3-chloro-1-{[2-(trimethylsilyl)ethoxy]methyl}-1H-pyrrolo[2,3-b]pyridin-4-yl)oxy]-3,5-difluorophenyl}carbamate (150 mg, 275 µmol, **S27**) in DMF (4.0mL) was added 1-(3-fluorooxetan-3-yl)methanamine (57.7 mg, 549 µmol, CAS No. [883311-82-8]). The resulting mixture was stirred at 60°C for 16 hours, at which time the reaction mixture was cooled to room temperature, diluted with ethyl acetate and water was added. The layers were separated, and the aqueous phase was extracted two times with ethyl acetate. The combined organic phases were washed with brine, dried over sodium sulfate, filtered and concentrated to dryness to give crude 1-{4-[(3-chloro-1-{[2-(trimethylsilyl)ethoxy]methyl}-1H-pyrrolo[2,3-b]pyridin-4-yl)oxy]-3,5-difluorophenyl}-3-[(3-fluorooxetan-3-yl)methyl]urea, which was used in the subsequent reaction without further purification.

Method 1, UPLC-MS (ESI+): t_R = 1.47 min; *m/z* calcd for $C_{24}H_{29}ClF_3N_4O_4Si$ $[M + H]^+$: 557.2; found: 557.4

Compound 19

(+/-)-[2-({4-[(3-chloro-1H-pyrrolo[2,3-b]pyridin-4-yl)oxy]-3,5-difluorophenyl}amino)-5-fluoro-5,6-dihydro-4H-1,3-oxazin-5-yl]methanol

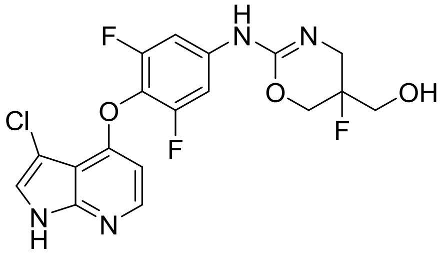

To a solution of 1-{4-[(3-chloro-1-{[2-(trimethylsilyl)ethoxy]methyl}-1H-pyrrolo[2,3-b]pyridin-4-yl)oxy]-3,5-difluorophenyl}-3-[(3-fluorooxetan-3-yl)methyl]urea (150 mg, 0.27 mmol, **S42**) in dichloromethane (5.0 mL) was added trifluoroacetic acid (1.0 mL, 13 mmol). The resulting mixture was stirred at room temperature for 16 hours. 2M NaOH was added, and the mixture extracted two times with ethyl acetate. The combined organic layers were washed with water, brine, dried over sodium sulfate, filtered and evaporated to afford the crude oxazine. The crude product was purified by preparative HPLC to afford (+/-)-[2-({4-[(3-chloro-1H-pyrrolo[2,3-b]pyridin-4-yl)oxy]-3,5-difluorophenyl}amino)-5-fluoro-5,6-dihydro-4H-1,3-oxazin-5-yl]methanol (40 mg, 34% over two steps).

Method 1, UPLC-MS (ESI+): t_R = 0.97 min; *m/z* calcd for $C_{18}H_{15}ClF_3N_4O_3$ $[M + H]^+$: 427.1; found: 427.3

^1H NMR (400 MHz, DMSO-d_6) δ ppm 3.38 - 3.67 (m, 4 H), 4.22 - 4.37 (m, 2 H), 5.25 (br t, 1 H), 6.31 (d, 1 H), 7.53 - 7.71 (m, 3 H), 8.09 (d, 1 H), 9.23 (br s, 1 H), 12.11 (br s, 1 H)

N-{4-[(3-chloro-1-{[2-(trimethylsilyl)ethoxy]methyl}-1H-pyrrolo[2,3-b]pyridin-4-yl)oxy]-3,5-difluorophenyl}-N'-(2,2-difluoro-3-hydroxypropyl)thiourea (S43)

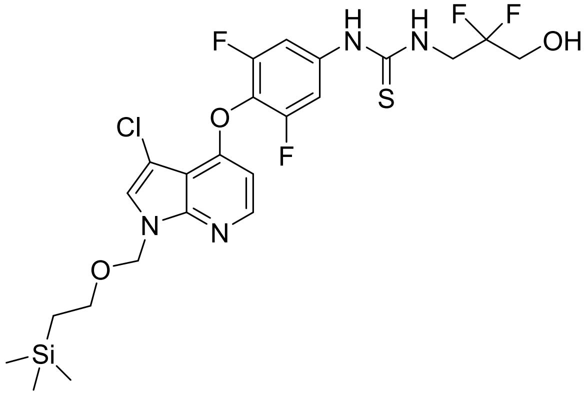

In analogy to **S35**, O-phenyl {4-[(3-chloro-1-{[2-(trimethylsilyl)ethoxy]methyl}-1H-pyrrolo[2,3-b]pyridin-4-yl)oxy]-3,5-difluorophenyl}carbamothioate (370 mg, 658 µmol, **S29**), and 3-amino-2,2-difluoropropan-1-ol (146 mg, 1.32 mmol, CAS No. [2867-59-6]), in DMF (3.0 mL) were reacted to obtain a crude product which was used in the next step without further purification.

Method 1, UPLC-MS (ESI+): t_R = 1.43 min; *m/z* calcd for $C_{23}H_{28}ClF_4N_4O_3SSi$ $[M + H]^+$: 579.1; found: 579.4

N-{4-[(3-chloro-1-{[2-(trimethylsilyl)ethoxy]methyl}-1H-pyrrolo[2,3-b]pyridin-4-yl)oxy]-3,5-difluorophenyl}-5,5-difluoro-5,6-dihydro-4H-1,3-oxazin-2-amine (S44)

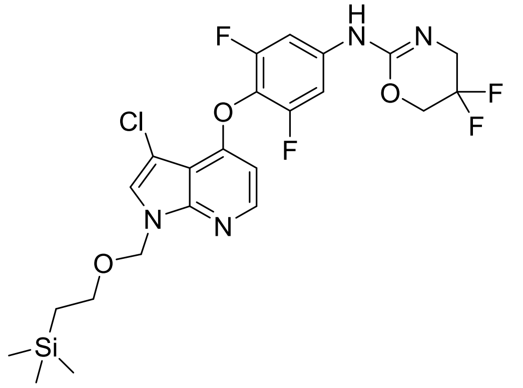

In analogy to **S36**, N-{4-[(3-chloro-1-{[2-(trimethylsilyl)ethoxy]methyl}-1H-pyrrolo[2,3-b]pyridin-4-yl)oxy]-3,5-difluorophenyl}-N'-(2,2-difluoro-3-hydroxypropyl)thiourea (380 mg, 656 µmol, **S43**) was reacted with 1-(3-Dimethylaminopropyl)-3-ethylcarbodiimide hydrochloride (252 mg, 1.31 mmol) and triethylamine (270 µL, 2.0 mmol) in acetonitrile (10 mL) to obtain a crude product which was used in the next step without further purification.

Method 1, UPLC-MS (ESI+): t_R = 1.58 min; *m/z* calcd for $C_{23}H_{26}ClF_4N_4O_3Si$ $[M + H]^+$: 545.1; found: 545.4

<u>Compound 20</u>

N-{4-[(3-chloro-1H-pyrrolo[2,3-b]pyridin-4-yl)oxy]-3,5-difluorophenyl}-5,5-difluoro-5,6-dihydro-4H-1,3-oxazin-2-amine

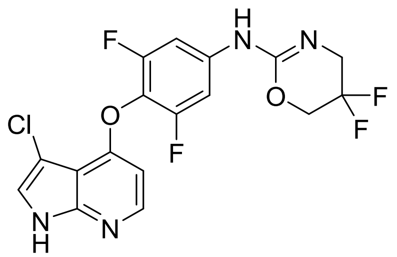

In analogy to compound **2**, N-{4-[(3-chloro-1-{[2-(trimethylsilyl)ethoxy]methyl}-1H-pyrrolo[2,3-b]pyridin-4-yl)oxy]-3,5-difluorophenyl}-5,5-difluoro-5,6-dihydro-4H-1,3-oxazin-2-amine (350 mg, 642 µmol) was treated with trifluoroacetic acid (4.0 mL, 52 mmol) in dichloromethane (4.0 mL) to afford after preparative HPLC purification the title compound (24 mg, 9 % yield).

Method 1, UPLC-MS (ESI+): t_R = 1.16 min; *m*/*z* calcd for $C_{17}H_{12}ClF_4N_4O_2$ $[M + H]^+$: 415.1; found: 415.3

^1H NMR (400 MHz, DMSO-d_6) δ ppm 3.79 (br t, 2 H), 4.48 (t, 2 H), 6.31 (d, 1 H), 7.57 (br d, 2 H), 7.61 (d, 1 H), 8.09 (d, 1 H), 9.49 (s, 1 H), 12.12 (br s, 1 H)

Phenyl {3,5-difluoro-4-[(1-{[2-(trimethylsilyl)ethoxy]methyl}-1H-pyrrolo[2,3-b]pyridin-4-yl)oxy]phenyl}carbamate (S45)

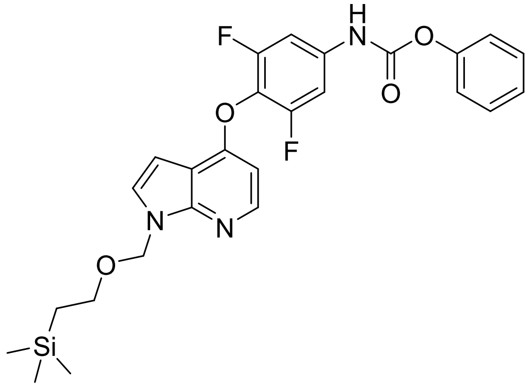

In analogy to **S2**, 3,5-difluoro-4-[(1-{[2-(trimethylsilyl)ethoxy]methyl}-1H-pyrrolo[2,3-b]pyridin-4-yl)oxy]aniline (309 mg, 789 µmol, see *Org. Process Res. Dev.* **2010,** page 168–173) was reacted with phenyl carbonochloridate (110 µL, 870 µmol) in pyridine (370 µL) and THF (5.6 mL). After purification using a Biotage chromatography system we obtained 410 mg (91 % purity, 93 % yield) of the desired title compound.

Method 1, UPLC-MS (ESI+): t_R = 1.59 min; *m*/*z* calcd for $C_{26}H_{28}F_2N_3O_4Si$ $[M + H]^+$: 512.2; found: 512.4

^{1}H-NMR (400 MHz, DMSO-d6) δ [ppm]: -0.12 - -0.09 (m, 9H), 0.77 - 0.85 (m, 2H), 3.49 - 3.55 (m, 2H), 5.63 (s, 2H), 6.42 (d, 1H), 6.52 (d, 1H), 7.25 - 7.32 (m, 3H), 7.40 - 7.49 (m, 4H), 7.61 (d, 1H), 8.16 (d, 1H), 10.80 (s, 1H).

N-{3,5-difluoro-4-[(1-{[2-(trimethylsilyl)ethoxy]methyl}-1H-pyrrolo[2,3-b]pyridin-4-yl)oxy]phenyl}-N'-[(3-methyloxetan-3-yl)methyl]urea (S46)

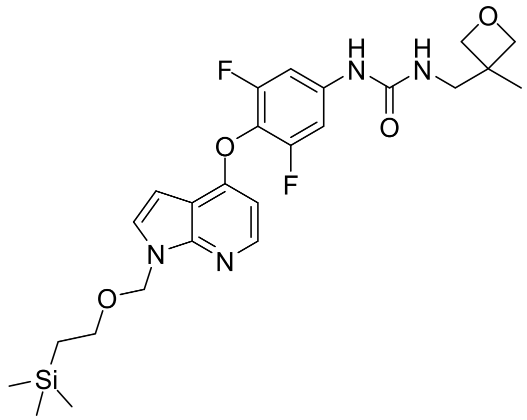

In analogy to **S3**, {3,5-difluoro-4-[(1-{[2-(trimethylsilyl)ethoxy]methyl}-1H-pyrrolo[2,3-b]pyridin-4-yl)oxy]phenyl}carbamate (100 mg, 195 µmol, **S45**) was reacted with 1-(3-methyloxetan-3-yl)methanamine (21.7 mg, 215 µmol) in DMF (1.1 mL). After purification using a Biotage chromatography system we obtained 84.6 mg (100 % purity, 83 % yield) of the desired title compound.

Method 1, UPLC-MS (ESI+): t_R = 1.40 min; *m/z* calcd for $C_{25}H_{33}F_2N_4O_4Si$ $[M + H]^+$: 519.2; found: 519.4

^{1}H-NMR (400 MHz, DMSO-d6) δ [ppm]: -0.12 - -0.08 (m, 9H), 0.77 - 0.85 (m, 2H), 1.23 (s, 3H), 3.28 - 3.32 (m, 2H), 3.48 - 3.54 (m, 2H), 4.21 (d, 2H), 4.38 (d, 2H), 5.62 (s, 2H), 6.42 (d, 1H), 6.49 (d, 1H), 6.71 (t, 1H), 7.35 - 7.42 (m, 2H), 7.59 (d, 1H), 8.14 (d, 1H), 9.01 (s, 1H).

Compound 21

(+/-)-[2-{3,5-difluoro-4-[(1H-pyrrolo[2,3-b]pyridin-4-yl)oxy]anilino}-5-methyl-5,6-dihydro-4H-1,3-oxazin-5-yl]methanol

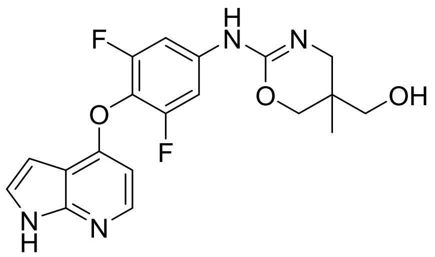

In analogy to compound **2**, N-{3,5-difluoro-4-[(1-{[2-(trimethylsilyl)ethoxy]methyl}-1H-pyrrolo[2,3-b]pyridin-4-yl)oxy]phenyl}-N'-[(3-methyloxetan-3-yl)methyl]urea (81.0 mg, 156 µmol) was stirred with trifluoroacetic acid (1.1 mL, 14 mmol) in dichloromethane (2.2 mL). After purification using a Biotage chromatography system followed by HPLC (method 5) we obtained 24.6 mg (90 % purity, 37 % yield) of the desired title compound.

Method 1, UPLC-MS (ESI+): t_R = 0.88 min; *m/z* calcd for $C_{19}H_{19}F_2N_4O_3$ $[M + H]^+$: 389.1; found: 389.7

^{1}H-NMR (400 MHz, DMSO-d6) δ [ppm]: 0.90 (s, 3H), 3.01 (br d, 1H), 3.14 - 3.31 (m, 3H), 3.87 (br d, 1H), 4.06 (br d, 1H), 4.82 (br s, 1H), 6.29 (br s, 1H), 6.38 (d, 1H), 7.39 (dd, 1H), 7.46 - 7.69 (m, 2H), 8.06 (d, 1H), 9.02 (br s, 1H), 11.81 (br s, 1H).

4-(2,6-difluoro-4-nitrophenoxy)-3-iodo-1-{[2-(trimethylsilyl)ethoxy]methyl}-1H-pyrrolo[2,3-b]pyridine (S47)

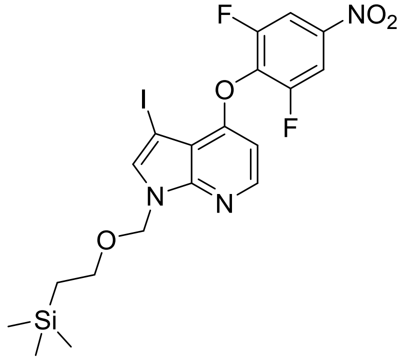

A solution of 4-(2,6-difluoro-4-nitrophenoxy)-1-{[2-(trimethylsilyl)ethoxy]methyl}-1*H*-pyrrolo[2,3-b]pyridine (5.00 g, 11.9 mmol, **S19**) in DMF (100 mL) was treated with 1-iodopyrrolidine-2,5-dione (1.50 eq, 4.00 g, 17.8 mmol) and stirred at room temperature for 2 hours. The reaction mixture was diluted with water and ethyl acetate, the layers were separated and the aqueous layer extracted with ethyl acetate. The combined organic layers were washed with aqueous sat. sodium hydrocarbonate solution and brine, filtrated over a hydrophobic phase separation filter paper and concentrated in vacuo. The obtained material

was purified by flash chromatography (100g SI Snap-colum,hexane/ 0--34 % ethyl acetate) to give the title compound (6.27 g, 92 % yield).

Method 1, UPLC-MS (ESI+): t_R = 1.64 min; *m*/*z* calcd for $C_{19}H_{21}F_2IN_3O_4Si$ $[M + H]^+$: 548.0; found: 548.4

^{1}H-NMR (400 MHz, DMSO-d_6) δ [ppm] = -0.09 (s, 9H), 0.80 – 0.84 (m, 2H), 3.51 – 3.55 (m, 2H), 5.61 (s, 2H), 6.64 (d, 1H), 7.93 (s, 1H), 8.21 (d, 1H), 8.40 – 8.45 (m, 2H).

4-(2,6-difluoro-4-nitrophenoxy)-1-{[2-(trimethylsilyl)ethoxy]methyl}-1H-pyrrolo[2,3-b]pyridine-3-carbonitrile (S48)

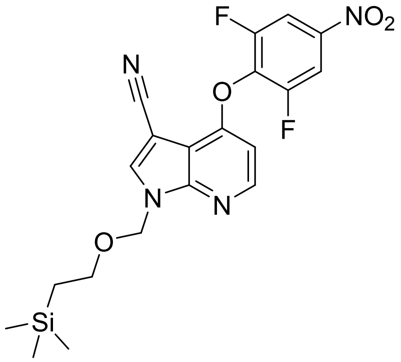

A solution of 4-(2,6-difluoro-4-nitrophenoxy)-3-iodo-1-{[2-(trimethylsilyl)ethoxy]methyl}-1*H*-pyrrolo[2,3-b]pyridine (3.04 g, 5.55 mmol, **S47**) in DMF (15 mL) was treated with copper(I) cyanide (CAS No. [544-92-3]; 1.30 eq., 647 mg, 7.22 mmol) and stirred at 120 C for 20 hours. The reaction mixture was taken up with ethyl acetate and water, the phases separated, and the aqueous phase extracted with ethyl acetate (2x). The combined organic phases were washed with water and brine, dried with sodium sulfate and concentrated in vacuo. The obtained material was purified by flash chromatography (SiO_2-hexane/ ethyl acetate) to give the title compound (2.17 g, 87%).

Method 1, UPLC-MS (ESI+): t_R = 1.50 min; *m*/*z* calcd for $C_{20}H_{21}F_2N_4O_4Si$ $[M + H]^+$: 447.1; found: 447.1

^{1}H-NMR (400 MHz, DMSO-d_6) δ [ppm] = -0.10 (s, 9H), 0.82 – 0.86 (m, 2H), 3.56 – 3.60 (m, 2H), 5.70 (s, 2H), 6.87 (d, 1H), 8.36 (d, 1H), 8.43 – 8.48 (m, 2H), 8.73 (s, 1H).

4-(4-amino-2,6-difluorophenoxy)-1-{[2-(trimethylsilyl)ethoxy]methyl}-1H-pyrrolo[2,3-b]pyridine-3-carbonitrile (S49)

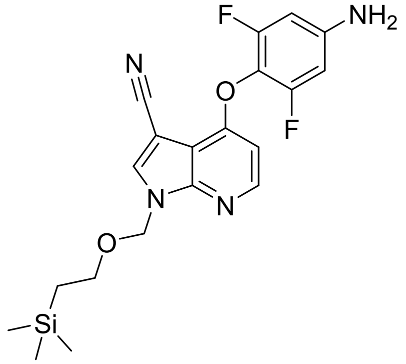

A solution of 4-(2,6-difluoro-4-nitrophenoxy)-1-{[2-(trimethylsilyl)ethoxy]methyl}-1*H*-pyrrolo[2,3-b]pyridine-3-carbonitrile (1.05 g, 2.35 mmol, **S48**) in methanol (15 mL) was treated with tin(II) chloride dihydrate (CAS No. [10025-69-1]; 3.00 eq, 1.59 g, 7.06 mmol) and stirred at 65°C overnight. The reaction mixture was cooled to rt and concentrated in vacuo. The residue was taken up with ethyl acetate and aqueous sat. sodium carbonate solution, filtrated over diatomite and the phases separated. The organic phase was washed with aqueous sat. sodium carbonate solution, dried with sodium sulfate and concentrated in vacuo to give the title compound (630 mg, 61 %) which was not further purified.

Method 1, UPLC-MS (ESI+): t_R = 1.37 min; *m*/*z* calcd for $C_{20}H_{23}F_2N_4O_2Si$ $[M + H]^+$: 417.2; found: 417.5

^{1}H-NMR (400 MHz, DMSO-d_6) δ [ppm] = -0.09 (s, 9H), 0.81 – 0.85 (m, 2H), 3.55 – 3.59 (m, 2H), 5.66 (s, 2H), 5.86 (br s, 2H), 6.38 – 6.44 (m, 2H), 6.57 (d, 1H), 8.29 (d, 1H), 8.63 (s, 1H).

phenyl {4-[(3-cyano-1-{[2-(trimethylsilyl)ethoxy]methyl}-1H-pyrrolo[2,3-b]pyridin-4-yl)oxy]-3,5-difluorophenyl}carbamate (S50)

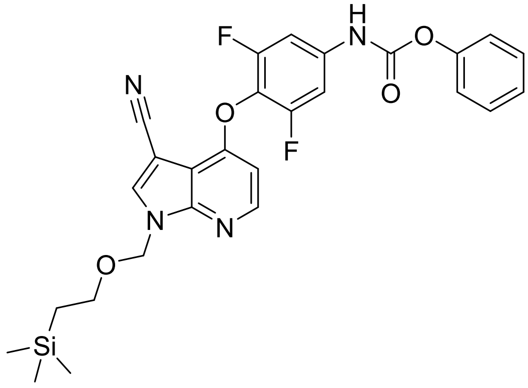

A solution of 4-(4-amino-2,6-difluorophenoxy)-1-{[2-(trimethylsilyl)ethoxy]methyl}-1*H*-pyrrolo[2,3-b]pyridine-3-carbonitrile (390 mg, 0.936 mmol, **S49**) in ethyl acetate (5 mL) was treated with an aqueous sat. sodium hydrocarbonate solution (5 mL) and with phenyl chloroformate (CAS No. [1885-14-9]; 5.0 eq, 0.59 mL, 4.7 mmol) and the mixture stirred at rt for 18 hours. The reaction mixture was diluted with ethyl acetate and water, the phases separated and the aqueous phase extracted with ethyl acetate (twice). The combined organic phases were washed with brine, dried with sodium sulfate and concentrated in vacuo. The obtained material was purified by flash chromatography (SiO_2-hexane/ ethyl acetate) to give the title compound (310 mg) containing 1-{4-[(3-cyano-1-{[2-(trimethylsilyl)ethoxy]methyl}-1H-pyrrolo[2,3-b]pyridin-4-yl)oxy]-3,5-difluorophenyl}urea. This material was directly used in the next step without further purification. (22% purity by LC/MS)

Method 1, UPLC-MS (ESI+): t_R = 1.53 min; *m*/*z* calcd for $C_{27}H_{27}F_2N_4O_4Si$ $[M + H]^+$: 537.2; found: 537.5

1-{4-[(3-cyano-1-{[2-(trimethylsilyl)ethoxy]methyl}-1H-pyrrolo[2,3-b]pyridin-4-yl)oxy]-3,5-difluorophenyl}-3-[(3-methyloxetan-3-yl)methyl]urea (S51)

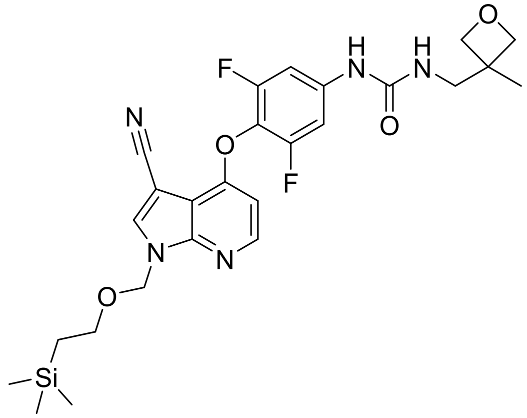

A solution of phenyl {4-[(3-cyano-1-{[2-(trimethylsilyl)ethoxy]methyl}-1*H*-pyrrolo[2,3-b]pyridin-4-yl)oxy]-3,5-difluorophenyl}carbamate (330 mg, 615 µmol, **S50**) in DMF (3 mL) was treated with 1-(3-methyloxetan-3-yl)methanamine (1.2 eq., 75 mg, 738 µmol) and stirred at 70 °C for 20 hours. The reaction mixture was diluted with ethyl acetate and water and the layers separated. The organic layer was concentrated in vacuo to give the crude title compound which was used in the next step without any further purification.

Method 1, UPLC-MS (ESI+): t_R = 1.34 min; *m/z* calcd for $C_{26}H_{32}F_2N_5O_4Si$ $[M + H]^+$: 544.2; found: 544.5

Compound 22

(+/-)-4-(2,6-difluoro-4-{[5-(hydroxymethyl)-5-methyl-5,6-dihydro-4*H*-1,3-oxazin-2-yl]amino}phenoxy)-1*H*-pyrrolo[2,3-b]pyridine-3-carbonitrile

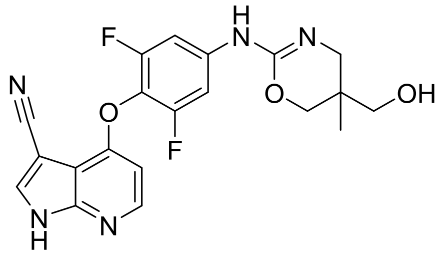

A solution of crude 1-{4-[(3-cyano-1-{[2-(trimethylsilyl)ethoxy]methyl}-1*H*-pyrrolo[2,3-b]pyridin-4-yl)oxy]-3,5-difluorophenyl}-3-[(3-methyloxetan-3-yl)methyl]urea (334 mg, 615 µmol, **S51**) in dichloromethane (2 mL) was treated with trifluoroacetic acid (40 eq., 1.9 mL, 25 mmol) at room temperature overnight. The reaction mixture was concentrated in vacuo and the residue subjected to preparative HPLC to give the title compound (72 mg, 26%).

Method 4, LC-MS (ESI+): t_R = 0.74 min; *m/z* calcd for $C_{20}H_{18}F_2N_5O_3$ $[M + H]^+$: 414.1; found: 414.2

^{1}H-NMR (400 MHz, DMSO-d_6) δ [ppm] = 0.91 (s, 3H), 3.00 – 3.03 (m, 1H), 3.20 – 3.35 (m, 3H), 3.88 (d, 1H), 4.07 (d, 1H), 4.83 (br s, 1H), 6.50 (d, 1H), 7.58 (m_c, 2H), 8.22 (d, 1H), 8.43 (s, 1H), 9.07 (br s, 1H), 12.95 (br s, 1H).

3-bromo-4-(2,6-difluoro-4-nitrophenoxy)-1-{[2-(trimethylsilyl)ethoxy]methyl}-1H-pyrrolo[2,3-b]pyridine (S52)

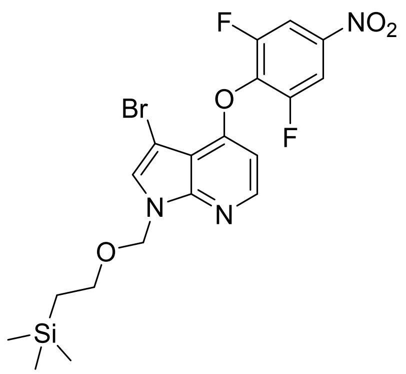

To a stirred solution of 4-(2,6-difluoro-4-nitrophenoxy)-1-{[2-(trimethylsilyl)ethoxy]methyl}-1H-pyrrolo[2,3-b]pyridine (10.0 g, 23.7 mmol, **S19**) in *N,N*-dimethylformamide (200 mL) was added N-bromosuccinimide (4.65 g, 26.1 mmol). The resulting reaction mixture was stirred at rt overnight. The mixture was diluted with water and ethyl acetate and a saturated aqueous solution of sodium bicarbonate was added. The layers were separated and the aqueous phase was extracted two times with ethyl acetate. The combined organic layers were washed with brine, dried over magnesium sulphate, filtered, and concentrated in vacuo to afford the crude product. The crude product was purified by flash column chromatography over silica gel to afford the title product (10.3 g, 87% yield).

Method 1, UPLC-MS (ESI+): t_R = 1.67 min; *m*/*z* calcd for $C_{19}H_{20}BrF_2N_3O_4Si$ $[M+H]^+$: 500.04; found: 500.0

^{1}H-NMR (400 MHz, DMSO-d_6) δ [ppm] = -0.10 (s, 9H), 0.80 – 0.84 (m, 2H), 3.52 – 3.56 (m, 2H), 5.62 (s, 2H), 6.66 (d, 1H), 7.94 (d, 1H), 8.21 (d, 1H), 8.39 – 8.45 (m, 2H).

5-[4-(2,6-difluoro-4-nitrophenoxy)-1-{[2-(trimethylsilyl)ethoxy]methyl}-1H-pyrrolo[2,3-b]pyridin-3-yl]-2-(propan-2-yloxy)benzonitrile (S53)

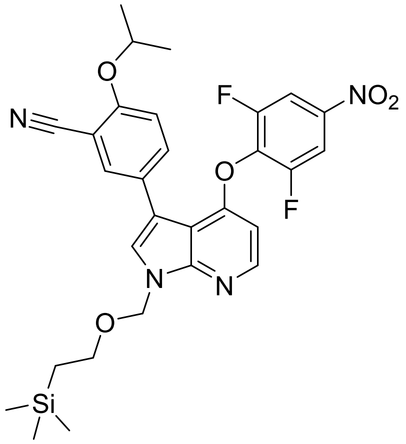

3-bromo-4-(2,6-difluoro-4-nitrophenoxy)-1-{[2-(trimethylsilyl)ethoxy]methyl}-1H-pyrrolo[2,3-b]pyridine (3.66 g, 7.32 mmol, **S52**), [1,1′-Bis(diphenylphosphino)-ferrocene]dichloropalladium(II) (535 mg, 0.73 mmol), [3-cyano-4-(propan-2-yloxy)phenyl]boronic acid (3.0 mg, 14.6 mmol, CAS No. [1009303-59-6]), and potassium carbonate (5.06 g, 36.6 mmol), were dissolved in a mixture of 1,4-dioxane (70 mL), and water (35 mL). The resulting mixture was degassed with argon for 10 min, after which time it was heated to 100°C for 3h. The reaction mixture was cooled to room temperature, diluted with ethyl acetate and water was added. The layers were separated, and the aqueous phase was extracted two times with ethyl acetate. The combined organic phases were washed with brine, dried over sodium sulfate, filtered and concentrated to dryness to give the crude product. The crude produt was purified by flash column chromatography to afford 5-[4-(2,6-difluoro-4-nitrophenoxy)-1-{[2-(trimethylsilyl)ethoxy]methyl}-1H-pyrrolo[2,3-b]pyridin-3-yl]-2-(propan-2-yloxy)benzonitrile (2.92 g, 69% Yield).

Method 1, UPLC-MS (ESI+): t_R = 1.66 min; *m*/*z* calcd for $C_{29}H_{31}F_2N_4O_5Si$ $[M + H]^+$: 581.2; found: 581.4

^1H NMR (400 MHz, DMSO-d_6) δ ppm -0.09 (s, 9 H), 0.78 - 0.93 (m, 1 H), 1.31 (d, 6 H), 3.52 - 3.65 (m, 4 H), 4.80 (spt, 1 H), 5.68 (s, 1 H), 6.66 (d, 1 H), 7.32 (d, 1 H), 7.85 - 7.90 (m, 2 H), 7.96 (s, 1 H), 8.23 (d, 1 H), 8.38 - 8.46 (m, 2 H)

5-[4-(4-amino-2,6-difluorophenoxy)-1-{[2-(trimethylsilyl)ethoxy]methyl}-1H-pyrrolo[2,3-b]pyridin-3-yl]-2-(propan-2-yloxy)benzonitrile (S54)

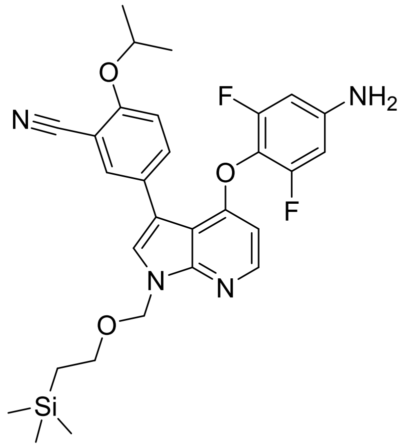

To a solution of 5-[4-(2,6-difluoro-4-nitrophenoxy)-1-{[2-(trimethylsilyl)ethoxy]methyl}-1H-pyrrolo[2,3-b]pyridin-3-yl]-2-(propan-2-yloxy)benzonitrile (2.92 g, 5.03 mmol, **S53**) in a mixture of THF (35 mL), water (70 mL), and methanol (35 mL) was added ammonium chloride

(1.35 g, 25.1 mmol) and iron powder (1.40 g, 25.4 mmol). The resulting mixture was stirred at 80 degrees for 2 hours at which time the mixture was cooled and filtered. The filtrate was extracted three times with ethyl acetate and the combined organic layers washed with brine, dried over sodium sulfate, and evaporated to afford 5-[4-(4-amino-2,6-difluorophenoxy)-1-{[2-(trimethylsilyl)ethoxy]methyl}-1H-pyrrolo[2,3-b]pyridin-3-yl]-2-(propan-2-yloxy)benzonitrile (2.21 g, 80% Yield), which required no further purification.

Method 1, UPLC-MS (ESI+): t_R = 1.57 min; *m/z* calcd for $C_{29}H_{33}F_2N_4O_3Si$ $[M + H]^+$: 551.2; found: 551.4

^1H NMR (400 MHz, DMSO-d_6) δ ppm -0.08 (s, 9 H), 0.79 - 0.94 (m, 2 H), 1.32 (d, 6 H), 3.53 - 3.66 (m, 2 H), 4.81 (spt, 1 H), 5.65 (s, 2 H), 5.82 (s, 2 H), 6.37 - 6.44 (m, 3 H), 7.32 (d, 1 H), 7.87 - 7.94 (m, 3 H), 8.18 (d, 1 H)

1-{4-[(3-[3-cyano-4-(propan-2-yloxy)phenyl]-1-{[2-(trimethylsilyl)ethoxy]methyl}-1H-pyrrolo[2,3-b]pyridin-4-yl)oxy]-3,5-difluorophenyl}-3-[(3-methyloxetan-3-yl)methyl]urea (S55)

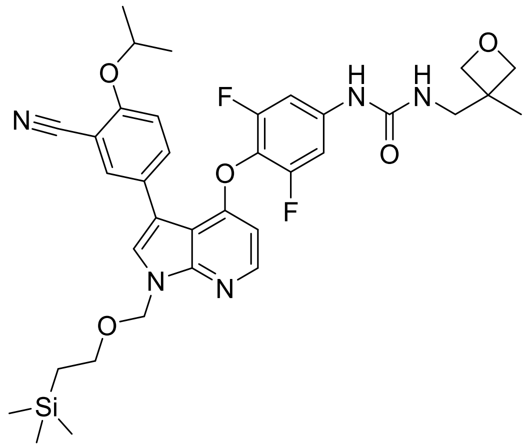

To a stirred solution of 5-[4-(4-amino-2,6-difluorophenoxy)-1-{[2-(trimethylsilyl)ethoxy]methyl}-1H-pyrrolo[2,3-b]pyridin-3-yl]-2-(propan-2-yloxy)benzonitrile (118 mg, 0.21 mmol, **S54**) in a mixture of dichloromethane (1.0 mL) and pyridine (1.0 mL) was added 3-(isocyanatomethyl)-3-methyloxetane (CAS No. [1260665-88-0]) (136 mg, 1.07 mmol). The resulting mixture was stirred at 60°C for 16 hours, at which time the reaction was cooled to room temperature and ethyl acetate and water were added. The layers were separated, and the aqueous phase was extracted two times with ethyl acetate. The combined organic phases were washed with brine, dried over sodium sulfate, filtered and concentrated to dryness to give 1-{4-[(3-[3-cyano-4-(propan-2-yloxy)phenyl]-1-{[2-

(trimethylsilyl)ethoxy]methyl}-1H-pyrrolo[2,3-b]pyridin-4-yl)oxy]-3,5-difluorophenyl}-3-[(3-methyloxetan-3-yl)methyl]urea, which was used in the subsequent reaction without further purification.

Method 1, UPLC-MS (ESI+): t_R = 1.55 min; *m/z* calcd for $C_{35}H_{42}F_2N_5O_5Si$ $[M + H]^+$: 678.3; found: 678.6

Compound 23

(+/-)-5-[4-(2,6-difluoro-4-{[5-(hydroxymethyl)-5-methyl-5,6-dihydro-4H-1,3-oxazin-2-yl]amino}phenoxy)-1H-pyrrolo[2,3-b]pyridin-3-yl]-2-(propan-2-yloxy)benzonitrile

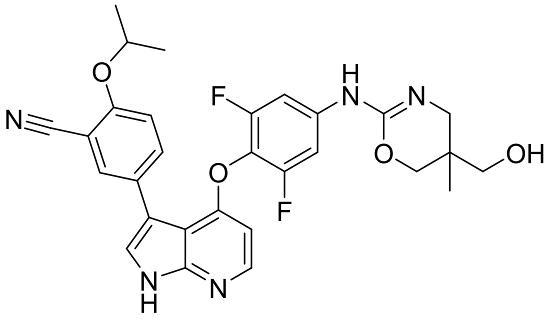

To a solution of 1-{4-[(3-[3-cyano-4-(propan-2-yloxy)phenyl]-1-{[2-(trimethylsilyl)ethoxy]methyl}-1H-pyrrolo[2,3-b]pyridin-4-yl)oxy]-3,5-difluorophenyl}-3-[(3-methyloxetan-3-yl)methyl]urea (80 mg, 0.12 mmol, **S55**) in dichloromethane (4.0 mL) was added trifluoroacetic acid (1.0 mL, 13 mmol). The resulting mixture was stirred at room temperature for 16 hours. 2M NaOH was added, and the mixture extracted two times with ethyl acetate. The combined organic layers were washed with water, brine, dried over sodium sulfate, filtered and evaporated to afford the crude oxazine. The crude product was purified by preparative HPLC to afford (+/-)-5-[4-(2,6-difluoro-4-{[5-(hydroxymethyl)-5-methyl-5,6-dihydro-4H-1,3-oxazin-2-yl]amino}phenoxy)-1H-pyrrolo[2,3-b]pyridin-3-yl]-2-(propan-2-yloxy)benzonitrile (38 mg, 33% over two steps).

Method 1, UPLC-MS (ESI+): t_R = 1.14 min; *m/z* calcd for $C_{29}H_{28}F_2N_5O_4$ $[M + H]^+$: 548.2; found: 548.3

^{1}H NMR (400 MHz, DMSO-d_6) δ ppm 0.90 (s, 3 H), 1.31 (d, 6 H), 2.96 - 3.09 (m, 1 H), 3.16 - 3.33 (m, 3 H), 3.87 (br d, 1 H), 4.06 (br d, 1 H), 4.74 - 4.87 (m, 2 H), 6.33 (d, 1 H), 7.30 (d, 1 H), 7.58 (br s, 2 H), 7.70 (s, 1 H), 7.89 - 7.95 (m, 2 H), 8.10 (d, 1 H), 9.02 (br s, 1 H), 12.14 (s, 1 H)

4-(2,6-difluoro-4-nitrophenoxy)-3-[1-(propan-2-yl)-1H-pyrazol-5-yl]-1-{[2-(trimethylsilyl)ethoxy]methyl}-1H-pyrrolo[2,3-b]pyridine (S56)

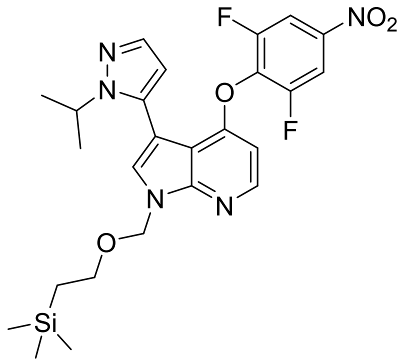

3-bromo-4-(2,6-difluoro-4-nitrophenoxy)-1-{[2-(trimethylsilyl)ethoxy]methyl}-1H-pyrrolo[2,3-b]pyridine (1.00 g, 2.00 mmol, **S52**), [1,1′-Bis(diphenylphosphino)-ferrocene]dichloropalladium(II) (146 mg, 0.20 mmol), [1-(propan-2-yl)-1H-pyrazol-5-yl]boronic acid (615 mg, 4.00 mmol, CAS No. [839714-33-9]), and potassium carbonate (1.38 g, 9.99 mmol), were dissolved in a mixture of 1,4-dioxane (20 mL), and water (10 mL). The resulting mixture was degassed with argon for 10 min, after which time it was heated to 100°C for 3h. The reaction mixture was cooled to room temperature, diluted with ethyl acetate and water was added. The layers were separated, and the aqueous phase was extracted two times with ethyl acetate. The combined organic phases were washed with brine, dried over sodium sulfate, filtered and concentrated to dryness to give the crude product. The crude product was purified by flash column chromatography to afford the title compound (278 mg, 26% Yield).

Method 1, UPLC-MS (ESI+): t_R = 1.58 min; *m/z* calcd for $C_{25}H_{30}F_2N_5O_4Si$ $[M + H]^+$: 530.2; found: 530.5

^1H NMR (400 MHz, DMSO-d_6) δ ppm -0.11 (s, 9 H), 0.78 - 0.87 (m, 2 H), 1.30 (d, 6 H), 3.55 - 3.67 (m, 2 H), 4.51 (spt, 1 H), 5.71 (s, 2 H), 6.28 (d, 1 H), 6.63 (d, 1 H), 7.47 (d, 1 H), 7.87 (s, 1 H), 8.24 (d, 1 H), 8.33 - 8.40 (m, 2 H)

3,5-difluoro-4-[(3-[1-(propan-2-yl)-1H-pyrazol-5-yl]-1-{[2-(trimethylsilyl)ethoxy]methyl}-1H-pyrrolo[2,3-b]pyridin-4-yl)oxy]aniline (S57)

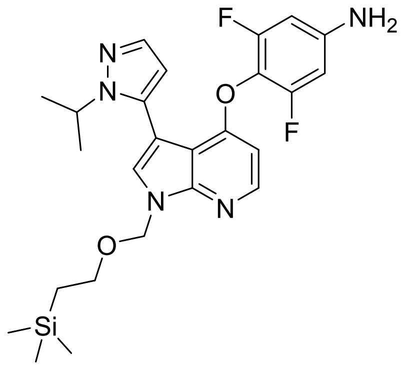

To a solution of 4-(2,6-difluoro-4-nitrophenoxy)-3-[1-(propan-2-yl)-1H-pyrazol-5-yl]-1-{[2-(trimethylsilyl)ethoxy]methyl}-1H-pyrrolo[2,3-b]pyridine (278 mg, 0.53 mmol, **S56**) in a mixture of THF (2 mL), water (4 mL), and methanol (2 mL) was added ammonium chloride (140 mg, 2.62 mmol) and iron powder (147 mg, 2.62 mmol). The resulting mixture was stirred at 80 degrees for 2 hours at which time the mixture was cooled and filtered. The filtrate was extracted three times with ethyl acetate and the combined organic layers washed with brine, dried over sodium sulfate, and evaporated to afford 3,5-difluoro-4-[(3-[1-(propan-2-yl)-1H-pyrazol-5-yl]-1-{[2-(trimethylsilyl)ethoxy]methyl}-1H-pyrrolo[2,3-b]pyridin-4-yl)oxy]aniline (259 mg, 99% Yield), which required no further purification.

Method 1, UPLC-MS (ESI+): t_R = 1.46 min; *m*/*z* calcd for $C_{25}H_{32}F_2N_5O_2Si$ $[M + H]^+$: 500.2; found: 500.4

^1H NMR (400 MHz, DMSO-d_6) δ ppm -0.10 (s, 9 H), 0.78 - 0.87 (m, 2 H), 1.29 (d, 6 H), 3.58 (t, 2 H), 4.53 (spt, 1 H), 5.67 (s, 2 H), 5.78 (s, 2 H), 6.26 (d, 1 H), 6.31 - 6.41 (m, 3 H), 7.47 (d, 1 H), 7.75 (s, 1 H), 8.18 (d, 1 H)

1-{3,5-difluoro-4-[(3-[1-(propan-2-yl)-1H-pyrazol-5-yl]-1-{[2-(trimethylsilyl)ethoxy]methyl}-1H-pyrrolo[2,3-b]pyridin-4-yl)oxy]phenyl}-3-[(3-methyloxetan-3-yl)methyl]urea (S58)

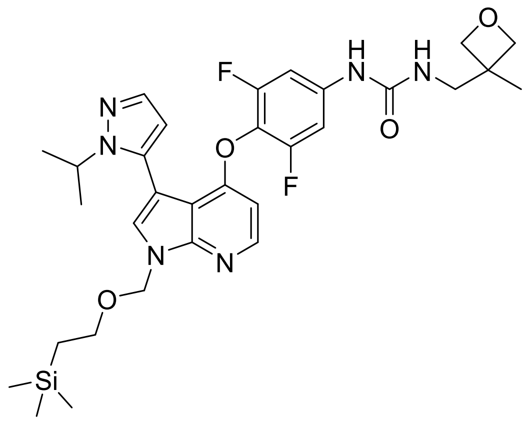

To a stirred solution of 3,5-difluoro-4-[(3-[1-(propan-2-yl)-1H-pyrazol-5-yl]-1-{[2-(trimethylsilyl)ethoxy]methyl}-1H-pyrrolo[2,3-b]pyridin-4-yl)oxy]aniline (85.0 mg, 0.17 mmol, **S57**) in a mixture of dichloromethane (1.0 mL) and pyridine (1.0 mL) was added 3-(isocyanatomethyl)-3-methyloxetane (CAS No. [1260665-88-0]) (108 mg, 0.85 mmol). The resulting mixture was stirred at 60°C for 16 hours, at which time the reaction was cooled to room temperature and ethyl acetate and water were added. The layers were separated, and the aqueous phase was extracted two times with ethyl acetate. The combined organic phases were washed with brine, dried over sodium sulfate, filtered and concentrated to dryness to give the crude title product, which was used in the subsequent reaction without further purification.

Method 1, UPLC-MS (ESI+): t_R = 1.45 min; *m/z* calcd for $C_{31}H_{41}F_2N_6O_4Si$ $[M + H]^+$: 627.3; found: 627.6

<u>Compound 24</u>

(+/-)-[2-{[3,5-difluoro-4-({3-[1-(propan-2-yl)-1H-pyrazol-5-yl]-1H-pyrrolo[2,3-b]pyridin-4-yl}oxy)phenyl]amino}-5-methyl-5,6-dihydro-4H-1,3-oxazin-5-yl]methanol

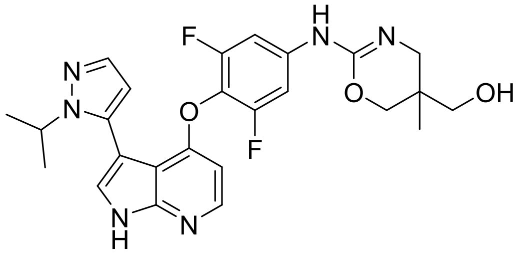

To a solution of 1-{3,5-difluoro-4-[(3-[1-(propan-2-yl)-1H-pyrazol-5-yl]-1-{[2-(trimethylsilyl)ethoxy]methyl}-1H-pyrrolo[2,3-b]pyridin-4-yl)oxy]phenyl}-3-[(3-methyloxetan-3-yl)methyl]urea (105 mg, 0.17 mmol, **S58**) in dichloromethane (2.0 mL) was added trifluoroacetic acid (1.0 mL, 13 mmol). The resulting mixture was stirred at room temperature

for 16 hours. 2M NaOH was added, and the mixture extracted two times with ethyl acetate. The combined organic layers were washed with water, brine, dried over sodium sulfate, filtered and evaporated to afford the crude oxazine. The crude product was purified by preparative HPLC to afford the title compound (31 mg, 37% over two steps).

Method 1, UPLC-MS (ESI+): t_R = 0.92 min; *m*/*z* calcd for $C_{25}H_{26}F_2N_6O_3$ $[M + H]^+$: 497.2; found: 497.6

^{1}H NMR (400 MHz, DMSO-d_6) δ ppm 0.89 (br s, 3 H), 1.28 (d, 6 H), 2.96 - 3.06 (m, 1 H), 3.19 - 3.32 (m, 2 H), 3.86 (br d, 1 H), 4.05 (br d, 1 H), 4.55 (spt, 1 H), 4.80 (br s, 1 H), 6.25 (d, 1 H), 6.28 (d, 1 H), 7.45 (d, 1 H), 7.52 (br s, 2 H), 7.55 (d, 1 H), 8.11 (d, 1 H), 8.99 (br s, 1 H), 12.24 (s, 1 H)

3-bromo-4-(2,6-difluoro-4-nitrophenoxy)-1H-pyrrolo[2,3-b]pyridine (S59)

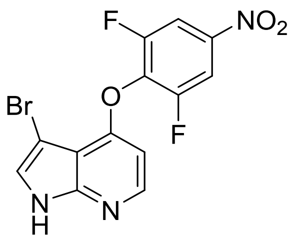

To a stirred solution of 4-(2,6-difluoro-4-nitrophenoxy)-1H-pyrrolo[2,3-b]pyridine (5.70 g, 19.6 mmol, **S18**) in DMF (100 mL) was added N-bromosuccinimide (3.83 g, 21.5 mmol). The resulting mixture was stirred at room temperature for 2 hours at which time the reaction was cooled to 0°C and water was slowly added. The resulting precipitate was filtered and dried to afford the title compound (7.20 g, 99 % yield)

Method 1, UPLC-MS (ESI+): t_R = 1.18 min; *m*/*z* calcd for $C_{13}H_7BrF_2N_3O_3$ $[M + H]^+$: 369.9; found: 369.4

^{1}H NMR (400 MHz, DMSO-d_6) δ ppm 6.55 (d, 1 H), 7.72 (d, 1 H), 8.14 (d, 1 H), 8.41 (d, 2 H), 12.34 (br s, 1 H)

3-bromo-4-(2,6-difluoro-4-nitrophenoxy)-1-(4-methylbenzene-1-sulfonyl)-1H-pyrrolo[2,3-b]pyridine (S60)

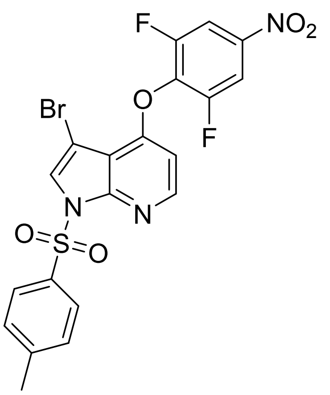

To a cooled (0°C) solution of 3-bromo-4-(2,6-difluoro-4-nitrophenoxy)-1H-pyrrolo[2,3-b]pyridine (13.0 g, 35.1 mmol, **S59**) in DMF (200 mL) was added sodium hydride (1.83 g, 60 % suspension in oil, 45.7 mmol) portion-wise. The resulting mixture was stirred for 15 minutes at 0°C at which time p-toluenesulfonylchloride (7.37 g, 38.6 mmol) was added and the reaction was allowed to warm slowly to room temperature overnight. The mixture was slowly poured into ice-water, and the resulting precipitate was filtered and dried to afford 15 g (81% yield) of the desired product.

Method 1, UPLC-MS (ESI+): t_R = 1.51 min; *m*/*z* calcd for $C_{20}H_{13}BrF_2N_3O_5S$ $[M + H]^+$: 524.0; found: 524.2

^1H NMR (400 MHz, DMSO-d_6) δ ppm 2.37 (s, 3 H), 6.87 (d, 1 H), 7.46 (d, 2 H), 8.04 (d, 2 H), 8.22 (s, 1 H), 8.31 (d, 1 H), 8.42 (d, 2 H)

4-{[3-bromo-1-(4-methylbenzene-1-sulfonyl)-1H-pyrrolo[2,3-b]pyridin-4-yl]oxy}-3,5-difluoroaniline (S61)

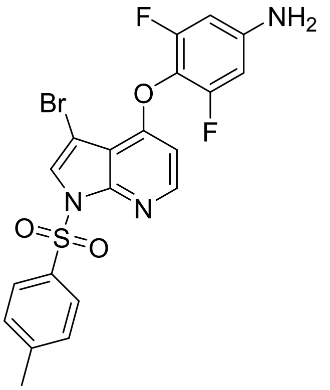

To a solution of 3-bromo-4-(2,6-difluoro-4-nitrophenoxy)-1-(4-methylbenzene-1-sulfonyl)-1H-pyrrolo[2,3-b]pyridine (5.20 g, 9.92 mmol, **S60**) in a mixture of THF: water: methanol (1:2:1, 200 mL), was added ammonium chloride (2.65 g, 49.6 mmol) and iron powder (2.77 g, 49.6

mmol). The resulting mixture was stirred at 80°C for 2 hours at which time the mixture was cooled and filtered over celite. The filtrate was extracted twice with ethyl acetate and the combined organic layers washed with brine, dried (Na2SO4), and evaporated to give the crude product 4.90 g (100% yield) which was sufficiently pure for the next step without further purification.

Method 1, UPLC-MS (ESI+): t_R = 1.39 min; *m*/*z* calcd for $C_{20}H_{15}BrF_2N_3O_3S$ [M + H]$^+$: 494.0; found: 494.3

^{1}H NMR (400 MHz, DMSO-d_6) δ ppm 2.35 (s, 3 H), 5.85 (s, 2 H), 6.37 (d, 2 H), 6.57 (d, 1 H), 7.44 (d, 2 H), 8.02 (d, 2 H), 8.10 (s, 1 H), 8.24 (d, 1 H)

tert-butyl (4-{[3-bromo-1-(4-methylbenzene-1-sulfonyl)-1H-pyrrolo[2,3-b]pyridin-4-yl]oxy}-3,5-difluorophenyl)carbamate (S62)

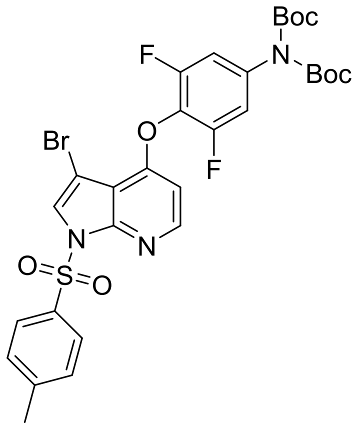

To a solution of 4-{[3-bromo-1-(4-methylbenzene-1-sulfonyl)-1H-pyrrolo[2,3-b]pyridin-4-yl]oxy}-3,5-difluoroaniline (4.30 g, 8.70 mmol, **S61**) in THF (30 mL) was added di-tert-butyl dicarbonate (4.7 mL, 22 mmol) and dimethylaminopyridine (106 mg, 870 µmol). The resulting mixture was stirred at 75°C for 3h, at which time ethyl acetate and water were added and the layers were separated. The aqueous phase was extracted twice with ethyl acetate and the combined organic layers were washed with brine, dried over sodium sulfate, and evaporated to afford the crude product. The crude product was purified by flash column chromatography followed by crystallization from a mixture of dichloromethane and methanol to afford the title compound (1.68g, 28% yield).

Method 1, UPLC-MS (ESI+): t_R = 1.70 min; *m*/*z* calcd for $C_{30}H_{31}BrF_2N_3O_7S$ [M + H]$^+$: 694.1; found: 694.4

^{1}H NMR (400 MHz, DMSO-d_6) δ ppm 1.41 (s, 18 H), 2.36 (s, 3 H), 6.46 (d, 1 H), 7.45 (d, 2 H), 7.53 (d, 2 H), 8.04 (d, 2 H), 8.19 (s, 1 H), 8.31 (d, 1 H)

di-tert-butyl (3,5-difluoro-4-{[1-(4-methylbenzene-1-sulfonyl)-3-(propan-2-yl)-1H-pyrrolo[2,3-b]pyridin-4-yl]oxy}phenyl)-2-imidodicarbonate (S63)

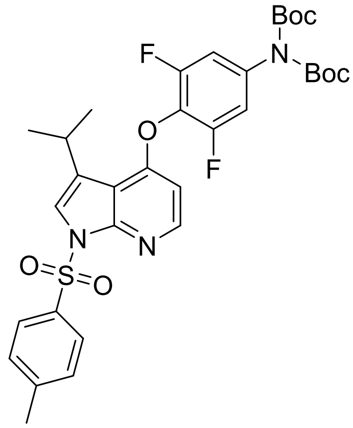

di-tert-butyl (4-{[3-bromo-1-(4-methylbenzene-1-sulfonyl)-1H-pyrrolo[2,3-b]pyridin-4-yl]oxy}-3,5-difluorophenyl)-2-imidodicarbonate (500 mg, 720 µmol, **S62**), tris(trimethylsilyl)silane (220µL, 720µmol, CAS No. [1873-77-4]), 2-bromopropane (300 µL, 3.2 mmol, CAS No. [75-26-3]), 2,6-dimethoxypyridine (570 µL, 4.3mmol) and Ir(4',6'-dF-5-CF_3-ppy)$_2$(4,4'-dtbbpy)PF_6 (16 mg, 14 µmol, CAS No. [870987-63-6]) were dissolved in the reaction vial in trifluorotoluene (11 mL). In a separate vial, the Ni-catalyst was prepared by dissolving Nickel (II) chloride dimethoxyethane adduct (8 mg, 36 µmol, CAS No. [29046-78-4]) and 4,4'-Di-tert-butyl-2,2'-bipyridine (10 mg, 36 µmol, CAS No. [72914-19-3]) in N,N-dimethylacetamide (4.0 mL) followed by stirring for 5 min. The catalyst solution was syringed to the sealed reaction vial and degassed by sparging with argon for 10 minutes. The MW-vial was placed in a heatblock and tempered to 40°C. The reaction mixture was pumped through the flow tubing using a peristaltic pump (Flow Setup: Loop Volume: 2mL, Tube: inner diameter: 0,2 mm wall thickness 0,2mm, 30% Peristaltic pump speed ~35seconds irradiated residence time, 9h circleflow) for 9 hours at which time water and ethyl acetate were added and the layers were separated. The aqueous phase was extracted twice with ethyl acetate and the combined organic layers were washed with brine, dried over sodium sulfate, and evaporated to give the crude material. The crude material was purified by flash column chromatography to afford a 2:1 inseparable mixture of the desired product di-tert-butyl (3,5-difluoro-4-{[1-(4-methylbenzene-1-sulfonyl)-3-(propan-2-yl)-1H-pyrrolo[2,3-b]pyridin-4-yl]oxy}phenyl)-2-

imidodicarbonate and the de-brominated starting material di-tert-butyl [3,5-difluoro-4-({1-[(4-methylphenyl)sulfonyl]-1H-pyrrolo[2,3-b]pyridin-4-yl}oxy)phenyl]-2-imidodicarbonate (1.69 g, 59% combined yield)

Method 1, UPLC-MS (ESI+): t_R = 1.74 min; *m*/*z* calcd for $C_{33}H_{38}F_2N_3O_7S$ [M + H]$^+$: 658.2; found: 658.3

3,5-difluoro-4-{[1-(4-methylbenzene-1-sulfonyl)-3-(propan-2-yl)-1H-pyrrolo[2,3-b]pyridin-4-yl]oxy}aniline—hydrogen chloride (1/1) (S64)

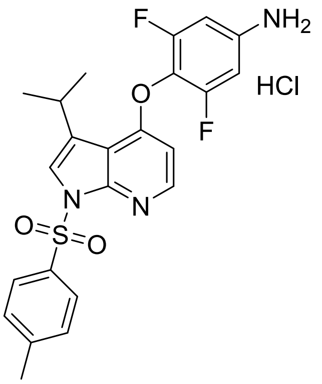

di-tert-butyl (3,5-difluoro-4-{[1-(4-methylbenzene-1-sulfonyl)-3-(propan-2-yl)-1H-pyrrolo[2,3-b]pyridin-4-yl]oxy}phenyl)-2-imidodicarbonate (1.69 g, 66 % purity, 1.70 mmol, **S63**) was dissolved in a 4 M solution of hydrochloric acid in dioxane (30 mL) and stirred at room temperature overnight. The solvent was subsequently evaporated to give the crude product which was used in the subsequent step without further purification.

Method 1, UPLC-MS (ESI+): t_R = 1.45 min; *m*/*z* calcd for $C_{23}H_{22}F_2N_3O_3S$ [M + H]$^+$: 458.1; found: 458.3

Phenyl (3,5-difluoro-4-{[1-(4-methylbenzene-1-sulfonyl)-3-(propan-2-yl)-1H-pyrrolo[2,3-b]pyridin-4-yl]oxy}phenyl)carbamate (S65)

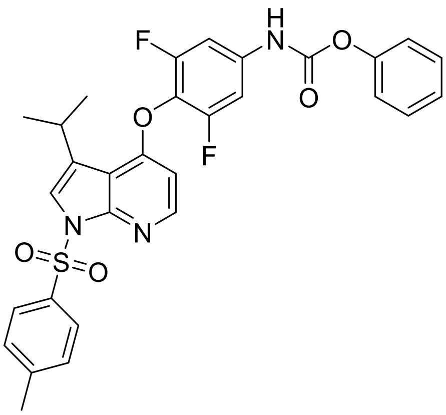

To a stirred solution of 3,5-difluoro-4-{[1-(4-methylbenzene-1-sulfonyl)-3-(propan-2-yl)-1H-pyrrolo[2,3-b]pyridin-4-yl]oxy}aniline—hydrogen chloride (1/1) (1.40 g, 66 % purity, 1.87 mmol, **S64**) in THF (15 mL) and pyridine was added (2.0 mL, 25 mmol). The reaction mixture was cooled to 0°C and phenyl carbonochloridate (360 µL, 2.9 mmol, CAS No. [1885-14-9]) was added. The resulting mixture was stirred for 30 min at 0°C, at which time it was diluted with ethyl acetate and a 2M aqueous solution of hydrochloric acid was added slowly. The layers were separated and the aqueous phase was extracted twice with ethyl acetate. The combined organic phases were washed with a saturated solution of sodium bicarbonate, followed by brine, dried over sodium sulfate, and evaporated to afford the crude product. The crude product was used without further purification.

Method 2, UPLC-MS (ESI+): t_R = 1.62 min; *m*/*z* calcd for $C_{30}H_{26}F_2N_3O_5S$ $[M + H]^+$: 578.2; found: 578.6

N-(3,5-difluoro-4-{[1-(4-methylbenzene-1-sulfonyl)-3-(propan-2-yl)-1H-pyrrolo[2,3-b]pyridin-4-yl]oxy}phenyl)-N'-[(3-methyloxetan-3-yl)methyl]urea (S66)

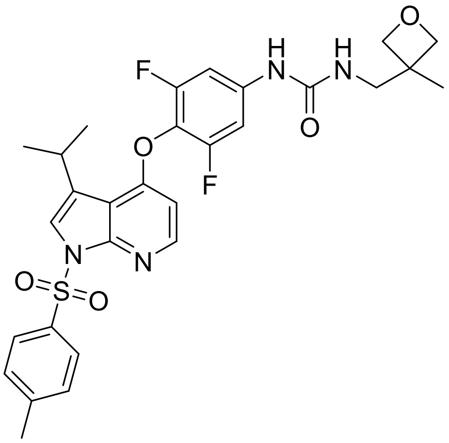

In analogy to **S3**, phenyl (3,5-difluoro-4-{[1-(4-methylbenzene-1-sulfonyl)-3-(propan-2-yl)-1H-pyrrolo[2,3-b]pyridin-4-yl]oxy}phenyl)carbamate (250 mg, 66 % purity, 286 µmol, **S65**), and 1-(3-methyloxetan-3-yl)methanamine (72.2 mg, 714 µmol, CAS No. [153209-97-3]), in DMF (2.0 mL) were reacted to obtain a crude product which was used in the next step without further purification.

Method 1, UPLC-MS (ESI+): t_R = 1.40 min; *m*/*z* calcd for $C_{29}H_{31}F_2N_4O_5S$ $[M + H]^+$: 585.2; found: 585.4

N-(3,5-difluoro-4-{[3-(propan-2-yl)-1H-pyrrolo[2,3-b]pyridin-4-yl]oxy}phenyl)-N'-[(3-methyloxetan-3-yl)methyl]urea (S67)

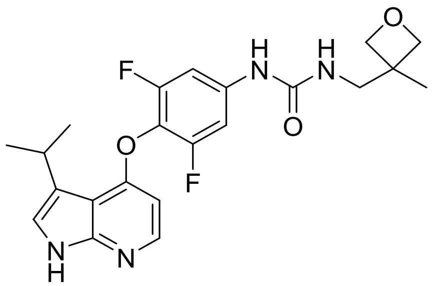

In analogy to **S76**, N-(3,5-difluoro-4-{[1-(4-methylbenzene-1-sulfonyl)-3-(propan-2-yl)-1H-pyrrolo[2,3-b]pyridin-4-yl]oxy}phenyl)-N'-[(3-methyloxetan-3-yl)methyl]urea (160 mg, 274 µmol, **S66**) was reacted with sodium hydroxide (32.8 mg, 821 µmol) in methanol (6.0 mL), to afford a crude product which was used without further purification.

Method 1, UPLC-MS (ESI+): t_R = 1.12 min; *m*/*z* calcd for $C_{22}H_{25}F_2N_4O_3$ $[M + H]^+$: 431.2; found: 431.5

^{1}H NMR (400 MHz, DMSO-d_6) δ ppm 1.23 (s, 3 H), 1.32 (d, 6 H), 3.27 - 3.31 (m, 3 H), 4.19 (d, 2 H), 4.39 (d, 2 H), 6.19 (d, 1 H), 7.14 (s, 1 H), 7.41 (d, 2 H), 7.49 (br s, 1 H), 7.98 (d, 1 H), 9.88 (br s, 1 H), 11.46 (br s, 1 H)

<u>**Compound 25**</u>

(+/-)-[2-(3,5-difluoro-4-{[3-(propan-2-yl)-1H-pyrrolo[2,3-b]pyridin-4-yl]oxy}anilino)-5-methyl-5,6-dihydro-4H-1,3-oxazin-5-yl]methanol

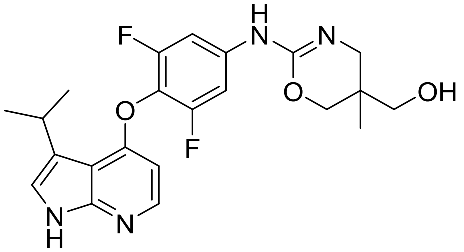

In analogy to compound **2**, N-(3,5-difluoro-4-{[3-(propan-2-yl)-1H-pyrrolo[2,3-b]pyridin-4-yl]oxy}phenyl)-N'-[(3-methyloxetan-3-yl)methyl]urea (110 mg, 256 µmol, **S67**) was treated with trifluoroacetic acid (200 µL, 2.6 mmol) in dichloromethane (2.0 mL) to afford after preparative HPLC purification the title compound (12 mg, 10 % yield).

Method 1, UPLC-MS (ESI+): t_R = 1.09 min; *m/z* calcd for $C_{22}H_{25}F_2N_4O_3$ $[M + H]^+$: 431.2; found: 431.6

^{1}H NMR (400 MHz, DMSO-d_6) δ ppm 0.90 (br s, 3 H), 1.31 (d, 6 H), 2.95 - 3.09 (m, 1 H), 3.18 - 3.36 (m, 4 H), 3.87 (br d, 1 H), 4.06 (br d, 1 H), 4.82 (br s, 1 H), 6.17 (d, 1 H), 7.14 (d, 1 H), 7.56 (br s, 2 H), 7.98 (d, 1 H), 9.01 (br s, 1 H), 11.44 (d, 1 H)

3-cyclopropyl-4-(2,6-difluoro-4-nitrophenoxy)-1-{[2-(trimethylsilyl)ethoxy]methyl}-1*H*-pyrrolo[2,3-b]pyridine (S68)

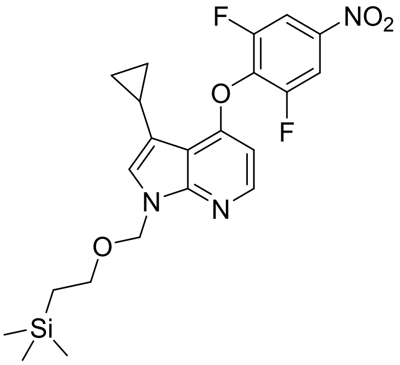

In a microwave glass vial a mixture of 3-bromo-4-(2,6-difluoro-4-nitrophenoxy)-1-{[2-(trimethylsilyl)ethoxy]methyl}-1*H*-pyrrolo[2,3-b]pyridine (6.00 g, 12.0 mmol, **S52**), potassium cyclopropyl(trifluoro)borate (1.10 eq, 1.95 g, 13.2 mmol), palladium acetate (CAS No. [3375-31-3]; 0.02 eq., 54 mg, 240 µmol), potassium carbonate (2.0 eq., 3.3g, 24 mmol) and tricyclohexylphosphine (0.04 eq., 135 mg, 480 µmol) in a mixture of toluene (60 mL) and water (2.4 mL) was evacuated and back-filled with nitrogen several times under stirring. The vial was closed and stirred at 90°C overnight. The reaction mixture was cooled to rt, filtrated

over a pad of Celite® and the filter cake washed with ethyl acetate. The filtrate was concentrated in vacuo and the obtained material purified by flash chromatography (SiO_2-hexane/ dichloromethane to dichloromethane/ methanol) to give the title compound (2.37 g, 36%, contaminated with small amounts of de-halogenated starting material) along with recovered starting material (2.1 g, 35%).

Method 1, UPLC-MS (ESI+): t_R = 1.64 min; *m/z* calcd for $C_{22}H_{26}F_2N_3O_4Si$ $[M + H]^+$: 462.2; found: 462.6

^{1}H-NMR (400 MHz, DMSO-d_6) δ [ppm] = -0.11 (s, 9H), 0.61 – 0.65 (m, 2H), 0.78 – 0.88 (m, 4H), 2.12 – 2.18 (m, 1H), 3.47 – 3.51 (m, 2H), 5.54 (s, 2H), 6.52 (d, 1H), 7.33 (d, 1H), 8.11 (d, 1H), 8.38 – 8.44 (m, 2H).

4-[(3-cyclopropyl-1-{[2-(trimethylsilyl)ethoxy]methyl}-1*H*-pyrrolo[2,3-b]pyridin-4-yl)oxy]-3,5-difluoroaniline (S69)

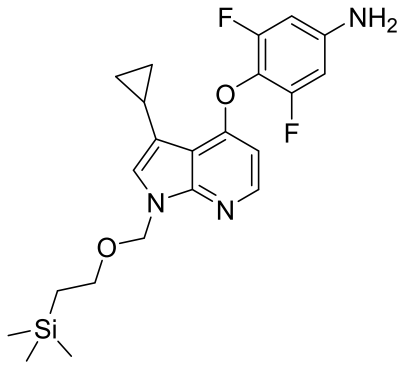

A solution of 3-cyclopropyl-4-(2,6-difluoro-4-nitrophenoxy)-1-{[2-(trimethylsilyl)ethoxy]methyl}-1*H*-pyrrolo[2,3-b]pyridine (2.3 g, 5.0 mmol, **S68**) in methanol (41 mL) was treated with tin(II) chloride dihydrate (CAS No. [10025-69-1]; 1.6 eq, 1.8 g, 7.8 mmol) and stirred at 64°C for 5 hours followed by stirring at rt overnight. The reaction mixture was concentrated in vacuo, the residue taken up with ethyl acetate and aqueous sat. sodium carbonate solution, filtrated over diatomite and the phases separated. The organic phase was washed with aqueous sat. sodium carbonate solution, dried with sodium sulfate and concentrated in vacuo. The obtained material was used in the next step without further purification (1.49 g).

Method 1, UPLC-MS (ESI+): t_R = 1.52 min; *m/z* calcd for $C_{22}H_{28}F_2N_3O_2Si$ $[M + H]^+$: 432.2; found: 432.6

^{1}H-NMR (400 MHz, DMSO-d_6) δ [ppm] = -0.10 (s, 9H), 0.60 – 0.65 (m, 2H), 0.78 – 0.82 (m, 2H), 0.84 – 0.88 (m, 2H), 2.15 – 2.22 (m, 1H), 3.45 – 3.49 (m, 2H), 5.51 (s, 2H), 5.78 (br s, 2H), 6.28 (d, 1H), 6.36 – 6.42 (m, 2H), 7.22 (s, 1H), 8.05 (d, 1H).

Phenyl {4-[(3-cyclopropyl-1-{[2-(trimethylsilyl)ethoxy]methyl}-1*H*-pyrrolo[2,3-b]pyridin-4-yl)oxy]-3,5-difluorophenyl}carbamate (S70)

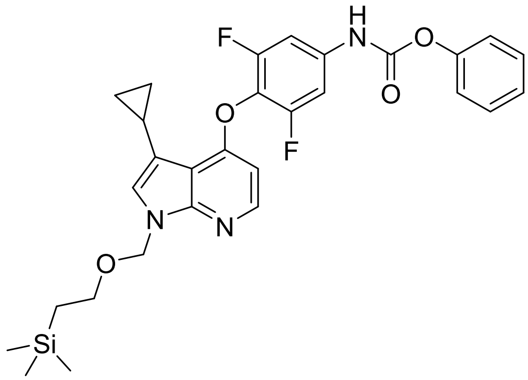

An ice-cooled solution of 4-[(3-cyclopropyl-1-{[2-(trimethylsilyl)ethoxy]methyl}-1*H*-pyrrolo[2,3-b]pyridin-4-yl)oxy]-3,5-difluoroaniline (186 mg, 345 µmol, **S69**) and pyridine (5.0 eq., 140 µL, 1.7 mmol) in THF (2 mL) was treated with phenyl chloroformate (CAS No. [1885-14-9]; 1.1 eq, 48 µL, 380 µmol), the mixture warmed to rt and stirring continued for 25 minutes.

The reaction mixture was diluted with ethyl acetate and washed with 1 M aqueous hydrochloric acid, aqueous sat. sodium hydrocarbonate solution and brine. The resulting organic layer was dried with sodium sulfate and concentrated in vacuo to give the crude title compound (295 mg) which was used as is in the next steps.

Method 1, UPLC-MS (ESI+): t_R = 1.66 min; m/z calcd for $C_{29}H_{32}F_2N_3O_4Si$ $[M + H]^+$: 552.2; found: 552.5

1-{4-[(3-cyclopropyl-1-{[2-(trimethylsilyl)ethoxy]methyl}-1*H*-pyrrolo[2,3-b]pyridin-4-yl)oxy]-3,5-difluorophenyl}-3-[(3-methyloxetan-3-yl)methyl]urea (S71)

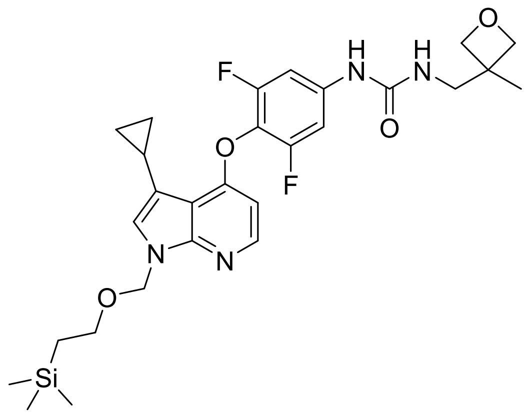

A solution of crude phenyl {4-[(3-cyclopropyl-1-{[2-(trimethylsilyl)ethoxy]methyl}-1*H*-pyrrolo[2,3-b]pyridin-4-yl)oxy]-3,5-difluorophenyl}carbamate (300 mg, 0.5 mmol, **S70**) in *N*,*N*-dimethylformamide (6 mL) was treated with 1-(3-methyloxetan-3-yl)methanamine (CAS No. [153209-97-3]; 1.2 eq, 66 mg, 650 µmol) and the mixture stirred at 70°C for 18 hours. The reaction mixture was diluted with ethyl acetate and water and the phases separated. The organic layer was concentrated in vacuo to give the crude title compound (303 mg) which was used as is in the next step.

Method 1, UPLC-MS (ESI+): t_R = 1.49 min; *m/z* calcd for $C_{28}H_{37}F_2N_4O_4Si$ $[M + H]^+$: 559.3; found: 559.5

Compound 26

(+/-)-[2-({4-[(3-cyclopropyl-1*H*-pyrrolo[2,3-b]pyridin-4-yl)oxy]-3,5-difluorophenyl}amino)-5-methyl-5,6-dihydro-4*H*-1,3-oxazin-5-yl]methanol

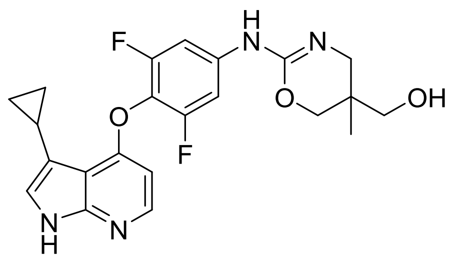

A solution of crude 1-{4-[(3-cyclopropyl-1-{[2-(trimethylsilyl)ethoxy]methyl}-1*H*-pyrrolo[2,3-b]pyridin-4-yl)oxy]-3,5-difluorophenyl}-3-[(3-methyloxetan-3-yl)methyl]urea (303 mg, 0.5 mmol, **S71**) in dichloromethane (3 mL) was treated with trifluoroacetic acid (40 eq., 1.7 mL, 22 mmol) at rt for 5 days. The reaction mixture was concentrated in vacuo and the residue subjected to preparative HPLC to give the title compound (14 mg).

Method 4, LC-MS (ESI+): t_R = 1.01 min; *m/z* calcd for $C_{22}H_{23}F_2N_4O_3$ $[M + H]^+$: 429.2; found: 429.2

^{1}H-NMR (400 MHz, DMSO-d_6) δ [ppm] = 0.61 – 0.64 (m, 2H), 0.81 – 0.85 (m, 2H), 0.90 (s, 3H), 2.14 – 2.20 (m, 1H), 2.99 – 3.03 (m, 1H), 3.20 – 3.27 (m, 3H), 3.87 (d, 1H), 4.06 (d, 1H), 4.83 (br s, 1H), 6.19 (d, 1H), 7.06 (d, 1H), 7.39 – 7.64 (m, 2H), 7.98 (d, 1H), 9.01 (br s, 1H), 11.44 (d, 1H).

di-tert-butyl (3,5-difluoro-4-{[1-(4-methylbenzene-1-sulfonyl)-3-(prop-1-en-2-yl)-1H-pyrrolo[2,3-b]pyridin-4-yl]oxy}phenyl)-2-imidodicarbonate (S72)

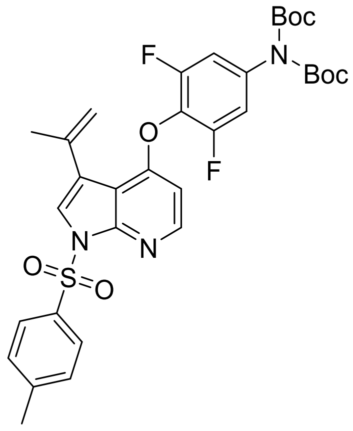

To a stirred solution of di-tert-butyl (4-{[3-bromo-1-(4-methylbenzene-1-sulfonyl)-1H-pyrrolo[2,3-b]pyridin-4-yl]oxy}-3,5-difluorophenyl)-2-imidodicarbonate (1.00 g, 1.44 mmol, **S62**) and 4,4,5,5-tetramethyl-2-(prop-1-en-2-yl)-1,3,2-dioxaborolane (540 µl, 2.9 mmol) and XPhosPdG4 (62 mg, 72.0 µmol) in 1,4-dioxane (27 mL) was added sodium carbonate (2M in water, 3.7 mmol). The reaction was degassed with argon while sonicating for 10min. The resulting mixture was stirred at 100°C for 16h, at which time the mixture was cooled, diluted with ethyl acetate, water was added, and the layer separated. The aqueous phase was extracted twice with ethyl acetate and the combined organic layers were washed with brine, dried over magnesium sulfate, filtered and concentrated under reduced pressure to afford the crude product. The crude product was used without further purification.

3,5-difluoro-4-{[1-(4-methylbenzene-1-sulfonyl)-3-(prop-1-en-2-yl)-1H-pyrrolo[2,3-b]pyridin-4-yl]oxy}aniline (S73)

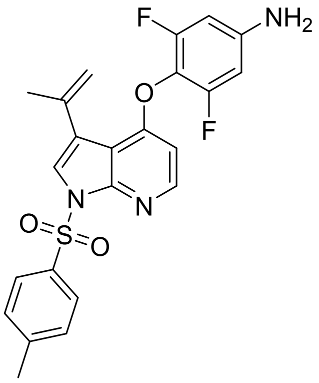

To a stirred solution of di-tert-butyl (3,5-difluoro-4-{[1-(4-methylbenzene-1-sulfonyl)-3-(prop-1-en-2-yl)-1H-pyrrolo[2,3-b]pyridin-4-yl]oxy}phenyl)-2-imidodicarbonate (944 mg, 1.44 mmol, **S72**) in trifluoroethanol (38 mL) was heated to 140°C for 4 h under microwave irradiation. The reaction mixture was cooled and the solvent was evaporated under reduced pressure to afford the crude product which was used without further purification.

Method 1, UPLC-MS (ESI+): t_R = 1.40 min; *m*/*z* calcd for $C_{23}H_{20}F_2N_3O_3S$ $[M + H]^+$: 456.1; found: 456.1

N-(3,5-difluoro-4-{[1-(4-methylbenzene-1-sulfonyl)-3-(prop-1-en-2-yl)-1H-pyrrolo[2,3-b]pyridin-4-yl]oxy}phenyl)-N'-[(3-methyloxetan-3-yl)methyl]urea (S74)

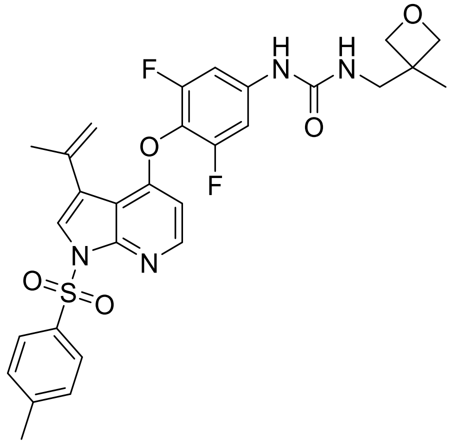

To a stirred solution of 3,5-difluoro-4-{[1-(4-methylbenzene-1-sulfonyl)-3-(prop-1-en-2-yl)-1H-pyrrolo[2,3-b]pyridin-4-yl]oxy}aniline (1.23 g, 53 % purity, 1.43 mmol, **S73**) in dichloromethane (15 mL) was added pyridine (15 ml, 190 mmol) followed by 3-(isocyanatomethyl)-3-methyloxetane (610 µl, 7.2 mmol). The resulting mixture was stirred for 4h at 60°C at which time water was added and the layers separated. The aqueous phase was extracted 3 times and the combined organic layers were washed with brine, dried over magnesium sulfate, filtered and evaporated under reduced pressure to afford the crude

product. The crude product was purified by preparative HPLC to afford the title product (480 mg, 56% over 3 steps).

Method 1, UPLC-MS (ESI+): t_R = 1.37 min; *m*/*z* calcd for $C_{29}H_{29}F_2N_4O_5S$ $[M + H]^+$: 583.2; found: 583.2

^{1}H-NMR (400 MHz, DMSO-d_6) δ [ppm] = 1.22 (s, 3H), 2.17 (s, 3H), 2.36 (s, 3H), 3.28 – 3.30 (m, 2H), 4.20 (d, *J* = 5.8 Hz, 2H), 4.37 (d, *J* = 5.8 Hz, 2H), 5.21 – 5.22 (m, 1H), 5.43 – 5.44 (m, 1H), 6.58 (d, 1H), 6.73 (t, 1H), 7.37 – 7.39 (m, 2H), 7.43 – 7.45 (m, 2H), 7.81 (s, 1H), 8.03 – 8.05 (m, 2H), 8.20 – 8.21 (m, 1H), 9.04 (br s, 1H).

N-(3,5-difluoro-4-{[1-(4-methylbenzene-1-sulfonyl)-3-(1-methylcyclopropyl)-1H-pyrrolo[2,3-b]pyridin-4-yl]oxy}phenyl)-N'-[(3-methyloxetan-3-yl)methyl]urea (S75)

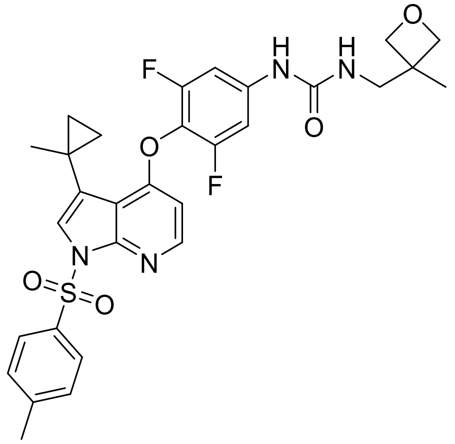

To a stirred solution of N-(3,5-difluoro-4-{[1-(4-methylbenzene-1-sulfonyl)-3-(prop-1-en-2-yl)-1H-pyrrolo[2,3-b]pyridin-4-yl]oxy}phenyl)-N'-[(3-methyloxetan-3-yl)methyl]urea (355 mg, 609 µmol, **S74**) in dimethyl sulfoxide (6.5 mL) was added N,N-diethylethanaminium bis[benzene-1,2-diolato(2-)-kappa^{2}O^1,O^2](iodomethyl)silicate(1-) (594 mg, 1.22 mmol, CAS No. 2230030-49-4)[59] and 1,2,3,5-Tetrakis(carbazol-9-yl)-4,6-dicyanobenzene, 2,4,5,6-Tetrakis(9H-carbazol-9-yl) isophthalonitrile (4CzIPN, 14.4 mg, 18.3 µmol, CAS No: 1416881-52-1). The reaction mixture was purged with argon for 5 minutes after which time the reaction was placed in a water bath and irradiated with two Kessil-lamps. After 16 hours the reaction mixture was diluted with ethyl acetate and a 1M aqueous solution of sodium hydroxide was added. The layers were separated and the aqueous phase was extracted twice with ethyl acetate. The combined organic phases were washed with brine, dried over magnesium sulfate, and evaporated under reduced pressure to afford the crude product. The crude product was used without further purification.

Method 1, UPLC-MS (ESI+): t_R = 1.39 min; m/z calcd for $C_{30}H_{31}F_2N_4O_5S$ $[M + H]^+$: 597.2; found: 597.3

N-(3,5-difluoro-4-{[3-(1-methylcyclopropyl)-1H-pyrrolo[2,3-b]pyridin-4-yl]oxy}phenyl)-N'-[(3-methyloxetan-3-yl)methyl]urea (S76)

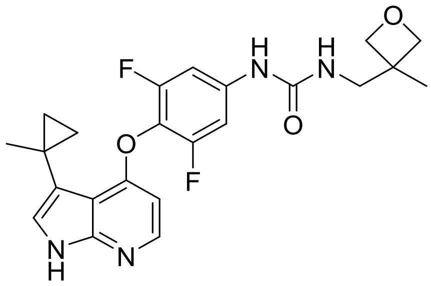

To a stirred solution of N-(3,5-difluoro-4-{[1-(4-methylbenzene-1-sulfonyl)-3-(1-methylcyclopropyl)-1H-pyrrolo[2,3-b]pyridin-4-yl]oxy}phenyl)-N'-[(3-methyloxetan-3-yl)methyl]urea (380 mg, 98 % purity, 624 µmol, **S75**) in methanol (10 mL) was added sodium hydroxide (99.9 mg, 2.50 mmol). The resulting mixture was stirred at room temperature for 16 hours, at which time water and ethyl acetate were added. The layers were separated, and the aqueous phase was extracted twice with ethyl acetate. The combined organic phases were washed with brine, dried over magnesium sulfate, filtered and evaporated under reduced pressure to give the crude product which was used without further purification.

Method 1, UPLC-MS (ESI+): t_R = 1.08 min; m/z calcd for $C_{23}H_{25}F_2N_4O_3$ $[M + H]^+$: 443.2; found: 443.3

<u>Compound 27:</u>

[(*rac*)-2-(3,5-difluoro-4-{[3-(1-methylcyclopropyl)-1H-pyrrolo[2,3-b]pyridin-4-yl]oxy}anilino)-5-methyl-5,6-dihydro-4H-1,3-oxazin-5-yl]methanol

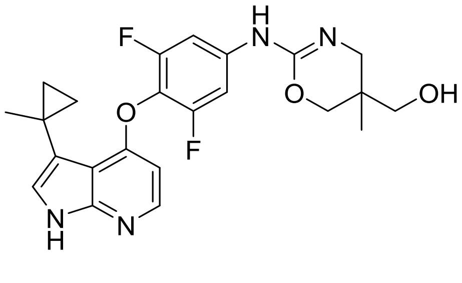

To a stirred solution of N-(3,5-difluoro-4-{[3-(1-methylcyclopropyl)-1H-pyrrolo[2,3-b]pyridin-4-yl]oxy}phenyl)-N'-[(3-methyloxetan-3-yl)methyl]urea (166 mg, 375 µmol, **S76**) in dichloromethane (2.3 mL) was added trifluoroacetic acid (230 µl, 3.0 mmol). The resulting mixture was stirred for 2 hours at room temperature at which time the mixture was diluted

with water and a saturated aqueous solution of sodium bicarbonate was added. The layers were separated, and the aqueous phase was extracted twice with ethyl acetate. The combined organic phases were washed with brine, dried over magnesium sulfate, and evaporated under reduced pressure to give the crude product. The crude product was purified by preparative HPLC to afford the title product (20mg, 1% yield over 3 steps)

Method 1, UPLC-MS (ESI+): t_R = 1.11 min; *m/z* calcd for $C_{23}H_{25}F_2N_4O_3$ $[M + H]^+$: 443.2; found: 443.5

^{1}H-NMR (400 MHz, DMSO-d_6) δ [ppm] = 0.59 – 0.61 (m, 2H), 0.80 – 0.82 (m, 2H), 0.91 (s, 3H), 1.43 (s, 3H), 2.98 – 3.04 (m, 1H), 3.19 – 3.28 (m, 3H), 3.86 – 3.89 (m, 1H), 4.05 – 4.07 (m, 1H), 4.83 (br s, 1H), 6.19 – 6.20 (m, 1H), 7.16 (d, *J* = 2.3 Hz, 1H), 7.45 – 7.63 (br m, 1H), 7.98 (d, *J* = 5.3 Hz, 1H), 11.41 (br s, 1H).

4-(2,6-difluoro-4-nitro-phenoxy)-1*H*-pyrrolo[2,3-*b*]pyridine-3-carbaldehyde (S77)

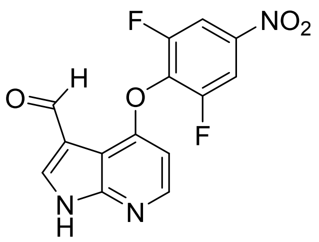

A mixture of 4-(2,6-difluoro-4-nitro-phenoxy)-1*H*-pyrrolo[2,3-*b*]pyridine (47.5 g, 163.11 mmol, **S18**) and hexamethylenetetramine (38 g, 271.07 mmol) in acetic acid (170 mL) and H_2O (340 mL) was stirred at 100 °C for 16 h. LC-MS indicated desired mass was detected. The mixture was cooled to rt. H_2O (300 mL) was added and the mixture was stirred at rt for 10 min. Then the mixture was filtered. The cake was slurried with H_2O (500 mL) and dried in vacuum to afford 4-(2,6-difluoro-4-nitro-phenoxy)-1*H*-pyrrolo[2,3-*b*]pyridine-3-carbaldehyde (31.5 g, 60.5% yield) as a yellow solid.

^{1}H NMR (400 MHz, DMSO-*d_6*) δ [ppm] = 13.05 (s, 1H), 10.12 (s, 1H), 8.44-8.38 (m, 3H), 8.24 (d, 1H), 6.75 (d, 1H).

4-(2,6-difluoro-4-nitrophenoxy)-1-tosyl-1*H*-pyrrolo[2,3-*b*]pyridine-3-carbaldehyde (S78)

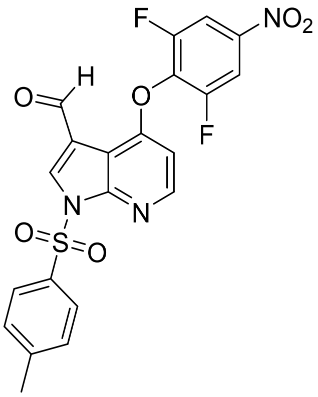

To a solution of 4-(2,6-difluoro-4-nitro-phenoxy)-1*H*-pyrrolo[2,3-*b*]pyridine-3-carbaldehyde (20 g, 62.65 mmol, **S77**) in DMF (200 mL) was added NaH (3 g, 75.01 mmol, 60% in mineral oil) at 0 °C. The mixture was stirred at 0 °C for 10 min. 4-methylbenzenesulfonyl chloride (12 g, 62.94 mmol) was added and the resulting mixture was stirred at rt for 2 h. TLC (PE: EA = 2: 1) indicated the reaction completed. The mixture was cooled to 0 °C. H_2O (800 mL) was added and the mixture was filtered. The filter cake was slurried with EtOH (250 mL) to give 4-(2,6-difluoro-4-nitrophenoxy)-1-tosyl-1*H*-pyrrolo[2,3-*b*]pyridine-3-carbaldehyde (21.6 g, 72.8% yield) as a yellow solid.

^{1}H NMR (400 MHz, DMSO-d_6) δ [ppm] = 10.16 (s, 1H), 8.5 (s, 1H), 8.41-8.35 (m, 3H), 8.13 (d, 2H), 7.48 (d, 2H), 6.97 (d, 1H), 2.38 (s, 3H).

[4-(2,6-difluoro-4-nitro-phenoxy)-1-tosyl-1*H*-pyrrolo[2,3-*b*]pyridin-3-yl]methanol (S79)

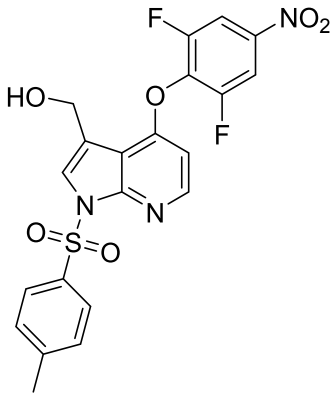

To a solution of 4-(2,6-difluoro-4-nitrophenoxy)-1-tosyl-1*H*-pyrrolo[2,3-*b*]pyridine-3-carbaldehyde (21.6 g, 45.63 mmol, **S78**) in THF (200 mL) and H_2O (40 mL) was added $NaBH_4$ (1.8 g, 47.58 mmol) at rt. The mixture was stirred at rt for 1 h. TLC (PE: EA = 2: 1) indicated the reaction completed. Brine (300 mL) was added and the mixture was extracted with EA

(300 mL x 2). The organic phase was dried over Na_2SO4, filtered and concentrated by evaporation in vacuum. The residue was purified by silica gel chromatography (20% to 35% of EA in PE) to give [4-(2,6-difluoro-4-nitro-phenoxy)-1-tosyl-1*H*-pyrrolo[2,3-*b*]pyridin-3-yl]methanol (11.8 g, 54.4% yield) as a yellow solid.

^{1}H NMR (400 MHz, DMSO-d_6) δ [ppm] = 8.46-8.35 (m, 2H), 8.24 (d, 1H), 8.01 (d, 2H), 7.44 (s, 1H), 7.42 (d, 2H), 6.75 (d, 1H), 5.28 (s, 1H), 4.75 (s, 2H), 2.35 (s, 3H).

2-[4-(2,6-difluoro-4-nitrophenoxy)-1-tosyl-1*H*-pyrrolo[2,3-*b*]pyridin-3-yl]acetonitrile (S80)

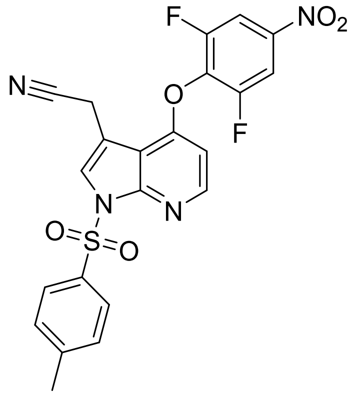

To a mixture of [4-(2,6-difluoro-4-nitro-phenoxy)-1-tosyl-1*H*-pyrrolo[2,3-*b*]pyridin-3-yl]methanol (11.8 g, 24.82 mmol, **S79**), 2-hydroxy-2-methylpropanenitrile (4.5 mL g, 49.3 mmol) and ADDP (12.5 g, 49.54 mmol) in THF (200 mL) was added tributylphosphine (12 mL, 48.64 mmol) at 0 °C. Then the mixture was stirred at rt for 1 h. The mixture was concentrated by evaporation in vacuum. Brine (300 mL) was added and the mixture was extracted with EA (500 mL). The organic phase was dried over Na_2SO_4, filtered and concentrated by evaporation in vacuum. The residue was purified by silica gel chromatography (10% to 30% of EA in PE) to give 2-[4-(2,6-difluoro-4-nitrophenoxy)-1-tosyl-1*H*-pyrrolo[2,3-*b*]pyridin-3-yl]acetonitrile (7 g, 52.3% yield, 89.8% purity) as a yellow solid.

^{1}H NMR (400 MHz, DMSO-d_6) δ [ppm] = 8.43-8.38 (m, 2H), 8.30 (d, 1H), 8.04 (d, 2H), 7.98 (s, 1H), 7.44 (d, 2H), 6.85-6.77 (m, 1H), 4.21 (s, 2H), 2.36 (s, 3H).

1-(4-(2,6-difluoro-4-nitrophenoxy)-1-tosyl-1*H*-pyrrolo[2,3-*b*]pyridin-3-yl)cyclopropanecarbonitrile (S81)

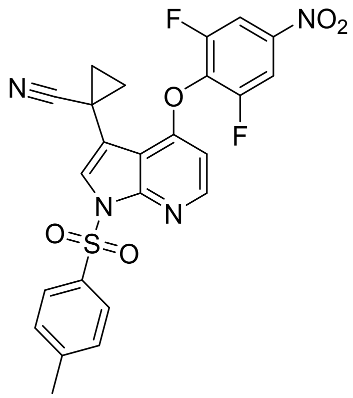

To a mixture of 2-(4-(2,6-difluoro-4-nitrophenoxy)-1-tosyl-1*H*-pyrrolo[2,3-*b*]pyridin-3-yl)acetonitrile (5.45 g, 89.8% purity, 10.1 mmol, **S80**) in DMF (60 mL) was added diphenyl(vinyl)sulfonium trifluoromethanesulfonate (6.8 g, 18.76 mmol) at 0 °C. The mixture was stirred for 10 min. Then DBU (8.5 mL, 56.39 mmol) was added and the resulting mixture was stirred at rt for 30 min. TLC (PE: EA = 3: 1) indicated the reaction completed. The reaction mixture was combined with another batch of reaction mixture (batch: EW4245-1095, 2 g (89% purity) of 2-[4-(2,6-difluoro-4-nitro-phenoxy)-1-tosyl-1*H*-pyrrolo[2,3-*b*]pyridin-3-yl]acetonitrile was used in this batch). The combined mixture was poured into H_2O (500 mL) and the mixture was extracted with EA (100 mL x 3). The combined organic phase was dried over Na_2SO_4, filtered and concentrated by evaporation in vacuum. The residue was purified by silica gel chromatography (PE: EA = 10: 1 to 3: 1) to give 1-(4-(2,6-difluoro-4-nitrophenoxy)-1-tosyl-1*H*-pyrrolo[2,3-*b*]pyridin-3-yl)cyclopropanecarbonitrile (5.5 g, 82% purity) as a yellow solid.

^{1}H NMR (400 MHz, DMSO-d_6) δ [ppm] = 8.41 (d, 2H), 8.29 (d, 1H), 8.06-8.04 (m, 3H), 7.46 (d, 2H), 6.91-6.81 (m, 1H), 2.35 (s, 3H), 1.66-1.56 (m, 4H).

1-(4-(4-amino-2,6-difluorophenoxy)-1-tosyl-1*H*-pyrrolo[2,3-*b*]pyridin-3-yl)cyclopropanecarbonitrile (S82)

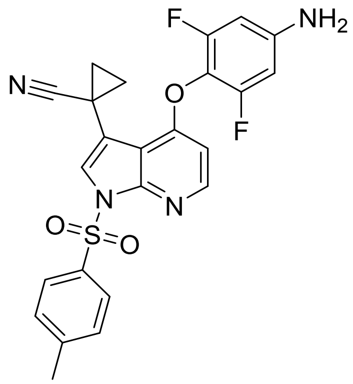

To a mixture of 1-(4-(2,6-difluoro-4-nitrophenoxy)-1-tosyl-1*H*-pyrrolo[2,3-*b*]pyridin-3-yl)cyclopropanecarbonitrile (4.3 g, 82% purity, 6.91 mmol, **S81**) in THF (50 mL) was added Pd/C (2 g, 10% purity, containing 50% of H_2O) and conc. HCl (0.2 mL) at rt. Then the mixture was stirred at rt for 16 h under H_2 (15 psi). LC-MS indicated desired mass was detected. The mixture was combined with another two batches of reaction mixtures (batch 1: EW4245-1098, 200 mg (82% purity) of 1-(4-(2,6-difluoro-4-nitrophenoxy)-1-tosyl-1*H*-pyrrolo[2,3-b]pyridin-3-yl)cyclopropanecarbonitrile was used; batch 2: EW4245-1099, 1 g (82% purity) of 1-(4-(2,6-difluoro-4-nitrophenoxy)-1-tosyl-1*H*-pyrrolo[2,3-b]pyridin-3-yl)cyclopropanecarbonitrile was used). The combined mixture was filtered, and the filter cake was washed with acetonitrile (100 mL). The combined filtrate was concentrated by evaporation in vacuum. The residue was purified by reversed phase (C18 330 g, A: H_2O (0.1% $NH_3{\cdot}H_2O$), B: MeCN, 55% B, 100 mL/min, 30 min) to give 1-(4-(4-amino-2,6-difluorophenoxy)-1-tosyl-1*H*-pyrrolo[2,3-*b*]pyridin-3-yl)cyclopropanecarbonitrile (3.57 g, 99+% purity) as a yellow solid.

^{1}H NMR (400 MHz, DMSO-d_6) δ [ppm] = 8.22 (d, 1H), 8.03 (d, 2H), 7.97 (s, 1H), 7.43 (d, 2H), 6.57 (d, 1H), 6.38 (d, 2H), 5.81 (s, 2H), 2.35 (s, 3H), 1.55-1.52 (m, 4H).

N-(4-{[3-(1-cyanocyclopropyl)-1-(4-methylbenzene-1-sulfonyl)-1H-pyrrolo[2,3-b]pyridin-4-yl]oxy}-3,5-difluorophenyl)-N'-[(3-methyloxetan-3-yl)methyl]urea (S83)

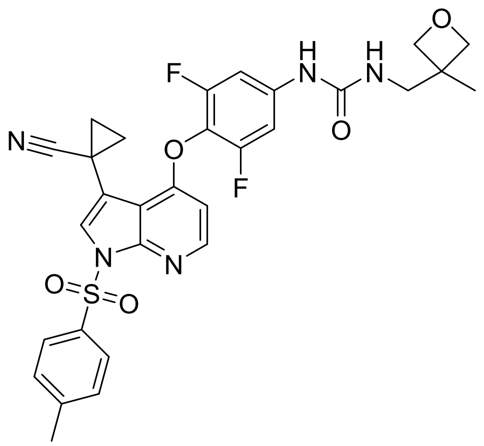

To a stirred solution of 1-[4-(4-amino-2,6-difluorophenoxy)-1-(4-methylbenzene-1-sulfonyl)-1H-pyrrolo[2,3-b]pyridin-3-yl]cyclopropane-1-carbonitrile (100 mg, 208 µmol, **S82**) in dichloromethane (1.8 mL) was added pyridine (1.9 mL, 23 mmol) followed by 3-(isocyanatomethyl)-3-methyloxetane (52.9 mg, 416 µmol). The resulting mixture was stirred at 60°C for 16 hours at which time toluene was added and the mixture was evaporated under reduced pressure to afford the crude product. The crude product was purified by flash column chromatography over silica gel to afford the title product (113mg, 85% yield).

Method 1, UPLC-MS (ESI+): t_R = 1.25 min; *m*/*z* calcd for $C_{30}H_{28}F_2N_5O_5S$ $[M + H]^+$: 608.2; found: 608.4

^{1}H-NMR (400 MHz, CHLOROFORM-d) δ [ppm] = 1.28 (s, 3H), 1.48 - 1.51 (m, 2H), 1.70 – 1.73 (m, 2H), 2.40 (s, 3H), 3.41 (d, *J* = 6.1 Hz, 2H), 4.37 – 4.39 (m, 2H), 4.46 – 4.48 (m, 2H), 5.35 (t, *J* = 5.8 Hz, 1H), 6.42 (d, *J* = 5.6 Hz, 1H), 7.08 – 7.10 (m, 3H), 7.31 – 7.33 (br d, 2H), 7.60 (s, 1H), 8.10 (br d, 2H), 8.22 - 8.24 (m, 1H).

1-[4-(2,6-difluoro-4-{[(5R)-5-(hydroxymethyl)-5-methyl-5,6-dihydro-4H-1,3-oxazin-2-yl]amino}phenoxy)-1-(4-methylbenzene-1-sulfonyl)-1H-pyrrolo[2,3-b]pyridin-3-yl]cyclopropane-1-carbonitrile (S84)

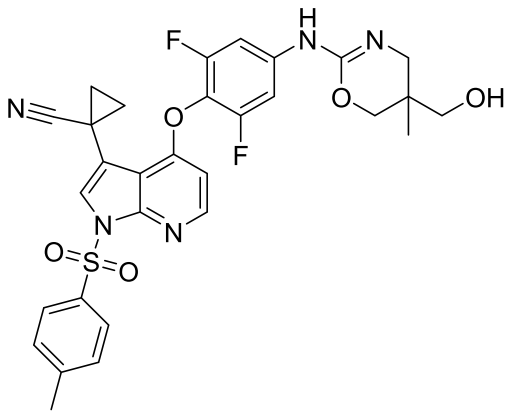

To a stirred solution of N-(4-{[3-(1-cyanocyclopropyl)-1-(4-methylbenzene-1-sulfonyl)-1H-pyrrolo[2,3-b]pyridin-4-yl]oxy}-3,5-difluorophenyl)-N'-[(3-methyloxetan-3-yl)methyl]urea (110 mg, 181 µmol, **S83**) in dichloromethane (1.2 mL) was added trifluoroacetic acid (140 µl, 1.8 mmol). The resulting mixture was stirred for 5 hours at room temperature at which time the reaction mixture was evaporated under reduced pressure. The resulting residue was dissolved in acetonitrile and an aqueous solution of ammonia in water (33%) was added. The resulting mixture was stirred for 2 hours at room temperature at which time the solvent was evaporated under reduced pressure. The residue was dissolved in ethyl acetate, washed twice with water, then brine, dried over sodium sulfate, filtered and evaporated under reduced pressure to afford the crude product which was used without further purification.

Method 1, UPLC-MS (ESI+): t_R = 1.23 min; *m*/*z* calcd for $C_{30}H_{28}F_2N_5O_5S$ $[M + H]^+$: 608.2; found: 608.8

__Compound 28__:

1-[4-(2,6-difluoro-4-{[(5R)-5-(hydroxymethyl)-5-methyl-5,6-dihydro-4H-1,3-oxazin-2-yl]amino}phenoxy)-1H-pyrrolo[2,3-b]pyridin-3-yl]cyclopropane-1-carbonitrile

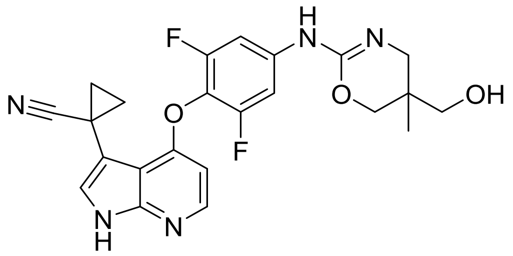

To a stirred solution of 1-[4-(2,6-difluoro-4-{[(5R)-5-(hydroxymethyl)-5-methyl-5,6-dihydro-4H-1,3-oxazin-2-yl]amino}phenoxy)-1-(4-methylbenzene-1-sulfonyl)-1H-pyrrolo[2,3-b]pyridin-3-yl]cyclopropane-1-carbonitrile (116 mg, 191 µmol, **S84**) in methanol (3.1 mL) was

added sodium hydroxide (15.3 mg, 0.38 mmol). The resulting mixture was stirred at room temperature for 2 hours at which time water and ethyl acetate were added. The layers were separated, and the aqueous phase was extracted twice with ethyl acetate. The combined organic layers were washed with brine, dried over sodium sulfate, filtered, and evaporated under reduced pressure to afford the crude product. The crude product was purified by flash column chromatography over silica gel (eluent: 9:1 dichloromethane : methanol) to afford the title product (18 mg, 22% yield over 2 steps).

Method 1, UPLC-MS (ESI+): t_R = 0.90 min; *m/z* calcd for $C_{23}H_{22}F_2N_5O_3$ $[M + H]^+$: 454.2; found: 454.3

^1H NMR (400 MHz, DMSO-d_6) δ [ppm] = 0.91 (s, 3H), 1.39 - 1.42 (m, 2H), 1.57 – 1.60 (m, 2H), 2.99 – 3.02 (m, 1H), 3.16 (br s, 2H), 3.19 – 3.27 (m, 2H), 3.88 (d, *J* = 10.1 Hz, 1H), 4.06 (d, *J* = 10.4 Hz, 1H), 4.82 – 4.86 (br m, 1H), 6.31 (d, *J* = 5.6 Hz, 1H), 7.51 (s, 1H), 8.05 (d, *J* = 5.6 Hz, 1H), 11.86 – 11.89 (br m, 1H).

1-[4-(2,6-difluoro-4-nitrophenoxy)-1H-pyrrolo[2,3-b]pyridin-3-yl]-2,2-difluoroethan-1-one (S85)

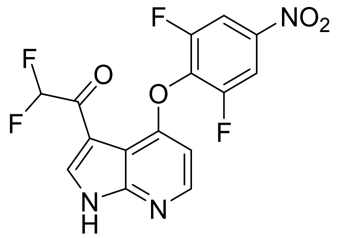

To a stirred solution of aluminum trichloride (8.0 g, 60 mmol) in 1,2-dichloroethane (100 mL) was added difluoro-acetic anhydride (3.7 ml, 30 mmol) dropwise. The resulting mixture was stirred at room temperature for 10 min, at which time a solution of 4-(2,6-difluoro-4-nitrophenoxy)-1H-pyrrolo[2,3-b]pyridine (5.00 g, 17.2 mmol, **S18**) in 1,2-dichloroethane (5 mL) was added dropwise over 2 minutes. The resulting mixture was stirred at room temperature for 5 hours at which time the mixture was poured into a beaker containing ice water. The obtained precipitate was filtered, washed with water and dried. The filtrate layers were separated, and the aqueous phase was extracted two times with dichloromethane. The combined organic phases were washed with brine, dried over sodium sulfate, and evaporated under reduced pressure. The resulting residue and filter cake were combined and stirred in ether for 30 minutes. The precipitate was filtered, washed with ether and dried to afford the crude product as a yellow solid which was used without further purification.

Method 1, UPLC-MS (ESI+): t_R = 0.83 min; *m/z* calcd for $C_{15}H_8F_4N_3O_4$ $[M + H]^+$: 370.0; found: 370.2

1-[4-(2,6-difluoro-4-nitrophenoxy)-1-{[2-(trimethylsilyl)ethoxy]methyl}-1H-pyrrolo[2,3-b]pyridin-3-yl]-2,2-difluoroethan-1-one (S86)

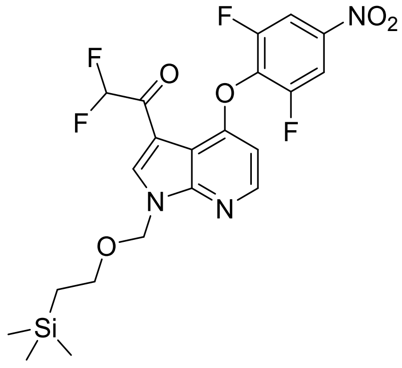

To a cooled (0°C), stirred solution of 1-[4-(2,6-difluoro-4-nitrophenoxy)-1H-pyrrolo[2,3-b]pyridin-3-yl]-2,2-difluoroethan-1-one (4.92 g, 13.3 mmol, **S85**) in acetonitrile (150 mL) was added N,N-diisopropylethylamine (3.5 ml, 20 mmol) followed by [2-(chloromethoxy)ethyl](trimethyl)silane (2.6 ml, 15 mmol). The resulting mixture was stirred at room temperature for 1 hour at which time the mixture was poured into a beaker containing ice water. The obtained precipitate was filtered and dried to obtain the crude product which was used without further purification.

Method 1, UPLC-MS (ESI+): t_R = 1.51 min; *m/z* calcd for $C_{21}H_{22}F_4N_3O_5Si$ $[M + H]^+$: 500.1; found: 500.2

4-(2,6-difluoro-4-nitrophenoxy)-3-(3,3-difluoroprop-1-en-2-yl)-1-{[2-(trimethylsilyl)ethoxy]methyl}-1H-pyrrolo[2,3-b]pyridine (S87)

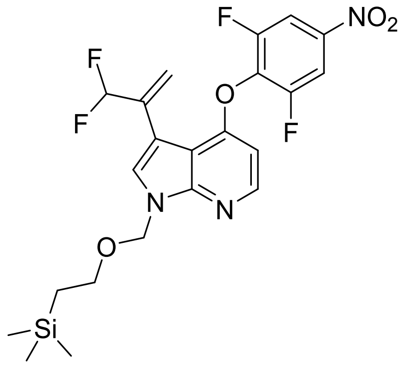

To a stirred solution of 1 methyl(triphenyl)phosphonium bromide (9.01 g, 25.2 mmol) in tetrahydrofuran (120 mL) was added n-butyllithium (10 mL, 2.5 M in hexane, 25 mmol). The

resulting mixture was stirred at room temperature for 30 minutes at which time a solution of 1-[4-(2,6-difluoro-4-nitrophenoxy)-1-{[2-(trimethylsilyl)ethoxy]methyl}-1H-pyrrolo[2,3-b]pyridin-3-yl]-2,2-difluoroethan-1-one (6.30 g, 12.6 mmol, **S86**) in tetrahydrofuran (5 mL) was added. The reaction mixture was stirred at room temperature for 1 hour at which time the mixture was poured into a saturated aqueous solution of ammonium chloride. The aqueous phase was extracted three times with ethyl acetate, and the combined organic phases were washed with brine, dried over sodium sulfate, filtered, and evaporated under reduced pressure to afford the crude product. The crude product was purified by flash column chromatography over silica gel to afford the product (2.63 g, 42% yield over 3 steps) as a yellow solid.

Method 1, UPLC-MS (ESI+): t_R = 1.61 min; *m/z* calcd for $C_{22}H_{24}F_4N_3O_4Si$ $[M + H]^+$: 498.1; found: 498.4

4-{[3-(3,3-difluoroprop-1-en-2-yl)-1-{[2-(trimethylsilyl)ethoxy]methyl}-1H-pyrrolo[2,3-b]pyridin-4-yl]oxy}-3,5-difluoroaniline (S88)

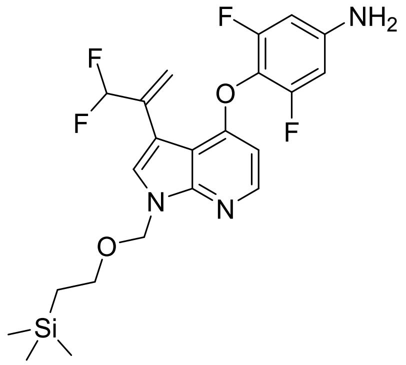

To a stirred suspension of ammonium chloride (839 mg, 15.7 mmol) and iron powder (876 mg, 15.7 mmol) was added a solution of 4-(2,6-difluoro-4-nitrophenoxy)-3-(3,3-difluoroprop-1-en-2-yl)-1-{[2-(trimethylsilyl)ethoxy]methyl}-1H-pyrrolo[2,3-b]pyridine (1.56 g, 3.14 mmol, **S87**) in a mixture of methanol (20 mL) and tetrahydrofuran (20 mL). The resulting mixture was stirred at 80°C for 3h, at which time the mixture was cooled and filtered. The filtrate was extracted two times with ethyl acetate and the combined organic phases were washed with brine, dried over sodium sulfate, filtered and evaporated under reduced pressure to afford the crude product which was used without further purification.

Method 1, UPLC-MS (ESI+): t_R = 1.51 min; *m/z* calcd for $C_{22}H_{26}F_4N_3O_2Si$ $[M + H]^+$: 468.2; found: 468.3

di-tert-butyl (4-{[3-(3,3-difluoroprop-1-en-2-yl)-1-{[2-(trimethylsilyl)ethoxy]methyl}-1H-pyrrolo[2,3-b]pyridin-4-yl]oxy}-3,5-difluorophenyl)-2-imidodicarbonate (S89)

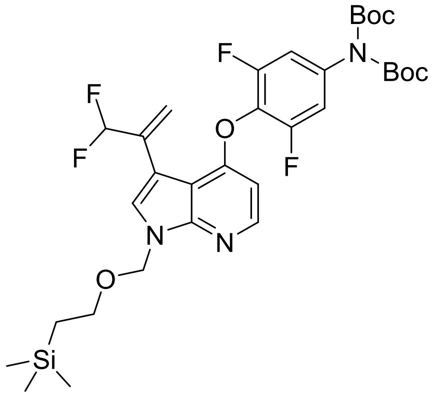

To a stirred solution of 4-{[3-(3,3-difluoroprop-1-en-2-yl)-1-{[2-(trimethylsilyl)ethoxy]methyl}-1H-pyrrolo[2,3-b]pyridin-4-yl]oxy}-3,5-difluoroaniline (1.20 g, 2.57 mmol, **S88**) in tetrahydrofuran (50 mL) was added 4-dimethylaminopyridine (31.4 mg, 0.26 mmol) followed by di-tert-butyl dicarbonate (1.6 ml, 7.7 mmol). The reaction mixture was stirred at 75°C for 16 hours at which time the reaction was diluted with ethyl acetate and water was added. The layers were separated, and the aqueous phase was extracted two times with ethyl acetate. The combined organic phases were washed with brine, dried over sodium sulfate, filtered and evaporated under reduced pressure to afford the crude product. The crude product was purified by flash column chromatography over silica gel followed by further purification by preparative HPLC to afford the title product (534 mg, 29% yield over two steps) as a colourless solid.

Method 1, UPLC-MS (ESI+): t_R = 1.75 min; *m/z* calcd for $C_{32}H_{42}F_4N_3O_6Si$ $[M + H]^+$: 668.3; found: 668.7

^{1}H NMR (400 MHz, DMSO-d_6) δ [ppm] = -0.09 (s, 9H), 0.81 – 0.85 (m, 2H), 1.42 (s, 18H), 3.54 – 3.58 (m, 2H), 5.67 (s, 2H), 5.84 (br s, 1H), 5.90 (br s, 1H), 6.28 (d, *J* = 5.6 Hz, 1H), 6.83 (t, *J* = 56 Hz, 1H), 7.51 – 7.54 (m, 2H), 7.81 (br s, 1H), 8.22 (d, *J* = 5.6 Hz, 1H)

di-tert-butyl {4-[(3-[1-(difluoromethyl)cyclopropyl]-1-{[2-(trimethylsilyl)ethoxy]methyl}-1H-pyrrolo[2,3-b]pyridin-4-yl)oxy]-3,5-difluorophenyl}-2-imidodicarbonate (S90)

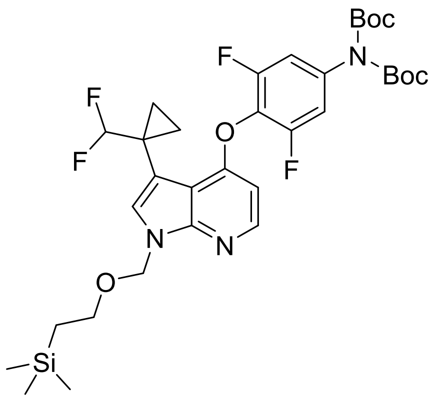

A stirred solution of di-tert-butyl (4-{[3-(3,3-difluoroprop-1-en-2-yl)-1-{[2-(trimethylsilyl)ethoxy]methyl}-1H-pyrrolo[2,3-b]pyridin-4-yl]oxy}-3,5-difluorophenyl)-2-imidodicarbonate (600 mg, 92 % purity, 827 µmol, **S89**), N,N-diethylethanaminium bis[benzene-1,2-diolato(2-)-kappa^{2}O^1,O^2](iodomethyl)silicate(1-) (927 mg, 1.90 mmol, CAS No. 2230030-49-4)[59], and 1,2,3,5-Tetrakis(carbazol-9-yl)-4,6-dicyanobenzene, 2,4,5,6-Tetrakis(9H-carbazol-9-yl) isophthalonitrile (4CzIPN, 19.6 mg, 25 µmol, CAS No. 1416881-52-1) in dimethyl sulfoxide (12 mL) was degassed with argon. The reaction mixture was placed in a water batch and irradiated with 2 Kessil lamps for 12 hours at which time a 1M aqueous solution of sodium hydroxide was added and the layers were separated. The aqueous phase was extracted two times with ethyl acetate and the combined organic phases were washed with a 1M aqueous solution of sodium hydroxide, followed by brine, then dried over sodium sulfate, filtered, and evaporated under reduced pressure to afford the crude product. The crude product was purified by flash column chromatography over silica gel to afford the title product (262 mg, 46% yield, ~70% pure) as a yellow solid which was used directly in the next without further purification.

Method 1, UPLC-MS (ESI+): t_R = 1.77 min; *m/z* calcd for $C_{33}H_{44}F_4N_3O_6Si$ $[M + H]^+$: 682.3; found: 682.7

4-[(3-[1-(difluoromethyl)cyclopropyl]-1-{[2-(trimethylsilyl)ethoxy]methyl}-1H-pyrrolo[2,3-b]pyridin-4-yl)oxy]-3,5-difluoroaniline (S91)

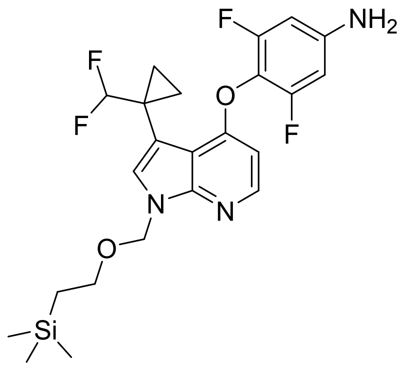

To a stirred solution of di-tert-butyl {4-[(3-[1-(difluoromethyl)cyclopropyl]-1-{[2-(trimethylsilyl)ethoxy]methyl}-1H-pyrrolo[2,3-b]pyridin-4-yl)oxy]-3,5-difluorophenyl}-2-imidodicarbonate (200 mg, 293 µmol, **S90**) in 1,2-dichloroethane (20 mL) was added zinc bromide (132 mg, 587 µmol; CAS No: 7699-45-8). The resulting mixture was stirred at room temperature for 16 hours, at which time dichloromethane and water were added and the layers were separated. The aqueous phase was extracted with dichloromethane two times, and the combined organic phases were washed with brine, dried over sodium sulfate, filtered, and evaporated under reduced pressure to give the crude product. The crude product was purified by preparative HPLC to afford the title product (45 mg, 32% yield).

Method 1, UPLC-MS (ESI+): t_R = 1.53 min; *m*/*z* calcd for $C_{23}H_{28}F_4N_3O_2Si$ $[M + H]^+$: 485.2; found: 482.5

^1H NMR (400 MHz, DMSO-d_6) δ [ppm] = -0.11 (s, 9H), 0.78 – 0.82 (m, 2H), 1.00 – 1.01 (br s, 2H), 1.12 – 1.15 (m, 2H), 3.50 – 3.54 (m, 2H), 5.57 (br s, 2H), 5.79 (br s, 2H), 6.04 (t, *J* = 60 Hz, 1H), 6.33 – 6. 34 (m, 1H), 6.37 – 6.41 (m, 2H), 7.57 (s, 1H), 8.09 – 8.11 (m, 1H)

N-{4-[(3-[1-(difluoromethyl)cyclopropyl]-1-{[2-(trimethylsilyl)ethoxy]methyl}-1H-pyrrolo[2,3-b]pyridin-4-yl)oxy]-3,5-difluorophenyl}-N'-[(3-methyloxetan-3-yl)methyl]urea (S92)

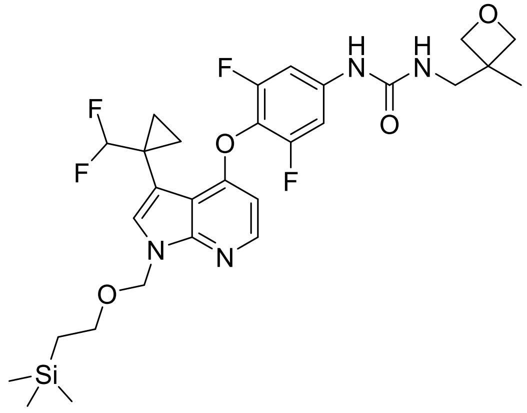

To a stirred solution of 4-[(3-[1-(difluoromethyl)cyclopropyl]-1-{[2-(trimethylsilyl)ethoxy]methyl}-1H-pyrrolo[2,3-b]pyridin-4-yl)oxy]-3,5-difluoroaniline (45.0 mg, 93.4 µmol, **S91**) in a mixture of pyridine (1.0 ml, 12 mmol) in dichloromethane (1.0 mL) was added 3-(isocyanatomethyl)-3-methyloxetane (59.4 mg, 467 µmol). The resulting mixture was stirred at 60°C for 2 hours at which time the mixture was cooled, water was added and the layers were separated. The aqueous phase was extracted two times with ethyl acetate and the combined organic phases were washed with brine, dried over sodium sulfate, and evaporated under reduced pressure to give the crude product. The crude product was used without further purification.

Method 1, UPLC-MS (ESI+): t_R = 1.50 min; *m/z* calcd for $C_{29}H_{37}F_4N_4O_4Si$ $[M + H]^+$: 609.2; found: 609.8

__**Compound 29**__:

{(5S)-2-[4-({3-[1-(difluoromethyl)cyclopropyl]-1H-pyrrolo[2,3-b]pyridin-4-yl}oxy)-3,5-difluoroanilino]-5-methyl-5,6-dihydro-4H-1,3-oxazin-5-yl}methanol

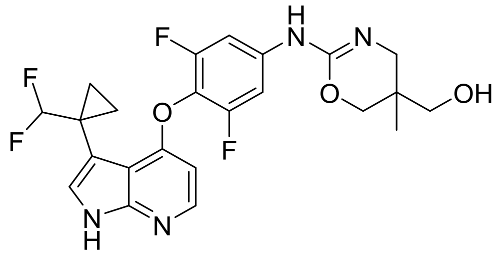

To a stirred solution of N-{4-[(3-[1-(difluoromethyl)cyclopropyl]-1-{[2-(trimethylsilyl)ethoxy]methyl}-1H-pyrrolo[2,3-b]pyridin-4-yl)oxy]-3,5-difluorophenyl}-N'-[(3-methyloxetan-3-yl)methyl]urea (55.0 mg, 90.4 µmol, **S92**) in dichloromethane (1.0 mL) was added trifluoro-acetatic acid (500 µl, 6.5 mmol). The resulting mixture was stirred at room temperature for 4 hours at which time the mixture was neutralized with a 2M aqueous solution of sodium hydroxide and then extracted 2 times with ethyl acetate. The combined organic phases were washed with brine, dried over sodium sulfate, and evaporated under reduced pressure. The resulting residue was dissolved in acetonitrile and treated with a 25% aqueous solution of ammonia (1 mL) for 1 hour to afford the crude product. The crude mixture was then purified by preparative HPLC to afford the title product (23 mg, 51% yield over two steps) as a white solid.

Method 1, UPLC-MS (ESI+): t_R = 1.04 min; *m/z* calcd for $C_{23}H_{23}F_4N_4O_3$ $[M + H]^+$: 479.2; found: 479.5

^{1}H NMR (400 MHz, DMSO-*d$_6$*) δ [ppm] = 0.90 (s, 3H), 1.01 – 1.02 (br s, 2H), 1.09 – 1.12 (m, 2H), 3.00 (d, *J* = 14.5 Hz, 1H), 3.21 (d, *J* = 14.5 Hz, 1H), 3.24 – 3.28 (m, 2H), 3.87 (d, *J* = 10.4 Hz, 1H) 4.06 (d, *J* = 10.4 Hz, 1H), 6.04 (t, *J* = 56 Hz, 1H), 6.25 (d, *J* = 5.3 Hz, 1H), 7.27 – 7.43 (br s, 2H), 7.38 (s, 1H), 8.02 (d, *J* = 5.6 Hz, 1H), 11.78 (br s, 1H)

N-{3,5-difluoro-4-[(3-[1-(trifluoromethyl)cyclopropyl]-1-{[2-(trimethylsilyl)ethoxy]methyl}-1H-pyrrolo[2,3-b]pyridin-4-yl)oxy]phenyl}-N'-[(3-methyloxetan-3-yl)methyl]urea (S93)

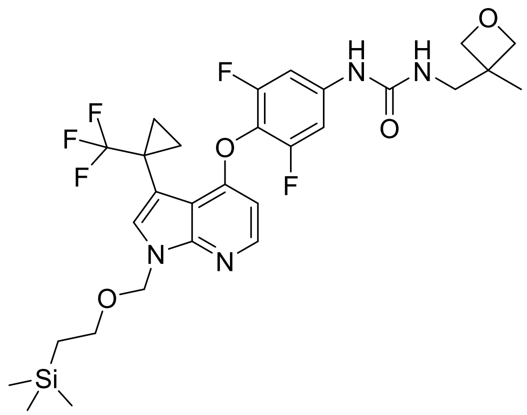

3,5-difluoro-4-[(3-[1-(trifluoromethyl)cyclopropyl]-1-{[2-(trimethylsilyl)ethoxy]methyl}-1H-pyrrolo[2,3-b]pyridin-4-yl)oxy]aniline (100 mg, 200 µmol, intermediate **47**) was dissolved in dichloromethane (1.7 mL) and 3-(isocyanatomethyl)-3-methyloxetane (CAS No: 1260665-88-0, 50.9 mg, 400 µmol) and pyridine (1.8 mL) were added. The mixture was stirred overnight at 60°C in a closed microwave vial. The crude was diluted with toluene and the solvent evaporated. The resulting residue was purified by silica gel chromatography using a Biotage system to yield the title compound (127 mg, 96% yield).

Method 1, UPLC-MS (ESI+): t_R = 1.51 min; *m/z* calcd for $C_{29}H_{36}F_5N_4O_4Si$ $[M + H]^+$: 627.2; found: 627.6

^{1}H-NMR (400 MHz, $CDCl_3$) δ [ppm] = -0.06 (s, 9H), 0.90 (m, 2H), 1.20 (m, 2H), 1.33 (s, 3H), 1.40 (m, 2H), 3.46 (m, 2H), 3.57 (m, 2H), 4.48 (d, 2H), 4.52 (d, 2H), 5.21 (m, 1H), 5.64 (s, 2H), 6.31 (m, 1H), 6.72 (m, 1H), 7.15 (m, 2H), 7.35 (s, 1H), 8.12 (d, 1H).

<u>Compound 30:</u>

(+/-)-{2-[3,5-difluoro-4-({3-[1-(trifluoromethyl)cyclopropyl]-1H-pyrrolo[2,3-b]pyridin-4-yl}oxy)anilino]-5-methyl-5,6-dihydro-4H-1,3-oxazin-5-yl}methanol

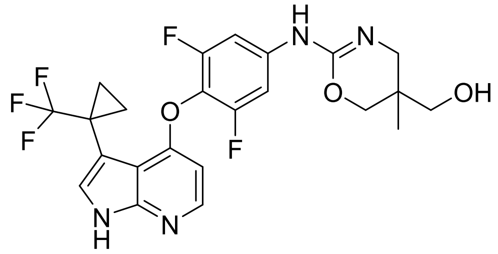

To a stirred solution of N-{3,5-difluoro-4-[(3-[1-(trifluoromethyl)cyclopropyl]-1-{[2-(trimethylsilyl)ethoxy]methyl}-1H-pyrrolo[2,3-b]pyridin-4-yl)oxy]phenyl}-N'-[(3-methyloxetan-3-yl)methyl]urea (125 mg, 189 µmol, **S93**) in dichloromethane (6.1 mL) was added trifluoroacetic acid (630 µL, 8.1 mmol). The mixture was stirred under argon overnight at room temperature. The solvent was removed under vacuum, and the residue stirred with acetonitrile (6 mL) and a 33% ammonia solution (2 mL) at room temperature for 1h and then dried again. The residue was dissolved with ethyl acetate and washed with water (x2) and brine (x1). The residue was purified by column chromatography using a Biotage system and digested with diethyl ether and dichloromethane to yield the title compound (44.1 mg, 42 % yield).

Method 1, UPLC-MS (ESI+): t_R = 1.08 min; *m/z* calcd for $C_{23}H_{22}F_5N_4O_3$ $[M + H]^+$: 497.2; found: 497.4

^{1}H-NMR (400 MHz, DMSO-d6) δ [ppm]: 1.00 (s, 3H), 1.17 (m, 2H), 1.33 (m, 2H9, 3.11 (d, 1H), 4.30 (m, 2H), 4.48 (m, 2H), 5.14 (br s, 1H), 6.32 (d, 1H), 7.38 (m, 2H), 7.57 (d, 1H), 8.08 (d, 1H), 12.02 (d, 1H).

N-methoxy-N-methyl-1-(trifluoromethyl)cyclobutanecarboxamide (S94)

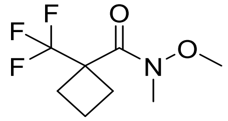

To a mixture of 1-(trifluoromethyl)cyclobutanecarboxylic acid (CAS No: 277756-45-3, 55 g, 327.16 mmol), N,O-dimethylhydroxylamine hydrochloride (38.5 g, 394.69 mmol) and 1-[bis(dimethylamino)methylene]-1H-1,2,3-triazolo[4,5-B]pyridinium-3-oxid-hexafluorophosphate (156.75 g, 412.25 mmol) in dichloromethane (1 L) was added triethylamine (150 mL, 1.08 mol) at room temperature under nitrogen. The mixture was stirred

at room temperature for 16 hours. The reaction mixture was concentrated by rotary evaporator under reduced pressure. The residue was diluted with *tert*-butyl methyl ether (1.5 L) and water (500 mL), and the two phases were separated. The organic phase was washed with 1 M hydrochloric acid (aq., 500 mL x 2), sat. aq. sodium bicarbonate (500 mL), brine (500 mL), dried over Sodium sulfate, filtered and concentrated under reduced pressure. The residue was purified by silica gel chromatography (*tert*-butyl methyl ether) to give N-methoxy-N-methyl-1-(trifluoromethyl)cyclobutanecarboxamide (58 g, 84% yield) as a light yellow oil.

^{1}H-NMR: (400 MHz, $CDCl_3$): δ [ppm] = 3.68 (s, 3H), 3.24 (s, 3H), 2.70-2.67 (m, 2H), 2.50-2.48 (m, 2H), 2.10-2.07 (m, 1H), 1.82-1.79 (m, 1H).

(2-fluoro-3-pyridyl)-[1-(trifluoromethyl)cyclobutyl]methanone (S95)

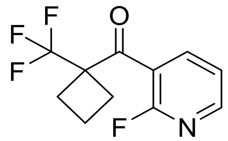

To a solution of 2,2,6,6-tetramethylpiperidine (110 mL, 647.91 mmol) in tetrahydrofuran (250 mL) was added n-BuLi (250 mL, 2.5 M in hexane) at -70 °C under nitrogen. The mixture was stirred at -70 °C for 1 hour. Then 2-fluoropyridine (CAS No: 372-48-5, 65.56 g, 675.25 mmol, **39**) was added drop-wise at -70 °C. The mixture was stirred at -70 °C for 1 hour. N-methoxy-N-methyl-1-(trifluoromethyl)cyclobutanecarboxamide (62 g, 293.59 mmol, **S94**) in tetrahydrofuran (60 mL) was added drop-wise to the mixture at -70 °C. The resulting solution was stirred at -70 °C for 2 hours. The reaction mixture was quenched with sat. aq. Ammonium chloride (500 mL) at -70 °C. The resulting mixture was extracted with *tert*-butyl methyl ether (2 L). The organic phase was washed with water (400 mL x 2), 1 M Sodium dihydrogen phosphate (aq., 400 mL x 2), brine (500 mL), dried over Sodium sulfate, filtered and concentrated under reduced pressure to give 100 g of crude product. This product was combined with another batch of crude product (20 g). The combined crude product was purified by silica gel chromatography (petroleum ether: ethyl acetate = 30: 1) to give (2-fluoro-3-pyridyl)-[1-(trifluoromethyl)cyclobutyl]methanone (76 g) as a yellow oil.

Method 5, LC-MS (ESI+): t_R = 0.82 min; *m*/*z* calcd for $C_{11}H_{10}F_4NO$ $[M + H]^+$: 248.1; found: 248.0

^{1}H-NMR: (400 MHz, $CDCl_3$): δ [ppm] = 8.40-8.38 (m, 1H), 8.04-8.03 (m, 1H), 7.33-7.30 (m, 1H), 2.84-2.81 (m, 2H), 2.65-2.61 (m, 2H), 2.16-1.94 (m, 1H), 1.93-1.89 (m, 1H).

2-fluoro-3-[2-[1-(trifluoromethyl)cyclobutyl]oxiran-2-yl]pyridine (S96)

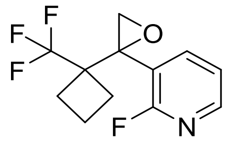

Sodium hydride (20.39 g, 509.73 mmol, 60% purity) was suspended in dimethyl sulfoxide (800 mL) at room temperature under nitrogen. The mixture was stirred at 65 °C for 1 hour. The mixture was then cooled to room temperature, and trimethylsulfoxonium iodide (117.6 g, 534.37 mmol) was added in portions to the mixture, which was stirred at room temperature for 1 hour. (2-fluoro-3-pyridyl)-[1-(trifluoromethyl)cyclobutyl]methanone (60 g, 242.73 mmol, **S95**) was added, and the reaction was allowed to proceed at room temperature for 2 hours. The reaction was quenched with ice-water (1 L). The resulting mixture was extracted with ethyl acetate (1 L x 2). The combined organic phase was washed with water (500 mL), brine (500 mL), dried over sodium sulfate, filtered and concentrated under reduced pressure to give 80 g of crude product. This crude product was combined with another batch of crude product (20 g). The combined crude product was purified by silica gel chromatography (petroleum ether: ethyl acetate = 10: 1) to give 2-fluoro-3-[2-[1-(trifluoromethyl)cyclobutyl]oxiran-2-yl]pyridine (68 g) as a yellow oil.

Method 5, LC-MS (ESI+): t_R = 0.83 min; *m/z* calcd for $C_{12}H_{12}F_4NO$ $[M + H]^+$: 262.1; found: 262.0

^{1}H-NMR: (400 MHz, $CDCl_3$): δ [ppm] = 8.21-8.19 (m, 1H), 7.86-7.85 (m, 1H), 7.20-7.18 (m, 1H), 3.47-3.38 (m, 1H), 2.98-2.97 (m, 1H), 2.52-2.26 (m, 1H), 2.25-1.91 (m, 5H).

3-[1-(trifluoromethyl)cyclobutyl]-1,2-dihydropyrrolo[2,3-b]pyridin-3-ol (S97)

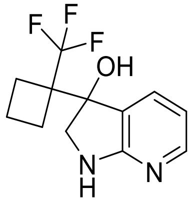

To a solution of 2-fluoro-3-[2-[1-(trifluoromethyl)cyclobutyl]oxiran-2-yl]pyridine (68 g, 260 mmol, **S96**) in tetrahydrofuran (120 mL) was added aqueous ammonia (1.5 L, 28% purity) in one portion at room temperature. The mixture was stirred at 100 °C in autoclave for 72 hours. The reaction mixture was cooled to room temperature and extracted with ethyl acetate (2 L). The organic phase was washed with brine (500 mL), dried over sodium sulfate, filtered and

concentrated under reduced pressure to give 3-[1-(trifluoromethyl)cyclobutyl]-1,2-dihydropyrrolo[2,3-b]pyridin-3-ol (69 g, crude) as a yellow oil.

^{1}H-NMR: (400 MHz, $CDCl_3$): δ [ppm] = 7.89-7.87 (m, 1H), 7.55-7.53 (m, 1H), 6.59-6.54 (m, 1H), 4.62 (brs, 1H), 3.80-3.77 (m, 1H), 3.50-3.47 (m, 1H), 2.61-2.56 (m, 2H), 2.39-2.35 (m, 2H), 2.06-2.04 (m, 2H).

3-[1-(trifluoromethyl)cyclobutyl]-1H-pyrrolo[2,3-b]pyridine (S98)

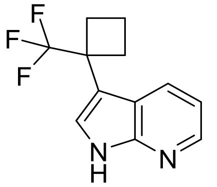

To a solution of 3-[1-(trifluoromethyl)cyclobutyl]-1,2-dihydropyrrolo[2,3-b]pyridin-3-ol (69 g, crude, **S97**) and pyridine (56 mL, 693.81 mmol) in dichloromethane (700 mL) was added thionyl chloride (50 mL, 689.25 mmol) at 0 °C under nitrogen. The resulting solution was stirred at room temperature for 16 hours. Ice-water (200 mL) was added to the solution slowly at 0 °C. To the resulting mixture was added sat. aq. Sodium bicarbonate drop-wise until no bubbles appeared. The two phases were separated, and the organic phase was washed with brine (200 mL), dried over magnesium sulfate, filtered and concentrated under reduced pressure. The residue was slurried with *tert*-butyl methyl ether (150 mL) at room temperature for 10 min. The suspension was filtered to yield 3-[1-(trifluoromethyl)cyclobutyl]-1H-pyrrolo[2,3-b]pyridine (41 g) as a light yellow solid.

^{1}H-NMR: (400 MHz, DMSO-d6): δ [ppm] = 11.77 (brs, 1H), 8.24-8.23 (m, 1H), 7.80 (d, 1H), 7.58 (d, 1H), 7.08-7.04 (m, 1H), 2.71-2.66 (m, 2H), 2.57-2.55 (m, 2H), 2.00-1.98 (m, 2H).

3-(1-(trifluoromethyl)cyclobutyl)-1H-pyrrolo[2,3-b]pyridine 7-oxide (S99)

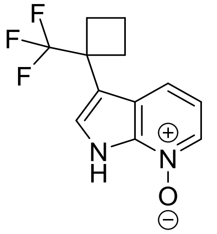

To a solution of 3-[1-(trifluoromethyl)cyclobutyl]-1H-pyrrolo[2,3-b]pyridine (31 g, 129 mmol, **S98**) in dichloromethane (500 mL) was added m-CPBA (40 g, 197.03 mmol, 85% purity) at 0 °C. The mixture was stirred at room temperature for 16 hours. To the reaction solution was

added sat. aq. sodium sulfate (500 mL) at room temperature. The resulting mixture was stirred at room temperature for 30 min. The two phases were separated, and the organic phase was washed with sat. sodium bicarbonate (300 mL x 3), brine (300 mL), dried over magnesium sulfate, filtered and concentrated under reduced pressure to give 3-(1-(trifluoromethyl)cyclobutyl)-1H-pyrrolo[2,3-b]pyridine 7-oxide (48 g, crude) as a yellow solid.

Method 5, LC-MS (ESI+): t_R = 0.72 min; *m/z* calcd for $C_{12}H_{12}F_3N_2O$ $[M + H]^+$: 257.1; found: 257.0

4-nitro-3-(1-(trifluoromethyl)cyclobutyl)-1H-pyrrolo[2,3-b]pyridine 7-oxide (S100)

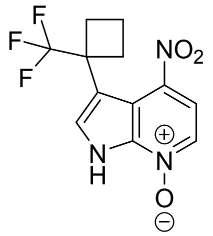

To a mixture of 3-(1-(trifluoromethyl)cyclobutyl)-1H-pyrrolo[2,3-b]pyridine 7-oxide (40 g, crude, **S99**) in trifluoroacetic acid (400 mL) was added nitric acid (22 mL, 317.71 mmol, 65% purity) drop-wise at 0 °C. The mixture was stirred at room temperature for 16 hours. The reaction mixture was poured into ice-water (500 mL). The resulting mixture was extracted with dichloromethane (500 mL x 2). The combined organic phase was washed with brine (300 mL x 2), dried over magnesium sulfate, filtered and concentrated by under reduced pressure. The residue was dissolved with dichloromethane (800 mL). The resulting solution was washed with sat. aq. sodium bicarbonate (500 mL), brine (500 mL), dried over magnesium sulfate, filtered and concentrated under reduced pressure to give 30 g of crude product. This crude product was combined with another batch of crude product (20 g) in dichloromethane (500 mL). The resulting solution was concentrated under reduced pressure. The residue was dried in high vacuum to give 4-nitro-3-(1-(trifluoromethyl)cyclobutyl)-1H-pyrrolo[2,3-b]pyridine 7-oxide (38 g, crude) as a yellow solid.

Method 5, LC-MS (ESI+): t_R = 0.72 min; *m/z* calcd for $C_{12}H_{11}F_3N_3O_3$ $[M + H]^+$: 302.1; found: 302.1

6-chloro-4-nitro-3-[1-(trifluoromethyl)cyclobutyl]-1H-pyrrolo[2,3-b]pyridine (S101)

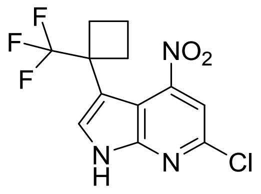

To a solution of 4-nitro-3-(1-(trifluoromethyl)cyclobutyl)-1H-pyrrolo[2,3-b]pyridine 7-oxide (28 g, crude, **S100**) in tetrahydrofuran (300 mL) was added hexamethyldisilazane (22 mL, 104.96 mmol) at 0 °C under nitrogen. Then 2,2,2-trichloroacetyl chloride (22 mL, 197.22 mmol) was added dropwise to the solution at 0 °C. The reaction solution was allowed to warm and stirred at room temperature for 3 hours. The reaction mixture was poured into ice-water (500 mL). The resulting mixture was extracted with ethyl acetate (1 L). The organic phase was washed with sat. aq. Sodium bicarbonate (500 mL x 3), brine (500 mL), dried over sodium sulfate, filtered and concentrated under reduced pressure to give 40 g of crude product. This crude product was combined with another batch of crude product (15 g) in *tert*-butyl methyl ether (400 mL). The resulting solution was concentrated under reduced pressure. The residue was slurried with a mixture of petroleum ether and *tert*-butyl methyl ether (4: 1, 300 mL) to give 6-chloro-4-nitro-3-[1-(trifluoromethyl)cyclobutyl]-1H-pyrrolo[2,3-b]pyridine (37 g, crude) as a yellow solid.

Method 5, LC-MS (ESI+): t_R = 1.07 min; *m*/*z* calcd for $C_{12}H_{10}ClF_3N_3O_2$ $[M + H]^+$: 320.0; found: 320.0

^{1}H-NMR: (400 MHz, $CDCl_3$): δ [ppm] = 10.91 (brs, 1H), 7.50-7.53 (m, 2H), 2.84-2.80 (m, 2H), 2.70-2.68 (m, 2H), 1.97-1.94 (m, 1H), 1.93-1.84 (m, 1H).

6-chloro-4-nitro-3-(1-(trifluoromethyl)cyclobutyl)-1-((2-(trimethylsilyl)ethoxy)methyl)-1H-pyrrolo[2,3-b]pyridine (S102)

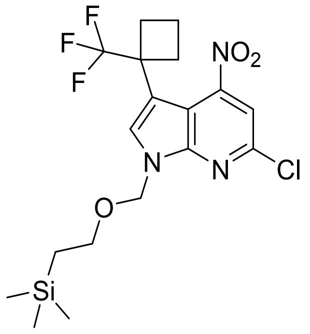

To a solution of 6-chloro-4-nitro-3-[1-(trifluoromethyl)cyclobutyl]-1H-pyrrolo[2,3-b]pyridine (22 g, crude, **S101**) in N,N-dimethylformamide (200 mL) was added N,N-diisopropylethylamine (25.3 mL, 145.25 mmol) at 0 °C. The solution was stirred at 0 °C for 10 min. Then 2-(trimethylsilyl)ethoxymethyl chloride (16.50 mL, 93.23 mmol) was added drop-wise to the solution at 0 °C. The resulting solution was stirred at 0 °C for 20 min. The mixture was diluted with *tert*-butyl methyl ether (500 mL), washed with water (100 mL x 2), brine (100 mL), dried over sodium sulfate, filtered and concentrated under reduced pressure to give 32 g of crude product. The crude product was purified by silica gel chromatography (petroleum ether:etyl acetate = 10: 1) to give 6-chloro-4-nitro-3-(1-(trifluoromethyl)cyclobutyl)-1-((2-(trimethylsilyl)ethoxy)methyl)-1*H*-pyrrolo[2,3-*b*]pyridine (13g, 42% yield) as a yellow oil.

^{1}H-NMR: (400 MHz, $CDCl_3$): δ [ppm] = 7.53-7.52 (m, 2H), 5.69 (s, 2H), 3.61-3.56 (m, 2H), 2.84-2.79 (m, 2H), 2.70-2.67 (m, 2H), 2.29-2.13 (m, 1H), 2.02-1.88 (m, 1H), 0.96-0.92 (m, 2H), -0.03 (s, 9H).

4-((6-chloro-3-(1-(trifluoromethyl)cyclobutyl)-1-((2-(trimethylsilyl)ethoxy)methyl)-1H-pyrrolo[2,3-b]pyridin-4-yl)oxy)-3,5-difluoroaniline (S103)

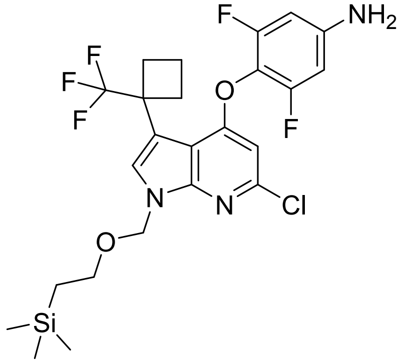

To a solution of 6-chloro-4-nitro-3-(1-(trifluoromethyl)cyclobutyl)-1-((2-(trimethylsilyl)ethoxy)methyl)-1*H*-pyrrolo[2,3-*b*]pyridine (15.4 g, 34.23 mmol, **S102**) and 4-amino-2,6-difluoro-phenol (Intermediate **45**, 10 g, crude, hydrochloric acid salt) in dimethyl sufoxide (150 mL) was added potassium carbonate (19 g, 137.48 mmol) at room temperature under nitrogen. The mixture was stirred at 50 °C for 16 hours. The reaction mixture was cooled to room temperature and filtered through a pad of celite. Ethyl acetate (500 mL) and water (100 mL) were added to the filtrate, and the two phases were separated. The organic phase was washed with water (200 mL x 2), brine (100 mL), dried over sodium sulfate, filtered and concentrated under reduced pressure. The residue was purified by silica gel

chromatography (petroleum ether:ethyl acetate = 4: 1) to give 4-((6-chloro-3-(1-(trifluoromethyl)cyclobutyl)-1-((2-(trimethylsilyl)ethoxy)methyl)-1*H*-pyrrolo[2,3-*b*]pyridin-4-yl)oxy)-3,5-difluoroaniline (16 g, 85% yield) as a yellow solid.

^{1}H-NMR: (400 MHz, $CDCl_3$): δ [ppm] = 7.18 (s, 1H), 6.34-6.32 (m, 3H), 5.61 (s, 2H), 3.92 (brs, 2H), 3.61-3.57 (m, 2H), 2.81-2.76 (m, 2H), 2.68-2.66 (m, 2H), 2.16-2.13 (m, 1H), 2.00-1.95 (m, 1H), 0.94-0.90 (m, 2H), -0.04 (s, 9H).

3,5-difluoro-4-((3-(1-(trifluoromethyl)cyclobutyl)-1-((2-(trimethylsilyl)ethoxy)methyl)-1H-pyrrolo[2,3-b]pyridin-4-yl)oxy)aniline (S104)

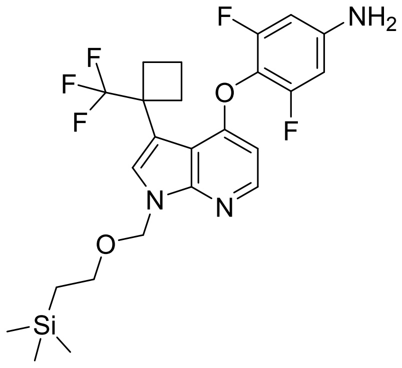

To a solution of 4-((6-chloro-3-(1-(trifluoromethyl) cyclobutyl)-1-((2-(trimethylsilyl)ethoxy)methyl)-1*H*-pyrrolo[2,3-*b*]pyridin-4-yl)oxy)-3,5-difluoroaniline (16 g, 29.20 mmol, **S103**) in tetrahydrofuran (150 mL) was added triethylamine (6.2 mL, 44.54 mmol) and Pd/C (3 g, 10% purity - wet, contains 50% of water) under nitrogen at room temperature. The suspension was put under vacuum and purged with H_2 several times. The mixture was stirred under H_2 (15 psi) at 45 °C for 12 hours. The mixture was filtered through a pad of celite, and the filtrate concentrated under reduced pressure. The residue was re-dissolved in tetrahydrofuran (150 mL) and subjected again to the reaction conditions. This operation had to be repeated once more (a total of 3 times) to achieve reaction completion. The reaction mixture was cooled to room temperature, then filtered through a pad of celite. The filtrate was concentrated under reduced pressure. The residue was purified by silica gel chromatography (petroleum ether:ethyl acetate = 5: 1 to 3: 1) and the resulting residue dissolved in 20 mL of acetonitrile. Upon addition of water (100 mL) a white solid precipitated. The resulting mixture was lyophilized to give 3,5-difluoro-4-((3-(1-(trifluoromethyl)cyclobutyl)-1-((2-(trimethylsilyl)ethoxy)methyl)-1*H*-pyrrolo[2,3-*b*]pyridin-4-yl)oxy)aniline (12.5 g, 83% yield) as a white solid.

Method 6, LC-MS (ESI+): t_R = 2.57 min; *m/z* calcd for $C_{24}H_{29}F_5N_3O_2Si$ $[M + H]^+$: 514.2; found: 514.2

^{1}H-NMR: (400 MHz, $CDCl_3$): δ [ppm] = 8.13 (s, 1H), 7.21 (s, 1H), 6.35-6.30 (m, 3H), 5.67 (s, 2H), 3.87 (brs, 2H), 3.62-3.57 (m, 2H), 2.83-2.80 (m, 2H), 2.78-2.70 (m, 2H), 2.16-2.14 (m, 1H), 1.99-1.97 (m, 1H), 0.94-0.90 (m, 2H), -0.04 (s, 9H).

O-phenyl {3,5-difluoro-4-[(3-[1-(trifluoromethyl)cyclobutyl]-1-{[2-(trimethylsilyl)ethoxy]methyl}-1H-pyrrolo[2,3-b]pyridin-4-yl)oxy]phenyl}carbamothioate (S105)

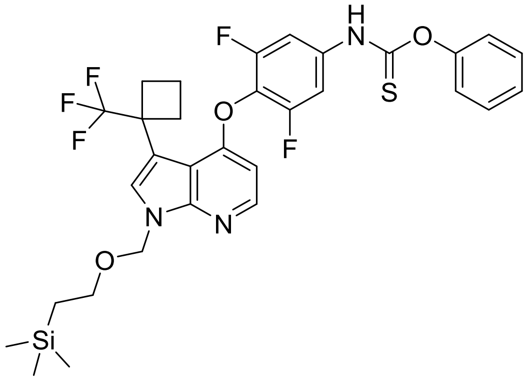

3,5-difluoro-4-[(3-[1-(trifluoromethyl)cyclobutyl]-1-{[2-(trimethylsilyl)ethoxy]methyl}-1H-pyrrolo[2,3-b]pyridin-4-yl)oxy]aniline (400 mg, 779 µmol, **S104**) were dissolved in a mixture of THF/pyridine (6.2 mL:2.8 mL) and cooled down to 0°C. O-phenyl carbonochloridothioate (120 µl, 860 µmol) was added dropwise, and the resulting reaction stirred for 1h at 0°C. The solvent was removed under vacuum and the product used in the next step with no further purification.

Method 2, UPLC-MS (ESI−): t_R = 1.90 min; *m/z* calcd for $C_{31}H_{31}F_5N_3O_3SSi$ $[M - H]^-$: 648.2; found: 648.4

N-{3,5-difluoro-4-[(3-[1-(trifluoromethyl)cyclobutyl]-1-{[2-(trimethylsilyl)ethoxy]methyl}-1H-pyrrolo[2,3-b]pyridin-4-yl)oxy]phenyl}-N'-[(3-methyloxetan-3-yl)methyl]urea (S106)

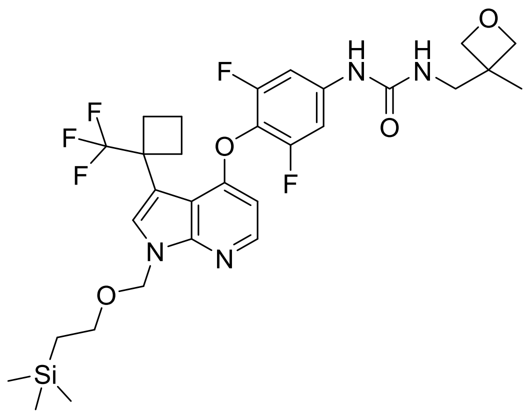

To a stirred solution of 3,5-difluoro-4-[(3-[1-(trifluoromethyl)cyclobutyl]-1-{[2-(trimethylsilyl)ethoxy]methyl}-1H-pyrrolo[2,3-b]pyridin-4-yl)oxy]aniline (80.0 mg, 156 µmol, **S105**) in a mixture of pyridine (0.63 mL) and dichloromethane (0.5 mL) was added 3-(isocyanatomethyl)-3-methyloxetane (99.0 mg, 779 µmol). The resulting mixture was stirred at 60°C for 16 hours at which time water was added and the layers were separated. The aqueous phase was extracted two times with ethyl acetate and the combined organic phases were washed with brine, dried over sodium sulfate, filtered and evaporated under reduced pressure to afford the crude product which was used without further purification.

Method 1, UPLC-MS (ESI+): t_R = 1.56 min; *m/z* calcd for $C_{30}H_{38}F_5N_4O_4Si$ $[M + H]^+$: 641.3; found: 641.7

__Compound 31__:

{(5S)-2-[3,5-difluoro-4-({3-[1-(trifluoromethyl)cyclobutyl]-1H-pyrrolo[2,3-b]pyridin-4-yl}oxy)anilino]-5-methyl-5,6-dihydro-4H-1,3-oxazin-5-yl}methanol

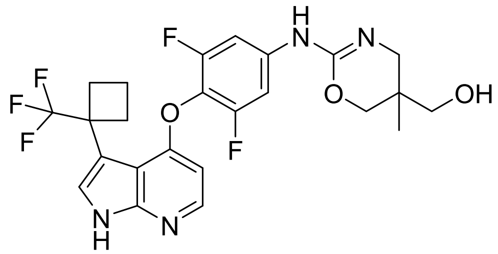

To a stirred solution of N-{3,5-difluoro-4-[(3-[1-(trifluoromethyl)cyclobutyl]-1-{[2-(trimethylsilyl)ethoxy]methyl}-1H-pyrrolo[2,3-b]pyridin-4-yl)oxy]phenyl}-N'-[(3-methyloxetan-3-yl)methyl]urea (50.0 mg, 78.0 µmol, **S106**) in dichloromethane (0.5 mL) was added trifluoro-acetatic acid (90 µl, 1.2 mmol). The resulting mixture was stirred at room temperature for 16 hours at which time the mixture was neutralized with a saturated aqueous solution of sodium bicarbonate and then extracted 2 times with ethyl acetate. The combined organic phases were

washed with brine, dried over sodium sulfate, and evaporated under reduced pressure. The resulting residue was dissolved in acetonitrile, treated with a 25% aqueous solution of ammonia (1 mL) for 1 hour, and evaporated to afford the crude product. The crude mixture was then purified by preparative HPLC to afford the title product (12 mg, 30% yield over two steps).

Method 1, UPLC-MS (ESI+): t_R = 1.13 min; *m/z* calcd for $C_{24}H_{24}F_5N_4O_3Si$ $[M + H]^+$: 511.2; found: 511.7

^1H NMR (400 MHz, DMSO-d_6) δ ppm = 0.90 (s, 3 H), 1.88 – 2.04 (m, 2H), 2.64 – 2.68 (m, 4H), 3.00 (d, *J* = 14.2 Hz, 1H), 3.19 - 3.33 (m, 3 H), 3.87 (d, *J* = 10.4 Hz, 1H), 4.06 (d, *J* = 10.4 Hz, 1H), 4.80 – 4.86 (br m, 1 H), 6.24 (d, *J* = 5.3 Hz, 1 H), 7.30 – 7.55 (br s, 1 H), 7.50 (d, *J* = 2.3 Hz, 1 H), 8.04 (d, *J* = 5.6 Hz, 1 H), 11.93 (br s, 1 H) (aromatic x 2H adjacent to F not visible)

(2-fluoropyridin-3-yl)[1-(trifluoromethyl)cyclopropyl]methanone (41)

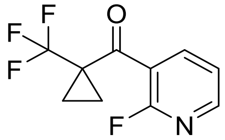

To a solution of 2-fluoropyridine (1.9 g, 19.6 mmol, **39**) in THF (40 mL) was added LDA (1 M, 25 mL, freshly prepared) dropwise at -78 °C under nitrogen. The mixture was stirred at -78 °C for 1 hour. Then N-methoxy-N-methyl-1 (trifluoromethyl)cyclopropanecarboxamide (3.8 g, 19.3 mmol, **40**, prepared as described in Org. Process Res. Dev., 2009, 13 (3), pp 576–580) was added. The mixture was warmed to 15 °C and stirred for 1 hour. TLC (Petroleum ether: Ethyl acetate = 5: 1) indicated the reaction completed. The mixture was quenched by adding a saturated aqueous solution of ammonium chloride (50 mL). The mixture was extracted with ethyl acetate (100 mL x 2). The combined organic phase was washed with brine (100 mL x 2), dried over anhydrous Na_2SO_4, filtered and concentrated by rotary evaporator in vacuum. The residue was purified by chromatography on silica gel (Petroleum ether: Ethyl acetate = 100: 1) to give (2-fluoropyridin-3-yl)(1-(trifluoromethyl)cyclopropyl)methanone (2.4 g, 53% yield) as a yellow oil.

^1H NMR ($CDCl_3$, 400 MHz): δ = 1.65-1.54 (m, 4H), 7.31 (t, 1H), 7.87 (t, 1H), 8.38-8.37 (m, 1H).

2-fluoro-3-{2-[1-(trifluoromethyl)cyclopropyl]oxiran-2-yl}pyridine (42)

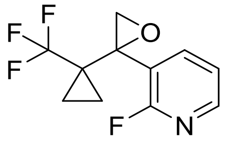

NaH (900 mg, 22.50 mmol, 60% purity) was added into DMSO (40 mL) at 15 °C in one portion. The mixture was heated to 65 °C for 1 hour. Then the mixture was cooled to 15 °C and trimethylsulfoxonium iodide (4.80 g, 21.81 mmol) was added. The mixture was stirred at 15 °C for 1 hour. Then (2-fluoropyridin-3-yl)[1-(trifluoromethyl)cyclopropyl]methanone (2.4 g, 10.3 mmol, **41**) was added. The mixture was stirred at 15 °C for further 13 hours. LC-MS indicated the reaction completed. The reaction mixture was quenched by water (100 mL) slowly. The suspension was extracted with ethyl acetate (100 mL x 2). The combined organic phase was washed with brine (100 mL x 2), dried over anhydrous Na_2SO_4, filtered and concentrated by rotary evaporator in vacuum to give 2-fluoro-3-(2-(1-(trifluoromethyl)cyclopropyl)oxiran-2-yl)pyridine, which was used without further purification.

Method 7, LC-MS (ESI+): t_R = 0.82 min; *m/z* calcd for $C_{11}H_{10}F_4NO$ $[M + H]^+$: 248.1; found: 248.1

3-[1-(trifluoromethyl)cyclopropyl]-2,3-dihydro-1H-pyrrolo[2,3-b]pyridin-3-ol (S107)

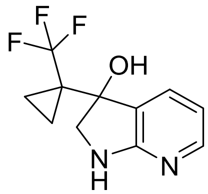

To a solution of 2-fluoro-3-{2-[1-(trifluoromethyl)cyclopropyl]oxiran-2-yl}pyridine (2.4 g, 9.7 mmol, **42**) in THF (12 mL) was added aq.$NH_3 \cdot H_2O$ (50 mL, 364 mmol, 28% purity) at 10 °C. The mixture was stirred at 60 °C for 32 hours. LC-MS indicated the reaction completed. The mixture was poured into water (50 mL). The suspension was extracted with ethyl acetate (50 mL x 3). The combined organic phase was washed with brine (50 mL x 2), dried over anhydrous Na_2SO_4, filtered and concentrated by rotary evaporator in vacuum. The residue was purified by prep-HPLC (column: Phenomenex Gemini C18 250*50mm*10 um; mobile phase: [water (0.05% ammonia hydroxide v/v)-ACN]; B%: 20%-45%, 26 MIN; 78% min) to get a solution, which was concentrated to 100 mL at 30 °C by rotary evaporator in vacuum. The formed solid was collected by filtration and dried in vacuum to give the first batch of 3-(1-(trifluoromethyl)cyclopropyl)-2,3-dihydro-1H-pyrrolo[2,3-b]pyridin-3-ol (1.0 g, 42% yield) as a white solid. The filtrate was lyophilized to give the second batch of 3-(1-(trifluoromethyl)cyclopropyl)-2,3-dihydro-1H-pyrrolo[2,3-b]pyridin-3-ol (200 mg, 8% yield) as a white solid.

Method 8, LC-MS (ESI+): t_R = 0.67 min; *m/z* calcd for $C_{11}H_{12}F_3N_2O$ $[M + H]^+$: 245.1; found: 245.0

^1H NMR (DMSO-d_6, 400 MHz): δ = 0.92-0.88 (m, 2H), 1.09-1.05 (m, 2H), 3.35 (d, 1H), 3.73 (d, 1H), 5.68 (s, 1H), 6.50 (dd, 1H), 6.53 (s, 1H), 7.40 (d, 1H), 7.85 (dd, 1H).

^{19}F NMR (DMSO-d_6, 400 MHz): δ = -62

3-[1-(trifluoromethyl)cyclopropyl]-1H-pyrrolo[2,3-b]pyridine (43)

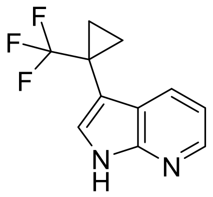

To a mixture of 3-[1-(trifluoromethyl)cyclopropyl]-2,3-dihydro-1H-pyrrolo[2,3-b]pyridin-3-ol (45 g, 158 mmol, 86% purity, **S107**) and pyridine (25 mL, 310 mmol) in dichloromethane (500 mL) was added thionyl chloride (22 mL, 303 mmol) drop-wise at 0 °C under a nitrogen atmosphere. The mixture was stirred at 15°C for 12 hours. The mixture was poured into ice-water (500 mL) and neutralized to pH = 5 ~ 6 with 10% aqueous sodium hydroxide. The aqueous phase was extracted with dichloromethane (300 mL x 2). The combined organic phase was washed with brine (300 mL x 2), dried over sodium sulfate, filtered, and concentrated by rotary evaporator in vacuum. The residue was purified by silica gel chromatography (1000 mesh silica gel, petrol ether : ethyl acetate = 10: 1 to 1: 1) to give the title compound (35 g, 98% yield) as a yellow solid.
^{1}H NMR (400 MHz, DMSO-d_6): δ = 1.17-1.14 (m, 2H), 1.39-1.36 (m, 2H), 7.35-7.32 (m, 1H), 7.74 (d, 1H), 9.30 (d, 1H), 8.39-8.37 (m, 1H), 12.41 (s, 1H).

3-[1-(trifluoromethyl)cyclopropyl]-1H-pyrrolo[2,3-b]pyridine 7-oxide (S108)

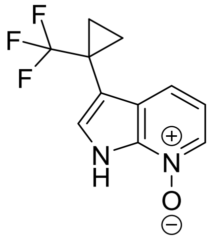

To a solution of 3-[1-(trifluoromethyl)cyclopropyl]-1H-pyrrolo[2,3-b]pyridine (35 g, 155 mmol, intermediate **43**) in dichloromethane (350 mL) was added m-chloroperoxybenzoic acid (47 g, 232 mmol, 85% purity) in portions at 0°C. The mixture was stirred at 15°C for 12 hours. The mixture was filtered, and the filtrate was washed with saturated sodium thiosulfate solution (300 mL x 2), brine (300 mL x 2), dried over sodium sulfate, filtered and concentrated by rotary evaporator in vacuum. The residue was suspended in methyl tert.-butylether (50 mL) and stirred for 30 min. The suspension was filtered, and the cake was washed with methyl tert.-butylether (20 mL x 2)

and dried by in vacuum to give the desired tile compound which was used without further purification.

^{1}H NMR (400 MHz, DMSO-d_6): δ = 1.13 (m, 2H), 1.37-1.34 (m, 2H), 7.15-7.12 (m, 1H), 7.59 (s, 1H), 7.64 (d, 1H), 8.17 (d, 1H), 12.62 (s, 1H).

4-nitro-3-[1-(trifluoromethyl)cyclopropyl]-1H-pyrrolo[2,3-b]pyridine 7-oxide (S109)

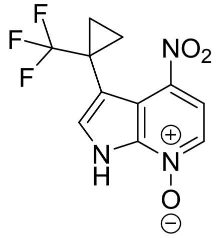

To a solution of 3-[1-(trifluoromethyl)cyclopropyl]-1H-pyrrolo[2,3-b]pyridine 7-oxide (37 g, crude, **S108**) in trifluoroacetic acid (400 mL) was added nitric acid (30 g, 309 mmol, 65% purity) drop-wise at 0°C. The mixture was warmed to 15°C and stirred for 14 hours. Then additional nitric acid (14 g, 222 mmol, 65% purity) was added at 0 °C, the mixture was stirred at 15°C for another 14 hours. The mixture was poured into ice-water (800 mL) and stirred for 10 min. The aqueous phase was extracted with dichloromethane (300 mL x 3). The combined organic phase was washed with brine (300 mL x 2), dried over sodium sulfate, filtered and concentrated by rotary evaporator in vacuum to give the desired title compound which was used without further purification.

^{1}H NMR (400 MHz, DMSO-d_6): δ =1.42-1.37 (m, 4H), 7.95-7.90 (m, 2H), 8.35 (d, 1H), 13.49 (s, 1H).

6-chloro-4-nitro-3-[1-(trifluoromethyl)cyclopropyl]-1H-pyrrolo[2,3-b]pyridine (44)

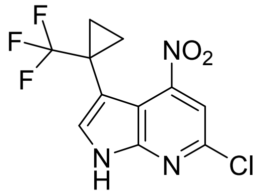

To a solution of 4-nitro-3-[1-(trifluoromethyl)cyclopropyl]-1H-pyrrolo[2,3-b]pyridine 7-oxide (60 g, crude, **S109**) in THF (600 mL) was added hexamethyidisilazane (25 mL, 119 mmol) in one portion at 0°C under nitrogen atmosphere. Then 2,2,2-trichloroacetyl chloride (30 mL, 269 mmol) was added drop-wise. The mixture was warmed to 15°C and stirred for 12 hours. The mixture was poured into ice-water (1 L) and stirred for 30 min. The aqueous phase was extracted with ethyl acetate (500 mL x 2). The combined organic phase was washed with a saturated aqueous solution of sodium bicarbonate (500 mL x 2) and brine (500 mL x 2), dried over sodium sulfate, filtered

and concentrated by rotary evaporator in vacuum. The residue was purified by silica gel chromatography (100-200 mesh silica gel, petrol ether : ethyl acetate = 100: 1 to 10: 1) to give the desired title compound (35 g, 57% purity) as a yellow solid.

Method 9, LC-MS (ESI+): t_R = 1.23 min; *m/z* calcd for $C_{11}H_8ClF_3N_3O_2$ [M + H]$^+$: 306.0; found: 306.0

^{1}H NMR (400 MHz, DMSO-d_6): δ = 1.30 - 1.39 (m, 4H), 7.88 (s, 1H), 8.09 (d, 1H).

6-chloro-4-nitro-3-[1-(trifluoromethyl)cyclopropyl]-1-{[2-(trimethylsilyl)ethoxy]methyl}-1H-pyrrolo[2,3-b]pyridine (S110)

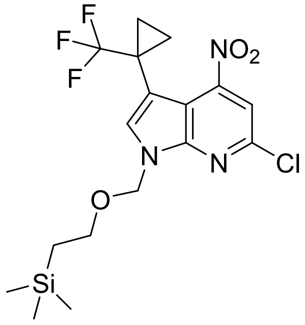

To a solution of 6-chloro-4-nitro-3-[1-(trifluoromethyl)cyclopropyl]-1H-pyrrolo[2,3-b]pyridine (35 g, 65 mmol, 57% purity, intermediate **44**) in DMF (350 mL) was added *N*,*N*-diisopropyl-ethylamine (24 mL, 138 mmol) at 15°C. The mixture was stirred at 15°C for 10 min, then 2-(trimethylsilyl)ethoxymethyl chloride (15 mL, 85 mmol) was added. The mixture was stirred at 15°C for 20 min. The mixture was poured into ice-water (1 L). The aqueous phase was extracted with ethyl acetate (500 mL x 2). The combined organic phase was washed with brine (500 mL x 2), dried over sodium sulfate, filtered and concentrated by rotary evaporator in vacuum. The residue was purified by silica gel chromatography (100-200 mesh silica gel, petrol ether to petrol ether: ethyl acetate = 50: 1) to give the desired title compound (25 g, 46.6% yield, 53% purity) as a yellow oil.

^{1}H NMR (400 MHz, DMSO-d_6): δ = 0.12 (s, 9H), 0.79-0.86 (m, 2H), 1.35-1.29 (m, 2H), 1.43 (m, 2H), 3.55 (d, 2H), 5.64 (s, 2H), 7.98 (s, 1H), 8.31 (s, 1H).

4-[(6-chloro-3-[1-(trifluoromethyl)cyclopropyl]-1-{[2-(trimethylsilyl)ethoxy]methyl}-1H-pyrrolo[2,3-b]pyridin-4-yl)oxy]-3,5-difluoroaniline (S111)

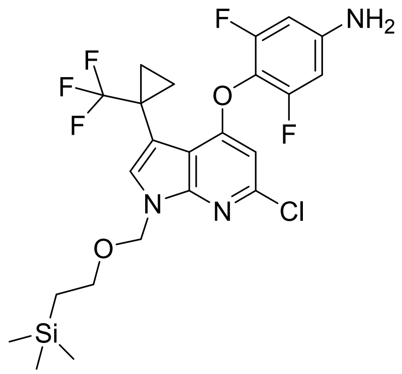

6-chloro-4-nitro-3-[1-(trifluoromethyl)cyclopropyl]-1-{[2-(trimethylsilyl)ethoxy]methyl}-1H-pyrrolo[2,3-b]pyridine (20 g, 24 mmol, 53% purity, **S110**) and 4-amino-2,6-difluoro-phenol (5.29 g, 36.5 mmol, **45**) in DMSO (200 mL) was added potassium carbonate (10.07 g, 72.86 mmol) at 15°C under a nitrogen atmosphere. The mixture was heated to 50°C and stirred for 2 hours. After cooling to room temperature, the reaction mixture was combined with another second identical reaction mixture using 5 g of 6-chloro-4-nitro-3-(1-(trifluoromethyl)cyclopropyl)-1-((2-(trimethylsilyl)ethoxy)methyl)-1*H*-pyrrolo[2,3-*b*]pyridine (intermediate **S110**). The combined reaction mixtures were poured into ice-water (500 mL). The aqueous phase was extracted with ethyl acetate (500 mL x 3). The combined organic phase was washed with brine (500 mL x 2), dried over sodium sulfate, filtered and concentrated by evaporator in vacuum. The residue was purified by silica gel chromatography (100-200 mesh silica gel, petrol ether : ethyl acetate = 30: 1 to 10: 1) to give the desired title compound (9 g, 86% purity) as a yellow solid. Meanwhile, 6-chloro-4-nitro-3-[1-(trifluoromethyl)cyclopropyl]-1-{[2-(trimethylsilyl)ethoxy]methyl}-1H-pyrrolo[2,3-b]pyridine (5 g, 68% purity) was recovered as a yellow oil.

Method 10, LC-MS (ESI+): t_R = 1.00 min; *m/z* calcd for $C_{23}H_{26}ClF_5N_3O_2Si$ $[M + H]^+$: 534.1; found: 534.1

^1H NMR (400 MHz, DMSO-d_6): δ = -0.11 (s, 9H), 0.80 (t, 2H), 1.19-1.16 (m, 2H), 1.37-1.36 (m, 2H), 3.54 (t, 2H), 5.54 (s, 2H), 5.83 (s, 2H), 6.32 (s, 1H), 6.40 (d, 1H), 7.79 (s, 1H).

3,5-difluoro-4-[(3-[1-(trifluoromethyl)cyclopropyl]-1-{[2-(trimethylsilyl)ethoxy]methyl}-1H-pyrrolo[2,3-b]pyridin-4-yl)oxy]aniline (46)

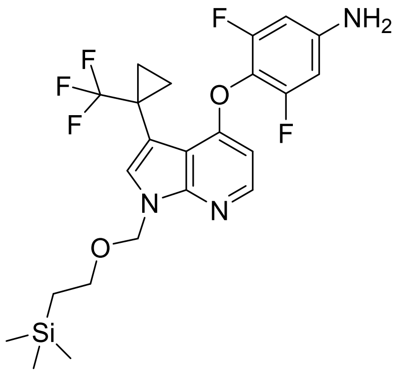

4-[(6-chloro-3-[1-(trifluoromethyl)cyclopropyl]-1-{[2-(trimethylsilyl)ethoxy]methyl}-1H-pyrrolo[2,3-b]pyridin-4-yl)oxy]-3,5-difluoroaniline (9 g, 86 % purity and 3 g, crude, **S111**) in THF (200 mL) were added palladium on charcoal (2 g, 10% purity, containing 50% water) and triethylamine (10 mL, 71.9 mmol) under a nitrogen atmosphere. The suspension was degassed under vacuum and purged with hydrogen several times. The mixture was stirred under hydrogen (15 psi) at 45°C for 36 hours. The mixture was filtered through a pad of *Celite* and the filtrate was concentrated in vacuum. The residue was dissolved in THF (200 mL) and palladium on charcoal (2 g, 10% purity, containing 50% water) was added. The mixture was stirred under hydrogen (15 psi) at 45°C for 60 hours. The mixture was filtered through a pad of *Celite*, and the cake was washed with ethanol (100 mL x 2). The filtrate was concentrated by evaporator in vacuum. The residue was purified by flash silica gel chromatography (0-10% of ethyl acetate in petroleum ether) to give the desired title compound (9 g, containing solvents residue) as brown oil. This product was combined with second batch of product (3 g, containing solvents residue) by dissolving in acetonitrile (200 mL). Water (100 mL) was added. The solution was concentrated by evaporation in vacuum to ~150 mL. The residue was lyophilized to give the desired title compound (10.2 g) as a white solid.

Method 5, LC-MS (ESI+): t_R = 1.03 min; *m*/*z* calcd for $C_{23}H_{27}F_5N_3O_2Si$ $[M + H]^+$: 500.2; found: 500.1

^1H NMR (400 MHz, DMSO-d_6): δ = -0.11 (s, 9H), 0.82-0.78 (m, 2H), 1.17 (m, 2H), 1.38-1.35 (m, 2H), 3.54 (t, 2H), 5.59 (m, 2H), 5.76 (s, 2H), 6.42-6.34 (m, 3H), 7.73 (s, 1H), 8.11 (d, 1H).

^{19}F NMR (400 MHz, DMSO-d_6): δ = -69, -129.

O-phenyl {3,5-difluoro-4-[(3-[1-(trifluoromethyl)cyclopropyl]-1-{[2-(trimethylsilyl)ethoxy]methyl}-1H-pyrrolo[2,3-b]pyridin-4-yl)oxy]phenyl}carbamothioate (47)

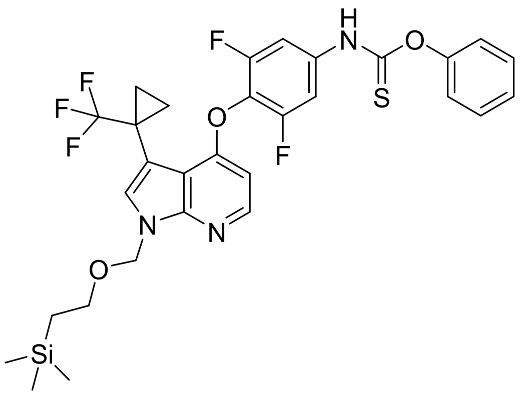

To a stirred solution of 3,5-difluoro-4-[(3-[1-(trifluoromethyl)cyclopropyl]-1-{[2-(trimethylsilyl)ethoxy]methyl}-1H-pyrrolo[2,3-b]pyridin-4-yl)oxy]aniline (500 mg, 1.00 mmol, intermediate **46**) in a mixture of pyridine (750 µl, 9.3 mmol) and THF (7.5 mL) was added O-phenyl carbonochloridothioate (150 µl, 1.1 mmol, CAS No. [1005-56-7]). The reaction mixture was stirred at 0°C for 1h, at which time the solvent was evaporated to afford the crude material which was used in the next step without further purification.

Method 2, UPLC-MS (ESI-): t_R = 1.73 min; *m/z* calcd for $C_{30}H_{29}F_5N_3O_3SSi$ $[M - H]^-$: 634.2; found: 634.5

N-{3,5-difluoro-4-[(3-[1-(trifluoromethyl)cyclopropyl]-1-{[2-(trimethylsilyl)ethoxy]methyl}-1H-pyrrolo[2,3-b]pyridin-4-yl)oxy]phenyl}-N'-{[1-(hydroxymethyl)cyclopropyl]methyl}thiourea (S112)

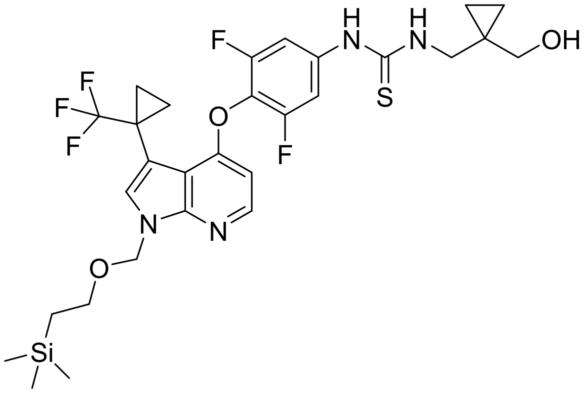

In analogy to **S35**, O-phenyl {3,5-difluoro-4-[(3-[1-(trifluoromethyl)cyclopropyl]-1-{[2-(trimethylsilyl)ethoxy]methyl}-1H-pyrrolo[2,3-b]pyridin-4-yl)oxy]phenyl}carbamothioate (190 mg, 299 µmol, **47**) and [1-(aminomethyl)cyclopropyl]methanol (60.5 mg, 598 µmol, CAS No. [45434-02-4]), in DMF (4.0 mL) were reacted to obtain a crude product which was used in the next step without further purification.

Method 1, UPLC-MS (ESI+): t_R = 1.55 min; *m/z* calcd for $C_{29}H_{36}F_5N_4O_3SSi$ $[M + H]^+$: 643.2; found: 643.4

N-{3,5-difluoro-4-[(3-[1-(trifluoromethyl)cyclopropyl]-1-{[2-(trimethylsilyl)ethoxy]methyl}-1H-pyrrolo[2,3-b]pyridin-4-yl)oxy]phenyl}-5-oxa-7-azaspiro[2.5]oct-6-en-6-amine (S113)

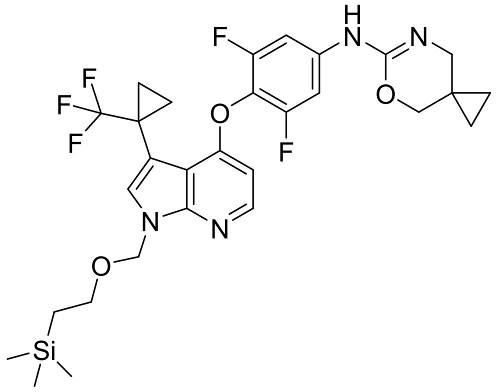

In analogy to **S36**, N-{3,5-difluoro-4-[(3-[1-(trifluoromethyl)cyclopropyl]-1-{[2-(trimethylsilyl)ethoxy]methyl}-1H-pyrrolo[2,3-b]pyridin-4-yl)oxy]phenyl}-N'-{[1-(hydroxymethyl)cyclopropyl]methyl}thiourea (190 mg, 296 µmol, **S112**) was reacted with 1-(3-Dimethylaminopropyl)-3-ethylcarbodiimide hydrochloride (113 mg, 591 µmol) and triethylamine (120 µl, 890 µmol) in acetonitrile (4.0 mL) to obtain a crude product which was used in the next step without further purification.

Method 1, UPLC-MS (ESI-): t_R = 1.63 min; *m/z* calcd for $C_{29}H_{32}F_5N_4O_3Si$ $[M - H]^-$: 607.2; found: 607.6

Compound 32

N-[3,5-difluoro-4-({3-[1-(trifluoromethyl)cyclopropyl]-1H-pyrrolo[2,3-b]pyridin-4-yl}oxy)phenyl]-5-oxa-7-azaspiro[2.5]oct-6-en-6-amine

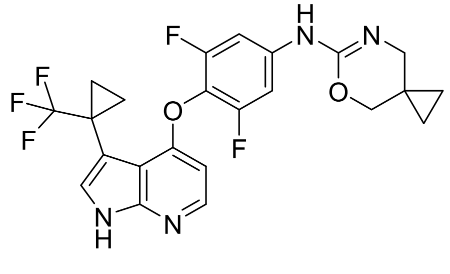

In analogy to compound **2**, N-{3,5-difluoro-4-[(3-[1-(trifluoromethyl)cyclopropyl]-1-{[2-(trimethylsilyl)ethoxy]methyl}-1H-pyrrolo[2,3-b]pyridin-4-yl)oxy]phenyl}-5-oxa-7-azaspiro[2.5]oct-

6-en-6-amine (175 mg, 288 µmol, **S113**) was treated with trifluoroacetic acid (1.0 mL) in dichloromethane (2.0 mL) to afford after preparative HPLC purification the title compound (28 mg, 19% yield over 3 steps).

Method 1, UPLC-MS (ESI+): t_R = 1.25 min; *m/z* calcd for $C_{23}H_{20}F_5N_4O_2$ $[M + H]^+$: 479.2; found: 479.7

^1H NMR (400 MHz, DMSO-d_6) δ ppm = 0.59 (br d, 4 H), 1.18 (br s, 2 H), 1.31 - 1.37 (m, 2 H), 3.21 (br s, 2 H), 4.02 (s, 2 H), 6.25 (d, 1 H), 7.53 (s, 1 H), 7.57 (br s, 1 H), 8.04 (d, 1 H), 8.99 (br s, 1 H), 11.91 (br s, 1 H)

N-{3,5-difluoro-4-[(3-[1-(trifluoromethyl)cyclopropyl]-1-{[2-(trimethylsilyl)ethoxy]methyl}-1H-pyrrolo[2,3-b]pyridin-4-yl)oxy]phenyl}-N'-[2-(1-hydroxycyclopropyl)ethyl]thiourea (S114)

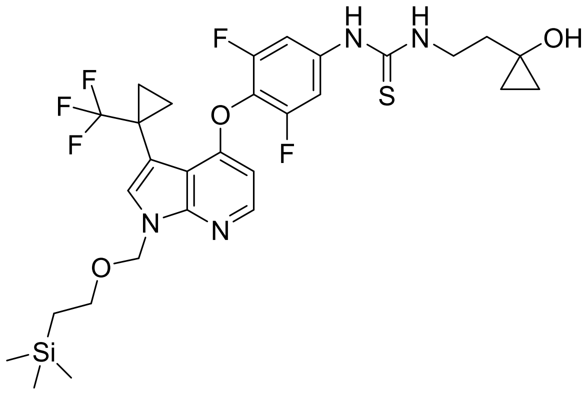

In analogy to **S35**, O-phenyl {3,5-difluoro-4-[(3-[1-(trifluoromethyl)cyclopropyl]-1-{[2-(trimethylsilyl)ethoxy]methyl}-1H-pyrrolo[2,3-b]pyridin-4-yl)oxy]phenyl}carbamothioate (310 mg, 488 µmol, **47**) and formic acid / 1-(2-aminoethyl)cyclopropan-1-ol (1/1) (144 mg, 975 µmol), in DMF (5.0 mL) were reacted to obtain a crude product which was used in the next step without further purification.

Method 1, UPLC-MS (ESI+): t_R = 1.55 min; *m/z* calcd for $C_{29}H_{36}F_5N_4O_3SSi$ $[M + H]^+$: 643.2; found: 643.8

N-{3,5-difluoro-4-[(3-[1-(trifluoromethyl)cyclopropyl]-1-{[2-(trimethylsilyl)ethoxy]methyl}-1H-pyrrolo[2,3-b]pyridin-4-yl)oxy]phenyl}-4-oxa-6-azaspiro[2.5]oct-5-en-5-amine (S115)

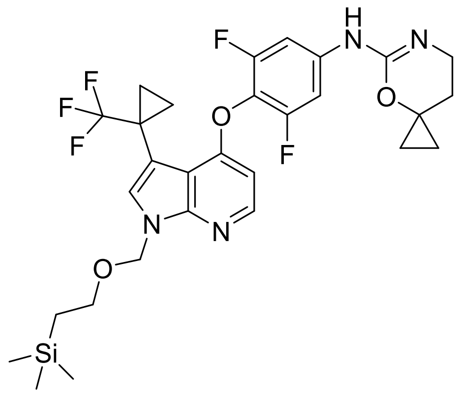

In analogy to **S36**, N-{3,5-difluoro-4-[(3-[1-(trifluoromethyl)cyclopropyl]-1-{[2-(trimethylsilyl)ethoxy]methyl}-1H-pyrrolo[2,3-b]pyridin-4-yl)oxy]phenyl}-N'-[2-(1-hydroxycyclopropyl)ethyl]thiourea (310 mg, 482 µmol, **S114**) was reacted with 1-(3-Dimethylaminopropyl)-3-ethylcarbodiimide hydrochloride (185 mg, 965 µmol) and triethylamine (200 µl, 1.4 mmol) in acetonitrile (5.0 mL) to obtain a crude product which was used in the next step without further purification.

Method 1, UPLC-MS (ESI-): t_R = 1.65 min; *m*/*z* calcd for $C_{29}H_{32}F_5N_4O_3Si$ $[M - H]^-$: 607.2; found: 607.5

Compound 33

N-[3,5-difluoro-4-({3-[1-(trifluoromethyl)cyclopropyl]-1H-pyrrolo[2,3-b]pyridin-4-yl}oxy)phenyl]-4-oxa-6-azaspiro[2.5]oct-5-en-5-amine

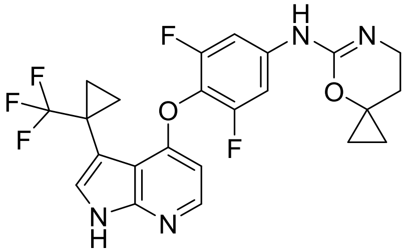

In analogy to **compound 2**, N-{3,5-difluoro-4-[(3-[1-(trifluoromethyl)cyclopropyl]-1-{[2-(trimethylsilyl)ethoxy]methyl}-1H-pyrrolo[2,3-b]pyridin-4-yl)oxy]phenyl}-4-oxa-6-azaspiro[2.5]oct-5-en-5-amine (290 mg, 476 µmol, **S115**) was treated with trifluoroacetic acid (0.5 mL, 6.5 mmol) in dichloromethane (3.0 mL) to afford after preparative HPLC purification the title compound (50 mg, 22% yield over 3 steps).

Method 1, UPLC-MS (ESI+): t_R = 1.25 min; *m*/*z* calcd for $C_{23}H_{20}F_5N_4O_2$ $[M + H]^+$: 479.1; found: 479.6

^{1}H NMR (400 MHz, DMSO-d_6): δ = 0.68 – 0.71 (m, 2H), 0.92 – 0.94 (m, 2H), 1.17 - 1.20 (m, 2H), 1.32 - 1.35 (m, 2H), 1.80 - 1.91 (br s, 2H), 3.42 - 3.56 (br s, 2H), 6.24 (d, *J* = 5.6 Hz, 1H), 7.53 (s, 1H), 8.03 (d, *J* = 5.6 Hz, 1H), 8.97 – 9.03 (br s, 1H), 11.91 (br s, 1H) (Aromatic 2 x C-H adjacent to F not visible)

N-{3,5-difluoro-4-[(3-[1-(trifluoromethyl)cyclopropyl]-1-{[2-(trimethylsilyl)ethoxy]methyl}-1H-pyrrolo[2,3-b]pyridin-4-yl)oxy]phenyl}-N'-(3-hydroxy-2,2-dimethylpropyl)thiourea (S116)

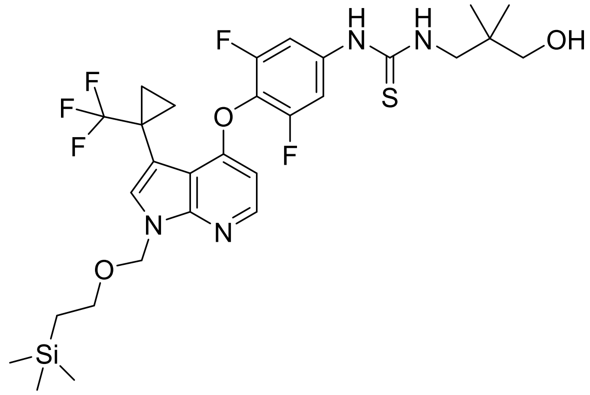

In analogy to **S35**, O-phenyl {3,5-difluoro-4-[(3-[1-(trifluoromethyl)cyclopropyl]-1-{[2-(trimethylsilyl)ethoxy]methyl}-1H-pyrrolo[2,3-b]pyridin-4-yl)oxy]phenyl}carbamothioate (300 mg, 472 µmol, **47**) and 3-amino-2,2-dimethylpropan-1-ol (97.4 mg, 944 µmol, CAS No. [141-43-5]), in DMF (12 mL) were reacted to obtain a crude product which was used in the next step without further purification.

Method 1, UPLC-MS (ESI+): t_R = 1.56 min; *m*/*z* calcd for $C_{29}H_{38}F_5N_4O_3SSi$ $[M + H]^+$: 645.2; found: 645.8

N-{3,5-difluoro-4-[(3-[1-(trifluoromethyl)cyclopropyl]-1-{[2-(trimethylsilyl)ethoxy]methyl}-1H-pyrrolo[2,3-b]pyridin-4-yl)oxy]phenyl}-5,5-dimethyl-5,6-dihydro-4H-1,3-oxazin-2-amine (S117)

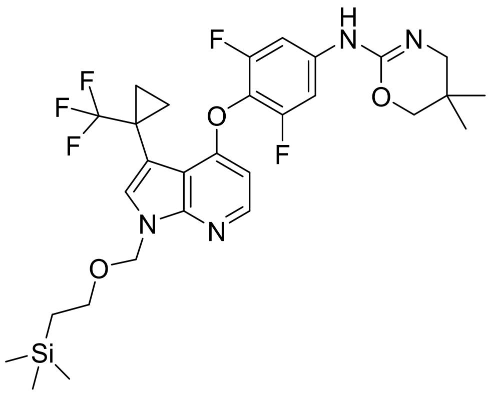

In analogy to **S36**, N-{3,5-difluoro-4-[(3-[1-(trifluoromethyl)cyclopropyl]-1-{[2-(trimethylsilyl)ethoxy]methyl}-1H-pyrrolo[2,3-b]pyridin-4-yl)oxy]phenyl}-N'-(3-hydroxy-2,2-dimethylpropyl)thiourea (300 mg, 465 µmol, **S116**) was reacted with 1-(3-Dimethylaminopropyl)-3-ethylcarbodiimide hydrochloride (178 mg, 931 µmol) and triethylamine (190 µl, 1.4 mmol) in acetonitrile (5.0 mL) to obtain a crude product which was used in the next step without further purification.

Method 1, UPLC-MS (ESI+): t_R = 1.67 min; *m/z* calcd for $C_{29}H_{36}F_5N_4O_3Si$ $[M + H]^+$: 611.2; found: 611.4

Compound 34

N-[3,5-difluoro-4-({3-[1-(trifluoromethyl)cyclopropyl]-1H-pyrrolo[2,3-b]pyridin-4-yl}oxy)phenyl]-5,5-dimethyl-5,6-dihydro-4H-1,3-oxazin-2-amine

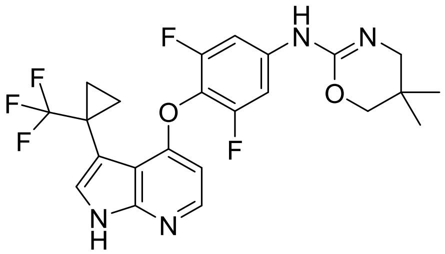

In analogy to **compound 2**, N-{3,5-difluoro-4-[(3-[1-(trifluoromethyl)cyclopropyl]-1-{[2-(trimethylsilyl)ethoxy]methyl}-1H-pyrrolo[2,3-b]pyridin-4-yl)oxy]phenyl}-5,5-dimethyl-5,6-dihydro-4H-1,3-oxazin-2-amine (280 mg, 458 µmol, **S117**) was treated with trifluoroacetic acid (2.0 mL) in dichloromethane (2.0 mL) to afford after preparative HPLC purification the title compound (82 mg, 37% yield over 3 steps).

Method 1, UPLC-MS (ESI+): t_R = 1.28 min; *m/z* calcd for $C_{23}H_{22}F_5N_4O_2$ $[M + H]^+$: 481.2; found: 481.8

^{1}H NMR (400 MHz, DMSO-d_6) δ ppm 0.96 (br s, 6 H), 1.18 (br s, 2 H), 1.30 - 1.36 (m, 2 H), 3.10 (br s, 2 H), 3.87 (br s, 2 H), 6.27 (d, 1 H), 7.53 (d, 1 H), 7.58 (br s, 1 H), 8.04 (d, 1 H), 9.01 (br s, 1 H), 11.91 (br d, 1 H)

N-{3,5-difluoro-4-[(3-[1-(trifluoromethyl)cyclopropyl]-1-{[2-(trimethylsilyl)ethoxy]methyl}-1H-pyrrolo[2,3-b]pyridin-4-yl)oxy]phenyl}-N'-{[1-(hydroxymethyl)cyclobutyl]methyl}thiourea (S118)

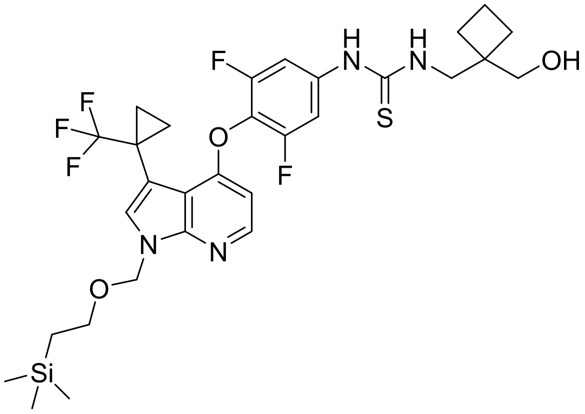

In analogy to **S35**, O-phenyl {3,5-difluoro-4-[(3-[1-(trifluoromethyl)cyclopropyl]-1-{[2-(trimethylsilyl)ethoxy]methyl}-1H-pyrrolo[2,3-b]pyridin-4-yl)oxy]phenyl}carbamothioate (300 mg, 472 µmol, **47**) and [1-(aminomethyl)cyclobutyl]methanol (109 mg, 944 µmol, CAS No. [2041-56-7]), in DMF (12 mL) were reacted to obtain a crude product which was used in the next step without further purification.

Method 1, UPLC-MS (ESI+): t_R = 1.57 min; *m/z* calcd for $C_{30}H_{38}F_5N_4O_3SSi$ $[M + H]^+$: 657.2; found: 657.6

N-{3,5-difluoro-4-[(3-[1-(trifluoromethyl)cyclopropyl]-1-{[2-(trimethylsilyl)ethoxy]methyl}-1H-pyrrolo[2,3-b]pyridin-4-yl)oxy]phenyl}-6-oxa-8-azaspiro[3.5]non-7-en-7-amine (S119)

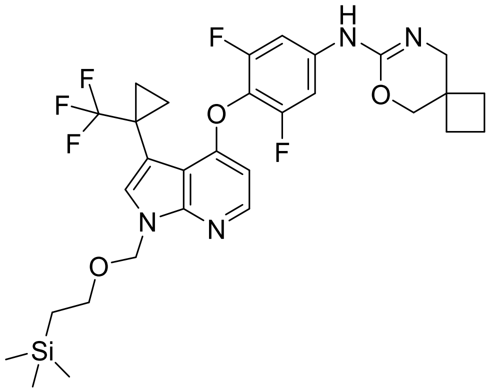

In analogy to **S36**, N-{3,5-difluoro-4-[(3-[1-(trifluoromethyl)cyclopropyl]-1-{[2-(trimethylsilyl)ethoxy]methyl}-1H-pyrrolo[2,3-b]pyridin-4-yl)oxy]phenyl}-N'-{[1-(hydroxymethyl)cyclobutyl]methyl}thiourea (300 mg, 457 µmol, **S118**) was reacted with 1-(3-Dimethylaminopropyl)-3-ethylcarbodiimide hydrochloride (175 mg, 914 µmol) and triethylamine (190 µl, 1.4 mmol) in acetonitrile (5.0 mL) to obtain a crude product which was used in the next step without further purification.

Method 1, UPLC-MS (ESI+): t_R = 1.68 min; *m/z* calcd for $C_{30}H_{36}F_5N_4O_3Si$ $[M + H]^+$: 623.2; found: 623.4

<u>Compound 35</u>

N-[3,5-difluoro-4-({3-[1-(trifluoromethyl)cyclopropyl]-1H-pyrrolo[2,3-b]pyridin-4-yl}oxy)phenyl]-6-oxa-8-azaspiro[3.5]non-7-en-7-amine

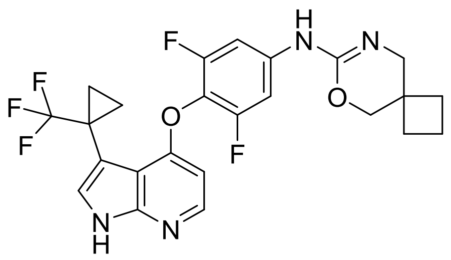

In analogy to **compound 2**, N-{3,5-difluoro-4-[(3-[1-(trifluoromethyl)cyclopropyl]-1-{[2-(trimethylsilyl)ethoxy]methyl}-1H-pyrrolo[2,3-b]pyridin-4-yl)oxy]phenyl}-6-oxa-8-azaspiro[3.5]non-7-en-7-amine (280 mg, 450 µmol, **S119**) was treated with trifluoroacetic acid (2.0 mL) in dichloromethane (2.0 mL) to afford after preparative HPLC purification the title compound (68 mg, 30% yield over 3 steps).

Method 1, UPLC-MS (ESI+): t_R = 1.29 min; *m/z* calcd for $C_{24}H_{22}F_5N_4O_2$ $[M + H]^+$: 493.2; found: 493.8

^{1}H NMR (400 MHz, DMSO-d_6) δ ppm 1.18 (br s, 2 H), 1.31 - 1.36 (m, 2 H), 1.78 - 2.02 (m, 6 H), 3.31 (br s, 2 H), 4.14 (s, 2 H), 6.26 (d, 1 H), 7.53 (d, 1 H), 8.04 (d, 1 H), 9.01 (br s, 1 H), 11.91 (d, 1 H)

N-{3,5-difluoro-4-[(3-[1-(trifluoromethyl)cyclopropyl]-1-{[2-(trimethylsilyl)ethoxy]methyl}-1H-pyrrolo[2,3-b]pyridin-4-yl)oxy]phenyl}-N'-[(3S,4S)-4-(hydroxymethyl)oxolan-3-yl]thiourea (S120)

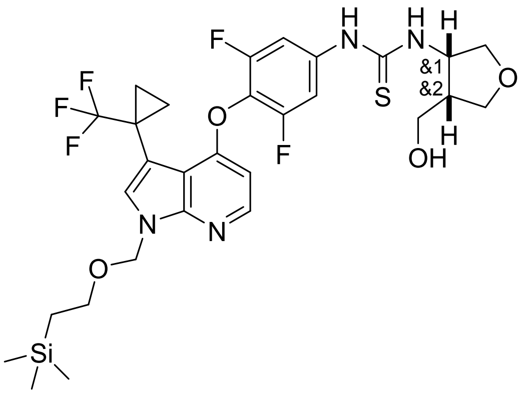

In analogy to **S35**, O-phenyl {3,5-difluoro-4-[(3-[1-(trifluoromethyl)cyclopropyl]-1-{[2-(trimethylsilyl)ethoxy]methyl}-1H-pyrrolo[2,3-b]pyridin-4-yl)oxy]phenyl}carbamothioate (300 mg, 472 µmol, **47**) and [(*rac*)-4-aminooxolan-3-yl]methanol - hydrogen chloride (1/1) (145 mg, 944 µmol), in DMF (12 mL) were reacted to obtain a crude product which was used in the next step without further purification.

Method 1, UPLC-MS (ESI+): t_R = 1.47 min; *m/z* calcd for $C_{29}H_{36}F_5N_4O_4SSi$ $[M + H]^+$: 659.2; found: 659.6

(*rac-cis*)-N-{3,5-difluoro-4-[(3-[1-(trifluoromethyl)cyclopropyl]-1-{[2-(trimethylsilyl)ethoxy]methyl}-1H-pyrrolo[2,3-b]pyridin-4-yl)oxy]phenyl}-4a,5,7,7a-tetrahydro-4H-furo[3,4-d][1,3]oxazin-2-amine (S121)

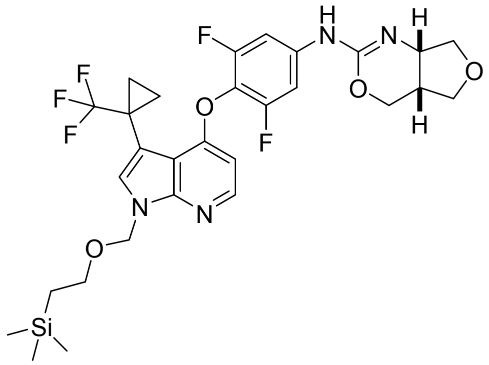

In analogy to **S36**, N-{3,5-difluoro-4-[(3-[1-(trifluoromethyl)cyclopropyl]-1-{[2-(trimethylsilyl)ethoxy]methyl}-1H-pyrrolo[2,3-b]pyridin-4-yl)oxy]phenyl}-N'-[(*rac*)-4-(hydroxymethyl)oxolan-3-yl]thiourea (300 mg, 455 µmol, **S120**) was reacted with 1-(3-Dimethylaminopropyl)-3-ethylcarbodiimide hydrochloride (175 mg, 911 µmol) and triethylamine (190 µl, 1.4 mmol) in acetonitrile (5.0 mL) to obtain a crude product which was used in the next step without further purification.

Method 1, UPLC-MS (ESI+): t_R = 1.54 min; *m/z* calcd for $C_{29}H_{34}F_5N_4O_4Si$ $[M + H]^+$: 625.2; found: 625.4

Compound 36

(4aR,7aS)-N-[3,5-difluoro-4-({3-[1-(trifluoromethyl)cyclopropyl]-1H-pyrrolo[2,3-b]pyridin-4-yl}oxy)phenyl]-4a,5,7,7a-tetrahydro-4H-furo[3,4-d][1,3]oxazin-2-amine

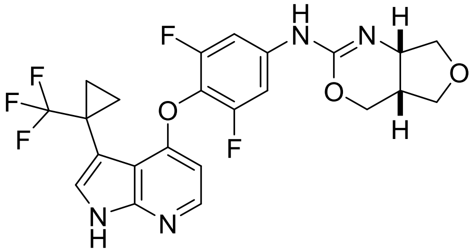

In analogy to compound **2**, (*rac*)-N-{3,5-difluoro-4-[(3-[1-(trifluoromethyl)cyclopropyl]-1-{[2-(trimethylsilyl)ethoxy]methyl}-1H-pyrrolo[2,3-b]pyridin-4-yl)oxy]phenyl}-4a,5,7,7a-tetrahydro-4H-furo[3,4-d][1,3]oxazin-2-amine (280 mg, 448 µmol, **S121**) was treated with trifluoroacetic acid (2.0 mL) in dichloromethane (2.0 mL) to afford after preparative HPLC purification the title compound (76 mg, 33% yield over 3 steps).

Method 1, UPLC-MS (ESI+): t_R = 1.12 min; *m/z* calcd for $C_{23}H_{20}F_5N_4O_3$ $[M + H]^+$: 495.1; found: 495.7

^{1}H NMR (400 MHz, DMSO-*d*$_6$) δ ppm = 1.16- 1.19 (br s, 2 H), 1.32 - 1.35 (m, 2 H), 2.57 – 2.65 (br. s, 1H), (br s, 2 H), 3.50 – 3.70 (br m, 2H), 3.81 – 3.91 (br s, 2H), 3.95 – 4.08 (br m, 2 H), 4.29 (dd, *J* = 10.9, 4.1 Hz, 1H), 6.26 (d, *J* = 5.6 Hz, 1 H), 7.53 (d, *J* = 2.3 Hz, 1 H), 7.55 – 7-60 (br m, 1 H), 8.04 (d, *J* = 5.6 Hz, 1 H), 9.11 (br s, 1 H), 11.91 (br d, 1 H)

(+/-)-N-{3,5-difluoro-4-[(3-[1-(trifluoromethyl)cyclopropyl]-1-{[2-(trimethylsilyl)ethoxy]methyl}-1H-pyrrolo[2,3-b]pyridin-4-yl)oxy]phenyl}-N'-{[3-(hydroxymethyl)oxolan-3-yl]methyl}thiourea (S122)

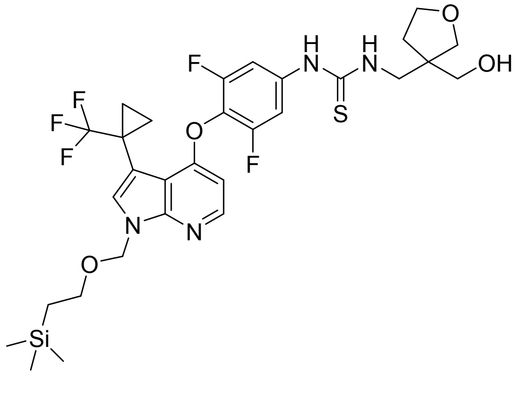

To a stirred solution of O-phenyl {3,5-difluoro-4-[(3-[1-(trifluoromethyl)cyclopropyl]-1-{[2-(trimethylsilyl)ethoxy]methyl}-1H-pyrrolo[2,3-b]pyridin-4-yl)oxy]phenyl}carbamothioate (300 mg, 472 µmol, **47**) in DMF (12.0 mL) was added 3-(aminomethyl)oxolan-3-yl]methanol (124 mg, 944 µmol, CAS No. [1506738-56-2]). The resulting mixture was heated to 60°C for 2h at which time water and ethyl acetate were added and the layers were separated. The aqueous phase was extracted twice with ethyl acetate and the combined organic layers were washed with brine, dried over sodium sulfate, filtered and evaporated to give the crude product which was used without further purification.

Method 1, UPLC-MS (ESI+): t_R = 1.51 min; *m*/*z* calcd for $C_{30}H_{38}F_5N_4O_4SSi$ $[M + H]^+$: 673.2; found: 673.5

(+/-)-N-{3,5-difluoro-4-[(3-[1-(trifluoromethyl)cyclopropyl]-1-{[2-(trimethylsilyl)ethoxy]methyl}-1H-pyrrolo[2,3-b]pyridin-4-yl)oxy]phenyl}-2,7-dioxa-9-azaspiro[4.5]dec-8-en-8-amine (S123)

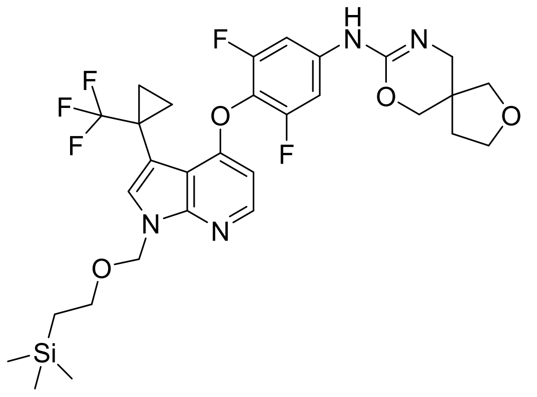

To a solution of (+/-)-*N*-{3,5-difluoro-4-[(3-[1-(trifluoromethyl)cyclopropyl]-1-{[2-(trimethylsilyl)ethoxy]methyl}-1H-pyrrolo[2,3-b]pyridin-4-yl)oxy]phenyl}-N'-{[3-(hydroxymethyl)oxolan-3-yl]methyl}thiourea (310 mg, 461 µmol) in acetonitrile (5.0 mL) was added 1-(3-Dimethylaminopropyl)-3-ethylcarbodiimide hydrochloride (177 mg, 922 µmol, **S122**) and triethylamine (190 µl, 1.4 mmol). The resulting mixture was stirred at 40°C overnight at which time water and ethyl acetate were added and the layers separated. The aqueous phase was extracted twice with ethyl acetate and the combined organic layers were washed with brine, dried over sodium sulfate, filtered and evaporated to afford the crude product which was used without further purification.

Method 1, UPLC-MS (ESI+): t_R = 1.57 min; *m*/*z* calcd for $C_{30}H_{36}F_5N_4O_4Si$ $[M + H]^+$: 639.2; found: 639.4

(+/-)-N-[3,5-difluoro-4-({3-[1-(trifluoromethyl)cyclopropyl]-1H-pyrrolo[2,3-b]pyridin-4-yl}oxy)phenyl]-2,7-dioxa-9-azaspiro[4.5]dec-8-en-8-amine (S124)

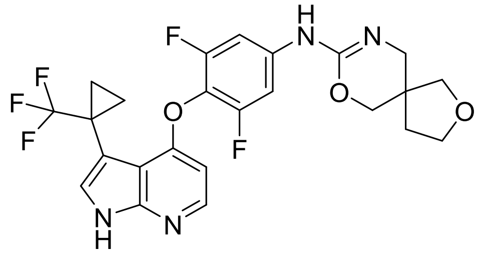

To a solution of (+/-)-N-{3,5-difluoro-4-[(3-[1-(trifluoromethyl)cyclopropyl]-1-{[2-(trimethylsilyl)ethoxy]methyl}-1H-pyrrolo[2,3-b]pyridin-4-yl)oxy]phenyl}-2,7-dioxa-9-azaspiro[4.5]dec-8-en-8-amine (600 mg, 0.94 mmol, **S123**) in dichloromethane (4.0 mL) was added trifluoroacetic acid (4.0 mL). The reaction mixture was stirred at room temperature overnight, at which time the mixture was treated with a 2M solution of sodium hydroxide until pH >10 and ethyl acetate was added. The layers were separated, and the aqueous layer was extracted three times with ethyl acetate. The combined organic layers were washed with brine, dried over sodium sulfate, filtered, and evaporated. The crude material was dissolved in acetonitrile (10 mL) and treated with a 25% aqueous solution of ammonia (5 mL). The resulting solution was stirred for 1 hour and then purified by preparative HPLC to afford the title compound (284 mg, 59% yield over 3 steps).

Method 1, UPLC-MS (ESI+): t_R = 1.15 min; *m/z* calcd for $C_{24}H_{22}F_5N_4O_3$ $[M + H]^+$: 509.2; found: 509.7

^{1}H NMR (400 MHz, DMSO-d_6) δ ppm 1.18 (br s, 2 H), 1.30 - 1.37 (m, 2 H), 1.65 - 1.82 (m, 2 H), 3.41 - 3.50 (m, 1 H), 3.60 (d, 1 H), 3.74 - 3.85 (m, 2 H), 4.10 (q, 2 H), 6.26 (d, 1 H), 7.53 (d, 1 H), 7.57 (br s, 1 H), 8.04 (d, 1 H), 9.07 (br s, 1 H), 11.91 (br d, 1 H)

__Compound 37__

N-[3,5-difluoro-4-({3-[1-(trifluoromethyl)cyclopropyl]-1H-pyrrolo[2,3-b]pyridin-4-yl}oxy)phenyl]-2,7-dioxa-9-azaspiro[4.5]dec-8-en-8-amine (single enantiomer 2)

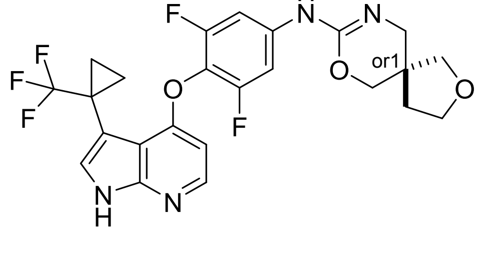

The racemic material **S124** was separated into its enantiomers by preparative chiral SFC to give stereoisomer 1 (76 mg), stereoisomer 2 (58 mg, **Compound 37**). For the isolation of stereoisomer 1, and stereoisomer 2 the following method was used:

Analytical chiral SFC method:

Instrument: Agilent: 1260, Aurora SFC-Module; Column: Chiralpak IC 5µ 100x4.6mm; Eluent A: CO_2; Eluent B: Ethanol + 0.1 Vol-% NH_4OH (32%); Isocratic: 20%B; Flowrate: 4 ml/min; Temperatur: 37.5°C; Pressure: 100bar; UV: 254 nm

Preparative chiral SFC method:

Instrument: Sepiatec: Prep SFC100; Columnn: Chiralpak IC 5µ 250x30mm; Eluent A: CO_2; Eluent B: Ethanol + 0.2 Vol-% NH_4OH (32%); Isocractic: 20%B; Flowrate: 100 ml/min; Temperatur: 40°C; Pressure: 150bar; UV: 254 nm

Analytical chiral HPLC: Rt = 2.62 min, ee = 96.2%

Method 1, UPLC-MS (ESI+): t_R = 1.15 min; *m*/*z* calcd for $C_{24}H_{22}F_5N_4O_3$ $[M + H]^+$: 509.2; found: 509.7

^1H NMR (400 MHz, DMSO-d_6) δ ppm 1.18 (br s, 2 H), 1.30 - 1.37 (m, 2 H), 1.65 - 1.82 (m, 2 H), 3.41 - 3.50 (m, 1 H), 3.60 (d, 1 H), 3.74 - 3.85 (m, 2 H), 4.10 (q, 2 H), 6.26 (d, 1 H), 7.53 (d, 1 H), 7.57 (br s, 1 H), 8.04 (d, 1 H), 9.07 (br s, 1 H), 11.91 (br d, 1 H)

Intermediate 49

N-{3,5-difluoro-4-[(3-[1-(trifluoromethyl)cyclopropyl]-1-{[2-(trimethylsilyl)ethoxy]methyl}-1H-pyrrolo[2,3-b]pyridin-4-yl)oxy]phenyl}-N'-{[4-(hydroxymethyl)oxan-4-yl]methyl}thiourea

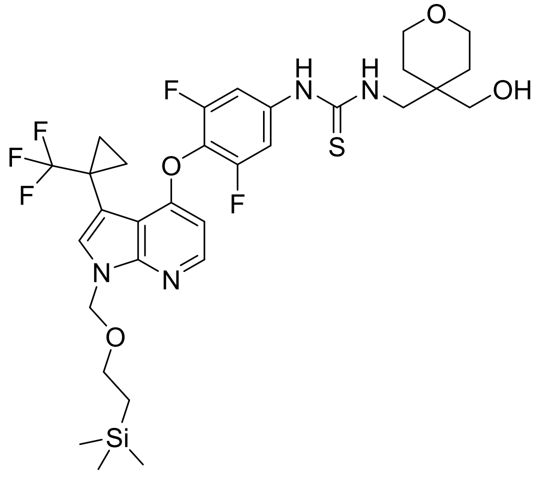

To a stirred solution of O-phenyl {3,5-difluoro-4-[(3-[1-(trifluoromethyl)cyclopropyl]-1-{[2-(trimethylsilyl)ethoxy]methyl}-1H-pyrrolo[2,3-b]pyridin-4-yl)oxy]phenyl}carbamothioate (190 mg, 0.30 mmol, intermediate **47**) in DMF (4.0 mL) was added [4-(aminomethyl)oxan-4-yl]methanol (87 mg, 0.60 mmol, CAS No. [959238-22-3]). The resulting mixture was heated to 60°C for 2 hours at which time water and ethyl acetate were added and the layers were separated. The aqueous phase was extracted twice with ethyl acetate and the combined organic layers were washed with brine, dried over sodium sulfate, filtered, and evaporated to give the crude product which was used without further purification.

Method 1, UPLC-MS (ESI+): t_R = 1.50 min; *m*/*z* calcd for $C_{31}H_{39}F_5N_4O_4SSi$ $[M + H]^+$: 687.2; found: 688

<u>Intermediate 50</u>

N-{3,5-difluoro-4-[(3-[1-(trifluoromethyl)cyclopropyl]-1-{[2-(trimethylsilyl)ethoxy]methyl}-1H-pyrrolo[2,3-b]pyridin-4-yl)oxy]phenyl}-2,9-dioxa-4-azaspiro[5.5]undec-3-en-3-amine

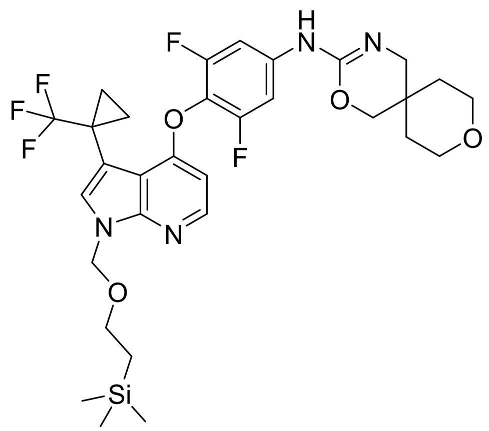

To a stirred solution of N-{3,5-difluoro-4-[(3-[1-(trifluoromethyl)cyclopropyl]-1-{[2-(trimethylsilyl)ethoxy]methyl}-1H-pyrrolo[2,3-b]pyridin-4-yl)oxy]phenyl}-N'-{[4-(hydroxymethyl)oxan-4-yl]methyl}thiourea (200 mg, 0.30 mmol, intermediate **49**) in acetonitrile (4.0 mL) was added 1-(3-Dimethylaminopropyl)-3-ethylcarbodiimide hydrochloride (112 mg, 582 µmol) and triethylamine (122 µl, 726 µmol). The resulting mixture was stirred at 40°C overnight at which time water and ethyl acetate were added and the layers separated. The aqueous phase was extracted twice with ethyl acetate and the combined organic layers were washed with brine, dried over sodium sulfate, filtered and evaporated to afford the crude product which was used without further purification.

Method 1, UPLC-MS (ESI+): t_R = 1.59 min; *m/z* calcd for $C_{31}H_{37}F_5N_4O_4Si$ [M + H]$^+$: 653.3; found: 654

Compound 38 (BAY-405)

N-[3,5-difluoro-4-({3-[1-(trifluoromethyl)cyclopropyl]-1H-pyrrolo[2,3-b]pyridin-4-yl}oxy)phenyl]-2,9-dioxa-4-azaspiro[5.5]undec-3-en-3-amine

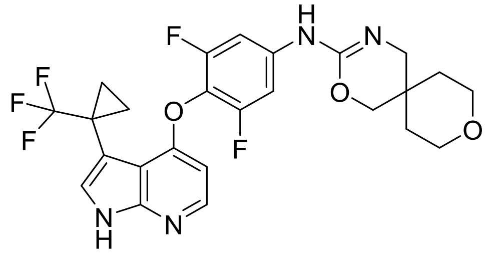

To a solution of N-{3,5-difluoro-4-[(3-[1-(trifluoromethyl)cyclopropyl]-1-{[2-(trimethylsilyl)ethoxy]methyl}-1H-pyrrolo[2,3-b]pyridin-4-yl)oxy]phenyl}-2,9-dioxa-4-azaspiro[5.5]undec-3-en-3-amine (190 mg, 291 μmol, intermediate **50**) in dichloromethane (2.0 mL) was added trifluoroacetic acid (1.0 mL). The reaction mixture was stirred at room temperature overnight, at which time the mixture was basified to pH >10 with 2M sodium hydroxide and ethyl acetate was added. The layers were separated, and the aqueous layer was extracted three times with ethyl acetate. The combined organic layers were washed with brine, dried over sodium sulfate, filtered, and evaporated. The crude material was dissolved in acetonitrile (10 mL) and treated with a 25% aqueous solution of ammonia (5 mL). The resulting solution was stirred for 1 hour and then purified by preparative HPLC to afford the title compound **38** (BAY-405) (38 mg, 24% yield).

Method 1, UPLC-MS (ESI+): t_R = 1.16 min; *m/z* calcd for $C_{25}H_{23}F_5N_4O_3$ [M + H]$^+$: 523.2; found: 523.5

^{1}H NMR (400 MHz, DMSO-d6) δ ppm 1.18 (br s, 2 H), 1.31 - 1.35 (m, 2 H), 1.44 (br s, 4 H), 3.28 (br d, 2 H), 3.54 - 3.67 (m, 4 H), 4.07 (br s, 2 H), 6.26 (d, 1 H), 7.53 (s, 1 H), 7.57 (br s, 1 H), 8.04 (d, 1 H), 9.03 (br s, 1 H), 11.91 (s, 1 H)

^{13}C NMR (DMSO-d_6, 126 MHz) δ 157.7, 155.6, 155.5, 153.6, 153.6, 150.4, 144.2, 127.4, 126.8, 125.2, 121.7, 108.6, 107.7, 98.8, 78.8, 78.7, 78.5, 78.3, 71.8, 62.2, 40.4, 40.1, 39.9, 39.8, 39.6, 39.4, 31.1, 28.1, 19.9, 19.7, 10.6

HPLC-MS Trace for Compound 38 (BAY-405)

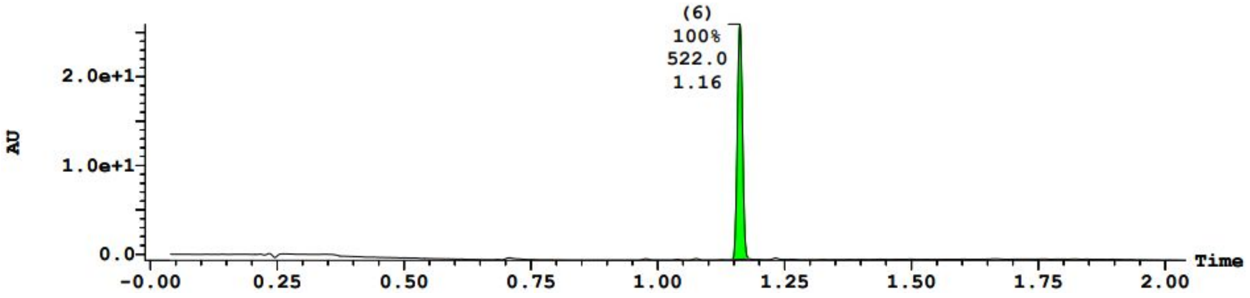

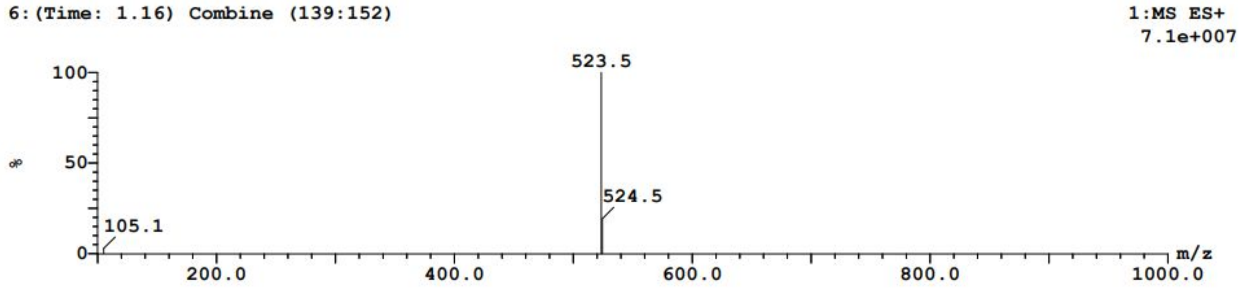

References

1. Alzabin, S.; Pyarajan, S.; Yee, H.; Kiefer, F.; Suzuki, A.; Burakoff, S.; Sawasdikosol, S., Hematopoietic progenitor kinase 1 is a critical component of prostaglandin E2-mediated suppression of the anti-tumor immune response. *Cancer Immunol Immunother* **2010,** *59* (3), 419-29.
2. Chuang, H. C.; Wang, X.; Tan, T. H., MAP4K Family Kinases in Immunity and Inflammation. *Adv Immunol* **2016,** *129*, 277-314.
3. Di Bartolo, V.; Montagne, B.; Salek, M.; Jungwirth, B.; Carrette, F.; Fourtane, J.; Sol-Foulon, N.; Michel, F.; Schwartz, O.; Lehmann, W. D.; Acuto, O., A novel pathway down-modulating T cell activation involves HPK-1-dependent recruitment of 14-3-3 proteins on SLP-76. *J Exp Med* **2007,** *204* (3), 681-91.
4. Hernandez, S.; Qing, J.; Thibodeau, R. H.; Du, X.; Park, S.; Lee, H. M.; Xu, M.; Oh, S.; Navarro, A.; Roose-Girma, M.; Newman, R. J.; Warming, S.; Nannini, M.; Sampath, D.; Kim, J. M.; Grogan, J. L.; Mellman, I., The Kinase Activity of Hematopoietic Progenitor Kinase 1 Is Essential for the Regulation of T Cell Function. *Cell Rep* **2018,** *25* (1), 80-94.
5. Sawasdikosol, S.; Zha, R.; Yang, B.; Burakoff, S., HPK1 as a novel target for cancer immunotherapy. *Immunol Res* **2012,** *54* (1-3), 262-5.
6. Shui, J. W.; Boomer, J. S.; Han, J.; Xu, J.; Dement, G. A.; Zhou, G.; Tan, T. H., Hematopoietic progenitor kinase 1 negatively regulates T cell receptor signaling and T cell-mediated immune responses. *Nat Immunol* **2007,** *8* (1), 84-91.
7. Knippschild, U.; Kruger, M.; Richter, J.; Xu, P.; Garcia-Reyes, B.; Peifer, C.; Halekotte, J.; Bakulev, V.; Bischof, J., The CK1 Family: Contribution to Cellular Stress Response and Its Role in Carcinogenesis. *Front Oncol* **2014,** *4*, 96.
8. Meng, Q. J.; Maywood, E. S.; Bechtold, D. A.; Lu, W. Q.; Li, J.; Gibbs, J. E.; Dupre, S. M.; Chesham, J. E.; Rajamohan, F.; Knafels, J.; Sneed, B.; Zawadzke, L. E.; Ohren, J. F.; Walton, K. M.; Wager, T. T.; Hastings, M. H.; Loudon, A. S., Entrainment of disrupted circadian behavior through inhibition of casein kinase 1 (CK1) enzymes. *Proc Natl Acad Sci U S A* **2010,** *107* (34), 15240-5.
9. Qiao, Y.; Chen, T.; Yang, H.; Chen, Y.; Lin, H.; Qu, W.; Feng, F.; Liu, W.; Guo, Q.; Liu, Z.; Sun, H., Small molecule modulators targeting protein kinase CK1 and CK2. *Eur J Med Chem* **2019,** *181*, 111581.
10. Duan, Q.; Ye, J.; Shi, M.; Chen, W.; Zhu, F., Research advances in the molecular functions and relevant diseases of TAOKs, novel STE20 kinase family members. *Curr Pharm Des* **2020**.
11. Zhang, Z.; Tang, Z.; Ma, X.; Sun, K.; Fan, L.; Fang, J.; Pan, J.; Wang, X.; An, H.; Zhou, J., TAOK1 negatively regulates IL-17-mediated signaling and inflammation. *Cell Mol Immunol* **2018,** *15* (8), 794-802.
12. Coppola, V.; Barrick, C. A.; Southon, E. A.; Celeste, A.; Wang, K.; Chen, B.; Haddad el, B.; Yin, J.; Nussenzweig, A.; Subramaniam, A.; Tessarollo, L., Ablation of TrkA function in the immune system causes B cell abnormalities. *Development* **2004,** *131* (20), 5185-95.
13. Yan, W.; Lakkaniga, N. R.; Carlomagno, F.; Santoro, M.; McDonald, N. Q.; Lv, F.; Gunaganti, N.; Frett, B.; Li, H. Y., Insights into Current Tropomyosin Receptor Kinase (TRK) Inhibitors: Development and Clinical Application. *J Med Chem* **2019,** *62* (4), 1731-1760.
14. Cook, R. S.; Jacobsen, K. M.; Wofford, A. M.; DeRyckere, D.; Stanford, J.; Prieto, A. L.; Redente, E.; Sandahl, M.; Hunter, D. M.; Strunk, K. E.; Graham, D. K.; Earp, H. S., 3rd, MerTK inhibition in tumor leukocytes decreases tumor growth and metastasis. *J Clin Invest* **2013,** *123* (8), 3231-42.
15. Huelse, J.; Fridlyand, D.; Earp, S.; DeRyckere, D.; Graham, D. K., MERTK in cancer therapy: Targeting the receptor tyrosine kinase in tumor cells and the immune system. *Pharmacol Ther* **2020**, 107577.
16. Zhou, Y.; Fei, M.; Zhang, G.; Liang, W. C.; Lin, W.; Wu, Y.; Piskol, R.; Ridgway, J.; McNamara, E.; Huang, H.; Zhang, J.; Oh, J.; Patel, J. M.; Jakubiak, D.; Lau, J.; Blackwood, B.; Bravo, D. D.; Shi, Y.; Wang, J.; Hu, H. M.; Lee, W. P.; Jesudason, R.; Sangaraju, D.; Modrusan, Z.; Anderson, K. R.; Warming, S.; Roose-

Girma, M.; Yan, M., Blockade of the Phagocytic Receptor MerTK on Tumor-Associated Macrophages Enhances P2X7R-Dependent STING Activation by Tumor-Derived cGAMP. *Immunity* **2020,** *52* (2), 357-373 e9.
17. Perez-Pinera, P.; Garcia-Suarez, O.; Germana, A.; Diaz-Esnal, B.; de Carlos, F.; Silos-Santiago, I.; del Valle, M. E.; Cobo, J.; Vega, J. A., Characterization of sensory deficits in TrkB knockout mice. *Neurosci Lett* **2008,** *433* (1), 43-7.
18. Tessarollo, L.; Tsoulfas, P.; Donovan, M. J.; Palko, M. E.; Blair-Flynn, J.; Hempstead, B. L.; Parada, L. F., Targeted deletion of all isoforms of the trkC gene suggests the use of alternate receptors by its ligand neurotrophin-3 in neuronal development and implicates trkC in normal cardiogenesis. *Proc Natl Acad Sci U S A* **1997,** *94* (26), 14776-81.
19. Howe, D. G.; Wiley, J. C.; McKnight, G. S., Molecular and behavioral effects of a null mutation in all PKA C beta isoforms. *Mol Cell Neurosci* **2002,** *20* (3), 515-24.
20. Amoussou, N. G.; Bigot, A.; Roussakis, C.; Robert, J. H., Haspin: a promising target for the design of inhibitors as potent anticancer drugs. *Drug Discov Today* **2018,** *23* (2), 409-415.
21. Eswaran, J.; Patnaik, D.; Filippakopoulos, P.; Wang, F.; Stein, R. L.; Murray, J. W.; Higgins, J. M.; Knapp, S., Structure and functional characterization of the atypical human kinase haspin. *Proc Natl Acad Sci U S A* **2009,** *106* (48), 20198-203.
22. Karanika, E.; Soupsana, K.; Christogianni, A.; Stellas, D.; Klinakis, A.; Politou, A. S.; Georgatos, S., Haspin-dependent and independent effects of the kinase inhibitor 5-Iodotubercidin on self-renewal and differentiation. *Sci Rep* **2020,** *10* (1), 232.
23. Shimada, M.; Goshima, T.; Matsuo, H.; Johmura, Y.; Haruta, M.; Murata, K.; Tanaka, H.; Ikawa, M.; Nakanishi, K.; Nakanishi, M., Essential role of autoactivation circuitry on Aurora B-mediated H2AX-pS121 in mitosis. *Nat Commun* **2016,** *7*, 12059.
24. Haiko, P.; Makinen, T.; Keskitalo, S.; Taipale, J.; Karkkainen, M. J.; Baldwin, M. E.; Stacker, S. A.; Achen, M. G.; Alitalo, K., Deletion of vascular endothelial growth factor C (VEGF-C) and VEGF-D is not equivalent to VEGF receptor 3 deletion in mouse embryos. *Mol Cell Biol* **2008,** *28* (15), 4843-50.
25. Hsu, M. C.; Pan, M. R.; Hung, W. C., Two Birds, One Stone: Double Hits on Tumor Growth and Lymphangiogenesis by Targeting Vascular Endothelial Growth Factor Receptor 3. *Cells* **2019,** *8* (3).
26. Apte, R. S.; Chen, D. S.; Ferrara, N., VEGF in Signaling and Disease: Beyond Discovery and Development. *Cell* **2019,** *176* (6), 1248-1264.
27. Corkery, D. P.; Holly, A. C.; Lahsaee, S.; Dellaire, G., Connecting the speckles: Splicing kinases and their role in tumorigenesis and treatment response. *Nucleus* **2015,** *6* (4), 279-88.
28. Hatting, M.; Rines, A. K.; Luo, C.; Tabata, M.; Sharabi, K.; Hall, J. A.; Verdeguer, F.; Trautwein, C.; Puigserver, P., Adipose Tissue CLK2 Promotes Energy Expenditure during High-Fat Diet Intermittent Fasting. *Cell Metab* **2017,** *25* (2), 428-437.
29. Quaresma, P. G.; Weissmann, L.; Zanotto, T. M.; Santos, A. C.; de Matos, A. H.; Furigo, I. C.; Simabuco, F. M.; Donato, J., Jr.; Bittencourt, J. C.; Lopes-Cendes, I.; Prada, P. O., Cdc2-like kinase 2 in the hypothalamus is necessary to maintain energy homeostasis. *Int J Obes (Lond)* **2017,** *41* (2), 268-278.
30. Tabata, M.; Rodgers, J. T.; Hall, J. A.; Lee, Y.; Jedrychowski, M. P.; Gygi, S. P.; Puigserver, P., Cdc2-like kinase 2 suppresses hepatic fatty acid oxidation and ketogenesis through disruption of the PGC-1alpha and MED1 complex. *Diabetes* **2014,** *63* (5), 1519-32.
31. Walter, A.; Chaikuad, A.; Helmer, R.; Loaec, N.; Preu, L.; Ott, I.; Knapp, S.; Meijer, L.; Kunick, C., Molecular structures of cdc2-like kinases in complex with a new inhibitor chemotype. *PLoS One* **2018,** *13* (5), e0196761.
32. Wang, J.; Ji, X.; Liu, J.; Zhang, X., Serine/Threonine Protein Kinase STK16. *Int J Mol Sci* **2019,** *20* (7).
33. Bian, Y.; Teper, Y.; Mathews Griner, L. A.; Aiken, T. J.; Shukla, V.; Guha, R.; Shinn, P.; Xin, H. W.; Pflicke, H.; Powers, A. S.; Li, D.; Jiang, J. K.; Patel, P.; Rogers, S. A.; Aube, J.; Ferrer, M.; Thomas, C. J.; Rudloff, U., Target Deconvolution of a Multikinase Inhibitor with Antimetastatic Properties Identifies

TAOK3 as a Key Contributor to a Cancer Stem Cell-Like Phenotype. *Mol Cancer Ther* **2019,** *18* (11), 2097-2110.
34. Hammad, H.; Vanderkerken, M.; Pouliot, P.; Deswarte, K.; Toussaint, W.; Vergote, K.; Vandersarren, L.; Janssens, S.; Ramou, I.; Savvides, S. N.; Haigh, J. J.; Hendriks, R.; Kopf, M.; Craessaerts, K.; de Strooper, B.; Kearney, J. F.; Conrad, D. H.; Lambrecht, B. N., Transitional B cells commit to marginal zone B cell fate by Taok3-mediated surface expression of ADAM10. *Nat Immunol* **2017,** *18* (3), 313-320.
35. Ormonde, J. V. S.; Li, Z.; Stegen, C.; Madrenas, J., TAOK3 Regulates Canonical TCR Signaling by Preventing Early SHP-1-Mediated Inactivation of LCK. *J Immunol* **2018,** *201* (11), 3431-3442.
36. Bela, S. R.; Dutra, M. S.; Mui, E.; Montpetit, A.; Oliveira, F. S.; Oliveira, S. C.; Arantes, R. M.; Antonelli, L. R.; McLeod, R.; Gazzinelli, R. T., Impaired innate immunity in mice deficient in interleukin-1 receptor-associated kinase 4 leads to defective type 1 T cell responses, B cell expansion, and enhanced susceptibility to infection with Toxoplasma gondii. *Infect Immun* **2012,** *80* (12), 4298-308.
37. Jain, A.; Kaczanowska, S.; Davila, E., IL-1 Receptor-Associated Kinase Signaling and Its Role in Inflammation, Cancer Progression, and Therapy Resistance. *Front Immunol* **2014,** *5*, 553.
38. Suzuki, N.; Suzuki, S.; Duncan, G. S.; Millar, D. G.; Wada, T.; Mirtsos, C.; Takada, H.; Wakeham, A.; Itie, A.; Li, S.; Penninger, J. M.; Wesche, H.; Ohashi, P. S.; Mak, T. W.; Yeh, W. C., Severe impairment of interleukin-1 and Toll-like receptor signalling in mice lacking IRAK-4. *Nature* **2002,** *416* (6882), 750-6.
39. Della Mina, E.; Borghesi, A.; Zhou, H.; Bougarn, S.; Boughorbel, S.; Israel, L.; Meloni, I.; Chrabieh, M.; Ling, Y.; Itan, Y.; Renieri, A.; Mazzucchelli, I.; Basso, S.; Pavone, P.; Falsaperla, R.; Ciccone, R.; Cerbo, R. M.; Stronati, M.; Picard, C.; Zuffardi, O.; Abel, L.; Chaussabel, D.; Marr, N.; Li, X.; Casanova, J. L.; Puel, A., Inherited human IRAK-1 deficiency selectively impairs TLR signaling in fibroblasts. *Proc Natl Acad Sci U S A* **2017,** *114* (4), E514-E523.
40. Yang, D.; Chen, W.; Xiong, J.; Sherrod, C. J.; Henry, D. H.; Dittmer, D. P., Interleukin 1 receptor-associated kinase 1 (IRAK1) mutation is a common, essential driver for Kaposi sarcoma herpesvirus lymphoma. *Proc Natl Acad Sci U S A* **2014,** *111* (44), E4762-8.
41. Matschinsky, F. M.; Wilson, D. F., The Central Role of Glucokinase in Glucose Homeostasis: A Perspective 50 Years After Demonstrating the Presence of the Enzyme in Islets of Langerhans. *Front Physiol* **2019,** *10*, 148.
42. Unbekandt, M.; Olson, M. F., The actin-myosin regulatory MRCK kinases: regulation, biological functions and associations with human cancer. *J Mol Med (Berl)* **2014,** *92* (3), 217-25.
43. Zhao, Z.; Manser, E., Myotonic dystrophy kinase-related Cdc42-binding kinases (MRCK), the ROCK-like effectors of Cdc42 and Rac1. *Small GTPases* **2015,** *6* (2), 81-8.
44. Condorelli, F.; Stec-Martyna, E.; Zaborowska, J.; Felli, L.; Gnemmi, I.; Ponassi, M.; Rosano, C., Role of the non-receptor tyrosine kinase fes in cancer. *Curr Med Chem* **2011,** *18* (19), 2913-20.
45. Greer, P. A.; Kanda, S.; Smithgall, T. E., The contrasting oncogenic and tumor suppressor roles of FES. *Front Biosci (Schol Ed)* **2012,** *4*, 489-501.
46. Koch, J. P.; Aebersold, D. M.; Zimmer, Y.; Medova, M., MET targeting: time for a rematch. *Oncogene* **2020,** *39* (14), 2845-2862.
47. Zhang, H.; Pao, L. I.; Zhou, A.; Brace, A. D.; Halenbeck, R.; Hsu, A. W.; Bray, T. L.; Hestir, K.; Bosch, E.; Lee, E.; Wang, G.; Liu, H.; Wong, B. R.; Kavanaugh, W. M.; Williams, L. T., Deorphanization of the human leukocyte tyrosine kinase (LTK) receptor by a signaling screen of the extracellular proteome. *Proc Natl Acad Sci U S A* **2014,** *111* (44), 15741-5.
48. Babina, I. S.; Turner, N. C., Advances and challenges in targeting FGFR signalling in cancer. *Nat Rev Cancer* **2017,** *17* (5), 318-332.
49. Brewer, J. R.; Molotkov, A.; Mazot, P.; Hoch, R. V.; Soriano, P., Fgfr1 regulates development through the combinatorial use of signaling proteins. *Genes Dev* **2015,** *29* (17), 1863-74.
50. Akerblom, B.; Anneren, C.; Welsh, M., A role of FRK in regulation of embryonal pancreatic beta cell formation. *Mol Cell Endocrinol* **2007,** *270* (1-2), 73-8.

51. Goel, R. K.; Lukong, K. E., Understanding the cellular roles of Fyn-related kinase (FRK): implications in cancer biology. *Cancer Metastasis Rev* **2016,** *35* (2), 179-99.
52. McDonald, I. M.; Graves, L. M., Enigmatic MELK: The controversy surrounding its complex role in cancer. *J Biol Chem* **2020,** *295* (24), 8195-8203.
53. Wang, Y.; Lee, Y. M.; Baitsch, L.; Huang, A.; Xiang, Y.; Tong, H.; Lako, A.; Von, T.; Choi, C.; Lim, E.; Min, J.; Li, L.; Stegmeier, F.; Schlegel, R.; Eck, M. J.; Gray, N. S.; Mitchison, T. J.; Zhao, J. J., MELK is an oncogenic kinase essential for mitotic progression in basal-like breast cancer cells. *Elife* **2014,** *3*, e01763.
54. Klaeger, S.; Heinzlmeir, S.; Wilhelm, M.; Polzer, H.; Vick, B.; Koenig, P. A.; Reinecke, M.; Ruprecht, B.; Petzoldt, S.; Meng, C.; Zecha, J.; Reiter, K.; Qiao, H.; Helm, D.; Koch, H.; Schoof, M.; Canevari, G.; Casale, E.; Depaolini, S. R.; Feuchtinger, A.; Wu, Z.; Schmidt, T.; Rueckert, L.; Becker, W.; Huenges, J.; Garz, A. K.; Gohlke, B. O.; Zolg, D. P.; Kayser, G.; Vooder, T.; Preissner, R.; Hahne, H.; Tonisson, N.; Kramer, K.; Gotze, K.; Bassermann, F.; Schlegl, J.; Ehrlich, H. C.; Aiche, S.; Walch, A.; Greif, P. A.; Schneider, S.; Felder, E. R.; Ruland, J.; Medard, G.; Jeremias, I.; Spiekermann, K.; Kuster, B., The target landscape of clinical kinase drugs. *Science* **2017,** *358* (6367).
55. Banker, M. J.; Clark, T. H.; Williams, J. A., Development and validation of a 96-well equilibrium dialysis apparatus for measuring plasma protein binding. *J Pharm Sci* **2003,** *92* (5), 967-74.
56. Schirok, H.; Kast, R.; Figueroa-Perez, S.; Bennabi, S.; Gnoth, M. J.; Feurer, A.; Heckroth, H.; Thutewohl, M.; Paulsen, H.; Knorr, A.; Hutter, J.; Lobell, M.; Munter, K.; Geiss, V.; Ehmke, H.; Lang, D.; Radtke, M.; Mittendorf, J.; Stasch, J. P., Design and synthesis of potent and selective azaindole-based Rho kinase (ROCK) inhibitors. *ChemMedChem* **2008,** *3* (12), 1893-904.
57. Schirok, H., Synthesis and Derivatization of 3-Perfluoroalkyl-Substituted 7-Azaindoles. *Synthesis* **2007,** *2*, 251-258.
58. Schirok, H., Improved Synthesis of the Selective Rho-Kinase Inhibitor 6-Chloro-N4-{3,5-difluoro-4-[(3-methyl-1H-pyrrolo[2,3-b]pyridin-4-yl)oxy]phenyl}pyrimidin-2,4-diamine. *Organic Process Research & Development* **2010,** *14* (1), 168-173.
59. Phelan, J. P.; Lang, S. B.; Compton, J. S.; Kelly, C. B.; Dykstra, R.; Gutierrez, O.; Molander, G. A., Redox-Neutral Photocatalytic Cyclopropanation via Radical/Polar Crossover. *J Am Chem Soc* **2018,** *140* (25), 8037-8047.
